# Supplementary material for: Statins for extension of disability-free survival and primary prevention of cardiovascular events among older people: protocol for a randomised controlled trial in primary care (STAREE trial)
Source: BMJ Open. 2023 Apr 3;13(4):e069915. doi: 10.1136/bmjopen-2022-069915 (PMC10083753; doi:10.1136/bmjopen-2022-069915)
Supplement: Supplementary data [file bmjopen-2022-069915supp001.pdf]

Appendix 1. WHO Trial Registration Data Set

	Data Category	Information
1	Primary Registry and Trial Identifying Number	ClinicalTrials.gov<br>NCT02099123
2	Date of Registration in Primary Registry	28 March 2014
3	Secondary Identifying Numbers	
4	Source(s) of Monetary or Material Support	National Health and Medical Research Council of Australia<br>Heart Foundation (Australia)
5	Primary Sponsor	Monash University
6	Secondary Sponsor(s)	Not applicable
7	Contact for Public Queries	staree@monash.edu
8	Contact for Scientific Queries	Professor Sophia Zoungas (Principal Investigator)<br>sophia.zoungas@monash.edu<br>Phone: +61 (3) 9903 0711<br>Address: Level 5/99 Commercial Rd, Melbourne 3004
9	Public Title	STAtin therapy for Reducing Events in the Elderly (STAREE) trial
10	Scientific Title	A randomised clinical trial of STAtin therapy for Reducing Events in the Elderly
11	Countries of Recruitment	Australia
12	Health Condition(s) or Problem(s) Studied	Prevention of cardiovascular disease, dementia and disability
13	Intervention(s)	Atorvastatin (2 x 20 mg/day)
		Identical Placebo (2 tablets/day)
14	Key Inclusion and Exclusion Criteria	Inclusion criteria: Age ≥70 years, living independently in the community, willing and able to provide informed consent and accept the study requirements
		Exclusion criteria: History of clinical cardiovascular events, diabetes or dementia; Modified Mini-Mental State Examination (3MS) score <78 on screening; moderate or severe chronic kidney disease; moderate or severe liver disease; serious inter-current illness likely to cause death within the next 5 years; total cholesterol >7.5 mmol/L; current participation in another interventional clinical trial; and an absolute contraindication to statin therapy; current use of statin therapy or other lipid lowering therapy for primary prevention and unwilling to stop therapy; or current long term or permanent use of potent cytochrome P450 (CYP) 3A4 inhibitors.
15	Study Type	Interventional
		Allocation: randomised
		Double blind
		Primary purpose: Phase IV study
16	Date of First Enrolment	November 2015
17	Sample Size	9631
18	Recruitment Status	Recruiting

19	Primary Outcome(s)	i) a composite of cardiovascular death, non-fatal myocardial infarction, or non-fatal stroke; and<br>ii) a composite of all-cause death or dementia or development of persistent physical disability.
20	Key Secondary Outcomes	All cause death, dementia, other cognitive impairment, persistent physical disability, fatal and non-fatal myocardial infarction, fatal and non-fatal stroke, cardiovascular death, fatal and non-fatal cancer, heart failure hospitalisation, atrial fibrillation, all-cause hospitalisation, need for permanent residential care, quality of life
21	Ethics Review	Monash University Human Research Ethics Committee (Project ID 2787 and 21528)<br>Curtin University Human Research Ethics Committee (HR113/2015)<br>RACGP National Research and Evaluation Ethics Committee (14 - 017)<br>Tasmanian Health and Medical Research Ethics Committee (H0014918)<br>University of Newcastle Human Research Ethics Committee (H-2016-0266)
22	Completion date	Anticipated end 2025
23	Summary Results	Not applicable
24	IPD sharing statement	Requests for access to de-identified data (to be provided through a secure online environment) may be submitted to the researchers located at the School of Public Health and Preventive Medicine, Monash University, Melbourne, Australia.
