# Supplementary material for: Statins for extension of disability-free survival and primary prevention of cardiovascular events among older people: protocol for a randomised controlled trial in primary care (STAREE trial)
Source: BMJ Open. 2023 Apr 3;13(4):e069915. doi: 10.1136/bmjopen-2022-069915 (PMC10083753; doi:10.1136/bmjopen-2022-069915)
Supplement: Supplementary data [file bmjopen-2022-069915supp002.pdf]

A randomised clinical trial of STAtin therapy for Reducing Events in the Elderly (STAREE)

PROTOCOL

Version 3.1

Protocol authors:

Professor Sophia Zoungas, Dr Andrea Curtis, Dr Simone Spark, Professor Rory Wolfe

Affiliation:

School of Public Health and Preventive Medicine, Monash University, VIC, Australia

Contact for public enquiries

Phone: 1800 770 664

Email: staree@monash.edu

Fax: +61 (3) 9903 0737

Contact for scientific enquiries

Professor Sophia Zoungas (Principal Investigator)

sophia.zoungas@monash.edu

Phone: +61 (3) 9903 0711

Address: Level 5/99 Commercial Rd, Melbourne 3004

Recruitment Status: Recruiting

Zoungas S, *et al. BMJ Open* 2023; 13:e069915. doi: 10.1136/bmjopen-2022-069915

Acronyms used in this protocol:

ABS: Australian Bureau of Statistics

ACAP: Aged-care assessment dataset

ACR: Albumin to creatinine ratio

ADR: Adverse Drug Reaction

ADCS-IADL: Alzheimer's Disease Cooperative Study Instrumental Activities of Daily Living

AE: Adverse events

ASPREE: ASPirin in Reducing Events in the Elderly

CAM: Confusion Assessment Method

CES-D-10: Centre for Epidemiologic Studies Depression Scale – 10 items

CERAD: Consortium to Establish a Registry for Alzheimer's Disease

COWAT: Controlled Oral Word Association Test

CRF: Case Report Form

DMC: Data Management Committee

DOHA: Department of Health and Ageing

DSMB: Data Safety and Monitoring Board

EAC: End-point Adjudication Committee

GCP: Good Clinical Practice

HVLT-R: Hopkins Verbal Learning Test – Revised

HDL: High Density Lipoprotein

HIPAA: Health Insurance Portability and Accountability Act of 1996

IDL: Intermediate Density Lipoprotein

IPAQ-E: International Physical Activity Questionnaire modified for the elderly

LDL: Low Density Lipoprotein

MI: Myocardial Infarction

NCD : Neurocognitive Disorder

SAE: Serious Adverse Event

SDMT: Symbol Digit Modalities Test

SOP: Standard Operating Procedure

STAREE: STAtin therapy for Reducing Events in the Elderly

3MS: Modified Mini-Mental State test

TICS-M: Telephone Interview of Cognitive Status – modified version

VLDL: Very Low Density Lipoprotein

Zoungas S, et al. BMJ Open 2023; 13:e069915. doi: 10.1136/bmjopen-2022-069915

REVISIONS

No.	Changes
Version 1.2.2	Original approved version
Version 1.3	Changes to Study Activity Schedule including addition of cognitive tests, CESD10, and biobank sample collection
Version 1.3.1	SF-12 changed to SF-36; Biobank Steering Committee added to Governance structure
Version 1.3.2	Study medication: change to post out details and addition of medication compliance questionnaire
Version 1.3.3	Addition of International Steering Committee; Updated exclusion criteria regarding cytochrome P450 3A4 inhibitors; Change in biobank study samples collected;
Version 2.0	Updates to align protocol with SPIRIT 2013 statement recommendations
	Updates and clarification of primary and secondary endpoints; including change in dementia definition from DSM IV to DSM V
	Updated exclusion criteria regarding cytochrome P450 3A4 inhibitors
Version 3.0	Response to COVID-19 – schedule update
	Changes to power calculations, sample size and follow up period
Version 3.1	Addition of Frailty phenotype and functional independence as tertiary endpoints

Zoungas S, *et al. BMJ Open* 2023; 13:e069915. doi: 10.1136/bmjopen-2022-069915

Contents

Contents ... 4
1. Executive Summary ... 8
2. Introduction ... 9
2.1 Rationale ... 9
2.2 Statins ... 10
2.3 Other health outcomes ... 12
2.4 Statins in the Elderly: potential risks ... 12
2.4.1 Myopathy ... 12
2.4.2 Diabetes ... 13
2.4.3 Cancer ... 13
2.4.4 Cognitive impairment ... 13
2.4.5 Liver Injury ... 13
2.5 Utilisation ... 14
2.6 Implications of Guidelines on Treatment of Cholesterol in the Elderly ... 14
2.7 Summary of rationale ... 16
3. Study Objectives ... 17
3.1 Primary objectives ... 17
3.2 Secondary objectives ... 17
3.3 Tertiary objectives ... 17
4. Hypotheses ... 18
4.1 Primary null hypotheses ... 18
4.2 Primary alternative hypotheses ... 18
5. Study Design ... 19
5.1 Trial Design ... 19
5.2 Study Population ... 19
5.2.1 General considerations ... 19
5.2.2 Inclusion criteria ... 19
5.2.3 Exclusion criteria ... 19
5.2.4 Rescreening or reassessment ... 20
5.3 Study Intervention and Choice of Comparator ... 20
5.3.1 Intervention ... 20
5.3.2 Rationale for choice of atorvastatin ... 20
5.4 Participant recruitment ... 21

Zoungas S, *et al. BMJ Open* 2023; 13:e069915. doi: 10.1136/bmjopen-2022-069915

5.4.1 Recruitment 21
5.4.2 Setting 21
5.4.3 Community participants 22
5.4.4 STAREE hub locations 22
5.5 Participant timeline and schedule of assessments 22
5.6 Baseline Screening Phase 22
5.6.1 Screening call 22
5.6.2 Lifestyle profile and screening visits (Baseline visits) 26
5.6.3 Post screening GP/medical visit (Run-in phase between Baseline visits 1 and 2) . 26
5.6.4 Post screening visit study activity (Run-in phase between Baseline visits 1 and 2) 26
5.6.5 Determinations of Eligibility and Assessments visit (Baseline Visit 2) 26
5.7 Randomisation and follow up phase 27
5.7.1 Randomisation procedure 27
5.7.2 Post randomisation procedure 27
5.7.3 Blinding 28
5.7.4 Annual Follow up visit 28
5.7.5 Phone Contact 28
5.7.6 Other Actions 29
5.8 Economic Analysis 29
6. Study Endpoints 29
6.1 Primary endpoints 29
6.2 Secondary endpoints 30
6.3 Tertiary endpoints 30
6.3 Endpoint ascertainment 30
6.4 Endpoint adjudication process 31
7. Sub-studies and ancillary studies 31
7.1 STAREE-HEART 31
7.3 STAREE Healthy Ageing Biobank 32
8. Contingency plans for participant wellbeing 33
8.1 Commencement of regular lipid lowering agents 33
8.2 Development of an indication for statins 33
8.3 Post-trial care 33
9. Levels of participant involvement 34
9.1 Levels of participant involvement 34
9.2 Withdrawal 35

Zoungas S, *et al. BMJ Open* 2023; 13:e069915. doi: 10.1136/bmjopen-2022-069915

10. Study Medication and Supplies 36
10.1 Study treatments 36
10.2 Administration of study medication 36
10.3 Dispensing and randomisation 36
10.4 Study medication compliance 37
10.5 Study medication storage 37
10.6 Study medication accountability and dispensing logs 38
10.7 Study medication disposal 38
10.8 Overdose of study medication 38
11. Concomitant Therapy 39
11.1 Prescription medications 39
12. Sample Size and Power calculations 40
13. Safety Monitoring and Reporting 44
13.1 Introduction 44
13.2 Safety Monitoring 44
13.2.2 Adverse events (AE) 44
13.2.3 Serious adverse events (SAE) 44
13.2.4 Suspected Unexpected Serious Adverse Events (SUSARs) 45
13.3 Safety reporting 46
13.4 Adverse Events of Interest 46
13.4.1 New diagnosis of diabetes 46
13.4.2 New diagnosis of myopathy 46
13.4.3 Liver impairment 47
14. Adherence to Ethical, Regulatory and Administrative Considerations 48
14.1 Ethical considerations 48
14.1.1 General 48
14.1.2 Ethics Committee Approval 48
14.1.3 Information for participants 48
14.1.4 Informed consent 48
14.1.5 Protocol Amendments 48
14.1.6 Confidentiality 49
14.2 Regulatory considerations 49
14.2.1 Financing 49
14.2.2 Trial registration 49
14.2.3 Declaration of interest 49

14.3 Indemnity 49
14.4 Data Access 50
14.5 Governance 50
14.6 Administrative Organisation 51
14.6.1 Individual and committee responsibilities 51
14. Data Management and Training 55
14.1 Case report forms 55
14.2 Data Management 55
14.3 Data analysis 56
14.4 Training 57
14.4.1 Research Officers 57
14.4.2 GP Liaison Officers 57
14.4.3 Research Field Officers 57
15. Study Monitoring and Auditing 58
16. Dissemination Plan 59
17. References 60

Zoungas S, *et al. BMJ Open* 2023; 13:e069915. doi: 10.1136/bmjopen-2022-069915

1. Executive Summary

This protocol describes the background, rationale, study design, processes and governance arrangements for the Statins in Reducing Events in the Elderly (STAREE) trial and its sub-studies.

STAREE is a double blind, randomised placebo-controlled parallel group trial investigating whether statin therapy can prolong good health and maintain independence amongst older people. STAREE will recruit and randomise 9,631 Australian participants aged 70 years and over who are free from cardiovascular disease, diabetes and dementia and living independently in the community.

The STAREE trial will compare the effects of atorvastatin (2 x 20 mg/day) or identical placebo on two co-primary outcomes, a composite of all cause death, dementia or disability and a composite of cardiovascular death, non-fatal myocardial infarction or non-fatal stroke. Secondary outcomes include all cause death, cardiovascular death, dementia, other cognitive impairment, disability, all cause hospitalisation, need for permanent residential care, fatal and non-fatal myocardial infarction, fatal and non-fatal stroke (thromboembolic and haemorrhagic), fatal and non-fatal cancer, heart failure and quality of life. The cost effectiveness of statin therapy for primary prevention in people aged 70 years and over will also be assessed.

2. Introduction

2.1 Rationale

Ageing Population

The developed world is experiencing a rapid demographic transition towards an older population.[1] Increasing survival amongst older age groups is a major contributor to this shift, with almost twice as many older people surviving into their eighties and nineties compared to fifty years ago.[2]

In the US, the proportion of the population aged 65 and over is projected to increase from 40 million in 2009 to 89 million in 2050 with 19 million (20%) in the oldest age group (85+) by 2050.[3] Similar trends are anticipated in other developed nations such as Australia, Canada and the United Kingdom.[2]

As age advances quality of life is increasingly affected by frailty, cognitive decline/dementia and the consequences of a variety of chronic diseases including cardiovascular disease, arthritis, osteoporosis and malignancy. Although preventive therapies have reduced the burden of chronic diseases at younger ages there is limited evidence that these advances have improved the health of older people.[2] As a result, the majority of older individuals ultimately progress to residential care. In Australia, at 75 years of age the remaining lifetime need for residential nursing care is estimated at 72% for women and 53% for men, at a cost of approximately $7 billion Australian dollars per annum.[4]

The gains that have been made in increasing quantity of life have not been accompanied by similar gains in quality of life, for the additional later years of life lived. The critical question is therefore one of the balance of any benefits and harms of preventive therapies for older people.

One promising but inadequately tested preventive approach to extending life and maintaining independence in the elderly is statin therapy. In people with existing cardiovascular disease (secondary prevention), the benefits of statin therapy are clear with the risks of cardiovascular events reduced across all ages. In people without cardiovascular disease (primary prevention), the benefits of taking a statin are not clear for older people. Most national and international guidelines have not been able to make strong recommendations to guide prescribing for older people and any recommendations have generally been informed by trials of statins in younger people (<70 years) or trials of statins that have not studied the range of outcomes important to older people.[5]

Cholesterol and Clinical Outcomes in the Elderly

While high levels of total cholesterol or LDL cholesterol are known to increase the risk of mortality among young and middle-aged populations, the relationship between total cholesterol or LDL cholesterol and all-cause or cause-specific death in older populations is not consistent. Some observational studies have reported no association or a negative association between total cholesterol or LDL cholesterol levels and all-cause death[6-11] whilst others have reported a positive linear association between total cholesterol and LDL cholesterol levels and cardiovascular death.[12] One explanation for the discrepancy is "reverse

Zoungas S, et al. BMJ Open 2023; 13:e069915. doi: 10.1136/bmjopen-2022-069915

causality," whereby unquantified comorbid disease may be causing both lower total cholesterol or LDL cholesterol levels and an increased risk of death, thereby creating an apparent lack of association or negative association.

Given the uncertainty around optimal total and LDL cholesterol levels in the elderly, the decision as to whether statins should be initiated or even continued must be guided by randomised controlled trials.

2.2 Statins

Pharmacological effects

Statins (HMG-CoA reductase inhibitors) are a class of drug with the potential to delay the onset of several causes of incapacity in the elderly. Their best-recognised pharmacological action is to inhibit the mevalonate pathway and biosynthesis of cholesterol and isoprenoids by binding to HMG-CoA reductase and displacing the natural substrate HMG-CoA. This interaction prevents HMG-CoA reductase from attaining a functional conformation, stops the conversion of HMG-CoA to l-mevalonate and produces a reduction in intracellular cholesterol.[13] LDL-receptor gene expression and formation on the hepatocyte cell surface are then induced. The result is an increase in LDL cholesterol extraction (and its precursors, IDL and VLDL) from the blood and decrease in circulating LDL cholesterol levels (by 18-55%).[13] Beneficial effects on other lipid parameters have also been reported including increased HDL cholesterol (by 5-15%),[14] decreased levels of atherogenic lipoproteins, [15] decreased triglyceride concentrations (7-30%) and decreased synthesis and secretion of triglyceride-rich lipoproteins[15] as well as reduced susceptibility of LDL cholesterol to oxidation.[13]

The reduction in circulating lipid levels underpins the ability of statins to reduce the incidence of myocardial infarction and stroke and their long-term sequelae (cardiac failure, disability, cognitive decline). Statins also exert a number of pleiotropic effects including anti-oxidative, anti-thrombotic and a moderately powerful anti-inflammatory action.[16] The pleiotropic effects of statins have been attributed to the inhibition of isoprenoid metabolite formation (geranylgeranyl pyrophosphate and farnesyl pyrophosphate).[13] These metabolites are intermediates in the post-translational formation of several cell-signalling proteins that control multiple cell functions including maintenance of cell shape, motility, factor secretion, differentiation, and proliferation thus explaining improvements in endothelial function, vascular inflammation and oxidation, and atherosclerotic plaque stability.[13]

Clinical effects on Major Vascular Events and Death

The impact of statin therapy on cardiovascular events has been studied in primary (mostly high risk) and secondary prevention settings. A recent meta-analysis of pooled individual participant data from 28 trials of statin versus control/placebo including participants of ages 55 years and over has reported a 21% reduction in risk of major vascular events per 1 mmol/L reduction in LDL cholesterol, attributed to reduced risks of major coronary events, major stroke events and vascular death.[17]

In an analysis of mixed populations (both primary and secondary prevention), there was a trend for decreasing reductions in major vascular events with increasing age. Analyses by age group indicated risk reductions of major vascular events of 19% and 13% per 1 mmol/L

Zoungas S, et al. BMJ Open 2023; 13:e069915. doi: 10.1136/bmjopen-2022-069915

reduction in LDL cholesterol for participants aged >70 to ≤75 and >75 years respectively. This compared to a risk reduction of 25% for participants ≤55 years. [17] The risk of all-cause death was also reduced, albeit to a much lesser extent (9% per 1 mmol/L reduction in LDL cholesterol). No effect on non-vascular deaths was observed. Of note, participants were predominantly high risk, middle-aged men with high baseline LDL cholesterol levels (mean weighted LDL cholesterol level 3.7 mmol/L) or known cardiovascular disease.[17]

In those with cardiovascular disease (secondary prevention), the benefits of statin were clear with risks of major vascular events and all-cause death reduced in people of all ages.[18] Moreover, the number needed to treat was sufficiently low to justify treatment (28 patients would need to be treated for 5 years to prevent one death).[19]

In analyses limited to primary prevention populations, there was a significant trend toward smaller proportional risk reductions as age increased. (Figure 1) Analyses by age group indicated risk reductions of major vascular events of 16% and 8% per 1 mmol/L reduction in LDL cholesterol for participants aged >70 to ≤75 and >75 years respectively. This compared to a risk reduction of 32% for participants ≤55 years.[17] No data was presented on the risk of all-cause death in older people without cardiovascular disease.

Figure 1: Effects of statin therapy on major vascular events (MVE) per mmol/L reduction in LDL cholesterol by age at randomisation in participants without vascular disease. [17]

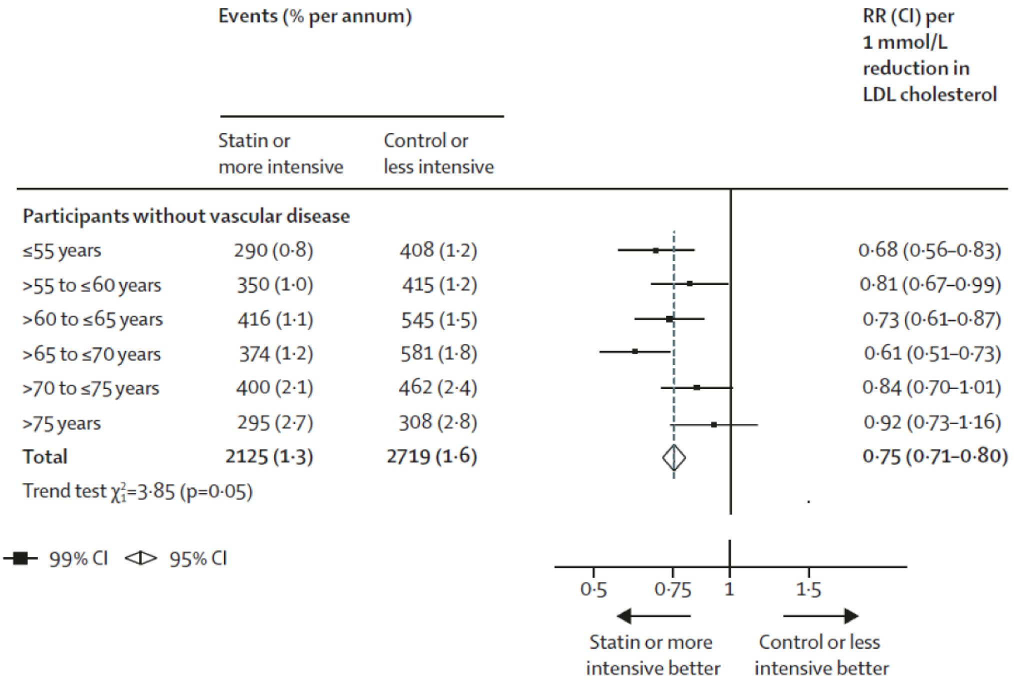

Post hoc subgroup analyses of older participants in statin trials

Pravastatin in the PROspective Study of Pravastatin in the Elderly at Risk (PROSPER) trial, which followed 3,239 people aged 70 years or older for on average 3.2 years, did not

significantly reduce cardiovascular events.[20] Pravastatin in the Antihypertensive and Lipid-Lowering Treatment to Prevent Heart Attack Trial (ALLHAT-LLT) trial which followed 2,867 people 65 years or older for over 4.5 years, did not significantly reduce cardiovascular events or mortality.[21] Rosuvastatin in the Justification for the Use of Statins in Prevention: an Intervention Trial Evaluating Rosuvastatin (JUPITER) trial, which followed 5695 people aged 70 years or older for on average 2 years, significantly reduced cardiovascular events (RRR 39% 95%CI 14 to 57%) but not mortality.[22] Rosuvastatin in the Heart Outcomes Prevention Evaluation (HOPE 3) Trial, which followed 3086 people aged 70 years or older for on average 4.6 years, did not significantly reduce cardiovascular events.[22] A pooling of data from the last 2 trials (JUPITER and HOPE 3) concluded that statins reduced cardiovascular events in those people aged 65-70 years (RRR 49% 95%CI 31 to 62%) and >70 years (RRR 26% 95%CI 9 to 39%).[23]

This demonstrates the degree of uncertainty about the effects of statins in the older age groups. Given the potential for widespread use of statins amongst older people, the limited data emphasises the need for more certainty about the scale of any likely benefit of this class of drugs.

2.3 Other health outcomes

Physical and cognitive function, independence, and health-related quality of life are important health outcomes for older people. These outcomes have not been comprehensively evaluated in previous statin trials.

Statins have been proposed to attenuate long term decline in physical and cognitive function and to preserve independent living by reducing the morbidity associated with coronary and cerebrovascular disease (including subclinical disease) in the elderly.[3]

2.4 Statins in the Elderly: potential risks

The likelihood of adverse effects of drug therapy increases in older people.[24] Much of the knowledge of specific adverse effects comes from spontaneous reports or from trials predominantly studying middle aged people. As with efficacy, the burden of statin adverse effects in older individuals has not been well studied. As a result, it is still not possible to know the balance of risks and benefits associated with the use of these drugs in this age-group.[25]

Amongst the elderly, the issues most likely to influence the risk/benefit balance are:

2.4.1 Myopathy

All statins have been reported to cause myopathy with the severity ranging from asymptomatic increases in creatinine kinase to muscle aches and weakness to fatal rhabdomyolysis.[26] The precise mechanisms are not well-understood. Of note, these myopathies are dose dependent and may occur even after therapy has been tolerated for up to 1 year.[27] The estimated incidence of statin myopathy in trials has ranged from 1.4 to 5% with the lowest rates reported for atorvastatin at low or high dosage.[27,28] Older age, female gender and concomitant use of inhibitors of cytochrome P450 3A4 are recognised risk factors for the development of statin myopathy.[29] There may also be a higher risk of disability in older adults using statins due to age-related sarcopaenia.[30]

2.4.2 Diabetes

In a recently published meta-analysis of statin trials, statin use was associated with a 9% increased risk for incident diabetes with little heterogeneity between trials.[31] Meta-regression indicated that the risk of diabetes was highest in trials of older participants.[31] In light of this data, in 2012 the FDA modified the labelling of statins to include a warning for the potential risk of increased blood glucose levels and new type 2 diabetes.[32]

2.4.3 Cancer

One trial (PROSPER) reported an increased risk of cancer and cancer mortality with statin therapy among men and women older than 70 years.[20] However, this effect was not observed in other statin trials. A meta-analysis of all statin trials suggested no overall increased risk of cancer or cancer mortality over an average follow up of 4.8 years, albeit in a mostly middle-aged population.[33] Nonetheless, more data on the effects of statin therapy on risk of cancer in elderly patients is required and further follow up studies have been called for in such patients.[25] Longer follow up would determine whether new cancer events could occur with time. This is especially important if statin therapy is to be advocated for primary prevention.

2.4.4 Cognitive impairment

The FDA recently reported a review of the US spontaneous reporting database (AERS), the published medical literature (case reports and observational studies), and randomised clinical trials to evaluate the effect of statin therapy on cognition.[32] The post-marketing adverse event reports generally described ill-defined memory loss that was reversible upon discontinuation of statin therapy. Most reports were from individuals over the age of 50 years. Time to onset of the impairment was highly variable, ranging from one day to years after statin exposure. The review did not find an association between cognitive impairment and any specific statin, any specific age group, any particular statin dose or concomitant medication use. Furthermore, the cognitive impairment did not appear to be associated with fixed or progressive dementia, such as Alzheimer's disease. Nonetheless, in 2012 the FDA modified the labelling of statins to include a warning for the potential risk of cognitive effects such as memory loss and confusion.[32] A subsequent meta-analysis of data from 25 randomised trials reported no statistically significant effect of statins on a crude overall measure of cognition across all cognitive domains.[34] This analysis included 12 trials undertaken exclusively in younger populations (< 65 years) where subtle effects may not be measurable and only 4 trials that had more than 1-year follow up. Indeed, this meta-analysis could not address either long-term safety or efficacy particularly in older populations, largely because few such trials exist.

2.4.5 Liver Injury

Statins have been associated with mild elevations in liver enzymes in approximately 0.5-2.0% of people, particularly within the first 12 weeks of therapy being initiated.[35] Meta-analysis of trials has demonstrated a 50% increased risk of elevations in liver transaminase compared to placebo or control.[36] The response appeared dose dependent. However, the elevations were usually transient, rarely associated with symptoms and deemed unlikely to be clinically relevant.

Serious statin induced liver injury was rare. Post marketing reviews of statins conducted by the FDA between 2000 and 2009 indicated fewer than 2 cases of statin associated serious liver injury per one-million patient years reported to the Adverse Event Reporting System.[37] Furthermore, although the use of statins has been rising over this period, no detectable increase in the annual rates of fatal or severe liver injury cases possibly or probably causally associated with statin use has been observed.[37]
Whether the risk of statin induced liver damage is increased in the elderly compared to younger people is currently unknown.

2.5 Utilisation

Statins are one of the most frequently dispensed drugs.[38] In the financial year ending 30 June 2012, over 10 million prescriptions for atorvastatin and 6 million prescriptions for rosuvastatin were dispensed in Australia.[39] In the period from April 2005 to March 2010, 86% of statin initiations came from general practitioners (GPs).[40] In 2011, the British Heart Foundation reported that 1 million statin prescriptions were provided each week in the United Kingdom.[41]

At present statin use in the elderly is lower than expected given the high absolute risk of this population. A UK survey of 41,250 GP medical records, reported that 23% of patients with no history of cardiovascular disease and aged over 75 were prescribed statin therapy compared to 29% in the 70-74 age group.[42] Amongst patients ≥75 years, for every 5 year increment in age the odds of being prescribed a statin decreased compared to the 40-44 year age group. One US study reported that 22% of people aged 80 years or older (in a primary prevention setting) were prescribed a statin[43] and another that those aged ≥70 years were less likely to use a statin than those aged 65-69 years (OR 0.57, 95% CI 0.39-1.09).[44] In an Australian survey of people aged 50 years and older, approximately 40% of respondents aged ≥75 years reported taking a statin.[45]

2.6 Implications of Guidelines on Treatment of Cholesterol in the Elderly

The potential for greater use of statin therapy amongst older people is illustrated by Figure 2 a) and b). This figure provides an approximation of the proportion of each age group where the estimated absolute cardiovascular risk exceeds a 15% likelihood of a new vascular event in the next five years (commonly regarded as the fundamental indication for statin therapy).[46] It is based on the cardiovascular death rate multiplied by four; in the WOSCOPS trial there were four incident cardiovascular events for each fatal event.[47] On this basis, most males over the age of 75 years and a high percentage of females would qualify for consideration of statin therapy.

Greater use of statins is also likely to be driven by the recent changes in guidelines on the treatment of cholesterol to reduce vascular events, which have caused controversy by now recommending moderate-to-high intensity statin therapy for people aged 40-75 years at much lower absolute risk (i.e. 10 year absolute cardiovascular risk of 7·5% or higher compared to 20% or higher in previous guidelines), with no requirement for monitoring of lipid levels.[48] In fact, guidelines that have moved from target-based to absolute cardiovascular risk based treatment strategies, controversially risk medicalisation of ‘healthy older populations’ as all elderly individuals (>70 years) have an estimated absolute cardiovascular risk greater than 10%.

Figure 2: The proportion of each age group in each absolute cardiovascular risk stratum for a) males and b) females. The shaded section under the thick black line shows the approximate percentage of each age group with a risk of 15% of developing a cardiovascular event in the next 5 years. The different shades of blue (from lighter to darker) show the proportions with a 5%, 10%, 15% and 20% risk.

a) Males

Percentile of 10-year CHD mortality risk	Age: 25-29	30-34	35-39	40-44	45-49	50-54	55-59	60-64	65-69	70-74	75-79	80-84	85+	SUM	% of all CHD deaths
2-5	0	0	1	2	4	7	9	13	21	38	54	54	102	305	2
6-10	0	0	1	2	5	9	12	16	25	44	61	64	109	348	3
11-15	0	0	1	3	6	10	14	18	29	50	69	73	116	389	3
16-20	0	1	2	3	7	12	16	21	32	54	76	83	123	430	3
21-25	0	1	2	4	7	13	18	23	36	57	82	91	128	462	3
26-30	0	1	2	4	8	14	19	25	39	59	88	98	133	490	4
31-35	0	1	2	5	9	15	21	27	41	62	93	104	140	520	4
36-40	0	1	3	5	10	16	22	30	44	65	97	109	147	549	4
41-45	0	1	3	6	11	17	24	32	45	68	101	114	154	576	4
46-50	0	1	3	6	11	18	25	34	48	71	105	119	160	601	5
41-55	0	1	3	7	12	19	27	37	51	75	110	124	166	632	5
56-60	1	2	4	7	13	21	29	40	55	79	115	129	171	666	5
61-65	1	2	4	8	14	22	31	43	58	84	120	133	176	696	5
66-70	1	2	4	9	16	24	34	47	63	88	126	139	182	735	6
71-75	1	2	5	10	18	26	37	51	67	94	133	145	189	778	6
76-80	1	3	5	11	20	29	42	56	73	102	141	153	197	833	6
81-85	1	3	6	13	23	33	48	62	79	111	150	162	210	901	7
86-90	2	4	8	16	28	39	57	72	89	124	161	174	236	1010	8
91-95	2	5	11	19	33	45	66	82	99	141	173	187	263	1126	8
96-100	2	6	14	22	39	52	75	92	109	158	185	199	290	1243	9
SUM	12	37	84	162	294	441	626	821	1103	1624	2240	2454	3392	13290	
% of all CHD deaths	0	0	1	1	2	3	5	6	8	12	17	18	26		

b) Females

Percentile of 10-year CHD mortality risk	Age: 25-29	30-34	35-39	40-44	45-49	50-54	55-59	60-64	65-69	70-74	75-79	80-84	85+	SUM	% of all CHD deaths
2-5	0	0	0	0	1	1	2	3	6	13	24	46	123	219	2
6-10	0	0	0	0	1	1	2	4	8	15	28	53	150	262	2
11-15	0	0	0	1	1	1	3	5	10	17	31	61	176	306	3
16-20	0	0	0	1	1	2	3	5	11	20	35	69	202	349	3
21-25	0	0	0	1	1	2	3	6	12	22	38	75	223	383	3
26-30	0	0	0	1	1	2	4	7	13	24	42	82	239	415	4
31-35	0	0	0	1	1	2	4	8	14	26	45	87	254	442	4
36-40	0	0	0	1	2	3	4	8	15	28	47	91	268	467	4
41-45	0	0	0	1	2	3	5	9	16	31	51	95	283	496	4
46-50	0	0	0	1	2	3	5	9	16	33	55	100	301	525	4
41-55	0	0	1	1	2	3	6	9	17	36	60	105	323	563	5
56-60	0	0	1	1	3	4	6	10	18	37	67	112	346	605	5
61-65	0	0	1	2	3	4	7	10	19	39	75	120	367	647	6
66-70	0	0	1	2	3	5	7	11	21	41	84	129	382	686	6
71-75	0	1	1	2	4	5	8	12	23	44	93	140	391	724	6
76-80	0	1	1	2	4	6	9	14	25	49	103	152	397	763	6
81-85	0	1	1	3	5	7	11	17	27	55	115	167	406	815	7
86-90	0	1	2	4	6	10	14	20	33	65	134	194	430	913	8
91-95	1	1	3	5	8	12	19	26	41	79	153	221	455	1024	9
96-100	1	1	3	6	10	14	24	32	51	93	172	249	482	1138	10
SUM	2	6	15	36	61	90	146	225	396	767	1452	2348	6198	11742	
% of all CHD deaths	0	0	0	0	1	1	1	2	3	7	12	20	53		

Zoungas S, *et al. BMJ Open* 2023; 13:e069915. doi: 10.1136/bmjopen-2022-069915

2.7 Summary of rationale

- Meta-analysis of trials highlights the uncertainty about the efficacy of statins in the prevention of morbidity and mortality in older populations (over 70 years). This may stem from inadequate numbers in this age group included in major trials.
- Warnings about adverse effects of statins have recently been added to the product information but their frequency is uncertain and appears higher in the elderly.
- A strong potential exists for greater promotion of statins amongst the over 70 age group on the basis of recent guidelines advocating use for primary prevention at lower absolute cardiovascular risk levels. Evidence in the elderly is currently insufficient to support this proposal.
- Real world trials examining the impact of statins on a range of clinical outcomes important to older people have not been conducted.

Zoungas S, *et al. BMJ Open* 2023; 13:e069915. doi: 10.1136/bmjopen-2022-069915

3. Study Objectives

3.1 Primary objectives

To determine in people aged ≥70 years the effect of statin therapy (40 mg atorvastatin) versus placebo, over an average 6-year treatment period, on two co-primary clinical endpoints:

(i) a composite of all cause death or dementia or development of disability and;
(ii) a composite of cardiovascular death, non-fatal myocardial infarction or non-fatal stroke.

3.2 Secondary objectives

(1) To determine the effects of statin therapy versus placebo in people aged ≥70 years on each of: all cause death, cardiovascular death, dementia, other cognitive impairment, disability, all cause hospitalisation, need for permanent residential care, fatal and non-fatal myocardial infarction, fatal and non-fatal stroke (thromboembolic and haemorrhagic), fatal and non-fatal cancer, atrial fibrillation, heart failure and quality of life.

(2) To assess the cost-effectiveness of statin therapy for primary prevention in people aged ≥70 years.

3.3 Tertiary objectives

These include effects of statin therapy on pathology measures such as fasting glucose/HbA1c, urine ACR and estimated glomerular filtration rate (eGFR) as well as frailty phenotype and functional independence.

Zoungas S, *et al. BMJ Open* 2023; 13:e069915. doi: 10.1136/bmjopen-2022-069915

4. Hypotheses

4.1 Primary null hypotheses

Treatment with statin (atorvastatin 40 mg) compared with placebo will not prolong overall disability free survival amongst a healthy elderly population (≥70 years).

Treatment with statin (atorvastatin 40 mg) compared with placebo will not reduce the incidence of major adverse cardiovascular events amongst a healthy elderly population (≥70 years).

4.2 Primary alternative hypotheses

Treatment with statin (atorvastatin 40 mg) will prolong overall disability free survival amongst a healthy elderly population (≥70 years).

Treatment with statin (atorvastatin 40 mg) will reduce the incidence of major adverse cardiovascular events amongst a healthy elderly population (≥70 years).

Zoungas S, *et al. BMJ Open* 2023; 13:e069915. doi: 10.1136/bmjopen-2022-069915

5. Study Design

5.1 Trial Design

STAREE is a double blind, randomised placebo-controlled parallel group trial that is investigating whether statin therapy can prolong good health and maintain independence amongst older people.

5.2 Study Population

5.2.1 General considerations

The study will enrol men and women 70 years of age and over who are free from cardiovascular disease, diabetes and dementia and living independently in the community.

The study sites are listed in Appendix 1.

5.2.2 Inclusion criteria

Potential participants must meet the following criteria to be included in the study:

- Men and women aged ≥70 years living independently in the community,
- Willing and able to provide informed consent and accept the study requirements (Note: competent physical ability to participate in the trial is assessed using the Life Ability ADL questionnaire)

5.2.3 Exclusion criteria

Potential participants who meet any of the following criteria will be excluded from the study:

- A history of clinical cardiovascular disease (defined as myocardial infarction, angina, coronary artery angioplasty and/or stenting, coronary artery bypass grafting surgery, heart failure, stroke, transient ischaemic attack (TIA), carotid stenosis, abdominal aortic aneurysm and peripheral vascular disease),
- Clinical diagnosis of dementia, treatment with medications for dementia (e.g. donepezil (Aricept), rivastigmine (Exelon) or Galantamine (Galantyl) or a 3MS score <78 on screening.
- A history of diabetes or evidence of diabetes on pathology results (WHO diagnostic criteria whereby HbA1c ≥ 6.5%/ 48 mmol/mol or fasting glucose ≥7.0 mmol/L on two consecutive tests),
- Total cholesterol >7.5 mmol/L, [49]
- Moderate or severe chronic kidney disease (persistent proteinuria whereby urine albumin: creatinine ratio >30 mg/mmol [50] and/or eGFR <45 ml/min/1.73m^2 on two consecutive tests), [49]
- Moderate or severe liver disease (persistent elevations of transaminases of more than 3 times the upper limit of the normal laboratory reference range),
- Serious inter-current illness likely to cause death within the next 5 years such as terminal cancer or obstructive airways disease,
- Current participation in another interventional clinical trial,
- Absolute contraindication to statin therapy,
- Current use of statin therapy or other lipid lowering therapy for primary prevention and unwilling to stop therapy,

- Current long term or permanent use of potent cytochrome P450 (CYP) 3A4 inhibitors as specified in Appendix 3.

5.2.4 Rescreening or reassessment

Potential participants who are excluded on the basis of either their 3MS total score (<78) or their responses on the Life Ability questionnaire (unable to perform items 9-14) may be re-screened if the result is likely to be related to a temporary issue that may resolve over time (e.g. temporary depressive symptoms as indicated by the CES-D 10 or temporary disability related to fracture).

To be eligible for re-screening, participants with a 3MS score below 78 that also score 8 or above on the CES-D 10 (indicating presence of depressive symptoms) may be re-screened in 3 months. There may be instances where rescreening or reassessment of certain tasks may be required, such as participants with a temporary disability, and these participants will be considered for such on a case-by-case basis.

5.3 Study Intervention and Choice of Comparator

5.3.1 Intervention

Two tablets of Atorvastatin (2 x 20 mg/day) or identical placebo.

5.3.2 Rationale for choice of atorvastatin

Atorvastatin is the most commonly prescribed statin in Australia.

Atorvastatin is well researched with wide practice experience. Importantly, relative to other statins, it has a favourable side effect profile and has been shown to be one of the most potent statins for reducing low density lipoprotein concentrations (LDL-C) and triglyceride levels. The dose of 40 mg is expected to produce a 30-50% reduction in LDL-C (see table below).

Zoungas S, *et al. BMJ Open* 2023; 13:e069915. doi: 10.1136/bmjopen-2022-069915

Table 1: Average relative reduction in LDL cholesterol concentrations with different doses of commonly used statins. (51)

	Daily dose of different statins				
	5 mg	10 mg	20 mg	40 mg	80 mg
Pravastatin	15%	20%	24%	29%	33%
Simvastatin	23%	27%	32%	37%	42%
Atorvastatin	31%	37%	43%	49%	55%
Rosuvastatin	38%	43%	48%	53%	58%

Shaded boxes indicate regimens that can produce about a halving or more in LDL cholesterol concentrations (largely irrespective of patient characteristics, including presenting concentrations of cholesterol). The 2016 cost for generic atorvastatin 40 mg daily in the UK is about £2 per 28 days of treatment;[184] rosuvastatin 20 mg daily currently costs about £25 per month,[185] but it became available as a generic in the USA during 2016.

Different statins have different potencies and newer therapies such as atorvastatin and rosuvastatin produce larger reductions in LDL-C per mg of drug than older therapies, such as simvastatin and pravastatin.[51] Adverse events associated with statin treatment may be more likely with higher doses of specific statins (i.e. simvastatin ≥ 80 mg) and with combination therapy (i.e. statin and fibrate). [52, 53] However, the flexibility of reducing from 40 mg (standard dose) to 20mg (low dose) will assist with exploring dose-response medication-related side effects and up or down titration will assist with reducing study medication discontinuation rates.

A very important issue that has not been addressed is the balance between expected benefits and harms of statin therapy in people aged over 70 years. STAREE will provide evidence to address this.

5.4 Participant recruitment

5.4.1 Recruitment

First participant expected to be consented to the study in October 2015 and the first participant expected to be randomised to study medication in November 2015.

5.4.2 Setting

STAREE will be conducted in Australian primary care (GP) practices and the community.

Registered GP co-investigators will access their computerised practice databases, in association with STAREE GP Liaison Officers if requested, to identify all patients aged 70 years and over. Depending on the practice database, patients will be further screened for inclusion and exclusion criteria. Those who are physically able to attend the practice and considered suitable for the study by their GP, will be invited to participate in the study via a letter of invitation.

5.4.3 Community participants

Any participants whose usual GPs are not registered as STAREE co-investigators will be considered as community participants. They will be assessed by a STAREE medical officer for suitability to be in the study. Their usual GP will be notified of their involvement and will be provided a copy of any pathology and any abnormal assessments that are performed as part of the study. Baseline and annual visits for community participants will be conducted at community venues or in the STAREE site offices.

5.4.4 STAREE hub locations

The STAREE central coordinating site is located in Melbourne, Australia.

STAREE hubs will be located in regional Victoria, Queensland, Tasmania, Western Australia, South Australia and New South Wales (see Appendix 1). Hubs will service the immediate and surrounding areas and may assist with other locations when requested.

5.5 Participant timeline and schedule of assessments

Figure 3 shows the participant timeline including the baseline screening phase, randomisation and follow up phase.

Table 2 shows a summary of the schedule of assessments to be undertaken at each study visit. Additional details of the cognitive assessments, physical measures and laboratory tests are provided in Appendix 6.

5.6 Baseline Screening Phase

The baseline screening phase will comprise a screening phone call, baseline visit 1, medication run in, GP visit and baseline visit 2.

Eligibility for randomisation into the study will be assessed across the baseline screening and medication run-in phase and will be based on the inclusion and exclusion criteria as well as compliance with the run-in medication.

5.6.1 Screening call

Potential participants will phone the study centre after they have received an invitation to participate in the study. At this phone screening call, they will be screened for eligibility to be included in the study. Those who pass this first screening stage will then be invited to attend baseline visit 1.

Figure 3: Participant Timeline

Call

Baseline Visit 1 (Face to face or phone visit)

- Informed consent obtained
- Questionnaires, exclusion criteria and pathology requested

4 week run-in

- Participant to take a 4-week run-in of 1 tablet of placebo
- Participant to see GP for inclusion in study

Baseline Visit 2 (Face to face or phone visit)

- Questionnaires are administered
- Review inclusion/exclusion criteria

Randomisation

- Participants randomised to placebo or active statin
- Participants to receive their study medication via tracked post (annual send outs)

4 week post randomisation

- Participants to take 1 tablet / 20 mg of study medication

Call 4 weeks after randomisation

- Participants to increase dosage to 2 tablets / 40 mg of study medication

Participant contact post-randomisation

- Participants will have a study follow up visit every 6 months (these study visits will alternate as phone visits or clinic/phone visit).
- Participants will have a final close-out study visit (clinic/phone visit)

Zoungas S, *et al. BMJ Open* 2023; 13:e069915. doi: 10.1136/bmjopen-2022-069915

Table 2: STAREE measurement and study activity schedule. A single X (with no superscripts) indicates that all specified measures carried out. The presence of a superscript indicates that the corresponding specific assessment is performed at that time point.

	Screening	Baseline visits (BV)			Post-randomisation			
Measurement/ Activity	Screening call	BV 1	GP visit	BV 2	1 month call	Annual visits (without cognitive testing)	Annual visits (with cognitive testing)	Close out visit
Type of visit	phone	clinic or phone	clinic or telehealth	clinic or phone	phone	clinic or phone	clinic or phone	clinic or phone
Month in trial (week)	0	0	0	0	(4)			
Review inclusion/ Exclusion criteria	X	X	X	X				
Obtain informed consent		X						
Dispense study medication (supply posted after annual study visit once compliance checked)		X (run-in med only)		Posted (after randomis ation)				
Medication titration					X			
Assess medication compliance				X		X	X	X
Concomitant medications		X				X	X	X
Blood pressure & heart rate/rhythm[a], height[b], weight[c], waist:hip ratio[c],		X[a,b,c]				X [a, c,]	X [a, c,]	X
Lifestyle profile & personal medical history[d], family medical history[e] & IPAQ-E[f], physical function		X[d, e]		X		X[d, f]	X[d]	X[d]
Routine Laboratory testing, GP referral to local pathology Bloods**: fasting glucose[1], fasting lipids (TC, HDL, LDL, triglyceride)[1], Hb[2], CK[2], creatinine[3], (eGFR)[3], ALT[3], AST[3], HbA1c[3], Urine: ACR[3]		X[1,2,3]				X[3]	X[3]	X[1,3]
Measurement/ Activity	Screening call	BV 1	GP visit	BV 2	Post-randomis ation			

Zoungas S, *et al. BMJ Open* 2023; 13:e069915. doi: 10.1136/bmjopen-2022-069915

	Screening	Baseline visits (BV)			Post-randomisation			
	Screening call	BV 1	GP visit	BV 2	1 month call	Annual visits (without cognitive testing)	Annual visits (with cognitive testing)	Close out visit
Type of visit	phone	clinic or phone	clinic	clinic or phone	phone	clinic or phone	clinic or phone	
Month in trial (week)	0	0	0	0	(4)			
**Quality of life**<br>SF-36				X		X	X	X
**Assess cognitive function**<br>3MS± (and CES-D 10 to exclude depression); CAM[1]		X					X	X
**Detailed cognitive tests**[β£]<br>COWAT, Stroop, HVLT-R, SDMT, CERAD Constructional Praxis, Lurian Overlapping Figures, Trail Making Test				X			X	X
**Assess physical disability**<br>Life disability ADL		X				X	X	X
**Biobank samples collected**				X				
**Clinical event reporting**<br>Questionnaire & medical report review						X	X	X

**All study visits, including baseline and follow up visits, may be either conducted in person or by phone call. For visits conducted by phone call, physical measures (blood pressure, heart rate and weight) will be collected from medical records if available.

± Full 3MS is conducted at clinic visits and a partial 3MS is conducted at phone visits

[β] Annual visits with cognitive testing conducted by phone will not include SDMT, STROOP, CERAD Constructional Praxis subscale and Lurian Overlapping Figures. These tests will only be conducted at in person visits.

[£]B-ADL (Bayer Activities of Daily Living) is administered to the named ‘study partner’ of any participant who shows a pre-specified decline in cognitive tests at an annual visit. If the participant does not consent to a study partner undertaking the B-ADL, then it may be administered to the participant themselves.

5.6.2 Lifestyle profile and screening visits (Baseline visits)

STAREE staff will explain the study to participants. After informed consent is provided measurement and medical history data will be collected to further assess eligibility for the study. Participants will be given a copy of their consent form. Participants will be assigned a unique identification number, which will be used throughout the study.

The information to be collected and reviewed at the baseline visits will include the following:

a. Basic demographic and lifestyle factors
b. Past medical history – identified by the participant and GP practice medical record or patient health summary (as available)
c. Cognitive assessment – 3MS[54] (full or modified version if phone visit)
d. Depressive symptoms assessment – CES-D 10(55)
e. Physical disability – ADL using Life Ability questionnaire[56]
f. Blood pressure, heart rate and rhythm, weight, height and waist circumference
g. Concomitant medications
h. Pathology – referred to local pathology collection service for blood and urine tests

Note: Pathology testing results from participants' medical records may be used to assess eligibility if performed no more than 6 months before Baseline visit 1. HbA1c may be used if performed within the past 12 months.

Medication for the run-in phase will be handed out.

5.6.3 Post screening GP/medical visit (Run-in phase between Baseline visits 1 and 2)

Participants will see their GP who will be an enrolled co-investigator (or the STAREE medical officer if the usual GP is not an enrolled co-investigator) and will complete an Inclusion/Exclusion Criteria Worksheet. The baseline pathology testing results will be reviewed by the GP (or the STAREE medical officer). These results will help determine eligibility via the inclusion and exclusion criteria.

5.6.4 Post screening visit study activity (Run-in phase between Baseline visits 1 and 2)

Participants will enter a run-in phase of 4 weeks whereby they will be asked to take one tablet of study medication (placebo) a day.

5.6.5 Determinations of Eligibility and Assessments visit (Baseline Visit 2)

Participants will have a second visit with STAREE field officers after the run-in phase. This visit will be conducted within 4 to 6 weeks of the baseline visit 1.

Participants will be deemed eligible to participate if they are compliant with run-in medication pill count (>85% taken), have satisfactory pathology testing and their GP (or the STAREE medical officer) has provided approval using the GP screening form.

If deemed eligible to continue to baseline visit 2, participants will undergo the following assessments at this second screening visit:

a. Quality of life – Short Form (SF)--36 Questionnaire.(57)

Zoungas S, *et al. BMJ Open* 2023; 13:e069915. doi: 10.1136/bmjopen-2022-069915

b. Comprehensive assessment of cognition – COWAT[58], STROOP[59], HVLT-R[60], SDMT[61], CERAD Constructional Praxis subscale[62], Lurian Overlapping Figures[63], Trail Making Test[64] (if funding available).

Participants' willingness to donate and have blood samples collected and stored in the STAREE biobank will be ascertained at the baseline visit 2 and offered at STAREE hubs with facilities for sample processing and storage.

The purpose of this sample collection is to allow identification and analysis of potential biomarkers that predict the onset of age-related diseases such as cardiovascular disease, dementia, cancer and diabetes.

5.7 Randomisation and follow up phase

The randomisation and follow up phases are illustrated in Figure 3.

5.7.1 Randomisation procedure

Following the completion of data collection at baseline visit 2, the Randomisation Form will be completed, and participants will be randomised into the STAREE study via the study website.

An independent statistician will generate the allocation list, using the statistical package R. This arrangement will ensure that the randomisation code remains inaccessible to all study staff and investigators. The allocation list will be generated using Cox model randomly permuted blocks (of size either 2 or 4), with randomisation stratified for site and age group (<80y, 80+y). Blocks will ensure ongoing approximate balance of randomisation to statin and placebo within strata. Treatment allocation will be achieved through a web or interactive voice recognition telephone-based system.

Following the completion of the randomisation process the study medication will be posted to participants.

Participants' contact details will be confirmed to ensure the correct mailing address for study medication is recorded and the addresses of the closest relative, friend and local doctor provided.
Participants will be instructed to take one tablet of study medication a day.
Participants will be instructed to call the study centre if they have any adverse effects.

The distribution of study medication is detailed in Section 8.

A record of all participants undergoing screening and randomisation will be made in the appropriate tracking log.

5.7.2 Post randomisation procedure

Participants will be contacted by phone one month after commencement of study medication to review tolerability and compliance with study medication and instruct them to increase the dose from one tablet to two tablets. The date of this phone contact will be taken from the drug receipt log as recorded in the STAREE database.

Zoungas S, *et al. BMJ Open* 2023; 13:e069915. doi: 10.1136/bmjopen-2022-069915

Participants reporting any tolerability issues with the study medication will be referred back to their GP for further advice and management.

Table 3. Study medication

	0	4 weeks
Number of tablets	1	2
	Receive study medication	Phone call to increase to 2 tablets

5.7.3 Blinding

All STAREE staff including the investigator team, management, administration, medical officers, GP Liaison Officers, Field Officers, Data Officers and students will be blinded to treatment allocation through the randomisation procedure and study duration.
All GP co-investigators and practice staff will be blinded to treatment allocation.
All participants will be blinded to treatment allocation.

In the event of a clinical emergency, participants will be assumed to be on active treatment and the recommendation will be to cease treatment.

A process for emergency unblinding will be in place at Ramsay Healthcare. Selected Ramsay Healthcare staff will be granted access to the randomisation code. This information will be accessible and shared using a Secure File Transfer Portal (SFTP).

The need for unblinding of study participants will be determined on a case-by-case basis after referral to a senior STAREE medical officer.

5.7.4 Annual Follow up visit

Each year after randomisation, participants will participate in a study visit with a Field Officer. Visits may be conducted face-to-face or by phone. Annual measurements will include cognitive tests and disability assessments, lifestyle questionnaires as well as physical measures. The Quality of Life (SF-36), Life Ability and Annual medical history questionnaires will be provided in an online format or posted to participants who have agreed to this. For all other participants these surveys will be conducted at face-to-face visits. Pathology will be repeated (excluding lipids). Participants will be asked about their recent medical history and any concomitant medications they are taking. Additionally, participants will be asked about serious adverse events (SAEs) and potential study endpoints.

Following notification, clinical information relating to any SAE or endpoint will be sourced through: a) medical record review and b) hospital records and discharge summaries.

5.7.5 Phone Contact

After randomisation and the mail out of study medication, if receipt of study medication has not been registered a participant will be contacted by phone.

Zoungas S, *et al. BMJ Open* 2023; 13:e069915. doi: 10.1136/bmjopen-2022-069915

Annual phone calls (6, 18, 30, 42, 54, 66 months) will be made to ask participants about SAEs and potential study endpoints and to encourage compliance with study medication.

Data to be collected during the phone calls include:

- Compliance with study medication
- Serious adverse event and endpoint notification
- Concomitant medications
- Physical disability (Life disability ADL)

5.7.6 Other Actions

If a participant does not proceed to randomisation, then the reason for non-randomisation is to be documented and collected on all relevant hard copy and electronic (database) Case Report Forms (CRF) such as the Screening CRF and Assessment and Randomisation CRF Contact with the participant regarding the outcome of the screening phase will be recorded within the Participant Communication Log

5.8 Economic Analysis

In addition to the disability free survival and cardiovascular outcomes, STAREE will examine the cost effectiveness of statin treatment for prolonging disability free survival and preventing major adverse cardiovascular events in healthy individuals aged ≥70 years.

Decision analysis will be used to compare the downstream consequences of atorvastatin versus placebo. Incorporation of Markov and life-tabling techniques will allow for the modelling of outcomes beyond the 6-year duration of the trial.

The main output of interest in health economic modelling will be the incremental cost-effectiveness ratio in terms of net costs per unit of health gain. Net costs will comprise the costs of statin minus costs saved from the reduction in downstream health services utilisation. Health gains will be measured by years of life and quality-adjusted life years (QALYs) gained. Both will be enabled by the collection of time-to-outcome data and QALYS also by collection of quality of life data. Cost data will be estimated by applying published unit costs (from an Australian healthcare system perspective) to data on health services utilisation. All health economic analyses will be undertaken in accordance with recommended approaches, such as 5% discounting of estimated future costs and health gains. To account for any uncertainty in the data inputs for health economic modelling, sensitivity and uncertainty analyses will be undertaken via Monte Carlo simulation.

6. Study Endpoints

6.1 Primary endpoints

STAREE will have two co-primary endpoints, disability free-survival and major adverse cardiovascular events, which are both composite endpoints.

1. Disability-free survival - defined as survival free of dementia or persistent physical disability (as derived from the endpoints of all-cause mortality, dementia and physical disability), and

2. Major adverse cardiovascular events – defined as the first occurrence of a non-fatal myocardial infarction, non-fatal stroke or cardiovascular death.

6.2 Secondary endpoints

Two types of outcome measures are pre-specified secondary endpoints in STAREE. The first group are components of the co-primary endpoints and additional cognitive and cardiovascular endpoints (heart failure, other acute coronary syndromes and revascularisations). The second group include non-cardiovascular endpoints (need for permanent residential care, hospitalisation and cancer).

1. All cause death
2. Cardiovascular death
3. Fatal and non-fatal myocardial infarction
4. Fatal and non-fatal stroke
   a. Haemorrhagic stroke
   b. Thromboembolic stroke
5. Persistent physical disability (in ADL based on Life Ability questionnaire[56])
6. Dementia
7. Other cognitive impairment (not meeting criteria for Dementia)
8. Approved need for permanent residential care (based on reporting by an Aged Care Assessment Team)
9. All cause hospitalisation (reasons and length of stay)
10. Heart failure
11. Atrial fibrillation
12. Revascularisation procedure
13. Fatal and non-fatal cancer (excluding non-melanoma skin cancer)
14. Quality of life (SF-36 questionnaire[57])
15. Cost-effectiveness

6.3 Tertiary endpoints

Pre-specified tertiary endpoints are:

1. Fasting glucose/HbA1c
2. Urine ACR
3. Estimated glomerular filtration rate (eGFR)
4. Frailty
5. Loss of functional independence (instrumental ADLs)

Please refer to Appendix 2 for brief definitions of primary and secondary endpoints.

6.3 Endpoint ascertainment

At 6-month telephone calls and annual visits, participants will be asked whether they have been diagnosed with any new health events which are from a list of study endpoints. Notification of a potential study endpoint will trigger the collection of further information for endpoint confirmation and adjudication. Confirmation of endpoints will be ascertained by collecting information from any of the following sources within 90 days of learning of the event:

- Details from medical records from the usual treating physician or practice held medical record.
- Medical records obtained by letter/fax/email contact with other treating specialist physicians or secondary/tertiary medical care centres.
- Case Report Forms.
- Hospital records/discharge summaries, pathology reports.
- Death Certification: The National Death Index will be routinely sourced for notification of death not identified through the above processes for up to 5 years after the completion of study.
- Review of Commonwealth Department of Health and Ageing (DOHA) database of aged care assessments (ACAP dataset).
- Review of cancer registries for up to 5 years after the completion of study.
- Aged Care Assessment Service (ACAS report): A copy is provided to the GP and assesses the needs and level of care recommended for the individual.

6.4 Endpoint adjudication process

An end-point adjudication committee (EAC) will be appointed with the task of assessing the clinical data relating to all endpoints. Data collected from GPs, hospitals, specialists and other sources by the research staff will be presented to the EAC. The information available to the EAC will not only include the clinical source documentation obtained from the GP records but also death certificates and, if available, autopsy reports blinded to participant identity. The EAC will adjudicate through an online system with meetings conducted to resolve discordant adjudications and to develop decision rules.

7. Sub-studies and ancillary studies

7.1 STAREE-HEART

STAREE-HEART will conduct a suite of additional cardiac assessments in a subset of up to 500 STAREE participants before they are randomised to study medication. It aims to determine the effect of statin treatment over a 3 year period compared with placebo on markers of cardiac ageing. These include an echocardiogram which measures global longitudinal strain (GLS), which may predict incident atrial fibrillation (AF) and heart failure, and twice daily handheld ECG recordings over a 2-week period which may improve AF detection 4-fold compared to usual screening. Additionally, a new energy waveform 12-lead ECG will be performed. The assessments will take place prior to randomisation and will be repeated at the year 3 follow up.

7.2 STAREE-MIND

STAREE-MIND will conduct neuroimaging assessments in a subset of up to 300 STAREE participants. STAREE-MIND aims to determine the effect of statin treatment on longitudinal measures of brain health including grey matter volume, white matter integrity and white matter hyperintensities, cerebral perfusion and metabolism, and iron loading. It will also examine the relationship between imaging changes in the brain and cognitive impairment and dementia. The imaging will be conducted prior to commencement of the study medication and will be repeated at 4 years of follow up. The detailed STAREE MIND protocol is shown in appendix 10.

Zoungas S, *et al. BMJ Open* 2023; 13:e069915. doi: 10.1136/bmjopen-2022-069915

7.3 STAREE Healthy Ageing Biobank

Up to 2000 STAREE participants located near STAREE study sites with facilities for processing and storage of biospecimens (Melbourne, Perth and Hobart) will be invited to provide a blood and urine sample for the STAREE Healthy Ageing Biobank. This study aims to create a collection of biospecimens which can be used in future research to identify new biomarkers that are linked with development of clinical outcomes such as stroke, myocardial infarction, cancer and dementia.

Zoungas S, *et al. BMJ Open* 2023; 13:e069915. doi: 10.1136/bmjopen-2022-069915

8. Contingency plans for participant wellbeing

Participants will be advised to seek care from their usual treating GP (or other health care providers) for any medical condition arising during the course of the study. Any test measurement result that is outside the normal range will trigger a notification to the participant's GP.

Treating GPs will be co-investigators and provided with information about the study to aid the participant's involvement in the study (wherever possible).

8.1 Commencement of regular lipid lowering agents

A prescription for a lipid lowering agent will be reason to stop the study medication. The participant will cease study medication but continue in the study for observation (routine follow up visits).

8.2 Development of an indication for statins

Any participant who develops a recognised clinical indication (such as a myocardial infarction or stroke) for statin treatment may commence routine statin therapy. The participant will cease study medication but continue in the study for observation (routine follow up visits).

8.3 Post-trial care

At the end of the interventional phase of the trial, it is anticipated that participants will be followed passively for an additional 5 years, contingent on funding being obtained. During this period, study participants will continue to be treated by their usual GPs and follow up will be conducted annually by face to face or phone contact. Study participants will be asked to provide informed consent for the extended post-trial follow up.

Zoungas S, *et al. BMJ Open* 2023; 13:e069915. doi: 10.1136/bmjopen-2022-069915

9. Levels of participant involvement

9.1 Levels of participant involvement

The aim of the study is to assess outcomes on an intention to treat basis, i.e. to test the decision to prescribe a statin and the consequences of that decision. Therefore, every effort will be made to minimise loss to follow up with respect to determining mortality, dementia, disability and major adverse cardiovascular events. Loss to follow up will be minimised by recording three contacts for each participant including their GP, close relative living apart, and close friend.

Full participation in STAREE means that a participant is currently taking study medication and attending for study visits and/or phone calls. If participants request to change their level of involvement or withdraw from the study it will be explained to them that there are number of options. These may include the following:

Changes to study medication
Participants may decide to interrupt or discontinue the study medication for a number or reasons but regardless of this decision, they can continue to be active participants in STAREE:

On Medication: Participants are taking study medication (one or two tablets daily).

Medication interrupted: Participants have temporarily stopped taking study medication due to circumstances such as illness, injury or holidays or for other personal reasons. Participants who have interrupted their medication intend to re-start medication at a future date when the circumstances that have led to the interruption are resolved.

Medication discontinued: Participants have stopped taking study medication permanently. This may be due to adverse effects or for other personal reasons, or because they have developed a clinical indication and been prescribed an open label statin. In all cases, when a participant discontinues study medication, the reason for discontinuation and date of the last dose of study medication will be recorded

Participants who have stopped taking study medication and are deemed by a STAREE medical officer as suitable for medication re-challenge will also be considered as “interrupted” until they either re-start or are deemed to have permanently discontinued medication.

Changes to study visits (clinic and/or phone calls)
Regardless of whether participants are taking study medication or not, they will still participate in study visits (clinic and/or phone calls). If participants do not want to participate in study visits (clinic and/or phone calls), they may elect to participate by review of their medical records corresponding with the trial’s duration (such as GP and hospital records) and national morbidity and mortality databases). All participants will be asked to provide approval for access to this information as part of the study enrolment procedures.

Zoungas S, *et al. BMJ Open* 2023; 13:e069915. doi: 10.1136/bmjopen-2022-069915

9.2 Withdrawal

Participants will be considered to have withdrawn from the study if they revoke their consent. This means that they are not taking study medication, are not participating in study visits (clinic and/or phone calls), and that medical record review is not being conducted. All data that participants have contributed to the study up to the date of withdrawal will remain in the study database, and review of medical records dated earlier than the withdrawal date may be completed. Publicly available data such as death (e.g. from death notices) may also be used in such cases.

Zoungas S, *et al. BMJ Open* 2023; 13:e069915. doi: 10.1136/bmjopen-2022-069915

10. Study Medication and Supplies

10.1 Study treatments

Run-in placebo
The 4 week placebo medication will be provided in either a labelled box or bottle (one box or bottle of 40 tablets). It will be given to participants at the completion of baseline visit 1.

Double-blind Study Medication
Study medication will be provided by Ramsay Healthcare in boxed blister packs (3 sheets of 10 tablets), with the STAREE logo printed on the box. Participants will be randomly allocated to one of two treatments; a) atorvastatin (2 x 20 mg tablets) or b) placebo (2 x 20 mg tablets with identical appearance). Study medication will be posted regularly throughout follow-up, at least every 3-9 months (usually at 6-month intervals).

Post randomisation
After randomisation, study medication will be posted directly to the participant's home or provided to the participant at an annual visit. Participants will be directed to start taking one tablet of study medication from a labelled first box containing 30 tablets. Subsequently participants will be directed to take two tablets from boxes containing 30 tablets.

10.2 Administration of study medication

Run-in period
Participants will be directed to take 1 tablet of run-in medication at the same time every day.

Double blind period
Participants will be directed to take 1 tablet (20 mg) at the same time every day for the first 30 days of the trial. After 30 days, participants will be directed to take 2 tablets (40 mg) at the same time every day.

If a suspected intolerance of the full dose (2 tablets) of study medication (atorvastatin or placebo) develops, the participants' GP may reduce the dose to 1 tablet at their own discretion.

Administration of study medication for those taking allowed Cytochrome P450 3A4 inhibitor at study entry and during follow up
Participants identified as taking certain (allowed) cytochrome P450 3A4 inhibitors will be directed to take 1 tablet (20 mg) of study medication per day for the duration of the study. The list of allowed cytochrome P450 3A4 can be found in Appendix 3.

10.3 Dispensing and randomisation

The number of authorised dispensing staff will be limited to assure proper adherence with established accountability and dispensing procedures. All procedures are described in the STAREE Drug Accountability Manual.

Medication for the run-in phase will be dispensed to the participant by the study staff at baseline visit 1. Each participant will be dispensed 40 placebo tablets and be directed to take one tablet at the same time daily. When the participant returns for the final assessments and

Zoungas S, et al. BMJ Open 2023; 13:e069915. doi: 10.1136/bmjopen-2022-069915

eligibility visit (baseline visit 2), medication compliance will be checked prior to randomisation.

Following the completion of the randomisation process, study medication will be posted to the participant at regular intervals, as required, to ensure continuity of study medication supply for each participant. The participant will be asked to confirm receipt of the study medication by calling the STAREE coordinating centre. If confirmation of receipt has not been made within 10 days of dispatch of the study medication research, staff will contact the participant to ensure receipt. Attempts to contact the participant will continue until receipt has been confirmed. In the rare instance of missing or undelivered study medication, the study medication will be re-issued and sent to the home of the participant. Note, the postage of study medication to participants will be tracked with the delivery or courier service with the signature of the recipient being required upon delivery.

The Medication ID number assigned to the participant will remain the same for the duration of the study with each separate batch being distinguished by a batch number appended to the medication ID number. The participant will receive study medication with the same Medication ID number at each visit.

A '*Study Drug Log'* will record the medication dispensed and the medication returned from the participant.

The *Study Drug Log* will record information on the dispensing of study drug and will include the following:

- Participant ID number and participant acrostic
- Medication ID number
- Date dispensed (initials of dispenser)
- Date of medication return (initialled by research staff)
- Number of tablets returned
- Comments section to allow for explanation of discrepancy, if applicable.

These processes are further detailed in the drug accountability manual.

10.4 Study medication compliance

Compliance with study medication will be checked at six-monthly telephone calls and annual visits. Questions regarding study medication use will also be asked at annual visits to encourage medication compliance (see Appendix 4). The questions are based on the Brief Medication Questionnaire [65] and Morisky questionnaire[66]. Participants will be asked to count and report any unused tablets at annual visits.

10.5 Study medication storage

Study medication storage will be the responsibility of Ramsay Healthcare. Study medication will be predominantly stored at a secure storage centre in a temperature-controlled environment. Placebo run-in medication is released to STAREE periodically, in-bulk, and stored at the coordinating centre. All placebo medication for the run-in phase will be stored in a secured area with restricted access, at room temperature under the supervision of the coordinating centre.

Zoungas S, *et al. BMJ Open* 2023; 13:e069915. doi: 10.1136/bmjopen-2022-069915

The receipt, storage, dispensing, accountability and study medication collection, for both the run-in and randomisation phases, will be the responsibility of both Ramsay Healthcare and the STAREE coordinating centre. Each party will be responsible for maintaining appropriate delivery, storage, and transfer records.

10.6 Study medication accountability and dispensing logs

All study medication dispensed in the trial will be accounted for by confirming the delivery, receipt and commencement dates of each study medication supply. Study medication compliance will be assessed and confirmed by collection, counting and documentation of unused study medication from participants and/or by confirmation of the completion and/or cessation dates of each individual supply with participants by phone.

All unused study medication will be collected to allow for proper destruction of the medication. Unused tablets and packaging will be returned by participants to trial staff at face to face study visits. If unused study medication is not able to be collected at study visits, attempts will be made to confirm safe destruction (see section 8.7 below). Any lost medication packs will be documented, re-dispensed and posted to the participant, with all the details recorded to allow an audit trail. Tablet counts will be made of all returned medication packs. The coordinating centre will make all reasonable attempts to verify that any unused or partially used medication supplies are returned by the study participant and that no remaining supplies are in the participant’s possession.

The coordinating centre will maintain records which adequately document that study participants were provided study medication supplies and doses as specified by the protocol and document and reconcile all study medication received from the participants (see section 8.3).

10.7 Study medication disposal

Unused study medication will be collected at annual visits by study staff whenever possible and disposed. Unused study medication will then be relinquished for safe destruction at a local pharmacy or destruction service. Each batch of destroyed study medication will be documented, along with the date, location and the pharmacy staff name, in the STAREE Study Medication Tracking Log (more detailed information is provided in the STAREE Drug Accountability Manual).

If unused study medication is not able to be collected at an annual study visit, participants will be given the option of disposing their supply at the local pharmacy (i.e. through the Return of Unwanted Medication or RUM scheme) or return via a reply-paid envelope satchel. Destruction of unused study medication by participants at pharmacies or other safe medical waste service will be documented on the STAREE Disposal Form, which will be returned to the STAREE trial for record keeping.

10.8 Overdose of study medication

There is no specific treatment for atorvastatin overdose. Should an overdose occur, the participant should seek emergency medical care and be treated symptomatically with

supportive measures instituted as required. The Poisons Information Centre on 13 11 26 (Australia) may also be contacted for advice on the management of an overdose.

11. Concomitant Therapy

11.1 Prescription medications

All prescription medicine will be recorded on a Concomitant Medications Form including indication for use and start/stop dates. Concomitant medications will be checked at annual study visits where participants will be asked to bring in any medications/vitamins/ supplements they are taking and by review of GP medical records.

Zoungas S, *et al. BMJ Open* 2023; 13:e069915. doi: 10.1136/bmjopen-2022-069915

12. Sample Size and Power calculations

Table 4. Sample size calculations

	ITT effect size (RR)	Power	Required number of events	Required number of participants	On treatment (true) effect
For disability free survival (Death or Dementia or Disability)	**14.5%**	**83.1%**	**1,397**	**9,631**	**18%**
For major adverse cardiovascular events (CV death, non-fatal MI or non-fatal stroke)	**20.2%**	**80.0%**	**619**	**9,631**	**25%**

Based on these sample size calculations 9,631 participants will be recruited and a total of 58,135 person-years accrued.

These calculations assume:

Loss to follow up: 2% per annum (which is expected to be conservative, i.e. the true loss to follow up is expected to be very small assuming good retention of participants and the ability to access medical records or perform record linkage for those who cease regular follow up). This translates to expecting an average of 6.04 years of follow up time per participant.

A two-sided significance level of 5% (alpha rate).

An anticipated age structure of STAREE participants of 56%: 27%: 12%: 5% in the respective age bands 70-74 years: 75-79 years: 80-84 years: 85+ years. These proportions are based on recruitment of the initial 7000 participants to the study and expectations about future recruitment.

Treatment cross-over: In year 1 of follow up we assume 8% of participants who have been randomised to statin will cease taking their study medication. Thereafter, we assume 3% of participants per annum in the statin group will cease taking study medication. In year 1 of follow up we assume 1% of participants in the placebo group will commence statin. Thereafter we assume 3% of participants per annum in the placebo group will commence statin therapy. Further we assume that there are no subsequent treatment "re-cross-overs", i.e. assume a worst-case scenario for the hazard ratio that will be observed in the intention to treat analysis.

The following event rates:

Death, dementia or persistent disability (disability-free survival) - co-primary endpoint A

All cause death

Zoungas S, *et al. BMJ Open* 2023; 13:e069915. doi: 10.1136/bmjopen-2022-069915

We anticipate death rates in STAREE randomised participants will be less than half those in the general population. Specifically, the STAREE anticipated age-specific death rates are as follows:

70-74y	10.0 per 1000 person-years
75-79y	17.7 per 1000 person-years
80-84y	33.6 per 1000 person-years
85+y	66.6 per 1000 person-years

These event rates have been informed by corresponding mortality rates from the ASPREE (Aspirin in Reducing Events in the Elderly) randomised clinical trial and from underlying Australian population death rates for 2011, obtained from http://www.abs.gov.au/AUSSTATS/abs@.nsf/DetailsPage/3302.02011?OpenDocument

Dementia

Population rates of dementia have been estimated as 11 per 1000 person years in the 75-59 year old age group although fluctuations have been observed. [67-69] For Australia, there is a paucity of national data to inform incidence rates, as noted by AIHW in 2012. The ASPREE trial provides a highly relevant contemporary source of information. A dementia rate of 6.8 per 1000 person years was observed in ASPREE (median age 74 years) although almost no cases occurred in the first year of follow up so the rate was higher in later years. In ASPREE, dementia was screened for using a 3MS reduction of at least 10 points and there was a 60% conversion of the screen-positives to confirmed cases of dementia. In STAREE a 3MS reduction of at least 5 points will be used, which is expected to approximately double the number of screen positives but lower the conversion rate to confirmed cases of dementia to 50%. From these considerations, 3MS screening in STAREE is expected to yield 33% more cases of dementia than in ASPREE. Given this, and detailed age-specific data from ASPREE, the dementia rates in the later years of follow up in STAREE are expected to be as follows, using participant age at randomisation:

70-74y	5.8 per 1000 person-years
75-79y	12.4 per 1000 person-years
80-84y	26.9 per 1000 person-years
85+y	36.6 per 1000 person-years

Physical Disability (defined as persistent loss of an activity of daily living)

This component is defined by a persistent “a lot of difficulty” or “unable to do the activity” rating, or “Yes” answer to receiving help to perform the same activity of daily living (ADL) domain/s (items 9 to 14), which is confirmed at least 6 months previously as loss of the same ADL. If it is not possible to obtain an ADL assessment, notification of admission to aged care or community assessment will initiate collection of relevant clinical records to substantiate a disability endpoint. The rate of this endpoint was negligible in the first year of ASPREE. The following rates are expected to be seen in later years of follow up in STAREE and are based on observed rates in ASPREE, using participant age at randomisation:

Zoungas S, *et al. BMJ Open* 2023; 13:e069915. doi: 10.1136/bmjopen-2022-069915

70-74y 4.5 per 1000 person-years
75-79y 7.6 per 1000 person-years
80-84y 12.5 per 1000 person-years
85+y 25.0 per 1000 person-years

Overlap of death, dementia and persistent physical disability

The overlap between these three components will be assumed to be 10%. This is consistent with the observed overlap between dementia and physical disability occurring prior to death in ASPREE. Hence the composite STAREE event rate is estimated to be the sum of the 3 component event rates less 10% of that sum.

The calculations of expected primary endpoint event rates in years 3-5 of follow up after randomisation are as follows:

70-74y: (10.0 + 5.8 + 4.5)*0.9 per 1000 person-years = 18.3 per 1000 person-years
75-79y: (17.7 + 12.4 + 7.6)*0.9 per 1000 person-years = 33.9 per 1000 person-years
80-84y: (33.6 + 26.9 + 12.5)*0.9 per 1000 person-years = 65.7 per 1000 person-years
85+y: (66.6 + 36.6 + 25.0)*0.9 per 1000 person-years = 115.4 per 1000 person-years

Consideration of healthy volunteer effect

It is anticipated that the event rates for disability-free survival calculated above will be reached by STAREE participants in years 3-5 following randomisation. In the initial 2 years of follow up participants are assumed to be healthy relative to the population, i.e. in the first 2 years of follow up STAREE participants are assumed to experience lower event rates. An event rate of 0 (zero) per 1000 person years is assumed in the first year of follow up for dementia and physical disability and a rate of 4 per 1000 person years is assumed for mortality. In the second year of follow up it is assumed that the primary endpoint event rate will be equal to half of that anticipated above in years 3-5.

Major adverse cardiovascular events (cardiovascular death, non-fatal myocardial infarction or non-fatal stroke) – co-primary endpoint B

This composite endpoint is defined as the first occurrence of cardiovascular death or non-fatal myocardial infarction or non-fatal stroke. In ASPREE this endpoint has been evaluated to the extent possible (of reported non-fatal myocardial infarction/angina events reported approximately 45% will be adjudicated as a non-fatal myocardial infarction). The figure below shows the estimated rate of major fatal and non-fatal cardiovascular events in ASPREE participants by age group. Of note, the underlying event rate seems fairly constant over the time from randomisation, i.e. the initial "healthy volunteer" effect that is seen in mortality and can be anticipated for admission to care, dementia and loss of activity of daily living, does not seem to apply to major adverse cardiovascular events. The three components, non-fatal myocardial infarction: non-fatal stroke: cardiovascular death, contribute events to the composite endpoint in the approximate ratio of 2:2:1 respectively.

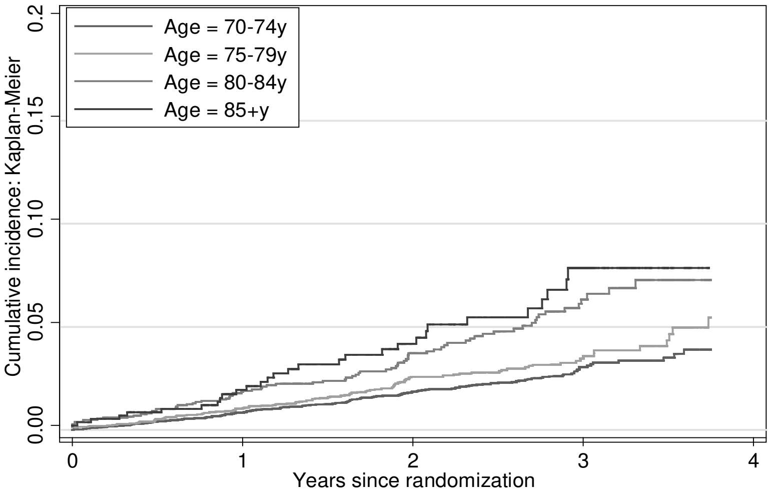

Based on the rates seen in ASPREE the following rates are anticipated in STAREE:

Years 1-5:

70-74y	9.5 per 1000 person-years
75-79y	11.5 per 1000 person-years
80-84y	18.5 per 1000 person-years
85+y	24.5 per 1000 person-years

Zoungas S, *et al. BMJ Open* 2023; 13:e069915. doi: 10.1136/bmjopen-2022-069915

13. Safety Monitoring and Reporting

13.1 Introduction

In STAREE the primary responsibility for the safety of individual participants will rest with the participant's usual general practitioner who is a co-investigator in the study. Additionally, an independent Data and Safety Monitoring Board (DSMB) will be appointed and will have primary responsibility for monitoring the study data for adverse trends and drug-related serious adverse events (SAEs). The responsibilities of the DSMB are outlined in the STAREE DSMB charter and DSMB plan.

13.2 Safety Monitoring

Several types of safety issues and SAEs may occur in STAREE participants. At each point of contact with participants, STAREE staff will specifically query participants for adverse events. Information on adverse events may also be reported to study staff between study visits by participants through contact with the trial centre by phone or email.

Participants will be encouraged to discuss any adverse events with their usual GP. GPs will be notified by STAREE staff of any abnormal test results and the STAREE medical officer will be available to discuss any issues with participants or their GPs.

13.2.2 Adverse events (AE)

The definition of an Adverse Event (AE) in the STAREE trial has been adapted from the National Institute of Aging definition. At the designated intervals for event collection, participants will be asked about specific events of interest as well as any new diseases or conditions. AEs will be obtained by self-report and some may require supporting documentation. AEs will be reported biannually to the Data Safety Monitoring Board (DSMB) by blinded treatment group.

AEs identified during monitoring will be classified by a trained medical officer or senior investigator as either serious or non-serious, and as potentially related or unrelated to study medication. If the AE is deemed related, therefore an adverse drug reaction (ADR or AR), further classification will occur regarding whether it is an expected or unexpected AR (see Appendix 5).

As the potential adverse effects of the active study drug in STAREE are well documented, AEs that are expected will not be considered as SAEs unless they meet the criteria for an SAE (section 11.2.3)

Source: AE definition adapted from the National Institute of Aging AE and SAE 2012 guidelines:
https://www.nia.nih.gov/sites/default/files/niaaeandsaeguidelinesfinal011012_0.doc

13.2.3 Serious adverse events (SAE)

Serious adverse events (SAEs) or Serious Adverse Drug Reactions (Serious ADRs) are defined as any unfavourable medical occurrence that:

- is fatal

Zoungas S, et al. BMJ Open 2023; 13:e069915. doi: 10.1136/bmjopen-2022-069915

- is life threatening [i.]
- requires in-patient-hospitalisation or prolongation of existing hospitalisation [ii.]
- causes persistent or significant disability/incapacity
- is a congenital anomaly /birth defect [iii.]
- is a medically important event or reaction [iv.] or
- is an accidental or intentional overdose.

Source: National Statement on Ethical Conduct in Human Research 2007; National Health and Medical Research Council Safety Monitoring and Reporting in Clinical Trials involving Therapeutic Goods November 2016

i. Life Threatening SAE: An SAE is considered life threatening when it places the participant at immediate risk of death from the event as it occurred.

ii. Hospitalisation: A hospitalisation is to be considered an SAE only if it is an official admission (inpatient) with a duration of more than 24hr or a minimum of 2 calendar days where exact time of stay is unavailable.

iii. Not applicable, since STAREE participants are aged 70 years or over at study entry

iv. In the opinion of the responsible investigator (i.e. an event that is not immediately life threatening, does not result in death or hospitalisation, but which may jeopardise the participant or require intervention to prevent one of the other SAEs listed above)

13.2.4 Suspected Unexpected Serious Adverse Events (SUSARs)

A SUSAR is defined as a SAE that is:

- not a predefined cardiovascular endpoint (see section 5),
- is unexpected (taking into account drug labelling and the participant history),
- is causally associated with the study medication based on a determination by a medical officer of 'definite', 'probable' or 'possible' using the Naranjo scale.

All SUSARs will be adjudicated by a study medical officer to determine if the event is unexpected in terms of both medication labelling and the participant's history.

A non-endpoint event will be deemed to be a SUSAR if:

- Evidence exists to support the possible association of the event with either or both of the medications (i.e. either the project office or the investigator has assessed the event as possibly causally related to blinded study medication); and
- The event is unexpected in terms of the product labelling for either product; and
- The event is unexpected in terms of the subject's history; and
- The event is serious as defined in section 11.2.

Zoungas S, *et al. BMJ Open* 2023; 13:e069915. doi: 10.1136/bmjopen-2022-069915

13.3 Safety reporting

In general, only AEs pre-specified to be of specific interest and those that are deemed to be serious will be reportable. AEs of interest are diabetes, myopathy (muscle symptoms) and liver impairment.

All SAEs will be reported from the study sites directly to the study medical officer within 24 hours of their notification to the clinical sites (study centres).

SAEs will be reviewed by the medical officer and adjudicated as related or unrelated to study medication, and as expected or unexpected serious adverse events.

All SAEs and SUSARS will be reported by the study centre to the ethics committees and DSMB according to the following schedule:

- Related and expected SAEs, will be reported to the Human Research and Ethics Committee (HREC)
- Related and unexpected SUSARs will be reported to the HREC within 72 hours and to the RACGP and TGA within 15 days and followed up with detailed reports when available
- Unrelated SAEs will be reported to the STAREE DSMB bi-annually

All SAEs and SUSARs will be reported to the DSMB in summary format by blinded treatment arm (A and B).

The data and safety monitoring reports will be prepared by an independent statistician.

13.4 Adverse Events of Interest

13.4.1 New diagnosis of diabetes

Diabetes as diagnosed on the basis of:

1) an annual HbA1c or fasting plasma glucose test using the WHO criteria (a HbA1c value of 6.5% or higher or a fasting plasma glucose of 7.0 mmol/L or higher, with confirmation by a second test within 4 weeks; or

2) community-based testing during usual clinical care (GP or other clinicians e.g. hospitalisation with a hyperglycaemic crisis or classic symptoms of hyperglycaemia and a random plasma glucose test of 11.1 mmol/L and/or commencement of glucose lowering medication for glucose control).

13.4.2 New diagnosis of myopathy

All participants will be advised to discuss a new diagnosis of myopathy with their usual treating GP. The decision to cease or reduce the study medication will be made by the GP in consultation with the participant. The participant will continue in the study on a reduced dose or remain off study medication and have routine follow up (study visits and phone calls) for observational purposes (see section 7.1).

13.4.3 Liver impairment

Liver impairment defined as:

1) an elevated ALT and/or AST across two or more tests (elevations greater than 3x ULN and elevated bilirubin (2x ULN)) and

2) no other demonstrable cause.

14. Adherence to Ethical, Regulatory and Administrative Considerations

14.1 Ethical considerations

14.1.1 General

This study will be conducted in accordance with the Declaration of Helsinki 1964 as revised in Edinburgh in 2000 and with the National Health & Medical Research Council (NHMRC) Guidelines on Human Experimentation.

14.1.2 Ethics Committee Approval

The primary ethics committee for the study is the Monash University Human Research Ethics Committee (Project ID 2787 and 21528).

The study has also been reviewed and approved by the following ethics committees:
Curtin University Human Research Ethics Committee (HR113/2015)
RACGP National Research and Evaluation Ethics Committee (14 - 017)
Tasmanian Health and Medical Research Ethics Committee (H0014918)
University of Newcastle Human Research Ethics Committee (H-2016-0266)

14.1.3 Information for participants

Before obtaining consent from the participant they must be informed of the objectives, benefits, risks and requirements of the study, as well as the nature of the study medication. A participant consent form will be provided to every participant prior to baseline visit 1 (posted if time permits) or at baseline visit 1.

14.1.4 Informed consent

a) All participants must give their written informed consent at baseline visit 1 to continue in the study.
b) Informed consent is obtained from the participant by the STAREE Field Officers. The study staff should fully inform the participant of all pertinent aspects of the STAREE study by reviewing the study information and consent form. All participants should be informed to the fullest extent possible about the study, in language and terms they are able to understand.
c) Prior to a participant’s involvement in the study, the written Informed Consent Form should be signed, name filled in and personally dated by the participant and by the staff member who conducted the informed consent discussion. A copy of the signed and dated written Informed Consent Form will be provided to the participant. The original consent form is to be stored in the participant’s individual study file, held by the investigator.
d) The form used for obtaining the participant's informed consent must be the current version that has been reviewed and approved by the appropriate Ethics Committee.

14.1.5 Protocol Amendments

All amendments and revisions to the trial protocol will be submitted to the Monash University Human Research Ethics Committee and on approval will be communicated to STAREE investigators and relevant staff through internal processes such as email and group meetings. All updates will be chronologically documented in the current version of the protocol.

14.1.6 Confidentiality

All study-related information will be stored securely at the study sites. All participant information will be stored in locked filing cabinets in areas accessible only by STAREE personnel. All participant CRFs will contain a coded identification number only to maintain participant confidentiality. All records that contain names or other personal identifiers, such as informed consent forms, will be stored separately from study records identified by code number. All local databases will be secured with password-protected access systems. Other forms and lists, that link participant ID numbers to other identifying information will be stored in a separate, locked file in an area with limited access. Hardcopy information will be uploaded into a secure study database housed in the high security Monash University Data Centre at Clayton, Melbourne. All information collected for this project will be retained for a period of no less than 15 years following the completion of the project. Hardcopy information will be shredded and destroyed, and electronic data will be deleted from the secure STAREE database after a period of no less than 15 years after the completion of the project, in accordance with the Monash University Human Research Ethics Committee recommendations for drug trials and Good Clinical Practice Guidelines.

14.2 Regulatory considerations

14.2.1 Financing

Monash University is the sponsor of the STAREE trial.

STAREE has been awarded project grants from the NHMRC (Australia) (APP1068146 and APP1161503) and from the Heart Foundation (#101663). STAREE MIND and STAREE HEART have been awarded NHMRC grant funding (#2006611 and #1165440).

14.2.2 Trial registration

STAREE is registered with clinicaltrials.gov (Identifier NCT02099123). First posted March 28, 2014.

14.2.3 Declaration of interest

The STAREE investigators have established a policy regarding Conflict of Interest. The policy aims to protect the integrity of the project's decision-making process and to manage risk. The policy requires full disclosure by all of the investigators of their, and their immediate families, financial relationships with all pharmaceutical and biomedical companies judged to have an active or potential interest in the conduct and outcome of the study. The disclosures of each project investigator will be documented in all publications.

14.3 Indemnity

Monash University shall at all times indemnify the study investigators and their staff from claims that may be made against them for any injury sustained by a study participant as a consequence of the follow up for this study as outlined in this protocol. GPs insurers will be asked to indemnify the GP co-investigators who are participating in the study. Ramsay Healthcare will indemnify the study medication.

14.4 Data Access

The Data Access and Publications Committee will oversee the intra-study data sharing process, with input from the STAREE Executive Committee.

Access to STAREE data for analysis, preparation and submission of publications and presentations will be decided according to the STAREE Data Access Policy and the STAREE Publications Policy.

The policies are based on the following principles:

- STAREE data is to be used for the purposes of furthering the public good in relation to health.
- STAREE data is a collaborative resource. Data elements cannot be reserved by researchers for exclusive use.
- STAREE data is to be used for projects that are scientifically, methodologically and ethically sound.
- The privacy of STAREE participants is to be protected.

14.5 Governance

Central Coordinating Centre

The STAREE Central Coordinating Centre is located at the School of Public Health and Preventive Medicine, Monash University, Melbourne.

Steering Committee (SC)

STAREE is overseen and coordinated by a steering committee comprised of Prof Sophia Zoungas (Chair), Monash University; Prof John McNeil, Monash University; Prof Andrew Tonkin, Monash University; Prof Rory Wolfe, Monash University; Prof Christopher Reid, Monash University; Prof Mark Nelson, University of Tasmania; Prof Lawrie Beilin, University of Western Australia; Prof Stephen Nicholls, Monash University; Associate Professor Trevor Chong, Monash University, Prof Geoffrey Cloud, Monash University, Dr Stephanie Ward, Monash University and Prof Anthony Wierzbicki (King's College, UK).

The Steering Committee will provide oversight to the overall policy and direction of the project. It will be responsible for protocol generation and, where appropriate, modification, budgeting and all funding applications. The Steering Committee will meet regularly throughout the planning, execution and data analysis phases of the project, when it will consider reports from the Study Director and the EAC. The Steering Committee will be responsible for all the publications and communications relating to the study.

Zoungas S, *et al. BMJ Open* 2023; 13:e069915. doi: 10.1136/bmjopen-2022-069915

STAREE Governance Structure

14.6 Administrative Organisation

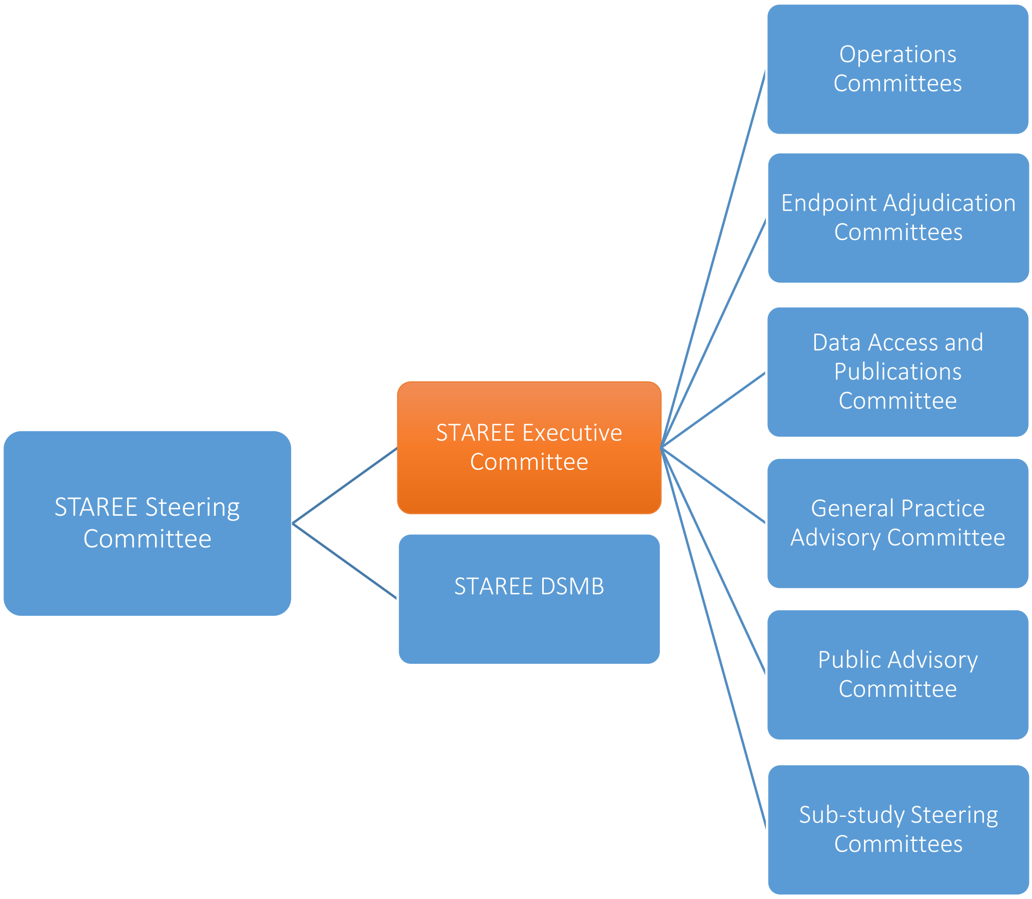

14.6.1 Individual and committee responsibilities

Principal Investigators: Prof Sophia Zoungas and Prof John McNeil

STAREE Executive Committee

The STAREE Executive Committee will be responsible for the overall design, management and conduct of STAREE including preparation of the protocol and revisions, preparation of investigators brochure and case report forms (CRFs), publication of study reports, approving the operational plan for the study, and financial management of the trial. The committee will monitor the progress of recruitment against targets, and if necessary, institute remedial actions. It will also review the rates of adherence to randomised treatments and the data from the quality assurance program.

National Operations Committee

The National Operations Committee will be responsible for implementing the study, monitoring the progress of recruitment and ensuring adherence and standardisation through the study. It will also need to ensure the coordination, consistency and transfer of data to the Data Management Centre. The committee will ensure quality control review of laboratory

data, clinical measurements and data collection, completeness and entry times of data, site monitoring and source documentation. These committees may consist of the Principal Investigator or Project Managers and other key research personnel who will report directly to the Steering Committee.

Internal Data Monitoring Committee (DMC)

The STAREE Internal Data Monitoring Committee (DMC) is responsible for reviewing information related to the conduct of the trial and monitoring activity as per STAREE's Monitoring Plan, Monash University's Research Data Management Guidelines and the NHMRC's Australian Code for the Responsible Conduct of Research. The STAREE Internal DMC shall make recommendations to the Monash University STAREE Executive Committee. Recommendations shall include all aspects of Data Monitoring as per the STAREE Monitoring Plan.

DMC members will be appointed by the Executive and Management of the STAREE Clinical Trial and may be involved in the study's collection and management of data, including data monitoring. Appointed committee members will include a Committee Chair, Data Statistician and an individual who has had previous experience with a DMC or experience with trials of medicinal investigational products.

Data Safety and Monitoring Board

The Data Safety and Monitoring Board (DSMB) will monitor the establishment and implementation of the STAREE Data Management System and review all safety, data and quality assurance reports for the study. The DSMB chair will report to the Steering Committee of STAREE on data management issues associated with the study and will provide recommendations to the Steering Committee in relation to subject recruitment, event rate tracking, monitoring adverse events and conducting interim analyses. The committee will operate in accordance with the DSMB Charter and Plan.

The DSMB will have responsibility for monitoring quality control of the data, progress of recruitment and safety aspects of the trial. The DMC will present data for review by the DSMB by a blinded treatment arm (e.g., 'A' and 'B'). The DSMB will monitor all-cause mortality rates in the two randomised groups. Additionally one pre-specified interim analysis may occur when an average of 3.25 years of follow up per participant has accrued and >50% of the primary endpoints have occurred. The DSMB will be responsible for reviewing any interim analysis on unblinded data and providing recommendations to the trial executive committee. The DSMB will review deaths, serious adverse events and other endpoint data on a periodic basis. Administrative support will be provided by an independent biostatistician and research assistant.

The DSMB will be provided with data every 6 months or as requested. The DSMB will meet annually for the first 2 years and thereafter will meet twice annually, by teleconference or in-person where possible to review study progress, data quality control, address policy issues and review total mortality data, adverse events and all safety data and monitor the study progress and data quality. The DSMB has expertise related to the conduct of clinical trials *per se* and in the primary care sector, the elderly, epidemiology, biostatistics, clinical pharmacology, clinicians, and cardiovascular disease. Confidentiality will be maintained

during all phases of the trial including monitoring, preparation of interim results, review, and response to monitoring recommendations.

Endpoint Adjudication Committees (EACs)
The Endpoint Adjudication Committees (EACs) will be responsible for developing adjudication criteria and processes to capture key data required for the identification and adjudication of the following study endpoints: All-cause mortality, myocardial infarction, heart failure, stroke, and dementia. The EACs will operated in accordance with the Endpoint Adjudication Committee Charter.

The following EACs will be responsible for the assessment of suspected endpoints through examination of relevant clinical information and for their adjudication according to the pre-specified endpoint definitions.

Death Subcommittee
Dementia Subcommittee
Heart Disease (Myocardial Infarction) Subcommittee
Heart Disease (Heart Failure) Subcommittee
Stroke Subcommittee

The EAC subcommittees will be chaired by specialist clinicians with expertise to oversee adjudication of the relevant endpoints. All EAC members will be selected by the STAREE Executive Committee and will be appointed for the duration of the study. Administrative support, training and source documentation required for EAC members to carry out their roles will be provided by the EAC Coordinator and STAREE study staff.

Data Access and Publications Committee
The Data Access and Publications Committee will be responsible for developing and implementing the data access, sub-studies and ancillary studies, and publication policies. It will oversee the processes of receiving, reviewing and approving applications from researchers for STAREE baseline data, for conduct of sub-studies and ancillary studies, and requests for publication, approval for abstracts and submission to conferences. Authorship of publications will be guided by the STAREE data access and publications policies.

General Practice Advisory Committee
The General Practice Advisory Committee will advise the National Operations Committee and other committees on aspects of the trial related to family practice.

Public Advisory Group
The STAREE Public Advisory Group will be a forum to facilitate the flow of information from the STAREE clinical trial to the public. It will also provide opportunities for members of the group to provide feedback and input from the community perspective on how information is disseminated to the public about the STAREE trial.

STAREE Healthy Ageing Biobank Steering committee
Access and utilisation of the biospecimens stored in the STAREE Healthy Ageing Biobank will be governed by the STAREE Healthy Ageing Biobank steering committee. The STAREE

Healthy Ageing Biobank steering committee will review detailed proposals outlining the research to be undertaken, and access to biospecimens will only be granted if the proposals are assessed to have scientific merit and are adequately funded. Accurate records of the aliquots of each stored biospecimen will be achieved through the use of a Nunc Next Generation CryoTube Scanner and software. The software will be utilised to track each sample which will include storage or retrieval of a specimen by an approved researcher. Any secondary research which uncovers significant genetic information will be notified to the Biobank Steering committee, which will determine who will be notified about these results.

STAREE MIND Steering committee

The STAREE MIND Steering Committee will oversee integration of STAREE MIND with the parent trial. It will be responsible for design, management and conduct of STAREE MIND including preparation of the protocol, investigators brochures and case report forms (CRFs), publication of study reports, approval of the operational plan, financial management and will monitor the progress of recruitment. The STAREE MIND committee will report on progress to the STAREE Steering and Executive committees.

STAREE HEART Steering committee

The STAREE HEART Steering Committee will oversee integration of STAREE HEART with the parent trial. It will be responsible for design, management and conduct of STAREE HEART including preparation of the protocol, investigators brochures and case report forms (CRFs), publication of study reports, approval of the operational plan, financial management and will monitor the progress of recruitment. The STAREE HEART committee will report on progress to the STAREE Steering and Executive committees.

Zoungas S, *et al. BMJ Open* 2023; 13:e069915. doi: 10.1136/bmjopen-2022-069915

14. Data Management and Training

14.1 Case report forms

All data from assessments conducted during the study will be recorded on an appropriate case report form (CRF) for each participant. Electronic CRFs will be accessible from the STAREE website. The CRFs must be completed during or after each participant visit (or as soon as all data is available, e.g. once pathology results are obtained) and uploaded onto the STAREE web-based portal. Original hard-copy CRFs and other source documents will be stored in the participant's file at the local study site.

CRFs will be kept current to reflect the participant's course throughout the study. Participants will not be identified on the CRF by name. Appropriately coded identification (GP, Practice and Subject Identification Numbers) and an acrostic of the participant's initials will be used.

The data from the CRFs will be uploaded onto the STAREE web-based data management portal. All information collected will be treated in accordance with professional and ethical guidelines. All corrections and alterations of data on the CRFs must be made according to the instructions provided and must be dated and initialled.

14.2 Data Management

Data collection and management will be centralised at the STAREE data management centre, Department of Epidemiology and Preventive Medicine, Monash University, Melbourne. Study data collected at local study sites will be entered on electronic CRFs with source documentation retained in the participant file. All information related to potential endpoints will be copied from the original documents sourced through clinical notes and hospital medical records and sent de-identified to the STAREE data management centre. Serious adverse event and endpoint data will be accepted as either a) photocopied clinical records or b) scanned or photographed image files of clinical records. The clinic will retain all original information related to the clinical event or endpoint in the subject files.

Electronic study records will be accessible through the internet at the STAREE web-portal. Access to this website will be made available to registered study staff via password protected log-in procedures.

Monash University implements a defence in depth approach to information security and employs a multitude of controls to protect the study's infrastructure and data. These controls are regularly audited to ensure they meet global best practices and are aligned with ISO 27001 security practices. Data collected as part of the STAREE clinical trial will be stored on University managed secure and resilient infrastructure located in Australia that complies with all applicable data protection and privacy obligations. Some specialist data collected as part of the STAREE clinical trial may be entered into applications that are operated by external third parties. This will only be permitted where all applicable data protection and privacy obligations are in place with those external third parties.

Monash University values the privacy of every individual's personal information and is committed to the protection of that information from unauthorised use and disclosure, except where permitted by law. Monash University is subject to and ensures that personal

Zoungas S, *et al. BMJ Open* 2023; 13:e069915. doi: 10.1136/bmjopen-2022-069915

and health information it holds or has access to, is handled in accordance with data protection and privacy legislation, including the Privacy and Data Protection Act 2014 (Vic) and the Health Records Act 2001 (Vic) and where required, the Privacy Act 1988 (Cth) and applicable international regulations.

14.3 Data analysis

The data will be analysed by statisticians from the Biostatistics Unit, Department of Epidemiology and Preventive Medicine, Monash University.

Baseline characteristics of the two treatment groups will be tabulated and imbalance defined as a 0.25 standard deviation difference in means (quantitative characteristics) or an odds ratio of 1.5 (binary characteristics).

All comparisons between treatment arms will be on an intention-to-treat basis i.e. according to the group to which participants were randomised and without reference to their actual compliance with assigned treatment.

Each of the co-primary endpoints will be analysed separately in time-to-event analyses. Event rates (time to first event within each endpoint definition) will be compared between groups using a hazard ratio, 95% confidence interval and two-sided p-value from a Cox proportional hazards regression model fitted to the endpoint with censoring for non-events at their most recent study visit and a single covariate being an indicator of the group to which the individual was randomised, statin or placebo. The proportional hazards assumption will be tested for each model.

A gate-keeping procedure will be used to adjust for the multiple testing created by the presence of two co-primary endpoints. First, MACE will be tested at alpha=0.05 and, if the MACE p-value is <0.05 then second, disability-free survival will be tested at alpha=0.05.

Secondary analyses of the co-primary endpoints will be performed using extended Cox proportional hazards regression models based on time(s) to any event within the endpoint definition (for relevant endpoints that can occur more than once).

No statistical adjustment will be made for the multiple secondary endpoints but the reporting of all secondary endpoint analyses will make clear whether any of the co-primary endpoints were statistically significant and will state the number of secondary endpoints proposed a priori in the study protocol.

In the survival analyses, loss to follow up will be considered a censoring event. This equates to an assumption that data is missing at random given the participant's treatment group and the timing of their loss to follow up. The adequacy of this assumption will be checked in sensitivity analyses that will include both imputation approaches and adjustment for baseline covariates predictive of propensity for dropout. The analyses will be performed using commercial statistical software packages (Stata or SAS).

Zoungas S, *et al. BMJ Open* 2023; 13:e069915. doi: 10.1136/bmjopen-2022-069915

14.4 Training

All STAREE staff will be trained by senior study staff and study investigators and undertake a competency assessment prior to commencing their specific role.

All Staff will be provided with background information on the rationale of the STAREE trial and the study protocol. They will also be required to familiarise themselves with the key principles of Good Clinical Practice (GCP) and will attend an accredited GCP training course.

All study activities are described in standard operating procedures (SOPs), and staff will be regularly encouraged to refer to SOPs and SOP updates for guidance.

14.4.1 Research Officers

STAREE Research Officers are responsible for and will be trained in the conduct of phone screening for potential study participants, in use of the STAREE database, booking and confirming participant visits, conducting 6 month participant phone calls and labelling and dispatch of medication, receipt of study medication and follow up missing documents for randomisation and endpoint ascertainment.

14.4.2 GP Liaison Officers

STAREE GP Liaison Officers are responsible for engaging with general practices and registering GPs as STAREE co-investigators. They will be provided with practical guidance in conducting GP practice visits to recruit GPs, in data entry, and in searching GP practice databases for potential participants.

14.4.3 Research Field Officers

STAREE Field Officers are responsible for conducting baseline screening visits and annual follow up visits with study participants. They will receive training in database entry, conducting informed consent discussions with potential participants, visit conduct and performing specific study assessments (e.g. weight, height and blood pressure measurement and taking personal and medical histories) and assessing the suitability of a participant to participate in the trial.

Field Officers will observe baseline 1 and 2 and annual visits that are being conducted by a trained Field Officer to become familiar with the visit procedures. Training in the conduct of cognitive assessments will be provided in the form of training videos, SOPs and role plays. Before Field Officers can conduct independent study visits, they will undertake specific training in cognitive assessments with a Senior Field Officer and specialist STAREE Investigator and conduct 3-6 study visits under the supervision from a Senior Field Officer.

Accreditation to perform study visits will be provided subject to satisfactory performance in both the cognitive assessment training and supervised visit conduct. To maintain accreditation, Field Officers will be required to conduct a minimum of 4 visits per month (at least 2 with cognitive assessments) and performance will be reviewed annually.

Zoungas S, *et al. BMJ Open* 2023; 13:e069915. doi: 10.1136/bmjopen-2022-069915

15. Study Monitoring and Auditing

Monitoring and auditing in STAREE will include monitoring of investigative sites and visit conduct by study staff. This will involve monitoring of all study documentation and activities including participant files, data quality, administrative activities and endpoint collection as detailed further in the STAREE monitoring plan.

All STAREE study sites will be monitored annually by trained STAREE clinical research Site Monitors, following GCP guidelines. Site monitoring will be undertaken at all STAREE sites to ensure that the study is being conducted according to the protocol and appropriate regulatory requirements, that protocol deviations are appropriately documented and reported, the site complies with safety regulations and that personnel at each site meet GCP obligations.

All field staff involved in the STAREE study will have their activity reviewed by the Visit Conduct Monitor. Monitoring will ensure that all visit activity is conducted in accordance with the study protocol, relevant SOPs and test administration SOPs.

Study monitors will submit written reports summarising their findings to the internal Data Monitoring Committee (DMC). Where monitoring identifies risks or issues for participant safety or trial processes, appropriate steps will be taken to remedy the issue including re-training of staff and repeat monitoring.

Zoungas S, *et al. BMJ Open* 2023; 13:e069915. doi: 10.1136/bmjopen-2022-069915

16. Dissemination Plan

The STAREE dissemination plan will ensure that the outputs from the research inform general practice and maximise the benefit to older people.

The key audiences for the STAREE results are:

- General practitioners
- Physicians and other health professionals/clinicians
- STAREE participants
- The general public (including community groups such as Council of the Older Australian, Probus groups etc.)
- Professional organisations (e.g. RACGP)
- Funding organisations (e.g. NHMRC, Monash University)
- Scientific and medical research communities

Representatives of these key stakeholder groups will be invited to take part in discussion of the STAREE findings in relation to current clinical practice and policy and how to best translate the research findings into recommendations for wider audiences.

Multiple channels of communication will be used to disseminate STAREE findings to the wider community. These will include the following:

- Written feedback to STAREE participants and GP co-investigators on the study results and recommendations arising from the study.
- Published academic journal articles, plain English summaries, research summaries for professional journals, newsletters, presentations, and frequently asked questions section will be made accessible on the STAREE website.
- Workshops and presentations at scientific meetings, GP conferences and community and public venues.

17. References

1. Bongaarts J. Human population growth and the demographic transition. Philos Trans R Soc Lond B Biol Sci. 2009;364(1532):2985-90.
2. Christensen K, Doblhammer G, Rau R, Vaupel JW. Ageing populations: the challenges ahead. Lancet. 2009;374(9696):1196-208.
3. Jacobsen L, Mather M, Lee M, Kent M. America's Ageing Population. Population Bulletin. 2011;66:2-3.
4. Productivity Commission 2011, Caring for Older Australians: Overview, Report No. 53, Final Inquiry Report, Canberra.
5. Zoungas S, Curtis A, Tonkin A, McNeil J. Statins in the elderly: an answered question? Curr Opin Cardiol. 2014;29(4):372-80.
6. Jonsson A, Sigvaldason H, Sigfusson N. Total cholesterol and mortality after age 80 years. Lancet. 1997;350(9093):1778-9.
7. Schatz IJ, Masaki K, Yano K, Chen R, Rodriguez BL, Curb JD. Cholesterol and all-cause mortality in elderly people from the Honolulu Heart Program: a cohort study. Lancet. 2001;358(9279):351-5.
8. Volpato S, Leveille SG, Corti MC, Harris TB, Guralnik JM. The value of serum albumin and high-density lipoprotein cholesterol in defining mortality risk in older persons with low serum cholesterol. J Am Geriatr Soc. 2001;49(9):1142-7.
9. Weverling-Rijnsburger AW, Jonkers IJ, van Exel E, Gussekloo J, Westendorp RG. High-density vs low-density lipoprotein cholesterol as the risk factor for coronary artery disease and stroke in old age. Arch Intern Med. 2003;163(13):1549-54.
10. Foody JM, Wang Y, Kiefe CI, Ellerbeck EF, Gold J, Radford MJ, et al. Long-term prognostic importance of total cholesterol in elderly survivors of an acute myocardial infarction: the Cooperative Cardiovascular Pilot Project. J Am Geriatr Soc. 2003;51(7):930-6.
11. Schupf N, Costa R, Luchsinger J, Tang MX, Lee JH, Mayeux R. Relationship between plasma lipids and all-cause mortality in nondemented elderly. J Am Geriatr Soc. 2005;53(2):219-26.
12. Prospective Studies C, Lewington S, Whitlock G, Clarke R, Sherliker P, Emberson J, et al. Blood cholesterol and vascular mortality by age, sex, and blood pressure: a meta-analysis of individual data from 61 prospective studies with 55,000 vascular deaths. Lancet. 2007;370(9602):1829-39.
13. Stancu C, Sima A. Statins: mechanism of action and effects. J Cell Mol Med. 2001;5(4):378-87.
14. Nicholls SJ, Tuzcu EM, Sipahi I, Grasso AW, Schoenhagen P, Hu T, et al. Statins, high-density lipoprotein cholesterol, and regression of coronary atherosclerosis. JAMA : the journal of the American Medical Association. 2007;297(5):499-508.
15. Maron DJ, Fazio S, Linton MF. Current perspectives on statins. Circulation. 2000;101(2):207-13.
16. Greenwood J, Mason JC. Statins and the vascular endothelial inflammatory response. Trends Immunol. 2007;28(2):88-98.
17. Efficacy and safety of statin therapy in older people: a meta-analysis of individual participant data from 28 randomised controlled trials. Lancet. 2019;393(10170):407-15.
18. Cholesterol Treatment Trialists C, Mihaylova B, Emberson J, Blackwell L, Keech A, Simes J, et al. The effects of lowering LDL cholesterol with statin therapy in people at low risk of vascular disease: meta-analysis of individual data from 27 randomised trials. Lancet. 2012;380(9841):581-90.

19. Afilalo J, Duque G, Steele R, Jukema JW, de Craen AJ, Eisenberg MJ. Statins for secondary prevention in elderly patients: a hierarchical bayesian meta-analysis. Journal of the American College of Cardiology. 2008;51(1):37-45.
20. Shepherd J, Blauw GJ, Murphy MB, Bollen EL, Buckley BM, Cobbe SM, et al. Pravastatin in elderly individuals at risk of vascular disease (PROSPER): a randomised controlled trial. Lancet. 2002;360(9346):1623-30.
21. Han BH, Sutin D, Williamson JD, Davis BR, Piller LB, Pervin H, et al. Effect of Statin Treatment vs Usual Care on Primary Cardiovascular Prevention Among Older Adults: The ALLHAT-LLT Randomized Clinical Trial. JAMA internal medicine. 2017;177(7):955-65.
22. Glynn RJ, Koenig W, Nordestgaard BG, Shepherd J, Ridker PM. Rosuvastatin for primary prevention in older persons with elevated C-reactive protein and low to average low-density lipoprotein cholesterol levels: exploratory analysis of a randomized trial. Annals of internal medicine. 2010;152(8):488-96, w174.
23. Ridker PM, Lonn E, Paynter NP, Glynn R, Yusuf S. Primary Prevention With Statin Therapy in the Elderly: New Meta-Analyses From the Contemporary JUPITER and HOPE-3 Randomized Trials. Circulation. 2017;135(20):1979-81.
24. Routledge PA, O'Mahony MS, Woodhouse KW. Adverse drug reactions in elderly patients. Br J Clin Pharmacol. 2004;57(2):121-6.
25. Brugts JJ, Yetgin T, Hoeks SE, Gotto AM, Shepherd J, Westendorp RG, et al. The benefits of statins in people without established cardiovascular disease but with cardiovascular risk factors: meta-analysis of randomised controlled trials. BMJ. 2009;338:b2376.
26. Bellosta S, Paoletti R, Corsini A. Safety of statins: focus on clinical pharmacokinetics and drug interactions. Circulation. 2004;109(23 Suppl 1):III50-7.
27. Graham DJ, Staffa JA, Shatin D, Andrade SE, Schech SD, La Grenade L, et al. Incidence of hospitalized rhabdomyolysis in patients treated with lipid-lowering drugs. JAMA : the journal of the American Medical Association. 2004;292(21):2585-90.
28. Davidson MH, Robinson JG. Safety of aggressive lipid management. Journal of the American College of Cardiology. 2007;49(17):1753-62.
29. Sathasivam S. Statin induced myotoxicity. European journal of internal medicine. 2012;23(4):317-24.
30. Hilmer S, Gnjidic D. Statins in older adults. Australian Prescriber. 2013;36(3):79-82.
31. Sattar N, Preiss D, Murray HM, Welsh P, Buckley BM, de Craen AJ, et al. Statins and risk of incident diabetes: a collaborative meta-analysis of randomised statin trials. Lancet. 2010;375(9716):735-42.
32. FDA drug safety communication: Important safety label changes to cholesterol-lowering statin drugs 2012.
33. Cholesterol Treatment Trialists C, Emberson JR, Kearney PM, Blackwell L, Newman C, Reith C, et al. Lack of effect of lowering LDL cholesterol on cancer: meta-analysis of individual data from 175,000 people in 27 randomised trials of statin therapy. PloS one. 2012;7(1):e29849.
34. Ott BR, Daiello LA, Dahabreh IJ, Springate BA, Bixby K, Murali M, et al. Do Statins Impair Cognition? A Systematic Review and Meta-Analysis of Randomized Controlled Trials. Journal of general internal medicine. 2015.
35. Catapano AL, Graham I, De Backer G, Wiklund O, Chapman MJ, Drexel H, et al. 2016 ESC/EAS Guidelines for the Management of Dyslipidaemias. European heart journal. 2016;37(39):2999-3058.

Zoungas S, *et al. BMJ Open* 2023; 13:e069915. doi: 10.1136/bmjopen-2022-069915

36. Naci H, Brugts J, Ades T. Comparative tolerability and harms of individual statins: a study-level network meta-analysis of 246 955 participants from 135 randomized, controlled trials. Circ Cardiovasc Qual Outcomes. 2013;6(4):390-9.
37. Bays H, Cohen DE, Chalasani N, Harrison SA. An assessment by the Statin Liver Safety Task Force: 2014 update. Journal of clinical lipidology. 2014;8(3):S47-S57.
38. Lindsley CW. 2012 trends and statistics for prescription medications in the United States: CNS therapeutics continue to hold leading positions. ACS chemical neuroscience. 2013;4(8):1133-5.
39. PBS Information Management Section. Pharmaceutical Policy Branch. Expenditure and prescriptions twelve months to 30 June 2014.
40. Simons LA, Ortiz M, Calcino G. Long term persistence with statin therapy -- experience in Australia 2006-2010. Australian family physician. 2011;40(5):319-22.
41. Trusler D. Statin prescriptions in UK now total a million each week. Bmj. 2011;343:d4350.
42. Sheppard JP, Singh S, Fletcher K, McManus RJ, Mant J. Impact of age and sex on primary preventive treatment for cardiovascular disease in the West Midlands, UK: cross sectional study. Bmj. 2012;345:e4535.
43. Chokshi NP, Messerli FH, Sutin D, Supariwala AA, Shah NR. Appropriateness of statins in patients aged >/=80 years and comparison to other age groups. The American journal of cardiology. 2012;110(10):1477-81.
44. Robinson JG, Booth B. Statin use and lipid levels in older adults: National Health and Nutrition Examination Survey, 2001 to 2006. Journal of clinical lipidology. 2010;4(6):483-90.
45. Morgan TK, Williamson M, Pirotta M, Stewart K, Myers SP, Barnes J. A national census of medicines use: a 24-hour snapshot of Australians aged 50 years and older. The Medical journal of Australia. 2012;196(1):50-3.
46. National Vascular Disease Prevention Alliance. Guidelines for the management of absolute cardiovascular disease risk. 2012.
47. Shepherd J, Cobbe SM, Ford I, Isles CG, Lorimer AR, MacFarlane PW, et al. Prevention of coronary heart disease with pravastatin in men with hypercholesterolemia. West of Scotland Coronary Prevention Study Group. The New England journal of medicine. 1995;333(20):1301-7.
48. Stone NJ, Robinson JG, Lichtenstein AH, Bairey Merz CN, Blum CB, Eckel RH, et al. 2013 ACC/AHA guideline on the treatment of blood cholesterol to reduce atherosclerotic cardiovascular risk in adults: a report of the American College of Cardiology/American Heart Association Task Force on Practice Guidelines. Journal of the American College of Cardiology. 2014;63(25 Pt B):2889-934.
49. East Melbourne: Royal Australian College of General Practitioners. Guidelines for preventative activities in general practice. (2012).
50. NICE. Chronic Kidney Disease - Early identification and management of chronic kidney disease in adults in primary and secondary care. (2014).
51. Collins R, Reith C, Emberson J, Armitage J, Baigent C, Blackwell L, et al. Interpretation of the evidence for the efficacy and safety of statin therapy. The Lancet. 2016;388(10059):2532-61.
52. Ridker PM, Pradhan A, MacFadyen JG, Libby P, Glynn RJ. Cardiovascular benefits and diabetes risks of statin therapy in primary prevention: an analysis from the JUPITER trial. Lancet. 2012;380(9841):565-71.

53. Ganga HV, Slim HB, Thompson PD. A systematic review of statin-induced muscle problems in clinical trials. American heart journal. 2014;168(1):6-15.
54. Bland RC, Newman SC. Mild dementia or cognitive impairment: the Modified Mini-Mental State examination (3MS) as a screen for dementia. Canadian journal of psychiatry Revue canadienne de psychiatrie. 2001;46(6):506-10.
55. Radloff LS. The CES-D Scale: A Self-Report Depresion Scale for Research in the General Population. Applied Psychological Measurement. 1977;1(3):385-401.
56. Manini TM, Beavers DP, Pahor M, Guralnik JM, Spring B, Church TS, et al. Effect of Physical Activity on Self-Reported Disability in Older Adults: Results from the LIFE Study. J Am Geriatr Soc. 2017;65(5):980-8.
57. Ware JE, Jr., Sherbourne CD. The MOS 36-item short-form health survey (SF-36). I. Conceptual framework and item selection. Medical care. 1992;30(6):473-83.
58. Ross TP. The reliability of cluster and switch scores for the Controlled Oral Word Association Test. Archives of clinical neuropsychology : the official journal of the National Academy of Neuropsychologists. 2003;18(2):153-64.
59. Koss E, Ober BA, Delis DC, Friedland RP. The Stroop color-word test: indicator of dementia severity. The International journal of neuroscience. 1984;24(1):53-61.
60. Shapirol AM, Benedict RHB, Schretlen D, Brandt J. Construct and Concurrent Validity of the Hopkins Verbal Learning Test-Revised. The Clinical Neuropsychologist. 1999;13(3):348-58.
61. Smith A. Symbol Digit Modalities Test (SDMT). Manual (Revised). Western Psychological Services, Los Angeles, 1982.
62. Morris JC, Heyman A, Mohs RC, Hughes JP, van Belle G, Fillenbaum G, et al. The Consortium to Establish a Registry for Alzheimer's Disease (CERAD). Part I. Clinical and neuropsychological assessment of Alzheimer's disease. Neurology. 1989;39(9):1159-65.
63. Lezak MD. Neuropsychological assessment, 3rd ed. New York: Oxford University Press. 1995.
64. Mitrushina M, Boone KB, Razani J, D'Elia LF. Handbook of Normative Data for Neuropsychological Assessment. 2 edn. ed: Oxford University Press Inc.; 2005.
65. Svarstad BL, Chewning BA, Sleath BL, Claesson C. The Brief Medication Questionnaire: a tool for screening patient adherence and barriers to adherence. Patient education and counseling. 1999;37(2):113-24.
66. Morisky DE, Green LW, Levine DM. Concurrent and predictive validity of a self-reported measure of medication adherence. Medical care. 1986;24(1):67-74.
67. Langa KM, Larson EB, Crimmins EM, Faul JD, Levine DA, Kabeto MU, et al. A Comparison of the Prevalence of Dementia in the United States in 2000 and 2012. JAMA internal medicine. 2017;177(1):51-8.
68. Ahmadi-Abhari S, Guzman-Castillo M, Bandosz P, Shipley MJ, Muniz-Terrera G, Singh-Manoux A, et al. Temporal trend in dementia incidence since 2002 and projections for prevalence in England and Wales to 2040: modelling study. Bmj. 2017;358:j2856.
69. Kawas CH, Katzman R. Epidemiology of Dementia and Alzheimer Disease. . Alzheimer Disease. 2nd Ed ed. Philadelphia:: Lippincott Williams & Wilkins; 1999. p. 95-116.
