# Supplementary material for: Statins for extension of disability-free survival and primary prevention of cardiovascular events among older people: protocol for a randomised controlled trial in primary care (STAREE trial)
Source: BMJ Open. 2023 Apr 3;13(4):e069915. doi: 10.1136/bmjopen-2022-069915 (PMC10083753; doi:10.1136/bmjopen-2022-069915)
Supplement: Supplementary data [file bmjopen-2022-069915supp003.pdf]

# A randomised clinical trial of STAtin therapy for Reducing Events in the Elderly (STAREE)

**PROTOCOL**

**Version 3.1**

**APPENDICES 1-11**

## Appendix 1: STAREE hub locations (update July 2019)

| Site location | STATE | HREC | Start Date | Office location |
|---|---|---|---|---|
| Melbourne and surrounds | VIC | MUHREC | 26/08/2014 | Monash University, 553 St Kilda Rd, Melbourne |
| Geelong | VIC | MUHREC | 05/09/2016 | Monash University, 553 St Kilda Rd, Melbourne |
| Mornington | VIC | MUHREC | 14/09/2016 | Monash University, 553 St Kilda Rd, Melbourne |
| Mildura | VIC | MUHREC | 16/01/2017 | School of Rural Health, Monash, Mildura (closed 2018) |
| Bendigo | VIC | MUHREC | 20/03/2017 | School of Rural Health, Monash, Bendigo |
| Gippsland | VIC | MUHREC | 20/03/2017 | Monash University, 553 St Kilda Rd, Melbourne |
| East Gippsland | VIC | MUHREC | 1/4/2019 | Monash University, 553 St Kilda Rd, Melbourne |
| Warrnambool | VIC | MUHREC | 26/04/2018 | Monash University, 553 St Kilda Rd, Melbourne |
| Albury/Wodonga | VIC | MUHREC | 19/04/2018 | Monash University, 553 St Kilda Rd, Melbourne |
| Hobart | TAS | HREC (Tasmania) network | 01/06/2015 | Menzies Institute for Medical research, University of Tasmania, Hobart |
| Burnie | TAS | HREC (Tasmania) network, | 01/01/2017 | Menzies Institute for Medical research, University of Tasmania, Launceston |
| Launceston | TAS | HREC (Tasmania) network | 01/01/2017 | Menzies Institute for Medical research, University of Tasmania, Launceston |
| Perth and regional WA | WA | HREC (Curtin), | 16/06/2015 | Curtin University, Perth |
| Newcastle | NSW | HREC (Uni Newcastle) | 11/09/2017 | University of Newcastle, Newcastle |
| Tamworth | NSW | HREC (Uni Newcastle) | 28/05/2018 | University of Newcastle, Newcastle |
| Port Macquarie | NSW | HREC (Uni Newcastle) | 22/07/2019 | University of Newcastle, Newcastle |
| Sydney | NSW | HREC (Uni Newcastle) | 21/05/2018 | University of Newcastle, Newcastle |
| Brisbane | Qld | MUHREC | 17/07/2017 | Corporate House, Greenslopes, Brisbane |
| Sunshine coast/Hervey Bay | Qld | MUHREC | 17/07/2017 | Corporate House, Greenslopes, Brisbane |
| Gold Coast | Qld | MUHREC | 17/07/2017 | Corporate House, Greenslopes, Brisbane |
| Toowoomba | Qld | MUHREC | 17/07/2017 | Corporate House, Greenslopes, Brisbane |
| Adelaide | SA | MUHREC | 24/04/2018 | Victoria Park, Dulwich, Adelaide |

## Appendix 2: Brief definitions of STAREE endpoints

| | |
|---|---|
| **PRIMARY ENDPOINTS** | |
| Death, Dementia and Disability | Survival free of dementia or persistent physical disability (as derived from the endpoints of all-cause mortality, dementia and persistent physical disability) |
| Major Adverse Cardiovascular Event (MACE) | Major Adverse Cardiac Event (MACE); cardiovascular death or acute and non-fatal myocardial infarction (MI) or stroke. |
| **SECONDARY ENDPOINTS** | |
| All-cause mortality | Any cause for death; will be classified based on primary cause such as stroke, MI, heart failure, cancer or other.<br>(All sudden deaths are presumed to be caused by a major cardiovascular event unless confirmed otherwise). |
| Cardiovascular death | Death resulting from a cardiovascular event (i.e. MI, stroke, heart failure). |
| Myocardial Infarction | Non-fatal: Evidence of myocardial necrosis in a clinical setting consistent with myocardial ischemia<br>Fatal: Death resulting from MI |
| Stroke | Fatal: Death resulting from stroke<br>Non-fatal: Acute episode of focal or global neurological dysfunction lasting more than 24 hours (unless interrupted by surgery or death) with no apparent cause other than of vascular origin. Imaging is required for classification.<br>OR |

| | |
|---|---|
| | Acute episode of focal or global neurological dysfunction lasting less than 24 hours and with no apparent cause other than of vascular origin AND imaging supporting stroke diagnosis. |
| Persistent Physical Disability | Persistent disability or loss of physical function as reflected on any one of the Life Ability ADL items (Q9-14) as either “a lot of difficulty” or “unable to do this activity” and/or “Yes” to receiving help, across 2 or more records (> 6 months duration).<br><br>OR<br><br>If it is not possible to obtain a Life Ability ADL assessment, relevant clinical records such as notification of admission to aged care or community assessment. |
| Dementia | Criteria-based: Acquired impairment in cognitive function that is significant (≥2 SD decline below population norms) and interferes with independence in everyday activities. [1]<br><br>Record-based: Credible diagnosis of dementia on medical record, without any supporting cognitive data available. |
| Other Cognitive Impairment | All other instances of cognitive impairment (not meeting criteria for “Dementia” above). |
| Approval for permanent residential care | Approval for permanent residential care (based on reporting by an Aged Care Assessment Team). |
| Diagnosis requiring hospitalisation | Hospitalisation extending more than 24 hours or across a minimum of two calendar days. Excludes elective procedures. |
| Heart Failure | A heart failure event, treated in the emergency room or being admitted to hospital and defined as including at least one criterion for heart failure symptoms, at least two for heart failure signs, and imaging or biomarker evidence. |
| Atrial Fibrillation | Verified: Any ECG rhythm strip showing AFib verified by a medical professional |

| | |
|---|---|
| | OR<br><br>Medical professional diagnosed AFib<br><br>OR<br><br>Procedures undertaken for AFib (e.g. cardio version, ablation)<br><br>OR<br><br>Possible: Irregular heart rhythm during heart rate measurement (e.g. irregular pulse on BP measure )<br><br>AND<br><br>Taking medication used to manage AF (oral anticoagulant agents without other indication (ADD ATC CODE), cardiac glycosides (ATC-Code C01A), antiarrhythmics, Class I and III (ATC-Code C01B)) |
| Hospitalisation for Arterial Revascularisation Procedures | Treatment or surgical intervention to relieve symptoms associated with coronary, arterial disease. Coronary, cerebral or peripheral revascularisation procedures, including percutaneous coronary intervention (PCI), coronary artery bypass graft surgery(CABG), carotid endarterectomy or carotid bypass, other arterial surgery (with or without stenting), other arterial surgery (aortic aneurysm repair, aortobifemoral bypass, femoropopliteal bypass, femoral-tibial bypass and cerebral aneurysm repair) |
| Fatal and non-fatal cancer | Malignant neoplasm confirmed by histopathology or imaging (non-melanoma skin cancers excluded). |
| Quality of life | Determined by the score (0-100) in each of the 8 domains of the SF-36; physical functioning, role limitations due to physical problems, bodily pain, general health perceptions, vitality, social functioning, role-limitations due to emotional problems, and mental health [56]. Based on population norms, a score of 50 is average, 10 point variation is one standard deviation (15 points = 1.5SD). Summary score collapses domains into two scores – age bracket specific |
| Cost-effectiveness of statin | To be derived from health economic analysis |

| **TERTIARY ENDPOINTS** | |
|---|---|
| Frailty Phenotype | Frailty phenotype will be measured using an approach based on the simplified Women's Health Initiative.[2] |
| **ADVERSE EVENTS OF INTEREST** | **BRIEF DEFINITION** |
| New diabetes | Diabetes as diagnosed on the basis of:<br>1) an annual HbA1c or fasting plasma glucose test using the WHO criteria (a HbA1c value of 6.5% or higher or a fasting plasma glucose of 7.0 mmol/L or higher, with confirmation by a second test within 4 weeks; or<br>2) community-based testing during usual clinical care (GP or other clinicians e.g. hospitalisation with a hyperglycaemic crisis or classic symptoms of hyperglycaemia and a random plasma glucose test of 11.1 mmol/L and/or commencement of glucose lowering medication for glucose control). |
| Myopathy | Participant description of any muscle symptoms including type, duration and effect of exercise. |
| Liver impairment | Elevated ALT and/or AST across two or more tests (elevations greater than 3x ULN and elevated bilirubin (2x ULN)) and no other demonstrable cause |

1. American Psychiatric Association. (2022). Neurocognitive Disorders. In *Diagnostic and statistical manual of mental disorders* (5th ed., text rev.).

2. Zaslavsky, Oleg, et al. "Comparison of the simplified sWHI and the standard CHS frailty phenotypes for prediction of mortality, incident falls, and hip fractures in older women." *Journals of Gerontology Series A: Biomedical Sciences and Medical Sciences* 72.10 (2017): 1394-1400.

For further information see also supplement document *Endpoint Definitions*

## Appendix 3 : Cytochrome P4503 A4 inhibitors clinical recommendations

(updated November 2020)

| **CYP450 3A4 Inhibitors** | | | | |
|---|---|---|---|---|
| **Brand Name** | **Generic Name** | **Indication** | **Drug Class** | **Study Recommendations** |
| Amiodarone<br>Aratac<br>Cardinorm<br>Cordarone<br>Rithmik | Amiodarone | Angina/Arrhythmia | Antiarrhythmic agent | **Limit study medication to one tablet (20 mg) for the duration of amiodarone therapy.** |
| Magicul<br>Tagamet | Cimetidine | Gastroesophageal reflux disease | Histamine receptor antagonist | **EXCLUDE/ withhold study medication for the duration of cimetidine therapy** |
| Cyclosporin<br>Neoral | Cyclosporin | Transplant rejection, rheumatoid arthritis | Immunosuppresant | **EXCLUDE/ withhold study medication for the duration of cyclosporin therapy** |
| Azol | Danazol | Endometriosis | Gonadotropin Inhibitor | **EXCLUDE/ withhold study medication for the duration of danazol therapy** |
| Cardizem<br>Coras<br>Diltahexal<br>Diltiazem<br>Dilzem<br>Vasocardol | Diltiazem | Angina/Hypertension | $Ca^+$ Channel Blocker | **Limit study medication to one tablet (20 mg) for the duration of diltiazem therapy.** |
| Telzir | Fosamprenavir | HIV | Protease Inhibitor | **EXCLUDE** |
| Kaletra | Lopinavir/ Ritonavir | HIV/AIDS | Protease Inhibitor | **EXCLUDE** |
| Anpec<br>Cordilox<br>Isoptin<br>Tarka<br>Veracaps | Verapamil | Angina/Hypertension/Arrhythmia | Ca+ Channel Blocker | **Limit study medication to one tablet (20 mg) for the duration of verapamil therapy.** |
| **CYP450 3A4 Inhibitors** | | | | |

| Brand Name | Generic Name | Indication | Drug Class | Study Recommendations |
|---|---|---|---|---|
| Norvir | Ritonavir | HIV/AIDS | Protease inhibitor and potent CYP3A4 inhibitor | **EXCLUDE** |
| Included in: Prezcobix Evotaz Stribild Genvoya Tybost | Cobicistat | HIV/AIDS | Potent CYP3A4 inhibitor | **EXCLUDE** |
| Maviret | Glecaprevir/ Pibrentasvir | Hepatitis C | Protease inhibitor | **EXCLUDE/ withhold study drug for the duration of protease inhibitor therapy (usually 12 weeks)** |
| Vosevi | Sofosbuvir/ Velpatasvir/ Voxilaprevir | Hepatitis C | Protease inhibitor | **EXCLUDE/ withhold study drug for the duration of protease inhibitor therapy (usually 12 weeks)** |
| Prevymis | Letermovir | Cytomegalovirus (CMV) prevention | CMV DNA terminase complex inhibitor | **EXCLUDE/ withhold study drug for the duration of letermovir therapy** |
| **Antibiotics and Antifungals for Long Term Oral Use Only** | | | | |
| **Brand Name** | **Generic Name** | **Indication** | **Drug Class** | **Study Recommendations** |
| Clarac Clarihexal Clarithro Clarithromycin Kalixocin Klacid Nexium Hp7 Combination pack | Clarithromycin | Bacterial infection | Macrolide antibiotic | **Limit study medication to one tablet (20 mg) for the duration of clarithromycin therapy.** |
| EES E-mycin Eryc Erythromycin | Erythromycin | Bacterial infection | Macrolide antibiotic | **Limit study medication to one tablet (20 mg) for the duration of erythromycin therapy.** |

| | | | | |
|---|---|---|---|---|
| Diflucan Fluconazole | Fluconazole | Fungal infection | Antifungal | **Limit study medication to one tablet (20 mg) for the duration of fluconazole therapy.** |
| Lozanoc Sporanox | Itraconazole | Fungal infection | Antifungal | **EXCLUDE/ withhold study medication for the duration of itraconazole therapy** |
| Nizora | Ketoconazole | Fungal infection | Antifungal | **EXCLUDE/ withhold study medication for the duration of ketoconazole therapy** |
| Fucidin | Fusidic acid or sodium fusidate | Bacterial infection | Antibiotic | **EXCLUDE/ withhold study medication for the duration of and one week after completion of fusidic acid therapy** |

1. www.drugbank.ca
2. www.medicines.org.uk
3. www.drugs.com/pro/atorvastatin.html
4. www.medsafe.govt.nz
5. Stockley's Drug Interactions

## Appendix 4: Medication compliance questions

1. Have you been taking study medication? Yes / No
2. If Yes, how may study tablets do you take each day? 1 tablet / 2 tablets / other___
3. In an average week, how many days do you take study medication? _____ days
4. Do you ever miss taking your study medications? Yes / No
5. Comments (i.e. reasons for occasionally missing or altering dose) ________

OR, if they answer NO to question 1, then:

2. What reason(s) did you stop taking study medication? ______________
3. What date did you stop taking study medication?
4. Who made this decision? GP or PCP / Self / Other

## Appendix 5: Reporting of AEs and SAEs

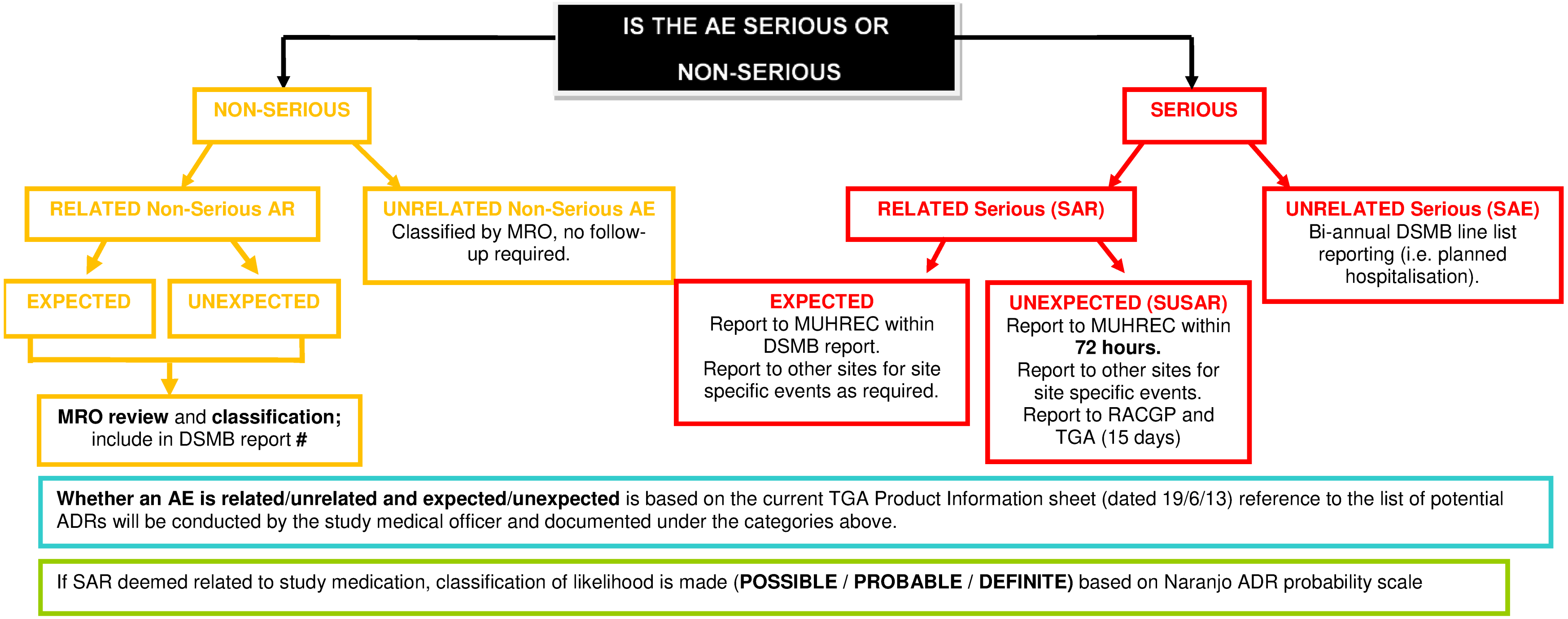

| | |
|---|---|
| **AE** Adverse Event | Any unfavourable medical occurrence |
| **SAE** Serious Adverse Event | An event that:<br>❖ requires hospitalisation/prolongation of existing hospitalisation<br>❖ results in death<br>❖ is life threatening<br>❖ results in persistent or significant disability/incapacity<br>❖ is a congenital anomaly/birth defect; or<br>❖ is a medically important event or reaction |
| **SAR** Serious Adverse Drug Reaction | A SAE that is deemed related to study medication (note serious ADR also used; also non-serious ADR) |
| **SUSAR** Serious, Unexpected, Suspected Adverse Reaction | An **unexpected** SAE where there is **some degree of probability** that the event is related to the study medication. |
| **PI** Principal Investigator | Principal Investigator or a co-investigator delegated this responsibility |
| **Comment from the PI** | A statement about the significance of the information, the possible impact on study participants, and action taken or recommended |

***DSMB report: submitted six-monthly: includes line list of non-serious AEs of interest # (muscle symptoms, changes to memory) and summary of related SAEs, both expected and unexpected; the former based on the current Product Information sheet; Reports from DSMB to be forwarded to all committees & ADR select section to TGA***

## Appendix 6: Assessment tools

| Outcome Measure | Tool (abbreviation) | References |
|---|---|---|
| Global cognitive function | Modified Mini-Mental State Examination (3MS) | Tombaugh, T.N. et al. (2005). Test-retest reliable coefficients and 5-year change scores for the MMSE and 3MS. *Archives of Clinical Neuropsychology*, 20, 485-503<br><br>Ryan, J., et al. (2019). Normative performance of healthy older individuals on the Modified Mini-Mental State (3MS) examination according to ethno-racial group, gender, age, and education level. *The Clinical Neuropsychologist*, 33(4), 779-797. |
| Verbal episodic memory | Hopkins Verbal Learning Test – Revised (HVLT-R) | Shapirol, A.M., et al. (1999). Construct and Concurrent Validity of the Hopkins Verbal Learning Test-Revised. *The Clinical Neuropsychologist,* 13(3), 348-358<br><br>Ryan, J., et al. (2021). Normative performance of older individuals on the Hopkins Verbal Learning Test-Revised (HVLT-R) according to ethno-racial group, gender, age and education level. *The Clinical Neuropsychologist*, 35(6), 1174-1190 |
| Verbal fluency, language | Controlled Oral Word Association Test (COWAT) | Ross, T.P., 2003. The reliability of cluster and switch scores for the Controlled Oral Word Association Test. *Archives of Clinical Neuropsychology*, 18(2), 153-164<br><br>Ravdin, L.D., et al. (2003). Letter and semantic fluency in older adults: effects of mild depressive symptoms and age-stratified normative data. *The Clinical Neuropsychologist*, 17(2), 195-202 |
| Visual agnosia | Lurian Overlapping Figures (Lurian) | Luria, A.R. (1966). Higher Cortical Functions in Man. New York, NY: Basic Books.<br><br>Reid, W., et al. (1996). Age at onset and pattern of neuropsychological impairment in mild early-stage Alzheimer disease: a study of a community-based population. *Archives of Neurology*, 53(10), 1056-1061 |
| Executive function | Victoria Stroop Colour-Word Test (Stroop) | Koss, E., et al. (1984). The Stroop color-word: Indicator of dementia severity. *International Journal of* Neuroscience, 24(1), 53-61<br><br>Troyer, A.K., et al. (2006). Aging and response inhibition: Normative data for the Victoria Stroop Test. *Aging, Neuropsychology, and Cognition*, 13(1), 20-25 |

| | | |
|---|---|---|
| Executive function | Trail Making Test (TMT) | Corrigan, J.D., & Hinkeldey, M.S. (1987). Relationships between Parts A and B of the Trail Making Test. *Journal of Clinical Psychology,* 43, 402-409<br><br>Mitrushina, M., et al. (2005). Handbook of Normative Data for Neuropsychological Assessment, Appendix 4M, Oxford, UK: Oxford University Press |
| Processing speed | Symbol Digit Modalities Test (SDMT) | Smith, A. Symbol Digit Modalities Test (SDMT). Manual (Revised) (1982). Los Angeles, CA: Western Psychological Services.<br><br>Kiely, K.M., et al. (2014). Butterworth, P., Watson, N. & Wooden, M. The Symbol Digit Modalities Test: Normative data from a large nationally representative sample of Australians. *Archives of Clinical Neuropsychology,* 29, 767-775 |
| Constructional praxis | Consortium to Establish a Registry for Alzheimer's Disease (CERAD) Neuropsychological Battery; Constructional Praxis Subscale (constructional praxis) | Morris, J.C. et al. (1989). The Consortium to Establish a Registry for Alzheimer's Disease (CERAD). Part I. Clinical and neuropsychological assessment of Alzheimer's disease. *Neurology,* 39, 1159-1165.<br><br>Welsh, K.A., et al. (1994). The Consortium to Establish a Registry for Alzheimer's Disease (CERAD). Part V. A normative study of the neuropsychological battery. *Neurology*, 44, 609-614. |

# PARTICIPANT CONSENT FORM

*MONASH UNIVERSITY*

## Appendix 7: STAREE participant information sheet/consent form



Zoungas S, et al. BMJ Open 2023; 13:e069915. doi: 10.1136/bmjopen-2022-069915

# PARTICIPANT CONSENT FORM

*MONASH UNIVERSITY*



Zoungas S, *et al. BMJ Open* 2023; 13:e069915. doi: 10.1136/bmjopen-2022-069915

# PARTICIPANT CONSENT FORM

*MONASH UNIVERSITY*

# PARTICIPANT INFORMATION SHEET/CONSENT FORM

*MONASH UNIVERSITY*

**Full Project Title:** A randomised clinical trial of STAtin therapy for Reducing Events in the Elderly (STAREE)

This participant information and consent form is 8 pages long. Please make sure you have all the pages.

This document tells you about the research project. It explains the tests and treatments involved. Knowing what is involved will help you decide if you want to take part.

Please read this information carefully. Ask questions about anything that you do not understand or want to know more about. Before deciding whether or not to take part, you may want to talk about it with a relative, friend or your local doctor.

If you decide to take part in the research project, you will be asked to sign the consent section. By signing it you are telling us that you:

- Have read the participant information sheet and consent form;
- Understand what you have read;
- Agree to take part in the research project;
- Agree to take the study medication as instructed for an average of 5 years;
- Agree to undergo the study assessments as described in this document;
- Agree to attend clinic visits annually;
- Agree to your medical records being accessed; and
- Agree to having your data accessed via data linkage (bringing together information from different sources such as registries, hospitals etc.).

You will be given a copy of this Participation Information and Consent Form to keep.

For any queries, information on the project or to speak to the research team please call **1800 770 664**.

### WHY ARE YOU BEING GIVEN THIS PARTICIPANT INFORMATION AND CONSENT FORM?

We would like to invite you to participate in this research project as you are aged 70 or older and do not have current serious disease or disability.

### WHO IS RUNNING THIS STUDY?

The study is run by the School of Public Health and Preventive Medicine, Monash University. Your general practitioner (GP) may be a co-investigator and may receive up to $100 per patient for the use of their practice facilities.

### WHAT IS THE STUDY ABOUT?

Statins (HMG-CoA reductase inhibitors) are a class of drug that block the action of a chemical (HMG-CoA reductase) in the liver that is needed to make cholesterol. This



Zoungas S, *et al. BMJ Open* 2023; 13:e069915. doi: 10.1136/bmjopen-2022-069915

# PARTICIPANT CONSENT FORM

*MONASH UNIVERSITY*

action lowers cholesterol and lipid levels in the blood. The health benefits of statins may include reducing the number of new cases of heart disease, stroke and dementia. However, there are side-effects from statins that are still being debated. We are investigating if the benefits of statins outweigh the risks in healthy people aged 70 and over. We aim to determine if taking statins prevents a decline in the physical and mental abilities associated with ageing which may lead to long term residential care and a loss of independence.

STAREE will be conducted in general practices across Australia and aims to recruit at least 10,000 participants nationally. Half of the participants will receive statin therapy and half will receive a placebo tablet (tablet with inactive ingredients). We will then observe the 2 groups over an average 5 year period to see what differences there are for levels of physical disability, death, heart attacks, diabetes, dementia and general quality of life. We will collect this data from you, your GP's records, and any hospital or specialist you visit.

You will also be asked to fill out a separate consent form authorising access to your complete Medicare, Pharmaceutical Benefits Scheme (PBS), Repatriation Pharmaceutical Benefits Scheme (RPBS) data and various health data sets. Medicare collects information on your doctor visits and the associated costs, while the PBS/RPBS collects information on the prescription medications you have filled at pharmacies. This consent form is sent securely to the Department of Human Services which holds this information confidentially. We also ask for your consent to access other health data information such as hospital discharge summaries and national registry health information (i.e. National Death Index).

Providing us with this information helps us to assess your health status throughout the study.

### WHAT ARE WE ASKING YOU TO DO?

**Baseline Visit 1**: If you agree to participate, you will be asked to give your consent by signing this form. After you give your consent to enter this study, research staff will briefly look at your medical records to ensure there are no underlying medical conditions that would make you ineligible for the study, and to check any pathology results from the previous 6 months to ensure no tests are repeated unnecessarily. We will then measure your blood pressure, heart rate and ask you some questions about your general health and medical history, your family medical history, current medications, and your daily activities. We will ask you to complete other questionnaires that will assess your mood and involve some thinking tasks. You will have a brief physical examination including measuring your weight, height, waist and hip circumference.

We will then ask you to take a placebo tablet of study medication once daily for four weeks. This will help get you in the routine of taking a tablet every day.

The research staff will also ask you to attend a local pathology service for a fasting blood test and a urine test. Approximately 12 ml (about 2 teaspoons) of blood will be taken and this sample will be used to check your cholesterol, liver and kidney function and sugar levels. Approximately 20 ml of urine will also be collected. This will provide information

# PARTICIPANT CONSENT FORM

*MONASH UNIVERSITY*

about your kidney function and cardiovascular health.

Based on these test results, the physical examination and your medical and medication history, we will ask you to see your GP to discuss if it is appropriate for you to continue in the study and attend baseline visit 2.

**Baseline Visit 2**: At the second visit, if you have taken your study medication, and you satisfy all other eligibility criteria, you will be randomly assigned to take two tablets of either statin (atorvastatin 40 mg) or placebo (a dummy tablet), at the same time every day with a drink of water for an average of 5 years. You will need to commit to taking the study medication regularly as well as attend all the study visits.

The medication you receive will be determined by a computer program. Neither you, research staff, nor your GP will know if you are taking the active (statin) or placebo medication. You will have equal chance of receiving either statin or placebo.

In the event of an emergency, any doctor can find out what treatment you are taking if they need to know.

During this visit, you will be asked to complete tasks that measure thinking and memory function and a questionnaire on your health and physical ability.

**Annual clinic visit:** Each year, you will be asked to attend a study visit or a phone call where staff will conduct similar activities as in your baseline visits. You will be asked to attend this visit, even if you stop taking the study medication.

**6 monthly phone call**: Every six months the research staff will phone you and ask you about your recent medical history, medications and how you are going on the trial.

### CAN I LEAVE THE STUDY?

Participation in any research project is voluntary. If you do not wish to take part, you do not have to. If you decide to take part and later change your mind, you can change your level of involvement with the project at any stage.

While full participation in the study means that you are taking the study medication and having study visits and phone calls, there are other levels of participation that you may consider. Stopping or discontinuing study medication (even for a short time period) does not mean you have to stop your involvement in the trial.

The others levels of involvement are:

- Stopping your study medication but still attending study visits and receiving phone calls
- Stopping your study medication and also stopping study visits and phone calls
- Stopping your study medication, stopping study visits and phone calls and stopping collection of data from your medical records

Please note, if you decide to not continue your participation in the study, the data that has been collected up to this point will remain part of the study data. It is important that this data remains in the study because if it is excluded the safety of the research project may be compromised. Retention of this data is also important to ensure appropriate reporting to the study Data and Safety Monitoring Board.



Zoungas S, *et al. BMJ Open* 2023; 13:e069915. doi: 10.1136/bmjopen-2022-069915

# PARTICIPANT CONSENT FORM

*MONASH UNIVERSITY*

If you do decide to stop your involvement from the study, we may ask you about your reasons. This information is important for the conduct of future trials.

Regardless of your level of participation or if you stop your involvement completely from the study, it will not affect your routine treatment, your relationship with your GP or your relationship with Monash University.

**WHAT ARE THE POSSIBLE BENEFITS OF TAKING PART?**

We cannot guarantee that you will receive any health benefits from this research. However, some participants may experience health benefits from being placed on atorvastatin. These benefits may include the following:

- Reductions in the levels of cholesterol in the blood,
- Improved health of blood vessels throughout the body,
- A reduced risk of cardiovascular disease (e.g.) heart attack or stroke).

In addition, some scientific studies have suggested that statins may be associated with reduced risk of dementia, depression, and cancer. However, these effects are less certain.

If the study shows that there are net health benefits from statin therapy, it may enable this treatment to be available to more people in the future. We will also inform your doctor of any abnormal findings from your measurements, and memory and thinking tasks, so that they can discuss this with you.

**WHAT ARE THE POSSIBLE RISKS AND DISADVANTAGES OF TAKING PART?**

Atorvastatin, the statin used in this study, is an established safe and well-tolerated medication to lower cholesterol. However, like all medications, atorvastatin can cause side-effects. These side-effects do not affect everyone. You may experience no side effects. If you do experience side effects from the study medication they may be mild, moderate or severe. Many side effects are temporary or reversible, however, some side effects can be serious, long lasting or permanent. If this occurs, your doctor may ask you to stop taking the medication.

You could experience side effects that researchers do not expect or do not know about and these side effects could be serious.

Tell your doctor immediately about any new or unusual symptoms that you experience. Your doctor will monitor any side effects that do occur and will discuss the best way of managing them with you.

The reported side effects of atorvastatin:

- Common side effects (occur in 1-10% of patients): headache, muscle pain, constipation, feeling sick and increase in blood sugar levels.
- Uncommon side effects (0.1-1%): skin rash, itching and hives.
- Rare side effects (0.01-0.1%): hypersensitivity (allergic) reactions and severe muscle disorders (myopathy).

You may also refer to the Product Information Sheet provided to you at your first study visit.



Zoungas S, *et al. BMJ Open* 2023; 13:e069915. doi: 10.1136/bmjopen-2022-069915

# PARTICIPANT CONSENT FORM

*MONASH UNIVERSITY*

You should not drink more than one or two small glasses of grapefruit juice each day as large quantities of grapefruit juice can change the effects of atorvastatin. You should also avoid consuming excessive amounts of alcohol on a regular basis while taking the study medication.

If you suffer any injuries or complications as a result of this research project, you should contact the study team as soon as possible so they can assist with arranging appropriate medical treatment. If you are eligible for Medicare, you can receive any medical treatment required, free of charge, as a public patient in any Australian public hospital.

**WHAT IF NEW INFORMATION ARISES DURING THE RESEARCH PROJECT?**

Sometimes during the course of a research project, new information becomes available about the treatment being studied. If this happens, your doctor will tell you about it and discuss with you whether you want to continue in the research project. If you decide to withdraw, your doctor will continue with your regular health care. If you decide to continue in the research project you will be asked to sign an updated consent form.

Also, on receiving new information, your doctor may consider it to be in your best interest to withdraw you from the research project. If this happens they will explain the reasons and arrange for your regular health care to continue.

**WHAT WILL HAPPEN TO INFORMATION ABOUT ME?**

Any information collected in this study will not be published in any manner that could identify you as an individual, during or after the conclusion of this project. We will only publish group data. Your personal and health information which we will collect as part of this study is reported on special forms by the research staff. Your name is not recorded on these forms. You are only identified by your initials and a study-specific number assigned by research staff.

By signing the attached consent form, you are agreeing to the release of your medical records held by your doctor or hospital. This information will be collected, stored and analysed only for the purposes of this study, and will be limited to the medical details related to this study. Any information collected from your clinic about you will be overseen by the clinic GPs.

Medical tests and results collected as part of the study may reveal an underlying condition. Any results outside the normal range (such as elevated blood pressure or abnormal pathology) will be communicated to your GP.

Final results of the project and the arm of the trial you were part of, will be communicated to you at the conclusion of the trial.

Monash University implements a defence in-depth approach (multiple layers of security) to information security and employs a multitude of controls to protect our infrastructure and data. These controls are regularly audited to ensure they meet global best practices and are aligned with ISO 27001 security practices. Data collected as part of the STAREE clinical trial will be stored on University-managed, secure and resilient infrastructure located in Australia that complies with all applicable data protection and privacy obligations. Some specialist data collected



Zoungas S, *et al. BMJ Open* 2023; 13:e069915. doi: 10.1136/bmjopen-2022-069915

# PARTICIPANT CONSENT FORM

*MONASH UNIVERSITY*

as part of the STAREE clinical trial may be entered into applications that are operated by external third parties. This will only be permitted where all applicable data protection and privacy obligations are in place with those external third parties.

All information collected for this project will be retained for a period of no less than 15 years following the completion of the project. Hardcopy information will be shredded and destroyed and electronic data will be deleted from the secure STAREE database after a period of no less than 15 years after the completion of the project, in accordance with the Monash University Human Research Ethics Committee recommendations for drug trials and Good Clinical Practice Guidelines.

**WHO IS FUNDING THE RESEARCH?**

The main funding body of STAREE is the National Health and Medical Research Council of Australia.

**WHO HAS REVIEWED THE RESEARCH PROJECT?**

All research in Australia involving humans is reviewed by an independent group of people called a Human Research Ethics Committee (HREC). The ethical aspects of this project have been approved by the Monash University Human Research Ethics Committee (MUHREC) and The Royal Australian College of General Practitioners National Research and Evaluation Ethics Committee (NREEC).

This project will be carried out according to the *National Statement on Ethical Conduct in Human Research (2007)*. This statement has been developed to protect the interests of people who agree to participate in human research studies.

**FURTHER INFORMATION AND WHO TO CONTACT**

Please ask the research staff member if you have any further questions. You can also consult your own doctor if you have any concerns during the course of the research.

Specific enquiries related to this study can also be made to the following person at STAREE:

Name: Professor Sophia Zoungas
(Monash University):
Phone: +61 3 9903 0711
E-mail: sophia.zoungas@monash.edu

For matters relating to research at the site at which you are participating, or if you have any complaints about any aspect of the project, the way it is being conducted or any questions about being a research participant in general, you may contact the Executive Officer, Monash University Human Research Ethics (MUHREC) quoting project 21528:

Executive Officer
Monash University Human Research
Ethics Committee (MUHREC)
Room 111, Building 3e

Research Office
Monash University VIC 3800
Tel: +61 3 9905 2052
Email: muhrec@monash.edu
Fax: +61 3 9905 3831

# PARTICIPANT CONSENT FORM

*MONASH UNIVERSITY*



Zoungas S, *et al. BMJ Open* 2023; 13:e069915. doi: 10.1136/bmjopen-2022-069915

# PARTICIPANT CONSENT FORM

*MONASH UNIVERSITY*

# PARTICIPANT CONSENT FORM

*MONASH UNIVERSITY*

**Title** A randomised clinical trial of STAtin therapy for Reducing Events in the Elderly (STAREE)

## DECLARATION BY PARTICIPANT

I have read the Participation Information Sheet or someone has read it to me in a language that I understand.

I understand the purposes, procedures and risks of the research described in the project.

I understand I will be asked to take a study medication for the duration of the project, and be contacted on a regular basis by phone or in person.

I give permission for my doctors, other health professionals, hospitals or laboratories to release information to Monash University concerning my health and treatment for the purposes of this project. I understand that such information will remain confidential.

I give permission for information that I provide for this research project to be linked to various health datasets for the purposes of the project's outcomes and future research.

I have had an opportunity to ask questions and I am satisfied with the answer I have received.

I freely agree to participate in this research project as described and understand that I am free to change my level of participation or withdraw at any time during the study without affecting my future health care.

I understand that if I decide to discontinue the study medication, I may be asked to attend follow-up visits to allow collection of information regarding my health status.

I understand that if I choose to withdraw from the project that any data that has been collected prior to my withdrawal will be retained in the study database.

I understand I will be given a signed copy of this document to keep.

Name of participant (please print)______________________________________________

Signature_______________________________________Date____________________

## DECLARATION BY RESEARCHER

I have given a verbal explanation of the research project, its procedures and risks and I believe that the participant has understood that explanation.

Name of researcher (please print)______________________________________________

Signature_______________________________________Date____________________



Zoungas S, *et al. BMJ Open* 2023; 13:e069915. doi: 10.1136/bmjopen-2022-069915

## Appendix 8: List of relevant documents.

- DSMB Charter and Plan
- Endpoint Adjudication Committee Charter
- Monitoring plan
- Register of interests for STAREE Investigators and DSMB members
- STAREE HEART protocol



Zoungas S, *et al. BMJ Open* 2023; 13:e069915. doi: 10.1136/bmjopen-2022-069915

## Appendix 9: COVID pandemic response

**As a result of government and workplace restrictions due to the COVID-19 pandemic and to ensure safety of staff and participants, the following changes were made to the STAREE clinical trial following ethics approval:**

13th March 2020: Risk management plan formed and approved by STAREE Executive committee

16th March 2020: Invitation letters to participants suspended

20th March 2020: Face to face visits ceased. GP recruitment suspended.

23rd March 2020: Annual Visit 1 was modified to be conducted as a phone call visit

24th April 2020: Baseline Visit 2 was modified to be conducted as a phone call visit

18th May 2020: Annual Visits 2 and 4 were modified to be conducted as phone call visits

27th May 2020: Resumed sending invitation letters to participants. Baseline Consent visit (BV-C) instituted as a phone/zoom call.

July 2020: Physically distanced face to face BV1 visits (SD-BV1) commenced (phone visits offered as alternative if participant preference).

**Changes to the schedule:**

To provide a physically distanced or phone visit the following modifications were made to the visit schedule:

Physically distanced BV1 (SD-BV1): The consent procedure was moved to a separate phone/zoom visit called Baseline Consent (BV-C). Some sections of the usual Baseline 1 visit were moved to the Baseline 2 visit.

3MS: to be completed in full during in-person SD-BV1 or to a modified version used for phone based BV1s.

SD-BV2: All BV2 visits will be conducted by phone.

Annual Visits: The annual visit schedule has been modified so that all annual visits can be conducted over the phone during the COVID-19 pandemic. Face to face visits will resume in 2023.



Zoungas S, *et al. BMJ Open* 2023; 13:e069915. doi: 10.1136/bmjopen-2022-069915

# Appendix 10: STAREE MIND protocol

**STAREE-MIND Imaging Substudy**

PROTOCOL

Version 2.2

Protocol authors: Dr Andrea Curtis, Dr Simone Spark, Dr Ian Harding, Professor Sophia Zoungas

Affiliation: School of Public Health and Preventive Medicine, Monash University

**Contact for public enquiries**

Phone: 1800 770 664

Email: staree@monash.edu

Fax: +61 (3) 9903 0737

**Contact for scientific enquiries**

Professor Sophia Zoungas (Principal Investigator)

Sophia.zoungas@monash.edu

Phone: +61 (3) 9903 0711

Address: Level 5/99 Commercial Rd, Melbourne 3004

**Recruitment Status:** Recruiting

**Acronyms used in this protocol**:

ABS: Australian Bureau of Statistics

ADR: Adverse Drug Reaction

ADCS-IADL: Alzheimer’s Disease Cooperative Study Instrumental Activities of Daily Living

ADAS-Cog: The Alzheimer's Disease Assessment Scale - Cognition

AE: Adverse events

CAM: Confusion Assessment Method

CES-D-10: Centre for Epidemiologic Studies Depression Scale – 10 items

COWAT: Controlled Oral Word Association Test

CRF: Case Report Forms

DMC: Data Management Committee

DOHA: Department of Health and Ageing

DSMB: Data Safety and Monitoring Board

EAC: End-point Adjudication Committee

HVLT-R: Hopkins Verbal Learning Test – Revised

MI: Myocardial Infarction

MRI: Magnetic Resonance Imaging

PET: Positron Emission Tomography

SAE: Serious Adverse Events

SDMT: Symbol Digit Modalities Test

STAREE: STAtin therapy for Reducing Events in the Elderly

3MS: Modified Mini-Mental State test

Zoungas S, *et al. BMJ Open* 2023; 13:e069915. doi: 10.1136/bmjopen-2022-069915

# 1 Introduction

## 1.1 Background

### 6.1.1 *Rationale*

Statins—HMG-CoA reductase inhibitors—are a cornerstone treatment of cardiovascular disease. They decrease circulating low density lipoprotein (LDL) cholesterol levels. This effect underpins their ability to reduce the incidence of coronary heart disease and stroke events and possibly their long-term sequelae which include cardiac failure, disability, cognitive decline and dementia.

There are several different types of statins but the most commonly prescribed is atorvastatin.[1] Statins can be sub-grouped according to their hydrophilic or lipophilic nature. Atorvastatin, lovastatin, and simvastatin are lipophilic. Pravastatin, rosuvastatin, and fluvastatin are hydrophilic. Lipophilic statins are able to cross cell membranes including the blood brain barrier.[2] It has been proposed that lipophilic statins may be more effective in the prevention of cognitive decline and dementia.[3]

***Statins, lipids and dementia***

The main lipid in the brain is cholesterol. Scientific evidence suggests that a diet high in fat and cholesterol is associated with an increased risk of AD.[4] High levels of cholesterol inhibit the release of soluble amyloid precursor protein, leading to overproduction of Aβ protein.[5-7] In vivo models of rabbits fed cholesterol show accumulation of excess Aβ protein, which is cleared with return to a normal diet.[8] An increase in intracranial atherosclerosis has also been associated with an increased risk of developing dementia.[9] Statins may reduce risk of dementia through reductions in atheroma burden and in the accumulation of Aβ protein, the major constituent of the senile plaques that are a principal pathological feature of AD.[10]

***Statins, inflammation and dementia***

Substantial evidence implicates inflammatory processes in the development of vascular dementia and AD. Transgenic mouse AD models show increased inflammatory markers with onset of AD pathology.[11] IL-1β is overexpressed sixfold in AD patients' brains compared with controls, especially near amyloid plaques.[12] Increased inflammatory protein levels have been found in the brains and plasma of dementia patients.[13-15] In human studies, serum pro-inflammatory markers may predict the likelihood of dementia and cognitive decline. For example, elevated C-reactive protein (CRP) levels were related to a significantly increased risk for vascular dementia and AD, with or without cardiovascular disease, in the Honolulu Asia Aging Study.[15] The Rotterdam Study found a relationship between elevated levels of α1 antichymotrypsin, CRP, IL-6, soluble intercellular adhesion molecule, and soluble vascular cell adhesion protein 1, and an increased risk for vascular dementia and AD.[13] Meta-analysis showed significantly higher concentrations of the pro-inflammatory cytokines IL-6, TNF-α, IL-1β, IL-12, and IL-18 in the peripheral blood of AD subjects compared with controls.[16] Such data indicate that inflammation may be a key pathogenic accompaniment of dementia. Statins may reduce risk of dementia by inhibiting the pro-inflammatory response that exacerbates and drives the pathological processes leading to neuronal loss.



Zoungas S, *et al. BMJ Open* 2023; 13:e069915. doi: 10.1136/bmjopen-2022-069915

***Proposed mechanisms by which statins inhibit pathognomonic Alzheimer's disease***

In AD, Amyloid-β protein (Aβ) is cleaved from trans-membranous amyloid precursor protein (APP) and secreted extracellularly.[17] Aβ may aggregate to oligomers in the synaptic space and alter ion channels by oxidative stress, leading to an influx of excitatory toxic ions. With iron ($Fe^{2+}$) and copper ($Cu^{2+}$) ions, Aβ oligomers lead to the production of hydrogen peroxide, which directly damages lipid-rich cell membranes. Aβ is internalized after attachment to apolipoprotein E4 (ApoE4) lipoprotein receptors. Intracellularly, Aβ may aggregate to form neuritic plaques which may either activate microglia, leading to inflammation, or form highly active free oxygen radicals that facilitate nitric oxide synthase (NOS). Statins directly inhibit this detrimental cascade by blocking Aβ cleavage from APP, by reducing the cellular uptake of Aβ, in part by the suppression of ApoE secretion, and by inhibiting the expression of inducible NOS which, has anti-inflammatory effects and counteracts microglia activation.[17].

***Statins, endothelial nitric oxide synthase (eNOS) and vascular dementia***

Statins are also proposed to reduce the risk of vascular dementia through their regulation of nitric oxide production and eNOS.[18] eNOS activation exerts a protective effect in ischemic stroke patients, which is partly due to its vasodilatory effects resulting in increased cerebral blood flow.[18]

***Effects of statins on cognition and dementia.***

Most of the data on the effect of statins on cognitive function and development of dementia, both AD and vascular dementia, are from population based studies which suggest a reduction in dementia among statin users (Table 1). A new observational study of older US patients reported that AD risk was reduced by 12-15% amongst those with high statin exposure compared to those with low statin exposure.[24] Furthermore, re-analysis of patient level observational data has reported a 20% lower risk of AD in older statin users, and a slower decline in global cognitive scores in those with an ApoE4/ApoE4 genotype.[25] However, these observational studies are subject to numerous biases, foremost among them the inability to establish causal relationships; confounding by indication and unmeasured factors; self-report of statin use; and survival bias. Whilst such biases can be overcome with an appropriately designed RCT, previous RCTs examining the effect of statins on cognitive function and dementia have not reported significant effects. A 2015 meta-analysis investigating randomised evidence on the impact of statins on cognitive outcomes found no statistically significant effect on a crude overall measure of cognition.[26] However, these findings were limited by inclusion of trials that were too small, too short (with less than 1 year of follow up) and too early (<65 years, where the base rate of cognitive decline is very low and subtle effects may not be measureable) or late in the disease course (disease established and irreversible). Indeed, this meta-analysis could not address either long-term efficacy or safety for neurocognitive outcomes, particularly in older populations, because very few such trials exist.

***Adverse effects of statins on cognitive impairment.***

In 2012, the FDA investigated the US spontaneous reporting database (AERS), the published medical literature (case reports and observational studies), and RCTs with respect to the



Zoungas S, *et al. BMJ Open* 2023; 13:e069915. doi: 10.1136/bmjopen-2022-069915

effect of statin therapy on cognition.[27] AERS post-marketing reports described ill-defined memory loss with statin treatment, which was reversible on withdrawal. Time to onset of impairment was highly variable, ranging from 1 day to years after first statin exposure. The investigation did not reveal associations between cognitive impairment and specific statins or statin doses, specific age groups or concomitant medication use. Also, cognitive decline was not associated with fixed or progressive impairment. Nonetheless, in 2012 the FDA modified the statin labelling to include a 'black box' warning for a potential risk of adverse cognitive effects such as reversible memory loss and confusion.[27]

### 1.2 Summary of rationale

Atorvastatin and Rosuvastatin are the two drugs with the highest cost to the Australian government and patients, at greater than $1 billion per year.

Based on absolute cardiovascular risk, for which age is the dominant factor, statin use is expected to increase, especially among those >70 years.

Neurological disorders are the outstanding significant cause of non-fatal burden of disability in this country.[28] By 2023, this disease group will contribute almost half of Australia's disability burden.[28] One of the most pervasive and debilitating of these conditions is dementia (including vascular dementia and Alzheimer disease (AD)). By 2050, the number of Australians living with dementia will triple to around 900,000.[29]

Equipoise around the efficacy and safety of statins in the prevention of cognitive decline and dementia exists. Effects in older people are unknown.

If statins produce even a modest reduction or increase in cognitive decline and dementia, the impact across the whole population and on the cost of care for older people, will be considerable. Identifying such effects requires a very large RCT such as STAREE.

STAREE-MIND aims to add an additional suite of neuroimaging assessments in a subset of participants in the parent STAREE trial, making it possible to identify effects with potentially significant impact.



Zoungas S, et al. BMJ Open 2023; 13:e069915. doi: 10.1136/bmjopen-2022-069915

## 2 Study MIND Design

STAREE MIND is a substudy nested in the STAREE double blind randomised placebo-controlled trial. STAREE is investigating whether statins can prolong good health and maintain independence amongst older people and is enrolling men and women over 70 years of age who are free from cardiovascular disease, diabetes and dementia. The STAREE protocol is Version 2 dated October 2019.

STAREE MIND will recruit a subset of 50 to 500 STAREE participants before they are randomised to STAREE study drug. STAREE MIND will involve an additional suite of brain imaging in these participants. The imaging will take place at study entry and at 4 years follow-up.

## 3 Objectives

The aims are to conduct an imaging ancillary study in STAREE participants that will:

1. Determine the effect of statin treatment over a period of 4 years on longitudinal measures of brain health, including magnetic resonance imaging (MRI) of grey matter volume, white matter integrity and white matter hyperintensities, cerebral perfusion and metabolism, and iron loading.
2. Determine the relationship between imaging changes in the brain and incident cognitive decline and dementia.

## 4 Study Population

### 4.1 Inclusion criteria

- Men and women aged ≥70 years living independently in the Australian community who are participants in the STAREE RCT.
- Willing and able to provide informed consent and accept the STAREE-MIND study requirements, including attending the Monash Biomedical Imaging Facility (located at Blackburn Rd, Clayton, VIC) or the Herston Imaging Research Facility (located at the Royal Brisbane & Women's Hospital campus, Herston, QLD).

### 4.2 Participant recruitment

STAREE-MIND will recruit participants from among those enrolled in the STAREE RCT. Participants will be provided with the STAREE-MIND PICF and invited to participate in the substudy. Participants will be invited to provide informed consent by signing the PICF prior to any assessments being conducted. For participants who consent to STAREE-MIND, a booking for neuroimaging will be made by STAREE administration staff.

The initial neuroimaging assessment will occur prior to commencing STAREE study drug.

STAREE-MIND imaging procedures will be conducted at the Monash Biomedical Imaging Facility (located at 770 Blackburn Rd, Clayton) and the Herston Imaging Research Facility (located at the Royal Brisbane & Women's Hospital campus, Herston, QLD). Participation in STAREE-MIND will therefore be limited to STAREE participants from Greater Melbourne



Zoungas S, et al. BMJ Open 2023; 13:e069915. doi: 10.1136/bmjopen-2022-069915

and regional Victoria, or Greater Brisbane and regional Queensland, who are able to travel to one of these locations. Participants will be offered $20 to cover their time and costs incurred for transport to the respective facility.

### 4.3 Contingency plans for participant well-being

The procedures used in STAREE-MIND are not diagnostic. However, MRI scans may incidentally reveal an unexpected brain abnormality. All MRI scans are reviewed by a clinical radiologist, and any abnormality is reported. A clinical radiologist will provide a report on the abnormality, including a recommendation as to whether clinical follow-up is necessary.

Participants will be informed on the PICF that if an abnormality is identified, and determined to require clinical follow-up by the consulting radiologist, a report will be forwarded to the participant's doctor and the participant will be contacted by the research team to indicate that follow-up with their doctor is necessary. In line with duty of care requirements, participants who do not wish to be informed of an abnormality under any circumstances, regardless of severity, will not be enrolled in the study.

### 4.4 Withdrawal of participants from the STAREE-MIND study

Participants will be free to withdraw from the STAREE-MIND study at any time by notifying the STAREE research team. If participants withdraw from STAREE-MIND they will be requested to allow the retention of the personal and health information that has been collected. Withdrawal from STAREE-MIND will not affect participants' continued participation in the STAREE RCT.

## 5 Measurements and Analytical Methods

### 5.1 Schedule of study visits

The STAREE participant timeline is shown in Figure 1. STAREE MIND is a substudy of the STAREE RCT and the neuroimaging assessments will align with the STAREE Measurement and Activity schedule as shown in Table 1.

Each neuroimaging visit will take part during normal office hours, and will require 1 hour of the participant's time on a date convenient to them. The neuroimaging sessions will be undertaken at Monash Biomedical Imaging (770 Blackburn Road, Clayton) or the Herston Imaging Research Facility (located at the Royal Brisbane & Women's Hospital campus, Herston, QLD). Study staff will acquire informed consent. MRI scanning will be performed by a MRI clinical radiographer from Monash Health or Metro North Hospital and Health Service.

### 5.2 Procedures at each Neuroimaging visit

At each STAREE-MIND neuroimaging visit the following steps will be undertaken:

1. The explanatory statement will be reviewed with the participant and written informed consent will be ascertained.

2. Participants will be screened for MRI contra-indications (e.g., pacemaker, implants, etc) by the MRI radiographer.



Zoungas S, et al. BMJ Open 2023; 13:e069915. doi: 10.1136/bmjopen-2022-069915

3. The participant will be prepared for scanning. This involves lying down on the scanner bed, inserting ear-plugs, and being provided with the 'emergency' buzzer (that can be used to terminate the scan), and being checked for claustrophobia.

4. The participant will be required to remain still for the 45-min scan period while MR measures are acquired.

5. The participant will be offered $20 re-imbursement for travel expenses and time.

**5.3 MRI procedure**

Standard MRI acquisitions will be obtained over no more than 45mins, and will include:

- Structural T1- and T2-weighted images of brain anatomy

- Susceptibility weighted imaging (SWI) for quantitative susceptibility mapping

- Diffusion-weighted imaging (DWI) of brain white matter microstructure

- Arterial Spin Labelling (ASL) of brain perfusion

- Phase-contrast angiography of carotid blood flow

All acquisitions are non-invasive and participants remain passive throughout, with no required responses or inputs.



Zoungas S, *et al. BMJ Open* 2023; 13:e069915. doi: 10.1136/bmjopen-2022-069915

Figure 1: STAREE and STAREE-MIND (in red) Participant Timeline (UPDATED)

**Call**

- Particpant calls study centre
- Participant screened

**Baseline Visits - STAREE**

- STAREE tria informed consent obtained
- Questionnaires and baseline assessments
- Review inclusion/excusion criteria

**Randomisation**

- Participants randomised to placebo or active statin
- Participants asked if they wish to participate in the STAREE-MIND substudy
- Participants consented to STAREE-MIND substudy
- Participants to attend STAREE-MIND neuroimaging assessment

**Post randomisation and STAREE-MIND visit**

- Participants posted STAREE study drug (continued annually)
- Participants to take 1 tablet of study drug

**Call 4 weeks after randomisation**

- Participants to increase dosage to 2 tablets of study drug

**Annually**

- Participants will be phoned annually at 6, 18, 30, 42 and 54 months

**Annually**

- Participants will attend an annual visit at 12, 24, 36, 48 and 60 months in person or by phone



Zoungas S, *et al. BMJ Open* 2023; 13:e069915. doi: 10.1136/bmjopen-2022-069915

**Table 1: STAREE measurement and study activity schedule**. X indicates that all measures carried out, except if a superscript is present, indicating only those tests are performed at that time point. (UPDATED)

| | Screening | Baseline visits | | | | Follow-up visits |
|---|---|---|---|---|---|---|
| Measurement/Activity | Screening call | Baseline Visit 1 | GP visit | Baseline Visit 2 | Post-randomisation | Follow-up (year 4) |
| **Type of visit** | phone | clinic | clinic | clinic | phone | Phone or clinic |
| **Month in trial (week)** | 0 | 0 | 0 | 0 | (0-4) | 48 |
| **Review inclusion/Exclusion criteria** | X | X | X | X | | |
| **Obtain informed consent** | | X | | | | |
| **Dispense study medication** | | X (run-in med only) | | Post (post randomisation) | | X |
| **Medication titration** | | | | | X | |
| **Assess medication compliance** | | | | X | | X |
| **Concomitant medications** | | X | | | | X |
| **Blood pressure & heart rate/rhythm[a], height[c], weight[b], waist:hip circumference[b]** | | X[a,b,c] | | | | As per STAREE protocol |
| **Demographics, family[1] & personal history & lifestyle factors, physical function** | | X[1] | | | | X |
| **Routine Laboratory testing, GP referral to local pathology**<br><br>Fasting bloods: total cholesterol[1], HDL[1], LDL[1], triglyceride[1], CK[2], glucose[2], Hb[2] creatinine (eGFR)[3], ALT[3], AST[3], HbA1c[3],<br><br>Urine: albumin:creatinine ratio[3] | | X[1,2,3] | | | | X[3] |
| **Quality of life**<br><br>-SF-36 | | | | X | | X |
| **Assess cognitive function** | | X | | | | As per STAREE protocol |

| | | | | | | |
|---|---|---|---|---|---|---|
| - 3MS (and CES-D 10 to exclude depression); CAM and ADCS-IADL as required | | | | | | |
| **Detailed cognitive tests**<br><br>- COWAT, Stroop, HVLT-R, SDMT, Constructional praxis, Lurian overlapping figures, Trail Making Test | | As per STAREE protocol | | As per STAREE protocol | | As per STAREE protocol |
| **Assess physical disability**<br><br>- Life Ability ADLs | | X | | | | X |
| **Biobank samples collected** | | | | X | | |
| **Clinical event reporting**<br><br>- Questionnaire & medical report review | | | | | | X |
| **STAREE-MIND substudy neuroimaging (MRI)*** | | | | | X | X |

*STAREE-MIND neuroimaging will be conducted at entry to the STAREE RCT (between Baseline visit 2 and Randomisation). All neuroimaging assessments will be conducted at the Monash Biomedical Imaging Facility (located at Blackburn Rd, Clayton) or the Herston Imaging Research Facility (located at the Royal Brisbane & Women's Hospital campus, Herston, QLD). Follow-up visits will take place in year 4.



Zoungas S, *et al. BMJ Open* 2023; 13:e069915. doi: 10.1136/bmjopen-2022-069915

# 6 Adherence to Ethical, Regulatory and Administrative Considerations

## 6.1 Ethical considerations

### 6.1.1 *General*

This study will be conducted in accordance with the Declaration of Helsinki 1964 as revised in Edinburgh in 2000 and with the National Health & Medical Research Council Guidelines on Human Experimentation.

### 6.1.2 *Ethics Committee Approval*

The primary ethics committee for the parent STAREE study is the Monash University Human Research Ethics Committee (CF14/1927 – 2014000975).
The study is also approved by the Governance Office (SSA) in Royal Brisbane and Women's Hospital (TBC).

### 6.1.3 *Information for participants*

Before obtaining consent from the participant they must be informed of the objectives, benefits, risks and requirements of the STAREE-MIND substudy, as well as the nature of the neuroimaging assessments. A participant consent form should be given to every participant at STAREE Baseline visit 1.

### 6.1.4 *Informed consent*

a) All STAREE participants must give their informed consent **before** they can be enrolled in the STAREE-MIND substudy.
b) Two copies of the consent form are to be provided, one for the participant and one for the investigator.
c) Informed consent is obtained from the participant by the site investigator and/or by the research staff. The research staff should fully inform the participant of all pertinent aspects of the STAREE-MIND substudy by reviewing the study information and consent form. All participants should be informed to the fullest extent possible about the study, in language and terms they are able to understand.
d) Prior to a participant's involvement in the study, the written Informed Consent Form should be signed, name filled in and personally dated by the participant and by the person who conducted the informed consent discussion. A copy of the signed and dated written Informed Consent Form will be provided to the participant. The original consent form is to be stored in the participant's individual study file, held by the investigator. A copy of signed, informed consent must be cited by study staff at the relevant Imaging Facility prior to commencement of the data collection session.
e) The form used for obtaining the participant's informed consent must be the current version that has been reviewed and approved by the appropriate Ethics Committee.



Zoungas S, *et al. BMJ Open* 2023; 13:e069915. doi: 10.1136/bmjopen-2022-069915

## 6.2 Regulatory considerations

### 6.1.5 *Financing*

STAREE MIND neuroimaging substudy has been awarded a project grant from the National Heart Foundation (Australia).

### 6.1.6 *Trial registration*

STAREE is registered with clinicaltrials.gov (Identifier NCT02099123). First posted March 28, 2014.

### 6.1.7 *Disclosure of conflict of interest*

Full disclosure by all of the investigators of their, and their immediate families, financial relationships with all pharmaceutical and biomedical companies judged to have an active or potential interest in the conduct and outcome of the study will be made.

## 6.3 Indemnity

Monash University shall at all times indemnify the study investigators and their staff from claims that may be made against them for any injury sustained by a study participant as a consequence of the follow-up for this study as outlined in this protocol. GPs insurers will be asked to indemnify the GP co-investigators who are participating in the study. Ramsay Healthcare will indemnify the study medication.

## 6.4 Governance

STAREE-MIND is overseen and co-ordinated by a steering committee comprised of Prof Sophia Zoungas (chair), Monash University; Prof John McNeil, Monash University; Dr Trevor Chong, Monash University; Dr Joanne Ryan, Monash University; Dr Ian Harding, Monash University.

The Steering Committee will provide oversight to the overall policy and direction of the project. It will be responsible for protocol generation and, where appropriate, modification, budgeting and all funding applications. The Steering Committee will meet regularly throughout the planning, execution and data analysis phases of the project, when it will consider reports from the Study Director and the EAC. The Steering Committee will be responsible for all the publications and communications relating to the study.

# 7 Data Management

## 7.1 Data handling and record keeping

All the data from assessments conducted during STAREE-MIND will be recorded as scanned images for each participant. All information will be treated in accordance with professional conduct. Scanned images will be stored on secure data servers at Monash University, and curated by staff at Monash Biomedical Imaging and Monash University IT (eSolutions). Scans from HIRF will be exported to Monash data servers, and stored locally temporarily for clinical reporting and short-term data redundancy.



Zoungas S, *et al. BMJ Open* 2023; 13:e069915. doi: 10.1136/bmjopen-2022-069915

## 8 References


1. Australian Government Department of Health. Australian Statistics on Medicines 2015.
2. Wood, W.G., Eckert, G.P., Igbavboa, U. & Muller, W.E. Statins and neuroprotection: a prescription to move the field forward. *Annals of the New York Academy of Sciences* **1199**, 69-76 (2010).
3. Bettermann, K., *et al.* Statins, risk of dementia, and cognitive function: secondary analysis of the ginkgo evaluation of memory study. *Journal of stroke and cerebrovascular diseases : the official journal of National Stroke Association* **21**, 436-444 (2012).
4. Mattson, M.P. Pathways towards and away from Alzheimer's disease. *Nature* **430**, 631-639 (2004).
5. Bodovitz, S. & Klein, W.L. Cholesterol modulates alpha-secretase cleavage of amyloid precursor protein. *The Journal of biological chemistry* **271**, 4436-4440 (1996).
6. Refolo, L.M., *et al.* Hypercholesterolemia accelerates the Alzheimer's amyloid pathology in a transgenic mouse model. *Neurobiology of disease* **7**, 321-331 (2000).
7. Urmoneit, B., Turner, J. & Dyrks, T. Cationic lipids and disturbance of cellular cholesterol and sphingomyelin distribution modulates gamma-secretase activity within amyloid precursor protein in vitro. *Prostaglandins & other lipid mediators* **55**, 331-343 (1998).
8. Sparks, D.L., Liu, H., Gross, D.R. & Scheff, S.W. Increased density of cortical apolipoprotein E immunoreactive neurons in rabbit brain after dietary administration of cholesterol. *Neuroscience letters* **187**, 142-144 (1995).
9. Dolan, H., *et al.* Atherosclerosis, dementia, and Alzheimer disease in the Baltimore Longitudinal Study of Aging cohort. *Annals of neurology* **68**, 231-240 (2010).
10. Barone, E., Di Domenico, F. & Butterfield, D.A. Statins more than cholesterol lowering agents in Alzheimer disease: their pleiotropic functions as potential therapeutic targets. *Biochemical pharmacology* **88**, 605-616 (2014).
11. Wyss-Coray, T. Inflammation in Alzheimer disease: driving force, bystander or beneficial response? *Nature medicine* **12**, 1005-1015 (2006).
12. Griffin, W.S., *et al.* Brain interleukin 1 and S-100 immunoreactivity are elevated in Down syndrome and Alzheimer disease. *Proceedings of the National Academy of Sciences of the United States of America* **86**, 7611-7615 (1989).
13. Engelhart, M.J., *et al.* Inflammatory proteins in plasma and the risk of dementia: the rotterdam study. *Archives of neurology* **61**, 668-672 (2004).
14. Peila, R. & Launer, L.J. Inflammation and dementia: epidemiologic evidence. *Acta neurologica Scandinavica. Supplementum* **185**, 102-106 (2006).
15. Schmidt, R., *et al.* Early inflammation and dementia: a 25-year follow-up of the Honolulu-Asia Aging Study. *Annals of neurology* **52**, 168-174 (2002).
16. Swardfager, W., *et al.* A meta-analysis of cytokines in Alzheimer's disease. *Biological psychiatry* **68**, 930-941 (2010).
17. Menge, T., Hartung, H.P. & Stuve, O. Statins--a cure-all for the brain? *Nature reviews. Neuroscience* **6**, 325-331 (2005).
18. Wang, Q., *et al.* Statins: multiple neuroprotective mechanisms in neurodegenerative diseases. *Experimental neurology* **230**, 27-34 (2011).

19. PBS Information Management Section. Pharmaceutical Policy Branch. Expenditure and prescriptions twelve months to 30 June 2013 PBS.
20. Simons, L.A., Ortiz, M. & Calcino, G. Long term persistence with statin therapy -- experience in Australia 2006-2010. *Australian family physician* **40**, 319-322 (2011).
21. Morgan, T.K.*, et al.* A national census of medicines use: a 24-hour snapshot of Australians aged 50 years and older. *The Medical journal of Australia* **196**, 50-53 (2012).
22. Ko, D.T., Mamdani, M. & Alter, D.A. Lipid-lowering therapy with statins in high-risk elderly patients: the treatment-risk paradox. *JAMA : the journal of the American Medical Association* **291**, 1864-1870 (2004).
23. Pencina, M.J.*, et al.* Application of new cholesterol guidelines to a population-based sample. *The New England journal of medicine* **370**, 1422-1431 (2014).
24. Zissimopoulos, J.M., Barthold, D., Brinton, R.D. & Joyce, G. Sex and Race Differences in the Association Between Statin Use and the Incidence of Alzheimer Disease. *JAMA Neurol* **74**, 225-232 (2017).
25. Geifman, N., Brinton, R.D., Kennedy, R.E., Schneider, L.S. & Butte, A.J. Evidence for benefit of statins to modify cognitive decline and risk in Alzheimer's disease. *Alzheimer's research & therapy* **9**, 10 (2017).
26. Ott, B.R.*, et al.* Do Statins Impair Cognition? A Systematic Review and Meta-Analysis of Randomized Controlled Trials. *Journal of general internal medicine* (2015).
27. FDA drug safety communication: Important safety label changes to cholesterol-lowering statin drugs 2012.
28. Begg, S.*, et al. The burden of disease and injury in Australia, 2003*, (Australian Institute of Health and Welfare (AIHW), Canberra, 2007).
29. Australian institute of Health and Welfare. Canberra. Dementia in Australia. Cat. no. AGE 70. Canberra: AIHW. (2012).

## Appendix 11: Detailed protocol revisions

| No. | Section | Section | Initials/Date |
|---|---|---|---|
| Version 1.2.2 June 2014 | Original approved version (drafts leading up to next version) | | |
| Version 1.3 March 2015 | Cognitive tests added | 5.2<br>7.1.5<br>Study Activity Schedule | SS 27/4/2015 |
| | CES-D-10 added | 5.2<br>7.1.2<br>Study Activity Schedule | |
| | Change from 2 yearly annual visits to yearly annual visits | 7.1.8<br>Study Activity Schedule | |
| | Biobank sub-study – sample collection | 7.1.5<br>Study Activity Schedule | |
| | One month follow up visit added | 7.1.7 | |
| Version 1.3.1 July 2015 | SF-12 changed to SF-36 | 5.2 | SS 17/8/2015 |
| | Biobank Steering Committee added to Governance structure | 12.5.1 | |
| Version 1.3.2 January 2016 | Endpoint definitions expanded | 5.2, 5.3, 11.1, 11.2<br>Appendix 2 | FOH 27/1/2016 |
| | Study medication post out change | | |
| | Study medication compliance questionnaire added | Appendix 4 | |
| | Table of contents added | | |
| | Expansion of SAE and SUSAR reporting | 11.2 | |
| Version 1.3.3 July 2016 | Updated exclusion criteria regarding cytochrome P450 3A4 inhibitors | 6.2<br>8.2.1 (added)<br>Appendix 3 | SS 13/7/2016 |
| | Community participant follow up added | 6.3.1 | |
| | International Steering Committee added | 12.5.1 | FO 14/7/2016 |

| | | | |
|---|---|---|---|
| | Change in biobank study samples collected | 7.1.5 | SS<br>13/9/2016 |
| | Addition of reporting requirements for AEs and SAEs | 11.1.0<br>Appendix 5 | SS<br>22/3/2017 |
| Version 2.0<br>July 2018 | Updates to align protocol with SPIRIT 2013 statement recommendations | Across protocol | AC/SZ/SS<br>18/7/2018 |
| | Addition of STAREE hubs | Appendix 1 | SS<br>18/7/2018 |
| | Addition of Data access policy | Section 12.4 | AC<br>12/3/2019 |
| | Addition of secondary endpoints and clarification of both primary and secondary endpoints | Section 5 | AC<br>12/3/2019 |
| | Addition of new Forest plot | Section 1.2 | AC<br>12/3/2019 |
| | Addition of single-lead ECG, IPAQ-E and TICS-M to measurement and study schedule | Table 2 | AC/SS<br>13/9/2019 |
| | Updated exclusion criteria regarding cytochrome P450 3A4 inhibitors | Appendix 3 | AC/IH/SS<br>9/9/2019 |
| | Updated dementia definition from DSM IV to DSM V | Appendix 2 | ES/SZ/SS<br>13/9/2019 |
| Version 3.0 | Removal of Bictegravir from table | Appendix 3 | AK<br>10/2/20 |
| | Addition of information on data security | Section 12.4 | AC/SS<br>10/2/20 |
| | Clarification of definition of disability free survival | Section 5.1 p 27 | AC<br>10/2/20 |
| | Modification of dementia and cognitive decline endpoint brief definitions | Appendix 2 | AC<br>13/2/20 &<br>July 2022 |
| | COVID response – schedule update | Appendix 9/ Table 2 | SS<br>27/05/2020 |
| | Modification of heart failure endpoint brief definition | Appendix 2 | AC<br>1/07/2020 |



Zoungas S, *et al. BMJ Open* 2023; 13:e069915. doi: 10.1136/bmjopen-2022-069915

| | | | |
|---|---|---|---|
| | Changes to power calculations, sample size and follow-up period | Section 10 | |
| | Addition of Executive Summary | Section 1 | AC 1/06/2021 |
| | Addition of STAREE MIND protocol | Appendix 10 | AC 1/06/2021 |
| | Extended Revisions table moved to appendices and replaced with summary revision table | Appendix 11 | AC 1/06/2021 |
| *Version 3.2* | Addition of Frailty phenotype as a pre-specified tertiary endpoint | Section 3.3, Section 6, Appendix 2 | AC 13/02/2023 |
| | Corrected references in Appendix 6 | Appendix 6 | AC 13/02/2023 |



Zoungas S, *et al. BMJ Open* 2023; 13:e069915. doi: 10.1136/bmjopen-2022-069915
