# Supplementary material for: Statins for extension of disability-free survival and primary prevention of cardiovascular events among older people: protocol for a randomised controlled trial in primary care (STAREE trial)
Source: BMJ Open. 2023 Apr 3;13(4):e069915. doi: 10.1136/bmjopen-2022-069915 (PMC10083753; doi:10.1136/bmjopen-2022-069915)
Supplement: Supplementary data [file bmjopen-2022-069915supp004.pdf]

PARTICIPANT CONSENT FORM

MONASH UNIVERSITY

PARTICIPANT INFORMATION SHEET/CONSENT FORM

MONASH UNIVERSITY

Full Project Title: The STAREE Healthy Ageing Biobank

This participant information and consent form is 9 pages long. Please make sure you have all the pages. For any queries, information on the project or to speak to the research team please call **1800 770 664**.

INTRODUCTION

You are being invited to provide a blood and urine sample for the STAREE Healthy Ageing Biobank because you are a participant in the STAREE Clinical Trial. The sample you provide for the STAREE Healthy Ageing Biobank is in addition to any blood and urine that you have taken for the STAREE clinical trial. This is because research using blood and urine samples may help us to find out more about what causes disease, how to prevent it, and how to treat it. The research project aims to create a collection of blood and urine samples which scientists can then use in the future to identify biomarkers or genes associated with conditions such as heart attacks, strokes, cancer and Alzheimer's disease.

This Participant Information and Consent Form tells you about the research project. It explains what is involved to help you decide if you want to take part.

Please read this information carefully. Ask questions about anything that you don't understand or want to know more about. Before deciding whether or not to take part, you might want to talk about it with a relative, friend or your local health worker.

Participation in this research is voluntary. If you don't wish to take part, you don't have to. Note that consent for this additional research is separate to the informed consent you have already provided for participation in the STAREE Clinical Trial. If you do not wish to take part in this additional project you may still participate in the STAREE Clinical Trial. Additionally, consent can be given for different parts of the study, as indicated by the checked boxes at the end of this consent form.

If you decide you want to take part in the research project, you will be asked to sign the consent section. By signing it you are telling us that you:

- understand what you have read;
- consent to take part in the research project;

PARTICIPANT CONSENT FORM

MONASH UNIVERSITY

- consent to be involved in the procedures described;
- consent to the use of your personal and health information as described.

You will be given a copy of this Participant Information and Consent Form to keep.

WHAT IS THE PURPOSE OF THIS RESEARCH PROJECT?

- *What are biomarkers and genes, and why are they important?*

Biomarkers, including proteins, which are found in the blood and urine, are molecules that are needed for the structure, function and regulation of cells that make up the body's tissues and organs. We are still learning about the proteins that are present in healthy people such as yourself.

In addition to proteins, genetic material (or DNA) can also be collected from blood and urine. The unique way in which our many genes are arranged, is what makes us all different. However there are some sections of DNA which will almost always cause some diseases, or at least increase the risk of someone suffering from a condition or disease. We still have much to learn about these critical sections of DNA. For example, it is estimated that the genetic predisposition for heart disease may involve more than 200 genes. However the exact role is known for only a small number of these genes.

Previous studies have shown that changes in certain proteins and genetic material can be detected in people with cardiovascular disease, forms of cancer, and in Alzheimer's disease. However much more work needs to be carried out before we know which particular proteins and genes play the biggest role in actually causing these diseases.

- What is this study about?

We would like to see whether we can identify known or new proteins and/or genes in the blood and urine that may be linked with the incidence of strokes, heart attacks, bleeding episodes, changes in mental function and development of cancer.

We are seeking to collect blood and urine samples from participants who decide to take part in the STAREE Clinical Trial.

This research has been funded by Monash University.

PARTICIPANT CONSENT FORM

MONASH UNIVERSITY

WHAT DOES PARTICIPATION IN THIS RESEARCH PROJECT INVOLVE?

Procedures: We are asking you to provide a blood and urine sample for the STAREE Biobank.

At a STAREE Biobank visit, either a research nurse, or other trained professional, will collect the blood sample for the STAREE Biobank. You will be provided with a collection cup for the urine sample.

The sample collection for the Biobank involves taking 30–40 ml (just over 2 tablespoons) of your blood and collecting up to 70mls of your urine, and will add about 15 minutes to the length of your second visit or take about 15 minutes at a convenient location near you.

You are **not** required to fast before providing the additional blood and urine sample for the STAREE Biobank. After you have provided the blood and urine sample, it will have your name and any identifying features removed, and replaced with a code number. That code number will be the only information attached to your sample.

Your blood and urine samples will be processed and transported (if necessary) to the STAREE Healthy Ageing Biobank laboratory at the Clinical Trials Centre, Monash University, for storage (and other locations, such as the Alfred hospital, may be used as long-term storage sites in future). Research staff at this laboratory will not be able to link the code number to your identity and therefore will not know to whom the sample belongs.

Follow-up: If you agree to provide a blood and urine sample for the Biobank, but do not ultimately continue on in the STAREE Clinical Trial for whatever reason, you can still choose to remain a participant in the Biobank. This will mean that your stored blood and urine samples are kept in the STAREE Biobank, and we will contact you on an annual basis for up to 5 years to ask you a few questions about your health. We will also continue to have access to your medical records (your doctor's records, and those of any hospital or specialist whom you may visit).

We also ask you for permission to obtain information about you from a government database known as the National Death Index. Providing us with this information greatly facilitates us knowing about your health status.

(If you remain in the STAREE Clinical Trial, follow-up will consist of the above, but annual contact will occur via face-to-face visits with the research staff).

You may be asked to provide another 30–40 ml of your blood and up to 70mls of urine at another time during the study, such as at the 3 year time point and/or at the end of the study (in about 5 years).

Zoungas S, *et al. BMJ Open* 2023; 13:e069915. doi: 10.1136/bmjopen-2022-069915

PARTICIPANT CONSENT FORM

MONASH UNIVERSITY

Reimbursement: You will not be paid for your participation in this research.

Although knowledge acquired through medical research may lead to discoveries that are of commercial value, there will be no financial benefit to yourself, your family or the STAREE Biobank.

WHAT ARE THE POSSIBLE BENEFITS?

You will not benefit directly from the results of this research, but it is hoped that the results of research on your blood and urine, and those from other participants, may help doctors gain further understanding of how to reduce the risk of death, heart attacks, strokes, heart failure, cancer or dementia in people like yourself. Your blood and urine will be helpful whether you develop a major disease or not.

WHAT ARE THE POSSIBLE RISKS?

Blood collection: When blood samples are taken from a vein in your arm you may experience some discomfort. A small number of participants may faint, have some bruising, or a minor infection or blood clot form under the skin. These are generally minor discomforts which should resolve themselves in a few days.

If you should feel light-headed or dizzy during or after the blood sample collection procedure you will be encouraged to remain at the Clinical Trials Centre (or alternative blood collection site) until you feel comfortable leaving.

Confidentiality: The code number assigned to your blood and urine sample will be linked to your personal information that is stored on a separate database held at Monash University, Department of Epidemiology & Preventive Medicine. Therefore, the possibility exists that your blood and urine sample could be re-identified, however this is most unlikely to occur – see the section **What will happen to information about me?**

Genetic testing: Genetic testing of samples may occur in the future if additional funding is available. Any future projects must be approved by a Human Research Ethics Committee. If any future research uncovers any significant information specific to your health, our STAREE Steering Committee may decide to authorise someone to contact you and offer you access to this information. <u>You may decline the information</u>. "Significant" information is any findings that are deemed very serious or highly significant, that have been validated and to which there is known medical support available. If you wish to be given this information a qualified person will explain it to you. You should also consider whether this information should be made known to your family members. Sharing these findings could help avoid similar medical problems in your family. Before deciding whether or not to receive these results, you may want to discuss this with a relative or a significant other first. By signing this consent, you will

PARTICIPANT CONSENT FORM

MONASH UNIVERSITY

be asked to select if you wish to be informed or not informed of any serious or highly significant genetic results in the future that has any specific health implications for you or your genetic relatives – see page 9.

DO I HAVE TO TAKE PART IN THIS RESEARCH PROJECT?

Participation in any research project is voluntary. If you do not wish to take part, you do not have to. If you decide to take part and later change your mind, you are free to withdraw from the project at a later stage.

If you decide to withdraw, please notify a member of the STAREE research team in writing. This will allow that person or the research supervisor to inform you if there are any special requirements linked to withdrawing.

If you decide to leave the project, the researchers would like to keep the blood and urine samples and the personal and health information about you that has already been collected. This is to help them make sure that the results of the research can be measured properly. However, you may elect to have samples destroyed and health and personal information removed from the study. If you choose to have your samples and information removed you must tell the researchers before you withdraw from the research project.

Your decision whether to take part or not, or to take part and then withdraw, will not affect your relationship with the researchers or your doctor.

HOW WILL I BE INFORMED OF THE FINAL RESULTS OF THIS RESEARCH PROJECT?

The establishment of the STAREE Healthy Ageing Biobank is purely for research purposes only. At this formative stage, the Biobank cannot be used for the diagnosis of diseases because we do not yet know which particular biomarkers or genes will emerge, in the years to come, as being the most significant in terms of predicting disease onset. The ongoing nature of the development of the Biobank means that the final results of this project will not be available for some considerable time. If however in the future, genetic testing is undertaken and you consent to take part in this study, you will be asked at the end of this information sheet to consider being notified or not notified of any significant result (see also page 9).

Eventually, results of group data from the research that is conducted will be published in medical journals, and presented at national and international conferences.

WHAT WILL HAPPEN TO INFORMATION ABOUT ME?

The STAREE Biobank laboratory is equipped to store and handle blood and urine samples appropriately for future tests. Your potentially re-identifiable blood and urine samples will be

Zoungas S, *et al. BMJ Open* 2023; 13:e069915. doi: 10.1136/bmjopen-2022-069915

PARTICIPANT CONSENT FORM

MONASH UNIVERSITY

stored for a long time, possibly for many years after the completion of the study. When all the samples are no longer required they will be destroyed by the STAREE Biobank laboratory, or the laboratory in which the research has been conducted, in a safe manner according to local laboratory procedures.

Who will make use of my blood and urine samples?

The STAREE Biobank is a non-profit service dedicated to providing a resource to scientists involved in medical research. Researchers from universities, hospitals, and other health organizations conduct research using blood and urine. They may contact the STAREE Biobank and request samples for their studies. The researcher must have funding to carry out the study, and must have the study reviewed scientifically, before they are permitted to access the blood and urine samples from the STAREE Biobank. All studies must have approval by a Human Research Ethics Committee. Your samples will NOT be sold. The STAREE Biobank may charge researchers a fee to recover some of the costs of storing and administering its collection of samples.

Your blood and urine sample will not be linked to your name, initials, date of birth or any other information that may identify you.

What types of tests will be performed?

The tests performed on your blood and urine may include tests for inflammation, nutrition, insulin, hormones and genes that are thought to be related to heart disease, diabetes, dementia, cancer or other major diseases. Some researchers may develop new tests to identify diseases. Others may develop new ways to treat or even cure diseases. In the future, some of the research may help develop new products, such as tests and drugs.

Some research looks at diseases that are passed on in families (called familial or hereditary genetic research). Research done with your blood and urine may look for genetic causes and signs of disease.

Rapid advances in technology make it impossible to predict what new tests or studies may be possible in the future. The selection of tests will be guided by current and future scientific information about heart disease, diabetes, dementia, cancer or other major diseases.

You may consent to having your blood and urine sample tested for biomarkers, genetic markers, or both; see Consent Form, Page 9.

Any information obtained in connection with this research project that can identify you will remain confidential and will only be used for the purpose of this research project. It will only be disclosed with your permission, except as required by law.

Zoungas S, *et al. BMJ Open* 2023; 13:e069915. doi: 10.1136/bmjopen-2022-069915

PARTICIPANT CONSENT FORM

MONASH UNIVERSITY

In any publication and/or presentation, information will be provided in such a way that you cannot be identified. The STAREE Biobank and the STAREE Co-ordinating Centre (Monash University) are responsible for ensuring the information about you is kept private. They will take every precaution to safeguard your identity, and prevent misuse of records. Only authorised people who have agreed in writing to protect your identity will have access to your linked information. Therefore, all other researchers working with your samples and clinical information will not know your identity. This will make it very difficult for any research results (and importantly, those relating to genetic information) to be linked to you or your family. Also, people outside the research process will not have access to results about any one person, which will help to protect your privacy.

CAN I ACCESS RESEARCH INFORMATION KEPT ABOUT ME?

A report of the individual results of any tests done on your blood and urine samples will not routinely be made available to you, your family members or any other person. This is because research can take a long time and must use blood and/or urine samples from many people before results are known. Results from research using your samples may not be ready for many years and will not affect your care right now, but they may be helpful to people like you in the future.

In accordance with relevant Australian and/or Victorian privacy and other relevant laws, you have the right to access the information collected and stored by the researchers about you. Please contact one of the researchers named at the end of this document if you would like to access your information.

Further, in accordance with regulatory guidelines, the information collected in this research project will be kept indefinitely. You must be aware that the information may become de-identified at some point and access to information about you after this point will not be possible.

IS THIS RESEARCH APPROVED?

The ethical aspects of this research project have been approved by the Alfred Hospital Ethics Committee.

This project will be carried out according to the *National Statement on Ethical Conduct in Human Research (2007)* produced by the National Health and Medical Research Council of Australia. This statement has been developed to protect the interests of people who agree to participate in human research.

Zoungas S, *et al. BMJ Open* 2023; 13:e069915. doi: 10.1136/bmjopen-2022-069915

PARTICIPANT CONSENT FORM

MONASH UNIVERSITY

FURTHER INFORMATION AND WHO TO CONTACT

Please ask the research staff member if you have any further questions.

For specific enquiries related to this study, or matters relating to research at the site at which you are participating, or any questions about being a research participant in general, you may contact:

Name: Associate Professor Sophia Zoungas (Monash University):
Phone: +61 3 9903 0711
E-mail: sophia.zoungas@monash.edu

If you have any complaints about any aspect of the project or the way it is being conducted, you may contact:
Complaints Officer
Office of Ethics & Research Governance
Alfred Health

Tel: (03) 9076 3619

You will need to quote the following Alfred Health project number: 266/15.

Zoungas S, *et al. BMJ Open* 2023; 13:e069915. doi: 10.1136/bmjopen-2022-069915

PARTICIPANT CONSENT FORM

MONASH UNIVERSITY

Title The STAREE Healthy Ageing Biobank

DECLARATION BY PARTICIPANT

I have read, or have had read to me, the *"The STAREE Healthy Ageing Biobank" Patient Information, Version 1.6.1, Dated 17 April 2018* in a language that I understand, and I understand the purposes, procedures and risks of this research project as described within it.

I have had an opportunity to ask questions and I am satisfied with the answers I have received.

I freely agree to participate in this research project, as described.

I understand that I will be given a signed copy of this document to keep.

The researcher has agreed not to reveal my identity and personal details if information about this project is published or presented in any public form.

I agree to my blood and urine samples being stored and tested for **biomarkers** in the future

☐ YES ☐ NO

I agree to my blood and urine samples being stored and tested for **genetic markers** in the future

☐ YES ☐ NO

I agree to be **informed** of any significant genetic results that have specific health implications for me and/or my genetic relatives

☐ YES ☐ NO

Name of participant (please print)__________________________________

Signature______________________________Date_______________

DECLARATION BY RESEARCHER

I have given a verbal explanation of the research project, its procedures and risks and I believe that the participant has understood that explanation.

Name of researcher (please print)__________________________________

Signature______________________________Date_______________
