# Supplementary material for: Statins for extension of disability-free survival and primary prevention of cardiovascular events among older people: protocol for a randomised controlled trial in primary care (STAREE trial)
Source: BMJ Open. 2023 Apr 3;13(4):e069915. doi: 10.1136/bmjopen-2022-069915 (PMC10083753; doi:10.1136/bmjopen-2022-069915)
Supplement: Supplementary data [file bmjopen-2022-069915supp005.pdf]

Appendix 4 Assumptions for STAREE disability free survival (DFS) event rates

DFS event rates for STAREE were calculated based on the event rates for Australian participants in the ASPREE study.

ASPREE Australian participants – rates averaged over 4.7years of follow up (Ryan J, Storey E, Murray AM et al. Randomized placebo-controlled trial of the effects of aspirin on dementia and cognitive decline. Neurology. 2020 Jul 21;95(3):e320-e331)

70-74y:	3.42 per 1000 person-years
75-79y:	7.31 per 1000 person-years
80-84y:	16.00 per 1000 person-years
85+y:	21.37 per 1000 person-years

ASPREE Australian participants – rates in years 2-7 if zero events in first year of 4.7y of follow-up

70-74y:	4.39 per 1000 person-years
75-79y:	9.30 per 1000 person-years
80-84y:	20.24 per 1000 person-years
85+y:	27.52 per 1000 person-years

Assuming STAREE dementia process produces rates 33% higher

70-74y:	5.84 per 1000 person-years
75-79y:	12.37 per 1000 person-years
80-84y:	26.92 per 1000 person-years
85+y:	36.60 per 1000 person-years

DFS rates based on assumed dementia rates above and combining with assumed death and persistent physical disability rates as follows: (death+dementia+disability) less 10% to account for individuals experiencing more than one of the three types of event

70-74y:	(10.0	+ 5.84 + 4.5)*0.9	= 18.3 per 1000 person-years
75-79y:	(17.7	+ 12.37 + 7.6)*0.9	= 33.9 per 1000 person-years
80-84y:	(33.6	+ 26.92 + 12.5)*0.9	= 65.7 per 1000 person-years
85+y:	(66.6	+ 36.60 + 25)*0.9	= 115.4 per 1000 person-years
