# Supplementary material for: Statins for extension of disability-free survival and primary prevention of cardiovascular events among older people: protocol for a randomised controlled trial in primary care (STAREE trial)
Source: BMJ Open. 2023 Apr 3;13(4):e069915. doi: 10.1136/bmjopen-2022-069915 (PMC10083753; doi:10.1136/bmjopen-2022-069915)
Supplement: Supplementary data [file bmjopen-2022-069915supp006.pdf]

Appendix 5

Data and Safety Monitoring Board Charter for A randomised clinical trial of STAtin therapy for Reducing Events in the Elderly (STAREE)

(Short title: STAREE DSMB Charter)

Charter approved by Monash University

Signature: ________________________________ Date:____________

Sponsor Signatory: Professor Sophia Zoungas

DSMB Approved:

Signature: ________________________________ Date:____________

DSMB Chair:

1. Introduction

Trial name: Statins in Reducing Events in the Elderly (STAREE)

Trial objectives:

Scope of Charter: This charter outlines the composition and responsibilities of the STAREE DSMB.

2. DSMB responsibilities

The Data and Safety Monitoring Board (DSMB) will monitor participant safety, data quality and evaluate the progress of the study.

- Review the research protocol, informed consent documents and plans for data safety and monitoring;
- Review any proposed modifications to the trial prior to their implementation;
- Evaluate the progress of the trial, including periodic assessments of data quality and timeliness, recruitment, accrual and retention, participant risk versus benefit, performance of the trial sites, and other factors that can affect study outcomes;
- Consider factors external to the study when relevant information becomes available, such as scientific or therapeutic developments that may have an impact on the safety of the participants or the ethics of the trial;
- Review study performance, make recommendations and assist in the resolution of problems reported by the Principal Investigators;
- Make recommendations to the Principal Investigators concerning continuation, termination or other modifications of the trial based on the observed beneficial or adverse effects of the treatment under study;
- If appropriate, review interim analyses in accordance with stopping rules, which are clearly defined in advance of data analysis and have the approval of the DSMB; and
- Ensure the confidentiality of the study data and the results of monitoring.

3. Membership

The DSMB will consist of 4 members and 3 will constitute a quorum.

Membership consists of persons completely independent of the investigators who have no financial, scientific, or other conflict of interest with the trial. Written documentation attesting to absence of conflict of interest is required from each member.

Professor John Simes, University of Sydney, has been selected by the Principal Investigators to serve as the Chairperson and is responsible for overseeing the meetings and developing the agenda in consultation with the Principal Investigators. The Chair is the contact person for the DSMB. Monash University will provide the logistical management and support for the DSMB.

4. Organisation of meetings

At the first meeting the DSMB will discuss the protocol, suggested modifications and establish guidelines to study monitoring by the Board. In consultation with the Principal Investigator, the DSMB chairperson will prepare the agenda to address the review of study materials, modifications to the study protocol and informed consent document, initiation of the trial, appointment of a safety

officer, reporting of adverse events, statistical analysis plan including interim analysis and stopping rules and/or other matters as appropriate.

Meetings of the DSMB will be held annually for the first two years and 6 monthly thereafter. An emergency meeting of the DSMB may be called at any time by the Chairperson if participant safety questions or other unanticipated problems arise.

Meetings will be closed to the public because discussions may address confidential participant data. Meetings may be convened as conference calls as well as in person.

Meetings will consist of open and closed sessions. Discussion held in all sessions will be confidential.

At least one of the Principle Investigators and key members of the study team will attend open sessions. Open session discussion will focus on the conduct and progress of the study, including participant accrual, protocol compliance and problems encountered. Unblinded data will not be presented in the open sessions.

Closed sessions will be attended by the DSMB members. The study statistician may be present at the request of the DSMB. Any data by blinded study group and, as necessary, unblinded data, will be presented during the closed session. The DSMB can request review of unblinded data during the closed session. Investigators will still remain blinded.

Each meeting must include a recommendation to continue or terminate the study made by a formal DSMB majority or unanimous vote. Should the DSMB decide to issue a termination recommendation, the full vote of the DSMB is required. A recommendation to terminate the study may be made by the DSMB at any time by majority vote.

Following each DSMB meeting, the Chair will formally write to the STAREE Chief Investigators to advise them that the monitoring of the study's progress is up to date, and that the DSMB recommends study continuation or discontinuation (whichever applies).

5. Documentation

DSMB interim report templates will be prepared by the study staff, typically the statistician, to be reviewed by the DSMB members at the first meeting. Interim data reports generally consist of two parts;

- Part 1 – Open session report, and
- Part 2 – Closed session report

The format and content of the reports for both the open and closed sessions and plans for interim analyses will be finalised and approved by the DSMB, although changes throughout the trial may be requested.

The reports will list and summarise safety data and describe the status of the study. All meeting materials will be sent to the DSMB at least 10 to 14 days prior to the meeting by secure email.

5.1 Open Session reports

Open session reports will include:

- Site administrative reports describing –
    - Numbers of participants screened, enrolled, completed and withdrawn
    - Baseline characteristics of the study population

   - General information on study status
   - Blinded listings of adverse events and serious adverse events as requested by the DSMB
   - Other information as requested by the DSMB on a continuing or one off basis

5.2 Closed Session reports

Closed session reports will include:

- Information described in Part 1 but by blinded treatment group (e.g. group A/ group B).
- Information on:
  - Study outcomes
  - Safety data
  - Efficacy data
  - Interim analyses

Closed session reports will be destroyed at the conclusion of the meeting. If meetings are held by telephone printed copies of the closed reports should be destroyed immediately following the meeting.

5.3 Additional reports

Mailings to the DSMB: As per the schedule (agreed by the DSMB) blinded safety data will be communicated to all or selected DSMB members

Access to Interim data: Access to the accumulating endpoint data will be limited to as small a group as possible, such as the independent statistician. Limiting the access of interim data to the DSMB members relieves the investigator of the burden of deciding whether it is ethical to continue to randomise individuals and helps protect the study from bias in the recruitment and/or evaluation.

5.4 Interim analyses

The DSMB will determine when interim analyses will take place. Interim analyses will be reviewed in closed sessions and may be presented in blinded group format (e.g. group A/ group B). The DSMB may also request review of the unblinded data.

Interim analyses of efficacy data will be performed only if they are planned and approved in advance and if stopping criteria are clearly defined.

6. DSMB reports

It is the responsibility of the Principal Investigators to distribute the DSMB recommendations to all co-investigators and to ensure that copies are submitted to all ethics committees associated with the study.

7. Confidentiality

All materials, discussions and proceedings of the DSMB are completely confidential. Members and other participants in DSMB meetings are expected to maintain confidentiality.
