# Supplementary material for: Statins for extension of disability-free survival and primary prevention of cardiovascular events among older people: protocol for a randomised controlled trial in primary care (STAREE trial)
Source: BMJ Open. 2023 Apr 3;13(4):e069915. doi: 10.1136/bmjopen-2022-069915 (PMC10083753; doi:10.1136/bmjopen-2022-069915)
Supplement: Supplementary data [file bmjopen-2022-069915supp007.pdf]

Appendix 6 – STAREE investigators, sites and acknowledgements

The STAREE investigator group includes the following:

Principal Investigator:

Prof. Sophia Zoungas MBBS (Hons) FRACP PhD

Steering Committee:

Prof. Sophia Zoungas (Chair), Prof. Lawrence Beilin, Ass. Prof. Trevor T-J Chong, Prof. Geoffrey C Cloud, Prof. John J McNeil, Prof. Mark R Nelson, Prof. Stephen J Nicholls, Prof. Christopher M Reid, Prof. Andrew Tonkin, Dr Stephanie A Ward, Prof. Anthony S Wierzbicki, Prof. Rory Wolfe

Executive Committee:

Prof. Sophia Zoungas (Chair), Dr Andrea J Curtis, Ass. Prof. Ingrid Hopper, Dr Alissia Kost, Prof. John J McNeil, Prof. Mark R Nelson, Prof. Christopher M Reid, Ass. Prof. Joanne Ryan, Dr Simone Spark, Prof. Rory Wolfe.

Data Safety Monitoring Committee:

Prof. John Simes (Chair), Prof. Graeme Hankey, Prof. A Mark Richards and Prof. Mark Woodward. Dr Alan Herschtal and Dr Thao Le (independent statisticians).

Endpoint Adjudication Committees:

Death:
Prof. Mark R Nelson (Chair), Prof. Danny Liew, Dr Vazhma Qaderi, Prof. Anthony S Wierzbicki.

Dementia/Disability:
Ass. Prof. Trevor T-J Chong (Chair), Dr Chris Moran, Dr Stephanie A Ward.

Myocardial Infarction:
Prof. Stephen J Nicholls (Chair), Ass. Prof. Dion Stub, Prof. Andrew Tonkin.

Stroke:
Prof. Geoffrey C Cloud (Chair), Prof.Craig Anderson, Dr Barry Moynihan, Prof. Michael O'Sullivan.

Heart Failure:
Ass. Prof. Ingrid Hopper (Chair), Prof. John Amerena, Prof. David Kaye.

General Practice Advisory Committee:

Prof. Mark R Nelson (Chair), Dr Gary Deed, Dr Alissia Kost.

Operations Committee:

Dr Simone Spark (Chair), Ms Lucy Boorn, Ms Sue Critchley, Dr Andrea J Curtis, Mr Zachary Flanagan, Mr Stephen Glanville, Dr Alissia Kost, Ms Kate Vaughan.

Data Access and Publications Policy Committee:

Prof. Sophia Zoungas, Dr Andrea J Curtis, Mr Zachary Flanagan, Prof. Christopher M Reid, Dr Simone Spark, Prof. Rory Wolfe.

STAREE Regional Leaders:

Victoria and South Australia:

Prof. Sophia Zoungas (Melbourne, Central Victoria, Northern Victoria, South West Victoria, Geelong, Gippsland, East Gippsland, Mildura, Adelaide, Mount Gambier)

Tasmania:

Prof. Mark R Nelson (Hobart, Launceston, Burnie)

Western Australia:

Prof. Christopher M Reid (Perth, Bunbury)

Queensland:

Dr Gary Deed (Brisbane, Gold Coast, Toowoomba, Sunshine Coast, Hervey Bay)

New South Wales:

Prof. Andrew Boyle (Sydney, Newcastle, Port Macquarie, Wollongong, North Coast, Tamworth)

Sub studies

STAREE-Heart Principal Investigator: Ass. Prof, Ingrid Hopper

STAREE-MIND Principal Investigator: Ass. Prof. Joanne Ryan

Acknowledgements

We thank all past and present STAREE staff.

We thank Professors Elsdon Storey and Robyn Langham for their past contributions to the STAREE trial Steering Committee.

A list of all current STAREE staff can be found at: https://www.monash.edu/medicine/staree/about-staree/our-team
