# Supplementary material for: Statins for extension of disability-free survival and primary prevention of cardiovascular events among older people: protocol for a randomised controlled trial in primary care (STAREE trial)
Source: BMJ Open. 2023 Apr 3;13(4):e069915. doi: 10.1136/bmjopen-2022-069915 (PMC10083753; doi:10.1136/bmjopen-2022-069915)
Supplement: Author's manuscript [file bmjopen-2022-069915.draft_revisions.pdf]

BMJ Open

BMJ Open is committed to open peer review. As part of this commitment we make the peer review history of every article we publish publicly available.

When an article is published we post the peer reviewers' comments and the authors' responses online. We also post the versions of the paper that were used during peer review. These are the versions that the peer review comments apply to.

The versions of the paper that follow are the versions that were submitted during the peer review process. They are not the versions of record or the final published versions. They should not be cited or distributed as the published version of this manuscript.

BMJ Open is an open access journal and the full, final, typeset and author-corrected version of record of the manuscript is available on our site with no access controls, subscription charges or pay-per-view fees (http://bmjopen.bmj.com).

If you have any questions on BMJ Open's open peer review process please email info.bmjopen@bmj.com

BMJ Open

# Statins for primary prevention among older people: protocol for a randomised controlled trial in primary care (STAREE trial)



| | |
|---|---|
| Journal: | *BMJ Open* |
| Manuscript ID | bmjopen-2022-069915 |
| Article Type: | Protocol |
| Date Submitted by the Author: | 07-Nov-2022 |



| | |
|---|---|
| Complete List of Authors: | Zoungas, Sophia; Monash University, School of Public Health and Preventive Medicine<br>Curtis, Andrea; Monash University, School of Public Health & Preventive Medicine<br>Spark, Simone; Monash University, School of Public Health and Preventive Medicine<br>Wolfe, Rory; Monash University, School of Public Health and Preventive Medicine<br>McNeil, John; Monash University, School of Public Health and Preventive Medicine<br>Beilin, Lawrence; University of Western Australia, School of Medicine<br>Chong, Trevor T-J; Monash University, Turner Institute for Brain and Mental Health; Alfred Health, Department of Neurology<br>Cloud, Geoffrey; Monash University, Department of Neuroscience; Alfred Health, Department of Neurology<br>Hopper, Ingrid; Monash University, School of Public Health and Preventive Medicine; Alfred Health, Department of Cardiology and General Medicine Unit<br>Kost, Alissia; Monash University, School of Public Health and Preventive Medicine<br>Nelson, Mark; University of Tasmania, Menzies Research Institute<br>Nicholls, Stephen; Monash University, School of Public Health and Preventive Medicine; Monash University, Victorian Heart Institute<br>Reid, Christopher; Monash University, School of Public Health and Preventive Medicine; Curtin University, School of Public Health<br>Ryan, Joanne; Monash University, School of Public Health and Preventive Medicine<br>Tonkin, Andrew; Monash University, School of Public Health and Preventive Medicine<br>Ward, Stephanie; Monash University, School of Public Health and Preventive Medicine; University of New South Wales, Centre for Healthy Brain Ageing, School of Psychiatry<br>Wierzbicki, Anthony; Guy's and St Thomas' Hospitals, Metabolic Medicine/Chemical Pathology |



| | |
|---|---|
| Keywords: | Dementia < NEUROLOGY, Ischaemic heart disease < CARDIOLOGY, PREVENTIVE MEDICINE |
| | |

# Statins for primary prevention among older people: protocol for a randomised controlled trial in primary care (STAREE trial)

**Authors:** Sophia Zoungas[1], Andrea J Curtis[1], Simone Spark[1], Rory Wolfe[1], John J McNeil[1], Lawrence Beilin[2], Trevor T-J Chong[3,4,5], Geoffrey C Cloud[4,6], Ingrid Hopper[1,7], Alissia Kost[1], Mark R Nelson[8], Stephen J Nicholls[1,9], Christopher M Reid[10], Joanne Ryan[1], Andrew Tonkin[1], Stephanie A Ward[1,11,12], Anthony S Wierzbicki[13] and the STAREE investigator group

Affiliations:

[1] School of Public Health and Preventive Medicine, Monash University, Melbourne, Australia.

[2] School of Medicine, Royal Perth Hospital, University of Western Australia, Perth, Western Australia.

[3] Turner Institute for Brain and Mental Health, Monash University, Victoria, Australia.

[4] Department of Neurology, Alfred Health, Melbourne, Victoria, Australia.

[5] Department of Clinical Neurosciences, St Vincent's Hospital, Melbourne, Victoria, Australia.

[6] Department of Neuroscience, Central Clinical School, Monash University, Melbourne, Australia.

[7] Department of Cardiology and General Medicine Unit, Alfred Health, Melbourne, Australia.

[8] Menzies Institute for Medical Research, University of Tasmania, Hobart, TAS, Australia.

[9] Victorian Heart Institute, Monash University, Clayton, Australia.

For peer review only



For peer review only - http://bmjopen.bmj.com/site/about/guidelines.xhtml

[10] School of Population Health, Curtin University, Perth, WA, Australia.

[11] Centre for Healthy Brain Ageing, School of Psychiatry, UNSW, Sydney, NSW.

[12] Department of Geriatric Medicine, The Prince of Wales Hospital, Randwick, NSW

[13] Department of Metabolic Medicine/Chemical Pathology, Guy's & St Thomas' Hospitals, London.

Corresponding author:

Professor Sophia Zoungas MBBS(Hons) PhD FRACP

School of Public Health and Preventive Medicine

Monash University

Melbourne, Australia

Email: sophia.zoungas@monash.edu

Phone: +61 3 9903 0711

Word Count: 4967

Keywords: statin, primary prevention, ageing, dementia, cardiovascular disease

For peer review only



For peer review only - http://bmjopen.bmj.com/site/about/guidelines.xhtml

## ABSTRACT

**Introduction:** The world is undergoing a demographic transition to an older population. Preventive healthcare has reduced the burden of chronic illness at younger ages but there is limited evidence that these advances can improve health at older ages. Statins are one class of drug with the potential to prevent or delay the onset of several causes of incapacity in older age, particularly major cardiovascular disease (CVD). This paper presents the protocol for the STAtins in Reducing Events in the Elderly (STAREE) trial, a randomised double-blind placebo-controlled trial (RCT) examining the effects of statins in community dwelling older people without CVD, diabetes or dementia.

**Methods and Analysis:** We will conduct a double blind, randomised placebo-controlled trial among people aged 70 years and over, recruited through Australian general practice and no history of clinical CVD, diabetes or dementia. Participants will be randomly assigned to oral atorvastatin (40 mg daily) or matching placebo (1:1 ratio). The co-primary endpoints are disability-free survival (DFS) defined as survival free of dementia and persistent physical disability and major CVD (cardiovascular death or non-fatal myocardial infarction or stroke). Secondary endpoints are all-cause death, dementia and other cognitive decline, persistent physical disability, fatal and non-fatal myocardial infarction, fatal and non-fatal stroke, heart failure, atrial fibrillation, fatal and non-fatal cancer, all-cause hospitalisation, need for permanent residential care and quality of life. Comparisons between assigned treatment arms will be on an intention-to-treat basis with each of the co-primary endpoints analysed separately in time-to-first-event analyses using Cox proportional hazards regression models.

**Ethics and Dissemination:** STAREE will address uncertainties about the preventive effects of statins on a range of clinical outcomes important to older people. Institutional ethics approval has been obtained. All research outputs will be disseminated to GP co-investigators


For peer review only - http://bmjopen.bmj.com/site/about/guidelines.xhtml

and participants, published in peer-reviewed journals and presented at national and international conferences.

## Trial registration number

ClinicalTrials.gov Identifier: NCT02099123. Date of registration: March 28, 2014.

## ARTICLE SUMMARY

### Strengths and limitations

- STAtins in Reducing Events in the Elderly (STAREE) is a double blind, placebo-controlled randomised clinical trial among a primary prevention population of people aged 70 years and over that will evaluate the effect of statin therapy on two co-primary outcomes including disability-free survival and major cardiovascular events.
- STAREE is a pragmatic trial run through general practice across metropolitan and regional/rural Australia with usual care provided by general practitioners.
- STAREE is one of the largest statin trials to carry out a comprehensive assessment of several causes of incapacity including dementia and disability.
- Complete vital status ascertainment will be facilitated by linkage to the National Death Index.
- STAREE will not be examining the effects of withdrawing statins or de-escalating therapy in older age.

For peer review only


For peer review only - http://bmjopen.bmj.com/site/about/guidelines.xhtml

## INTRODUCTION

The world is experiencing a rapid demographic transition towards an older population.[1] Increasing survival amongst older age groups is a major contributor to this shift, with almost twice as many older people surviving into their eighties and nineties compared to fifty years ago.[2] In the US, the proportion of the population aged 65 and over is projected to increase from 40 million in 2009 to 89 million in 2050 with 19 million (20%) in the oldest age group (85+) by 2050.[3] Similar trends are seen in other high income countries such as Australia, Canada, Japan, Europe and the United Kingdom.[2] However, over the same time period healthy life expectancy has not increased to the same extent as life expectancy.[4 5] While preventive therapies have reduced the burden of chronic diseases at younger ages, age remains an important determinant of cardiovascular disease (CVD), dementia and frailty.[6 7 8] The net impact of an ageing population is that more people are living with chronic diseases for longer, and these can lead to disability and loss of independence.[9 10] Loss of independence is not only one of the biggest concerns for older people[11] but has significant societal and economic impacts.[12-14] Identifying preventive interventions that can extend life free of dementia and disability (disability-free survival) is an important public health goal. One promising but insufficiently tested preventive approach to extending disability-free survival among older people is statin therapy.

### The debate about optimal LDL cholesterol levels, use of statin therapy and clinical outcomes in older people.

While high levels of LDL cholesterol (LDL-C) are known to increase the risk of CVD events and mortality among young and middle-aged populations [15 16], the relationship between LDL-C and clinical outcomes including all-cause or cause-specific death in older populations is not


For peer review only - http://bmjopen.bmj.com/site/about/guidelines.xhtml

consistent. Previous observational studies have reported a mix of no association, positive association or U-curve associations between LDL-C levels and all-cause death [17-20], cardiovascular events[21 22] and dementia.[16 23] One explanation for lack of an association or negative association is “reverse causality,” whereby unquantified comorbid disease may be causing both lower LDL-C levels and an increased risk of death or dementia.[24] Given the uncertainty around the relationship of LDL-C levels and outcomes in older people, the decision as to whether lipid lowering therapy such as statins should be initiated must be guided by randomised controlled trials. Furthermore, absolute CVD risk calculators, whose use is recommended to assist prescribing decisions for primary prevention, are principally driven by age such that universal treatment becomes the default option without certainty of the risks and benefit for older populations.[25]

Statins have the potential to delay the onset of several causes of incapacity in older people. Their best-recognised pharmacological action is to inhibit the mevalonate pathway and biosynthesis of cholesterol and isoprenoids by binding to HMG-CoA reductase. The reduction in circulating lipid levels, especially LDL-C, underpins their ability to reduce the incidence of acute myocardial infarction and stroke (including subclinical CVD) and their potential long-term sequelae (heart failure, cognitive decline).[26] Statins also exert a number of pleiotropic effects including anti-oxidative, anti-thrombotic and a moderately powerful anti-inflammatory action,[27] which could also help explain their reported beneficial effects in reducing disability and the risk of dementia.[28] The pleiotropic effects of statins have been attributed to the inhibition of isoprenoid metabolite formation (geranylgeranyl pyrophosphate and farnesyl pyrophosphate).[29] These metabolites are intermediates in the post-translational formation of several cell-signalling proteins that control multiple cell functions including maintenance of cell shape, motility, factor secretion, differentiation, and proliferation thus

For peer review only


For peer review only - http://bmjopen.bmj.com/site/about/guidelines.xhtml

explaining improvements in endothelial function, vascular inflammation and oxidation, and atherosclerotic plaque stability.[29] Statins also attenuate neuroinflammation, an enduring feature of neurodegenerative diseases through potent suppression of microglia (neuroimmune cells), activation and inhibition of cytokine production, and have been shown to mitigate the aggregation of amyloid β, a key factor in the development of Alzheimer's disease.[30 31]

In people with known CVD, the benefits of statin therapy are clear, with the risks of cardiovascular events reduced across all ages. In people without CVD, the benefits of taking a statin are not as clear at older ages and continue to be debated.[32 33] Most national and international guidelines have not been able to make strong recommendations to guide prescribing for older people, and any recommendations have generally been informed by subgroups within trials that have not studied the range of health outcomes meaningful to older people and that consider both beneficial and adverse effects.[34 35]

A meta-analysis of pooled individual participant data (aged 55 years +) from 28 statin trials that included participants with and without known CVD, reported a 21% reduction in risk of major vascular events per 1 mmol/L reduction in LDL-C, attributed to reduced risks of major coronary events, major stroke events and vascular death.[36] Analyses by age group indicated the greatest risk reductions (25% per 1 mmol/L reduction in LDL-C) among participants aged 55 years or less. This compared to risk reductions of 19% and 13% for older participants aged >70 to ≤75 and >75 years respectively.[36] The risk of all-cause death was also reduced, albeit to a much lesser extent (9% per 1 mmol/L reduction in LDL-C). There was no effect on non-vascular deaths including cancer deaths. Of note, participants were predominantly high risk, middle-aged men with high baseline LDL-C levels (mean weighted LDL-C level 3.7 mmol/L) or known CVD.[36] In analyses limited to participants with no history of vascular


For peer review only - http://bmjopen.bmj.com/site/about/guidelines.xhtml

disease, there was a trend toward smaller proportional risk reductions in major vascular events as age increased.[36] However, a *post hoc* pooling of subgroup data from 2 trials of rosuvastatin (the Justification for the Use of Statins in Prevention: an Intervention Trial Evaluating Rosuvastatin (JUPITER) trial, and the Heart Outcomes Prevention Evaluation (HOPE 3) trial, concluded that the effect on major vascular events in people aged ≥70 years (HR 0.74; 95% CI 0.61 to 0.91) was similar to those < 65 years (HR 0.75; 95% CI 0.57 to 0.97).[37] Importantly, the effects on dementia and physical disability were not comprehensively investigated by these studies.[38]

Yet statin treatment is not without potential risk of harm.[39] Observational studies have reported muscle weakness/pain, liver impairment, new-onset diabetes and haemorrhagic stroke[15 40 41] but the incidence of these adverse effects has been much lower in clinical trials.[42] Importantly, statins may interact with other medications, contributing to adverse drug reactions and avoidable hospitalisations.[43] Older adults exposed to polypharmacy are more vulnerable to adverse reactions and increased risks of adverse effects. As with efficacy, the burden of statin adverse effects in older people has not been well studied. As a result, it is still not possible to know the balance of risks and benefits in this age group.[44]

In conclusion, the gains made in increasing lifespan may not have been accompanied by similar gains in healthy life, for the additional later years of life lived. A strong potential exists for greater use of statins for primary prevention to extend healthy life and maintain independence amongst the ageing population however, current evidence among older people is insufficient to support this approach. The STAREE trial is a large scale RCT specifically designed to address this need.


For peer review only - http://bmjopen.bmj.com/site/about/guidelines.xhtml

## Study Objectives and Hypotheses

Primary objectives

To determine in people aged ≥70 years the effect of statin therapy (40 mg atorvastatin) versus placebo, on two co-primary clinical endpoints:

(i) a composite of all cause death or dementia or development of persistent physical disability; and

(ii) a composite of cardiovascular death, non-fatal myocardial infarction, or non-fatal stroke.

We hypothesise that treatment with a statin (atorvastatin 40 mg) will:

a) prolong overall disability free survival, and

b) reduce the incidence of major cardiovascular events amongst a primary prevention population aged ≥70 years.

Secondary objectives

To determine the effects of statin therapy (atorvastatin 40mg) versus placebo on each of all-cause death, dementia and other cognitive decline, persistent physical disability, fatal and non-fatal myocardial infarction, fatal and non-fatal stroke and cardiovascular death, fatal and non-fatal cancer, heart failure hospitalisation, atrial fibrillation, all-cause hospitalisation, need for permanent residential care, and quality of life.

## METHODS AND ANALYSIS

### Study design


For peer review only - http://bmjopen.bmj.com/site/about/guidelines.xhtml

STAREE is a double blind, randomised, placebo-controlled primary prevention trial. The WHO Trial Registration Data Set is shown in Appendix 2 and Appendix 3 details the study protocol.

**Setting, participant recruitment, screening, consent and follow up**

Recruitment is being conducted through general practices across metropolitan and regional/rural Australia with participating general practitioners (GPs) registered as STAREE co-investigators. General practice clinical database reviews create a list of potential participants aged ≥70 years without a history of clinical CVD, diabetes, dementia or other life-limiting conditions. Each GP screens the list for suitability and approves the sending of an invitation letter through the mail. Potential participants call the study centre and undergo an initial phone screening for trial inclusion and exclusion criteria. Those who are eligible are invited to take part in two baseline screening visits, either as a face-to-face visit at their usual GP clinic or as a remote phone visit with a trained study team member.

At the first baseline screening visit informed consent is obtained (Appendix 3) and then further eligibility assessments (described below) are completed. Demographic information, lifestyle factors, past medical history, concomitant medications, and other physical measurements (blood pressure, heart rate, height, weight, and waist circumference) are collected. Eligible participants then enter a 4-week run-in period and asked to take 1 tablet of run-in medication (matching placebo) each day to ascertain medication compliance. Placebo has been chosen for run in instead of active drug, so participants are not excluded on the basis of statin intolerance and the results are generalisable to the real-world use of statins. Eligible participants also attend their GP clinic for GP review of the trial inclusion and exclusion criteria, as well as any pathology testing.


For peer review only - http://bmjopen.bmj.com/site/about/guidelines.xhtml

At the second baseline screening visit after the run-in period, participants who achieve run-in medication compliance of >85% and have GP approval to participate in the trial, complete a cognitive battery to ascertain baseline cognitive performance (see Appendix 3).

See Appendix 3 for a detailed description of the schedule of visits and all tests performed.

## Inclusion and exclusion criteria

Individuals are deemed eligible if they; i) are aged ≥70 years; ii) live independently in the community; iii) are willing and able to provide informed consent and accept the study requirements and iv) do not have a history of clinical CVD events, diabetes or dementia. Exclusion criteria are a Modified Mini-Mental State Examination (3MS) score <78 on screening,  moderate or severe chronic kidney disease, moderate or severe liver disease, or serious inter-current illness likely to cause death within the next 5 years; total cholesterol >7.5 mmol/L; current participation in another interventional clinical trial; and an absolute contraindication to statin therapy, current use of statin therapy or other lipid lowering therapy for primary prevention and unwilling to stop therapy or current long term or permanent use of potent cytochrome P450 (CYP) 3A4 inhibitors.

## Randomisation to study treatment

Participants are randomly allocated to either atorvastatin or placebo treatment through a web-based system according to a randomisation schedule generated by an independent statistician. The allocation list was generated using randomly permuted blocks (of size either 2 or 4) with randomisation stratified by state (Victoria, Tasmania, South Australia, Western Australia,


For peer review only - http://bmjopen.bmj.com/site/about/guidelines.xhtml

Queensland and New South Wales) and age group (70-80 years, ≥80 years). Blocks ensure ongoing approximate balance of randomisation to statin and placebo within strata.

**Study medication**

Study medication comprises active treatment with atorvastatin tablets (20mg) or identical placebo. Participants are asked to take 1 tablet of study medication (20 mg of atorvastatin or placebo) per day for 4 weeks. They are then contacted by phone to assess study medication tolerability and instructed to increase the dose to two tablets per day (2x20 mg). Dose reduction of study medication to 1 tablet per day (20 mg) or temporary interruption is permitted throughout trial follow up to assist in managing tolerability and to reduce study medication discontinuation.

Atorvastatin has been chosen as one of the most commonly prescribed statins in Australia and many other countries with extensive GP experience in its use.[40] Importantly, relative to other statins, it is one of the most potent statins for reducing LDL-C with the dose of 40 mg expected to produce a 30-50% reduction.[45] The 2x20mg tablet formulation allows for dose adjustments and simplicity of supply of study product.

Medication is posted to participants at approximately 6 monthly intervals. Both active and placebo treatments are supplied by Ramsay Community Pharmacy and dispensed by an unblinded and independent pharmacist through a Monash University-affiliated central pharmacy dispensing centre.

**Study measures and post-randomisation follow up**


For peer review only - http://bmjopen.bmj.com/site/about/guidelines.xhtml

The trial activity schedule is summarized in Table 1. Participants are followed via phone or face to face visits every 6 months. At follow up visits, participants are asked about use of concomitant medications including commencement of new medications, adverse events and potential study endpoints. Supporting clinical information relating to any serious adverse events or endpoints is sourced from: a) medical record review; b) hospital records and discharge summaries; and c) linkage with national administrative death, hospitalisation and disease-specific registry datasets where possible.

A random subset of participants will also be offered the opportunity to attend STAREE sites with facilities for processing and storing biospecimens for collection of whole blood and/or urine samples, as outlined in Appendix 3 and 4.

**Adherence and retention strategies**

Adherence to medication is encouraged with multiple strategies including review of study medication use at follow up phone and face-to-face visits and through reminders included in quarterly participant newsletters and annual face-to-face or web-based study updates.

**Participant wellbeing**

Participants are advised to seek care from their usual treating GP (or other health care providers) for any medical condition arising during the study. Any tests or measurements performed as part of the trial trigger a notification to the participant's GP if outside the normal range or deemed clinically significant.

**Commencement of lipid lowering therapy**

For peer review only


For peer review only - http://bmjopen.bmj.com/site/about/guidelines.xhtml

Any participants who develop a recognised clinical indication (such as a myocardial infarction or stroke) for statin treatment can take open label statin as per usual practice by their treating clinician or clinical teams and will cease study medication whilst continuing in the study for observation (routine follow-up visits).

**Study conduct amendments in response to COVID-19**

As a result of government and workplace restrictions implemented during the COVID-19 pandemic, and to ensure safety of staff and participants, amendments to the trial protocol were implemented in 2020 to enable remote screening and follow up visits by phone instead of in-person visits. After easing of COVID-19 restrictions, participants were given the option of continuing with remote screening and follow up visits or face-to-face clinic visits depending on their risk profile, personal preference and location.

**Blinding and emergency unblinding**

All STAREE staff including the investigator team, management, administration, medical and research officers and students are blinded to treatment allocation through the randomisation procedure and study duration. All GP co-investigators and practice staff will be blinded to treatment allocation. All participants will be blinded to treatment allocation for the study duration. GP co-investigators are requested to not measure lipid levels in participants after randomisation, to minimise the risk of unmasking.

In the event of a clinical emergency, participants will be assumed to be on active treatment and the recommendation will be to cease study treatment if that were a requirement for statin use. The need for unblinding of study participants will be determined on a case-by-case basis after referral to a senior STAREE medical investigator who will discuss the clinical need for


For peer review only - http://bmjopen.bmj.com/site/about/guidelines.xhtml

unblinding with the requesting clinician and then authorise the request. The process of emergency unblinding will be overseen by the Monash University-affiliated, independent pharmacist who will have access to the randomisation code.

**Endpoint definitions and adjudication**

Ascertainment of primary endpoints will be performed by Endpoint Adjudication Committees (EAC) of expert clinicians. These Committees will adjudicate events related to possible myocardial infarction, stroke, dementia, disability, heart failure, and trajectory and mode of death. Each subcommittee will have a chair and a minimum of two other members. Two members will conduct adjudications for each event and discordant cases will be resolved via consensus of all committee members.

Disability free survival is defined as survival free of dementia or physical disability as derived from the endpoints of all-cause death, dementia, and persistent physical disability. It is a composite endpoint that has been used in recent clinical trials and enables an evaluation of the net benefit of statin by capturing both beneficial and adverse effects.[35]

Dementia is defined as a significant impairment in cognitive function (≥ 2 SD below population norms; Appendix 3) that interferes with independence in everyday activities[46] or as a clinical diagnosis based on the medical record. Persistent physical disability is defined as a loss of a basic activity of daily living assessed using the Katz Index of Independence in Activities of Daily Living (Katz ADL)[47] at two consecutive visits separated by a period of 6 months. A loss of an ADL (mobility, bathing, transferring, toileting, dressing, or feeding) will equate to a self-report of "a lot of difficulty", "unable to do this activity" or a requirement for help in performing the activity. If it is not possible to obtain a Katz ADL assessment,


For peer review only - http://bmjopen.bmj.com/site/about/guidelines.xhtml

notification of assessment for aged care services will initiate collection of relevant clinical records.

A major cardiovascular event is defined as the first occurrence of cardiovascular death or non-fatal myocardial infarction (MI) or non-fatal stroke. Cardiovascular death is defined as all deaths of cardiovascular cause including fatal MI, fatal stroke, sudden death and death due to other circulatory causes. MI is any ST and non-ST elevation event based on the fourth universal definition of MI.[48] Stroke is any ischaemic or non-ischaemic event defined by ICD-11 criteria[49] (not including transient ischaemic attack or subarachnoid haemorrhage and classified as disabling or non-disabling using the Modified Rankin Scale. Brain imaging will determine aetiology with ischaemic stroke further delineated according to the TOAST criteria.[50]

**Statistical considerations**

The number of participants needed in the trial was originally based on estimated event rates, using the ASPREE trial population which has similar demographic characteristics to the STAREE population, and on the magnitude of the treatment effect anticipated as a result of the LDL-C lowering effect of atorvastatin.[51]

**Modifications triggered by COVID-19**

The recruitment rate in Australia was impacted by the COVID-19 pandemic. In addition, STAREE was originally planned as an international endeavour with proposed funding and recruitment across multiple regions including Australia, however in late 2020 it became evident that the proposed funding for recruitment outside of Australia would not be secured. These developments were considered in July 2021 in finalisation of the trial design, reduction

For peer review only


For peer review only - http://bmjopen.bmj.com/site/about/guidelines.xhtml

in the sample size from an original target of n=18,000 and corresponding power calculation to allow for feasible completion of the trial in Australia alone.

**Sample size**

The sample size requirements are summarised in Table 1 for detecting relative risk as hazard ratios with a two-sided significance level of 5% and adopting a closed testing procedure of the two co-primary endpoints. The key assumptions of the power calculation are:

- Loss to follow-up will occur at a rate of 2% of study participants per annum (loss to follow-up includes those due to death and is expected to be minimised through good retention of participants and the ability to access medical records or perform record linkage for participants who cease in-person assessment). Hence an average of 6.0 years of follow-up time per participant is expected by the anticipated end of the trial in December 2025, at which time the required number of co-primary endpoints are expected to have accrued.
- An age structure of STAREE participants of 56%: 27%: 12%: 5% in the respective age bands 70-74 years: 75-79 years: 80-84 years: 85+ years. These proportions are based on recruitment of the initial approximately 7,500 participants to late 2020.
- With treatment adherence statins will reduce the rate of MACE by 25% and the rate of disability-free survival by 18%.
- Treatment cross-over will occur and impact the hazard ratio that will be observed in intention to treat analyses. In the first year of follow-up, we assume 8% of participants randomised to statin will cease taking their study medication. Thereafter we assume 3% of participants per annum in the statin group will cease taking study medication. In the first year of follow-up, we assume 1% of participants in the placebo group will commence open-label statin. Thereafter, we assume 3% of participants per annum in


For peer review only - http://bmjopen.bmj.com/site/about/guidelines.xhtml

the placebo group will commence statin therapy. Further, we assume that there will be no subsequent treatment "re-cross-overs”. It is assumed that the effects of statin use and non-use outside use of study medication is the same as the effects of randomised statin and placebo respectively.

- Rates of the co-primary endpoint death, dementia, or persistent physical disability (DFS) in the placebo group of 18.3 per 1000 person-years (70-74y), 33.9 per 1000 person-years (75-79y), 65.7 per 1000 person-years (80-84y), 115.4 per 1000 person-years (85+y) (See Appendix 5). To allow for a healthy participant effect at recruitment, it is assumed that these event rates will be observed from the start of the third year of an individuals' follow-up and that in the first and second year of follow-up the corresponding rates respectively will be zero and half of that of the third year of follow-up.
- Major cardiovascular event rates in the placebo group, according to age at randomisation, of 9.5 per 1000 person-years (70-74y), 11.5 per 1000 person-years (75-79y), 18.5 per 1000 person-years (80-84y), 24.5 per 1000 person-years (85+y).

Table 1: Sample size requirement and power justification for the STAREE trial's co-primary endpoints

| Co-primary endpoint | Effect size (relative reduction in risk for statin group versus placebo in intention to treat analysis based on treatment adherence assumptions) | Power | Required number of events | Required number of participants based on assumed event rates and follow-up |
|---|---|---|---|---|
| Major cardiovascular events (first occurrence of CV death, non-fatal MI or non-fatal stroke) | **20.2%** | **80.0%** | **619** | **9,631** |


For peer review only - http://bmjopen.bmj.com/site/about/guidelines.xhtml

| | | | | |
|---|---|---|---|---|
| Disability free survival (first occurrence of dementia, persistent physical disability or death) | **14.5%** | **83.1%** | **1,397** | **9,631** |

**Statistical analysis**

Baseline characteristics of the two treatment groups will be tabulated and imbalance defined as a 0.25 standard deviation difference in means (quantitative characteristics) or an odds ratio of 1.5 (binary characteristics). Given the large sample size, randomisation is anticipated to adequately balance baseline characteristics of participants in the two treatment groups. Hence unadjusted analyses will be considered primary. All primary comparisons between treatment arms will be on an intention-to-treat basis i.e., according to the group to which participants were randomised and without reference to their actual compliance with assigned treatment. Each of the co-primary endpoints will be analysed separately in time-to-event analyses. Event rates (time to first event within each endpoint definition) will be compared between groups using a hazard ratio and 95% confidence interval from a Cox proportional hazards regression model fitted to the endpoint, with censoring for individuals not experiencing an endpoint event at their most recent study visit, and a single covariate being an indicator of the group to which the individual was randomised, statin or placebo. The proportional hazards assumption will be tested for each model. Loss to follow-up will be considered a censoring event. This equates to an assumption that data is missing at random given the participant's treatment group and the timing of their loss to follow-up. The adequacy of this assumption will be checked in sensitivity analyses that will include both imputation approaches and adjustment for baseline covariates predictive of propensity for dropout.

A closed testing procedure will be used to allow for the multiple testing arising from two co-primary endpoints. This approach is based on the expectation that cardiovascular benefit will


For peer review only - http://bmjopen.bmj.com/site/about/guidelines.xhtml

be the main contributor to improved disability free survival and that a substantial effect of statins on the latter is unlikely in the absence of an effect on the former. First, major cardiovascular events will be tested at alpha=0.05 and, if the major cardiovascular events p-value is <0.05 then second, disability-free survival will be tested at alpha=0.05. If the major cardiovascular events p-value is not <0.05 than a p-value for disability-free survival will not be presented.

Secondary analyses of the co-primary endpoints will be performed using extended Cox proportional hazards regression models based on time(s) to any event within the endpoint definition (for relevant endpoints that can occur more than once). No statistical adjustment will be made for the multiple secondary endpoints and no p-values will be presented for the statin versus placebo comparison for these endpoints. The reporting of all secondary endpoint analyses will make clear whether either of the co-primary endpoints were statistically significant.

Subgroup analyses will be undertaken by sex (female, male), age (70-74, ≥75years), systolic and diastolic blood pressure levels (terciles), body mass index (<25, 25-29.9, 30+ kg/$m^2$), smoking status (never, past and current), 3MS score (above, below median), estimated GFR (<60, ≥60 mL/min per 1.73$m^2$), and LDL-C and HDL-C levels (terciles).

The impacts of COVID-19 will be investigated in sensitivity analyses that include examination of participant event rates by whether their randomisation occurred pre-, peri- or post-pandemic, and consider COVID-19 vaccination status (which will be an intercurrent event for those randomised pre-pandemic and a baseline covariate for those randomised post-


For peer review only - http://bmjopen.bmj.com/site/about/guidelines.xhtml

pandemic), following emerging guidance on analytical approaches and estimand definitions.[52]

**Data and Safety Monitoring Board (DSMB)**

An independent committee comprising experts in clinical trials, cardiology, and statistics are overseeing data accrual and safety. As outlined in the DSMB Charter (Appendix 6), the DSMB will monitor participant deaths, serious adverse events, endpoints, data quality and progress of the study. The DSMB will also review any interim analyses on unblinded data in accordance with stopping rules and will provide recommendations to the STAREE Executive Committee.

**Patient and public involvement**

STAREE has embedded mechanisms for ongoing participant and public involvement. These include a participant advisory committee, which reviewed study processes and documents, and a formal study that assessed participant preferences about the delivery of informed consent information. [53 54] Presentations to relevant community, social and professional groups have also offered the opportunity for public involvement.

During the trial, participants may provide feedback via access to a free phone line. Participants are also invited to annual trial updates and "question and answer" sessions. At the completion of the trial, participants will receive a summary of the study results and information about which treatment arm they were allocated to.

**Ethics and Dissemination**



For peer review only - http://bmjopen.bmj.com/site/about/guidelines.xhtml

STAREE is being conducted in accordance with the Declaration of Helsinki (1964, revised in 2008), the NHMRC Guidelines on Human Experimentation, the federal patient privacy (HIPAA) law and ICH-GCP guidelines and the International Conference of Harmonisation Guidelines for Good Clinical Practice. Institutional ethics approval has been obtained from the following:

Monash University Human Research Ethics Committee (Project ID 2787 and 21528).

Curtin University Human Research Ethics Committee (HR113/2015)

RACGP National Research and Evaluation Ethics Committee (14 - 017)

Tasmanian Health and Medical Research Ethics Committee (H0014918)

University of Newcastle Human Research Ethics Committee (H-2016-0266)

The trial is managed and co-ordinated by the STAREE steering committee (Appendix 1 which is responsible for developing and approving the protocol and operational plan. The trial central coordinating centre is based at the School of Public Health and Preventive Medicine, Monash University, 553 St Kilda Road, Melbourne 3004.

All research outputs will be disseminated to GP co-investigators and participants, published in peer-reviewed journals and presented at national and international conferences. Authorship of papers will be according to the criteria of the International Committee of Medical Journal Editors and all STAREE publications will list the lead authors and writing group, and the STAREE Investigator group.

On completion of the trial, and after publication of the primary and secondary outcomes of the study, requests for access to de-identified data (to be provided through a secure online environment) may be submitted to the researchers located at the School of Public Health and Preventive Medicine, Monash University, Melbourne, Australia.

For peer review only



For peer review only - http://bmjopen.bmj.com/site/about/guidelines.xhtml

## Trial status

Recruitment commenced in November 2015 and was expected to be completed in 2021. However, COVID-19 public health directives and work restrictions resulted in reduced access to general practices across Australia such that recruitment had to be extended for a further 18 months.

## DISCUSSION

There is continuing debate on the utility of statins for primary prevention in older people primarily due to the lack of RCT evidence in this population.[33] While no new RCTs of statins have reported since the conception and commencement of STAREE in 2015 and completion of HOPE-3 in 2016, new analyses of existing RCT data have added to the debate. Age specific analyses of pooled data from the HOPE 3 and JUPITER RCTs have suggested significant CVD benefits for all age groups above 65 years including a 26% risk reduction in CVD events in individuals aged 70 years and over.[37] A meta-analysis of efficacy and safety of individual participant data from 28 RCTs of statin therapy that included participants over 75 years indicated non-significant 16% and 8% reductions in major vascular events per 1.0 mml/L reduction in LDL-C amongst 70 to 75 and >75 year age groups, respectively, without known vascular disease.[36] However, no significant effects on vascular death, non-vascular death, cancer death or all-cause death were observed in either of these older age groups. Guidelines for statin prescription reflect this lack of robust trial evidence, with varying recommendations for apparently healthy older adults over 70 or 75 years, which have led to confusion amongst clinicians and consumers alike. In contrast, specific data exist for the use of aspirin and anti-hypertensive drugs in this population which has informed guideline


For peer review only - http://bmjopen.bmj.com/site/about/guidelines.xhtml

committees in making recommendations for intervention.[35 51 55] STAREE will provide new trial evidence to address this critical gap for statins.

STAREE is a large primary prevention trial that is assessing whether statins extend an independent and healthy lifespan. While STAREE captures the CVD endpoints that are the traditional primary outcomes of statin trials, we consider that a wider range of outcomes beyond major CVD events is important to evaluate the overall benefit of statins in an older population. To this end, our disability free survival outcome including detailed ascertainment of dementia using well validated measures of cognition, such as those used in the ASPREE trial [35] and advocated by others [33], will allow us to determine whether statins reduce major cardiovascular events and prolong survival free of dementia or physical disability; health outcomes that are highly valued by older people and the general population.

For peer review only


For peer review only - http://bmjopen.bmj.com/site/about/guidelines.xhtml

**Author contributions**

Conception and design of the STAREE trial. SZ, JJM, RW, LB, GCC, TT-JC, MRN, SJN, CMR, AT, ASW, SAW

Development of the protocol: SZ, AJC, SS, RW

Drafting of the manuscript: SZ, AJC, SS, RW

Review of the manuscript: All authors read and approved the final manuscript

**Funding statement**

STAREE is sponsored by Monash University and has received funding from the National Health and Medical Research Council (NHMRC APP1068146 and APP1161503) and the Heart Foundation of Australia (HF Stroke Prevention grant)**.** STAREE MIND and STAREE HEART have been awarded NHMRC grant funding (#2006611 and #1165440). The funders have had no role in the study design, collection, management, analysis and interpretation of data or in writing or submission of reports or publications.

For peer review only


For peer review only - http://bmjopen.bmj.com/site/about/guidelines.xhtml

## Competing Interests

SZ has received NHMRC and Australian Heart Foundation research funding as the principal investigator of the STAREE trial; and payment to the institution (Monash University) from Amgen Australia, AstraZeneca, Boehringer-Ingelheim, Eli Lilly Australia, Merck Sharp & Dohme Australia, Novo Nordisk, Sanofi, and Servier for consultancy work outside the submitted work.

IH has received research funding from NHMRC and Royal Australasian College of Physicians and honoraria for lectures or advisory board participation from Boehringer Ingelheim, Vifor and Eli Lilly.

JJM is supported by a NHMRC Leadership Fellowship (IG1173690).

MRN has served on a Novartis advisory board on lipid management in 2020.

CMR is supported through a NHMRC Principal Research Fellowship (APP 1136372).

SJN has received research support from AstraZeneca, New Amsterdam Pharma, Amgen, Anthera, Eli Lilly, Esperion, Novartis, Cerenis, The Medicines Company, Resverlogix, InfraReDx, Roche, Sanofi-Regeneron and LipoScience and is a consultant for AstraZeneca, Amarin, Akcea, Eli Lilly, Anthera, Omthera, Merck, Takeda, Resverlogix, Sanofi-Regeneron, CSL Behring, Esperion, Boehringer Ingelheim and Vaxxinity.

TC has received honoraria for lectures from Roche.

ASW has been a clinical trial investigator for Akcea, Amgen, Regeneron and Silence Therapeutics. He has served as a guideline chair and done consultancy work for the National Institute of Health and Care Excellence in the UK.

AT has received research support or honoraria for lectures, advisory board or data monitoring committee participation from Amgen, The Medicines Group, Merck, Novartis and Pfizer.

SW has received payment to attend an advisory meeting on dementia for Roche.

For peer review only


For peer review only - http://bmjopen.bmj.com/site/about/guidelines.xhtml

## References

1. Bongaarts J. Human population growth and the demographic transition. *Philos Trans R Soc Lond B Biol Sci* 2009;364(1532):2985-90. doi: 10.1098/rstb.2009.0137 [published Online First: 2009/09/23]
2. Christensen K, Doblhammer G, Rau R, et al. Ageing populations: the challenges ahead. *Lancet* 2009;374(9696):1196-208. doi: 10.1016/S0140-6736(09)61460-4 [published Online First: 2009/10/06]
3. Jacobsen L, Kent M, Lee M, et al. America's aging population. *Population Buletin* 2011;66(1)
4. Spiers GF, Kunonga TP, Beyer F, et al. Trends in health expectancies: a systematic review of international evidence. *BMJ Open* 2021;11(5):e045567. doi: 10.1136/bmjopen-2020-045567 [published Online First: 2021/05/27]
5. Welsh CE, Matthews FE, Jagger C. Trends in life expectancy and healthy life years at birth and age 65 in the UK, 2008-2016, and other countries of the EU28: An observational cross-sectional study. *Lancet Reg Health Eur* 2021;2:100023. doi: 10.1016/j.lanepe.2020.100023 [published Online First: 2021/04/20]
6. North BJ, Sinclair DA. The intersection between aging and cardiovascular disease. *Circ Res* 2012;110(8):1097-108. doi: 10.1161/CIRCRESAHA.111.246876 [published Online First: 2012/04/14]
7. van der Flier WM, Scheltens P. Epidemiology and risk factors of dementia. *J Neurol Neurosurg Psychiatry* 2005;76 Suppl 5:v2-7. doi: 10.1136/jnnp.2005.082867 [published Online First: 2005/11/18]
8. Gale CR, Cooper C, Sayer AA. Prevalence of frailty and disability: findings from the English Longitudinal Study of Ageing. *Age Ageing* 2015;44(1):162-5. doi: 10.1093/ageing/afu148 [published Online First: 2014/10/15]
9. Fong JH. Disability incidence and functional decline among older adults with major chronic diseases. *BMC Geriatr* 2019;19(1):323. doi: 10.1186/s12877-019-1348-z [published Online First: 2019/11/23]
10. Maresova P, Javanmardi E, Barakovic S, et al. Consequences of chronic diseases and other limitations associated with old age - a scoping review. *BMC Public Health* 2019;19(1):1431. doi: 10.1186/s12889-019-7762-5 [published Online First: 2019/11/05]
11. Quine S, Morrell S. Fear of loss of independence and nursing home admission in older Australians. *Health Soc Care Community* 2007;15(3):212-20. doi: 10.1111/j.1365-2524.2006.00675.x [published Online First: 2007/04/21]
12. Fried TR, Bradley EH, Williams CS, et al. Functional disability and health care expenditures for older persons. *Arch Intern Med* 2001;161(21):2602-7. doi: 10.1001/archinte.161.21.2602 [published Online First: 2001/12/26]
13. Wolff JL, Starfield B, Anderson G. Prevalence, expenditures, and complications of multiple chronic conditions in the elderly. *Arch Intern Med* 2002;162(20):2269-76. doi: 10.1001/archinte.162.20.2269 [published Online First: 2002/11/07]
14. El-Hayek YH, Wiley RE, Khoury CP, et al. Tip of the iceberg: Assessing the global socioeconomic costs of Alzheimer's Disease and related dementias and strategic implications for stakeholders. *J Alzheimers Dis* 2019;70(2):323-41. doi: 10.3233/JAD-190426 [published Online First: 2019/07/01]
15. Cholesterol Treatment Trialists C, Mihaylova B, Emberson J, et al. The effects of lowering LDL cholesterol with statin therapy in people at low risk of vascular disease: meta-analysis of individual data from 27 randomised trials. *Lancet*


For peer review only - http://bmjopen.bmj.com/site/about/guidelines.xhtml

2012;380(9841):581-90. doi: 10.1016/S0140-6736(12)60367-5 [published Online First: 2012/05/23]
16. Prospective Studies C, Lewington S, Whitlock G, et al. Blood cholesterol and vascular mortality by age, sex, and blood pressure: a meta-analysis of individual data from 61 prospective studies with 55,000 vascular deaths. *Lancet* 2007;370(9602):1829-39. doi: 10.1016/S0140-6736(07)61778-4 [published Online First: 2007/12/07]
17. Jonsson A, Sigvaldason H, Sigfusson N. Total cholesterol and mortality after age 80 years. *Lancet* 1997;350(9093):1778-9. doi: 10.1016/S0140-6736(05)63609-4 [published Online First: 1997/12/31]
18. Schatz IJ, Masaki K, Yano K, et al. Cholesterol and all-cause mortality in elderly people from the Honolulu Heart Program: a cohort study. *Lancet* 2001;358(9279):351-5. doi: 10.1016/S0140-6736(01)05553-2 [published Online First: 2001/08/15]
19. Volpato S, Leveille SG, Corti MC, et al. The value of serum albumin and high-density lipoprotein cholesterol in defining mortality risk in older persons with low serum cholesterol. *J Am Geriatr Soc* 2001;49(9):1142-7. doi: 10.1046/j.1532-5415.2001.49229.x [published Online First: 2001/09/18]
20. Schupf N, Costa R, Luchsinger J, et al. Relationship between plasma lipids and all-cause mortality in nondemented elderly. *J Am Geriatr Soc* 2005;53(2):219-26. doi: 10.1111/j.1532-5415.2005.53106.x [published Online First: 2005/01/28]
21. Weverling-Rijnsburger AW, Jonkers IJ, van Exel E, et al. High-density vs low-density lipoprotein cholesterol as the risk factor for coronary artery disease and stroke in old age. *Arch Intern Med* 2003;163(13):1549-54. doi: 10.1001/archinte.163.13.1549 [published Online First: 2003/07/16]
22. Foody JM, Wang Y, Kiefe CI, et al. Long-term prognostic importance of total cholesterol in elderly survivors of an acute myocardial infarction: the Cooperative Cardiovascular Pilot Project. *J Am Geriatr Soc* 2003;51(7):930-6. doi: 10.1046/j.1365-2389.2003.51305.x [published Online First: 2003/07/02]
23. Zingel R, Bohlken J, Riedel-Heller S, et al. Association between low-density lipoprotein cholesterol levels, statin use, and dementia in patients followed in German general practices. *J Alzheimers Dis* 2021;79(1):37-46. doi: 10.3233/JAD-201176 [published Online First: 2020/11/21]
24. Liang Y, Vetrano DL, Qiu C. Serum total cholesterol and risk of cardiovascular and non-cardiovascular mortality in old age: a population-based study. *BMC Geriatr* 2017;17(1):294. doi: 10.1186/s12877-017-0685-z [published Online First: 2017/12/29]
25. Pencina MJ, Navar AM, Wojdyla D, et al. Quantifying importance of major risk factors for coronary heart disease. *Circulation* 2019;139(13):1603-11. doi: 10.1161/CIRCULATIONAHA.117.031855 [published Online First: 2018/12/28]
26. Silverman MG, Ference BA, Im K, et al. Association between lowering LDL-C and cardiovascular risk reduction among different therapeutic interventions: A systematic review and meta-analysis. *JAMA* 2016;316(12):1289-97. doi: 10.1001/jama.2016.13985 [published Online First: 2016/09/28]
27. Greenwood J, Mason JC. Statins and the vascular endothelial inflammatory response. *Trends Immunol* 2007;28(2):88-98. doi: 10.1016/j.it.2006.12.003 [published Online First: 2007/01/02]
28. Zhou Z, Ofori-Asenso R, Curtis AJ, et al. Association of statin use with disability-free survival and cardiovascular disease among healthy older adults. *J Am Coll Cardiol* 2020;76(1):17-27. doi: 10.1016/j.jacc.2020.05.016 [published Online First: 2020/07/04]


For peer review only - http://bmjopen.bmj.com/site/about/guidelines.xhtml

29. Stancu C, Sima A. Statins: mechanism of action and effects. *J Cell Mol Med* 2001;5(4):378-87. doi: 10.1111/j.1582-4934.2001.tb00172.x [published Online First: 2002/06/18]
30. Menge T, Hartung HP, Stuve O. Statins--a cure-all for the brain? *Nat Rev Neurosci* 2005;6(4):325-31. doi: 10.1038/nrn1652 [published Online First: 2005/04/02]
31. Bagheri H, Ghasemi F, Barreto GE, et al. The effects of statins on microglial cells to protect against neurodegenerative disorders: A mechanistic review. *Biofactors* 2020;46(3):309-25. doi: 10.1002/biof.1597 [published Online First: 2019/12/18]
32. Gurwitz JH, Go AS, Fortmann SP. Statins for primary prevention in older adults: Uncertainty and the Need for More Evidence. *JAMA* 2016;316(19):1971-72. doi: 10.1001/jama.2016.15212 [published Online First: 2016/11/14]
33. Singh S, Zieman S, Go AS, et al. Statins for primary prevention in older adults-moving toward evidence-based decision-making. *J Am Geriatr Soc* 2018;66(11):2188-96. doi: 10.1111/jgs.15449 [published Online First: 2018/10/03]
34. Zoungas S, Curtis A, Tonkin A, et al. Statins in the elderly: an answered question? *Curr Opin Cardiol* 2014;29(4):372-80. doi: 10.1097/HCO.0000000000000082 [published Online First: 2014/05/29]
35. McNeil JJ, Woods RL, Nelson MR, et al. Effect of aspirin on disability-free survival in the healthy elderly. *N Engl J Med* 2018;379(16):1499-508. doi: 10.1056/NEJMoa1800722 [published Online First: 2018/09/18]
36. Cholesterol Treatment Trialists C. Efficacy and safety of statin therapy in older people: a meta-analysis of individual participant data from 28 randomised controlled trials. *Lancet* 2019;393(10170):407-15. doi: 10.1016/S0140-6736(18)31942-1 [published Online First: 2019/02/05]
37. Ridker PM, Lonn E, Paynter NP, et al. Primary prevention with statin therapy in the elderly: New meta-analyses from the contemporary JUPITER and HOPE-3 randomized trials. *Circulation* 2017;135(20):1979-81. doi: 10.1161/CIRCULATIONAHA.117.028271 [published Online First: 2017/04/08]
38. McGuinness B, O'Hare J, Craig D, et al. Cochrane review on 'Statins for the treatment of dementia'. *Int J Geriatr Psychiatry* 2013;28(2):119-26. doi: 10.1002/gps.3797 [published Online First: 2012/04/05]
39. Routledge PA, O'Mahony MS, Woodhouse KW. Adverse drug reactions in elderly patients. *Br J Clin Pharmacol* 2004;57(2):121-6. doi: 10.1046/j.1365-2125.2003.01875.x [published Online First: 2004/01/30]
40. Buettner C, Davis RB, Leveille SG, et al. Prevalence of musculoskeletal pain and statin use. *J Gen Intern Med* 2008;23(8):1182-6. doi: 10.1007/s11606-008-0636-7 [published Online First: 2008/05/02]
41. Collins R, Reith C, Emberson J, et al. Interpretation of the evidence for the efficacy and safety of statin therapy. *Lancet* 2016;388(10059):2532-61. doi: 10.1016/S0140-6736(16)31357-5 [published Online First: 2016/09/13]
42. Zhou Z, Albarqouni L, Curtis AJ, et al. The safety and tolerability of statin therapy in primary prevention in older adults: A systematic review and meta-analysis. *Drugs Aging* 2020;37(3):175-85. doi: 10.1007/s40266-019-00736-y [published Online First: 2020/01/11]
43. Pirmohamed M, James S, Meakin S, et al. Adverse drug reactions as cause of admission to hospital: prospective analysis of 18 820 patients. *BMJ* 2004;329(7456):15-9. doi: 10.1136/bmj.329.7456.15 [published Online First: 2004/07/03]
44. Brugts JJ, Yetgin T, Hoeks SE, et al. The benefits of statins in people without established cardiovascular disease but with cardiovascular risk factors: meta-analysis of


For peer review only - http://bmjopen.bmj.com/site/about/guidelines.xhtml

randomised controlled trials. *BMJ* 2009;338:b2376. doi: 10.1136/bmj.b2376 [published Online First: 2009/07/02]
45. Naci H, Brugts JJ, Fleurence R, et al. Dose-comparative effects of different statins on serum lipid levels: a network meta-analysis of 256,827 individuals in 181 randomized controlled trials. *Eur J Prev Cardiol* 2013;20(4):658-70. doi: 10.1177/2047487313483600 [published Online First: 2013/03/27]
46. American Psychiatric A. Neurocognitive Disorders. In *Diagnostic and statistical manual of mental disorders : DSM-5-TR*. Fifth edition, text revision. ed: Washington, DC : American Psychiatric Association Publishing 2022.
47. Katz S, Ford AB, Moskowitz RW, et al. Studies of illness in the aged. The index of ADL: A standardized measure of biological and psychosocial function. *JAMA* 1963;185:914-9. doi: 10.1001/jama.1963.03060120024016 [published Online First: 1963/09/21]
48. Thygesen K, Alpert JS, Jaffe AS, et al. Fourth universal definition of myocardial infarction (2018). *J Am Coll Cardiol* 2018;72(18):2231-64. doi: 10.1016/j.jacc.2018.08.1038 [published Online First: 2018/08/30]
49. Feigin V, Norrving B, Sudlow CLM, et al. Updated Criteria for Population-Based Stroke and Transient Ischemic Attack Incidence Studies for the 21st Century. *Stroke.* 2018;49(9):2248-2255. doi: 10.1161/STROKEAHA.118.022161.
50. Adams HP, Jr., Bendixen BH, Kappelle LJ, et al. Classification of subtype of acute ischemic stroke. Definitions for use in a multicenter clinical trial. TOAST. Trial of Org 10172 in Acute Stroke Treatment. *Stroke* 1993;24(1):35-41. doi: 10.1161/01.str.24.1.35 [published Online First: 1993/01/01]
51. McNeil JJ, Wolfe R, Woods RL, et al. Effect of aspirin on cardiovascular events and bleeding in the healthy elderly. *N Engl J Med* 2018;379(16):1509-18. doi: 10.1056/NEJMoa1805819 [published Online First: 2018/09/18]
52. Van Lancker K, Tarima S, Bartlett J, et al. Estimands and their Estimators for Clinical Trials Impacted by the COVID-19 Pandemic: A Report from the NISS Ingram Olkin Forum Series on Unplanned Clinical Trial Disruptions. Statistics in Biopharmaceutical Research, DOI: 10.1080/19466315.2022.2094459
53. O'Hare F, Flanagan Z, Nelson M, et al. Comparing two methods for delivering clinical trial informed consent information to older adults: singular versus stepped approach. *Clin Trials* 2018;15(6):610-15. doi: 10.1177/1740774518793377 [published Online First: 2018/08/04]
54. O'Hare F, Spark S, Flanagan Z, et al. Impact of informed consent content and length on recruitment of older adults into a community based primary prevention trial. *Contemp Clin Trials Commun* 2018;11:89-94. doi: 10.1016/j.conctc.2018.05.003 [published Online First: 2018/07/14]
55. Beckett NS, Peters R, Fletcher AE, et al. Treatment of hypertension in patients 80 years of age or older. *N Engl J Med* 2008;358(18):1887-98. doi: 10.1056/NEJMoa0801369 [published Online First: 2008/04/02]


For peer review only - http://bmjopen.bmj.com/site/about/guidelines.xhtml

**Appendices for protocol paper**

Appendix 1 STAREE investigators and sites

Appendix 2 WHO trial registration data set

Appendix 3 STAREE protocol V3.0: Part 1 and Part 2 (Appendices) – attached separately

Appendix 4 STAREE Biobank participant informed consent form – attached separately

Appendix 5 DFS event rate assumptions

Appendix 6 DSMB Charter

For peer review only

**Appendix 1 – STAREE investigators, sites and acknowledgements**

**The STAREE investigator group includes the following:**

**Principal Investigator:**

Prof. Sophia Zoungas MBBS (Hons) FRACP PhD

**Steering Committee:**

Prof. Sophia Zoungas (Chair), Prof. Lawrence Beilin, Ass. Prof. Trevor T-J Chong, Prof. Geoffrey C Cloud, Prof. John J McNeil, Prof. Mark R Nelson, Prof. Stephen J Nicholls, Prof. Christopher M Reid, Prof. Andrew Tonkin, Dr Stephanie A Ward, Prof. Anthony S Wierzbicki, Prof. Rory Wolfe

**Executive Committee:**

Prof. Sophia Zoungas (Chair), Dr Andrea J Curtis, Ass. Prof. Ingrid Hopper, Dr Alissia Kost, Prof. John J McNeil, Prof. Mark R Nelson, Prof. Christopher M Reid, Ass. Prof. Joanne Ryan, Dr Simone Spark, Prof. Rory Wolfe.

**Data Safety Monitoring Committee:**

Prof. John Simes (Chair), Prof. Graeme Hankey, Prof. A Mark Richards and Prof. Mark Woodward. Dr Alan Herschtal and Dr Thao Le (independent statisticians).

**Endpoint Adjudication Committees:**

**Death:**

Prof. Mark R Nelson (Chair), Prof. Danny Liew, Dr Vazhma Qaderi, Prof. Anthony S Wierzbicki.

**Dementia/Disability:**

Ass. Prof. Trevor T-J Chong (Chair), Dr Chris Moran, Dr Stephanie A Ward.

**Myocardial Infarction:**

Prof. Stephen J Nicholls (Chair), Ass. Prof. Dion Stub, Prof. Andrew Tonkin.

**Stroke:**

Prof. Geoffrey C Cloud (Chair), Prof.Craig Anderson, Dr Barry Moynihan, Prof. Michael O'Sullivan.

**Heart Failure:**

Ass. Prof. Ingrid Hopper (Chair), Prof. John Amerena, Prof. David Kaye.

**General Practice Advisory Committee:**

Prof. Mark R Nelson (Chair), Dr Gary Deed, Dr Alissia Kost.

**Operations Committee:**

Dr Simone Spark (Chair), Ms Lucy Boorn, Ms Sue Critchley, Dr Andrea J Curtis, Mr Zachary Flanagan, Mr Stephen Glanville, Dr Alissia Kost, Ms Kate Vaughan.

**Data Access and Publications Policy Committee:**

Prof. Sophia Zoungas, Dr Andrea J Curtis, Mr Zachary Flanagan, Prof. Christopher M Reid, Dr Simone Spark, Prof. Rory Wolfe.

**STAREE Regional Leaders:**

**Victoria and South Australia:**

For peer review only

Prof. Sophia Zoungas (Melbourne, Central Victoria, Northern Victoria, South West Victoria, Geelong, Gippsland, East Gippsland, Mildura, Adelaide, Mount Gambier)

**Tasmania:**

Prof. Mark R Nelson (Hobart, Launceston, Burnie)

**Western Australia:**

Prof. Christopher M Reid (Perth, Bunbury)

**Queensland:**

Dr Gary Deed (Brisbane, Gold Coast, Toowoomba, Sunshine Coast, Hervey Bay)

**New South Wales:**

Prof. Andrew Boyle (Sydney, Newcastle, Port Macquarie, Wollongong, North Coast, Tamworth)

**Sub studies**

STAREE-Heart Principal Investigator: Ass. Prof, Ingrid Hopper

STAREE-MIND Principal Investigator: Ass. Prof. Joanne Ryan

**Acknowledgements**

We thank all past and present STAREE staff.

We thank Professors Elsdon Storey and Robyn Langham for their past contributions to the STAREE trial Steering Committee.

A list of all current STAREE staff can be found at: https://www.monash.edu/medicine/staree/about-staree/our-team

For peer review only

**Appendix 2. WHO Trial Registration Data Set**

| | **Data Category** | **Information** |
|---|---|---|
| 1 | Primary Registry and Trial Identifying Number | ClinicalTrials.gov<br>NCT02099123 |
| 2 | Date of Registration in Primary Registry | 28 March 2014 |
| 3 | Secondary Identifying Numbers | |
| 4 | Source(s) of Monetary or Material Support | National Health and Medical Research Council of Australia<br>Heart Foundation (Australia) |
| 5 | Primary Sponsor | Monash University |
| 6 | Secondary Sponsor(s) | Not applicable |
| 7 | Contact for Public Queries | staree@monash.edu |
| 8 | Contact for Scientific Queries | Professor Sophia Zoungas (Principal Investigator)<br>sophia.zoungas@monash.edu<br>Phone: +61 (3) 9903 0711<br>Address: Level 5/99 Commercial Rd, Melbourne 3004 |
| 9 | Public Title | STAtin therapy for Reducing Events in the Elderly (STAREE) trial |
| 10 | Scientific Title | A randomised clinical trial of STAtin therapy for Reducing Events in the Elderly |
| 11 | Countries of Recruitment | Australia |
| 12 | Health Condition(s) or Problem(s) Studied | Prevention of cardiovascular disease, dementia and disability |
| 13 | Intervention(s) | Atorvastatin (2 x 20 mg/day) |
| | | Identical Placebo (2 tablets/day) |
| 14 | Key Inclusion and Exclusion Criteria | Inclusion criteria: Age ≥70 years, living independently in the community, willing and able to provide informed consent and accept the study requirements |
| | | Exclusion criteria: History of clinical cardiovascular events, diabetes or dementia; Modified Mini-Mental State Examination (3MS) score <78 on screening; moderate or severe chronic kidney disease; moderate or severe liver disease; serious inter-current illness likely to cause death within the next 5 years; total cholesterol >7.5 mmol/L; current participation in another interventional clinical trial; and an absolute contraindication to statin therapy; current use of statin therapy or other lipid lowering therapy for primary prevention and unwilling to stop therapy; or current long term or permanent use of potent cytochrome P450 (CYP) 3A4 inhibitors. |
| 15 | Study Type | Interventional |
| | | Allocation: randomised |
| | | Double blind |
| | | Primary purpose: Phase IV study |
| 16 | Date of First Enrolment | November 2015 |
| 17 | Sample Size | 9631 |
| 18 | Recruitment Status | Recruiting |

For peer review only

| | | |
|---|---|---|
| 19 | Primary Outcome(s) | i) a composite of cardiovascular death, non-fatal myocardial infarction, or non-fatal stroke; and<br>ii) a composite of all-cause death or dementia or development of persistent physical disability. |
| 20 | Key Secondary Outcomes | All cause death, dementia, other cognitive impairment, persistent physical disability, fatal and non-fatal myocardial infarction, fatal and non-fatal stroke, cardiovascular death, fatal and non-fatal cancer, heart failure hospitalisation, atrial fibrillation, all-cause hospitalisation, need for permanent residential care, quality of life |
| 21 | Ethics Review | Monash University Human Research Ethics Committee (Project ID 2787 and 21528)<br>Curtin University Human Research Ethics Committee (HR113/2015)<br>RACGP National Research and Evaluation Ethics Committee (14 - 017)<br>Tasmanian Health and Medical Research Ethics Committee (H0014918)<br>University of Newcastle Human Research Ethics Committee (H-2016-0266) |
| 22 | Completion date | Anticipated end 2025 |
| 23 | Summary Results | Not applicable |
| 24 | IPD sharing statement | Requests for access to de-identified data (to be provided through a secure online environment) may be submitted to the researchers located at the School of Public Health and Preventive Medicine, Monash University, Melbourne, Australia. |

For peer review only

**Appendix 5 Assumptions for STAREE disability free survival (DFS) event rates**

DFS event rates for STAREE were calculated based on the event rates for Australian participants in the ASPREE study.

ASPREE Australian participants – rates averaged over 4.7years of follow up (Ryan J, Storey E, Murray AM et al. Randomized placebo-controlled trial of the effects of aspirin on dementia and cognitive decline. Neurology. 2020 Jul 21;95(3):e320-e331)

70-74y: 3.42 per 1000 person-years
75-79y: 7.31 per 1000 person-years
80-84y: 16.00 per 1000 person-years
85+y: 21.37 per 1000 person-years

ASPREE Australian participants – rates in years 2-7 if zero events in first year of 4.7y of follow-up

70-74y: 4.39 per 1000 person-years
75-79y: 9.30 per 1000 person-years
80-84y: 20.24 per 1000 person-years
85+y: 27.52 per 1000 person-years

Assuming STAREE dementia process produces rates 33% higher

70-74y: 5.84 per 1000 person-years
75-79y: 12.37 per 1000 person-years
80-84y: 26.92 per 1000 person-years
85+y: 36.60 per 1000 person-years

<u>DFS rates based on assumed dementia rates above and</u> combining with assumed death and persistent physical disability rates as follows: (death+dementia+disability) less 10% to account for individuals experiencing more than one of the three types of event

70-74y: (10.0 + 5.84 + 4.5)*0.9 = 18.3 per 1000 person-years
75-79y: (17.7 + 12.37 + 7.6)*0.9 = 33.9 per 1000 person-years
80-84y: (33.6 + 26.92 + 12.5)*0.9 = 65.7 per 1000 person-years
85+y: (66.6 + 36.60 + 25)*0.9 = 115.4 per 1000 person-years

For peer review only

**Appendix 6**

**Data and Safety Monitoring Board Charter for A randomised clinical trial of STAtin therapy for Reducing Events in the Elderly (STAREE)**

**(Short title: STAREE DSMB Charter)**

**Charter approved by Monash University**

Signature: ________________________________ Date:____________

**Sponsor Signatory**: Professor Sophia Zoungas

**DSMB Approved:**

Signature: ________________________________ Date:____________

**DSMB Chair:**

For peer review only

## 1. Introduction

**Trial name:** Statins in Reducing Events in the Elderly (STAREE)

**Trial objectives:**

**Scope of Charter:** This charter outlines the composition and responsibilities of the STAREE DSMB.

## 2. DSMB responsibilities

The Data and Safety Monitoring Board (DSMB) will monitor participant safety, data quality and evaluate the progress of the study.

- Review the research protocol, informed consent documents and plans for data safety and monitoring;
- Review any proposed modifications to the trial prior to their implementation;
- Evaluate the progress of the trial, including periodic assessments of data quality and timeliness, recruitment, accrual and retention, participant risk versus benefit, performance of the trial sites, and other factors that can affect study outcomes;
- Consider factors external to the study when relevant information becomes available, such as scientific or therapeutic developments that may have an impact on the safety of the participants or the ethics of the trial;
- Review study performance, make recommendations and assist in the resolution of problems reported by the Principal Investigators;
- Make recommendations to the Principal Investigators concerning continuation, termination or other modifications of the trial based on the observed beneficial or adverse effects of the treatment under study;
- If appropriate, review interim analyses in accordance with stopping rules, which are clearly defined in advance of data analysis and have the approval of the DSMB; and
- Ensure the confidentiality of the study data and the results of monitoring.

## 3. Membership

The DSMB will consist of 4 members and 3 will constitute a quorum.

Membership consists of persons completely independent of the investigators who have no financial, scientific, or other conflict of interest with the trial. Written documentation attesting to absence of conflict of interest is required from each member.

Professor John Simes, University of Sydney, has been selected by the Principal Investigators to serve as the Chairperson and is responsible for overseeing the meetings and developing the agenda in consultation with the Principal Investigators. The Chair is the contact person for the DSMB. Monash University will provide the logistical management and support for the DSMB.

## 4. Organisation of meetings

At the first meeting the DSMB will discuss the protocol, suggested modifications and establish guidelines to study monitoring by the Board. In consultation with the Principal Investigator, the DSMB chairperson will prepare the agenda to address the review of study materials, modifications to the study protocol and informed consent document, initiation of the trial, appointment of a safety

For peer review only

officer, reporting of adverse events, statistical analysis plan including interim analysis and stopping rules and/or other matters as appropriate.

Meetings of the DSMB will be held annually for the first two years and 6 monthly thereafter. An emergency meeting of the DSMB may be called at any time by the Chairperson if participant safety questions or other unanticipated problems arise.

Meetings will be closed to the public because discussions may address confidential participant data. Meetings may be convened as conference calls as well as in person.

Meetings will consist of open and closed sessions. Discussion held in all sessions will be confidential.

At least one of the Principle Investigators and key members of the study team will attend open sessions. Open session discussion will focus on the conduct and progress of the study, including participant accrual, protocol compliance and problems encountered. Unblinded data will not be presented in the open sessions.

Closed sessions will be attended by the DSMB members. The study statistician may be present at the request of the DSMB. Any data by blinded study group and, as necessary, unblinded data, will be presented during the closed session. The DSMB can request review of unblinded data during the closed session. Investigators will still remain blinded.

Each meeting must include a recommendation to continue or terminate the study made by a formal DSMB majority or unanimous vote. Should the DSMB decide to issue a termination recommendation, the full vote of the DSMB is required. A recommendation to terminate the study may be made by the DSMB at any time by majority vote.

Following each DSMB meeting, the Chair will formally write to the STAREE Chief Investigators to advise them that the monitoring of the study's progress is up to date, and that the DSMB recommends study continuation or discontinuation (whichever applies).

**5. Documentation**

DSMB interim report templates will be prepared by the study staff, typically the statistician, to be reviewed by the DSMB members at the first meeting. Interim data reports generally consist of two parts;

- Part 1 – Open session report, and
- Part 2 – Closed session report

The format and content of the reports for both the open and closed sessions and plans for interim analyses will be finalised and approved by the DSMB, although changes throughout the trial may be requested.

The reports will list and summarise safety data and describe the status of the study. All meeting materials will be sent to the DSMB at least 10 to 14 days prior to the meeting by secure email.

5.1 Open Session reports

Open session reports will include:

- Site administrative reports describing –
    - Numbers of participants screened, enrolled, completed and withdrawn
    - Baseline characteristics of the study population

For peer review only

- General information on study status
- Blinded listings of adverse events and serious adverse events as requested by the DSMB
- Other information as requested by the DSMB on a continuing or one off basis

5.2 Closed Session reports

Closed session reports will include:

- Information described in Part 1 but by blinded treatment group (e.g. group A/ group B).
- Information on:
  - Study outcomes
  - Safety data
  - Efficacy data
  - Interim analyses

Closed session reports will be destroyed at the conclusion of the meeting. If meetings are held by telephone printed copies of the closed reports should be destroyed immediately following the meeting.

5.3 Additional reports

Mailings to the DSMB: As per the schedule (agreed by the DSMB) blinded safety data will be communicated to all or selected DSMB members

Access to Interim data: Access to the accumulating endpoint data will be limited to as small a group as possible, such as the independent statistician. Limiting the access of interim data to the DSMB members relieves the investigator of the burden of deciding whether it is ethical to continue to randomise individuals and helps protect the study from bias in the recruitment and/or evaluation.

5.4 Interim analyses

The DSMB will determine when interim analyses will take place. Interim analyses will be reviewed in closed sessions and may be presented in blinded group format (e.g. group A/ group B). The DSMB may also request review of the unblinded data.

Interim analyses of efficacy data will be performed only if they are planned and approved in advance and if stopping criteria are clearly defined.

**6. DSMB reports**

It is the responsibility of the Principal Investigators to distribute the DSMB recommendations to all co-investigators and to ensure that copies are submitted to all ethics committees associated with the study.

**7. Confidentiality**

All materials, discussions and proceedings of the DSMB are completely confidential. Members and other participants in DSMB meetings are expected to maintain confidentiality.

For peer review only

# A randomised clinical trial of STAtin therapy for Reducing Events in the Elderly (STAREE)

**PROTOCOL**

**Version 3.0**

**Protocol authors:**

Professor Sophia Zoungas, Dr Andrea Curtis, Dr Simone Spark, Professor Rory Wolfe

**Affiliation:**

School of Public Health and Preventive Medicine, Monash University, VIC, Australia

For peer review only

**Contact for public enquiries**

Phone: 1800 770 664

Email: staree@monash.edu

Fax: +61 (3) 9903 0737

**Contact for scientific enquiries**

Professor Sophia Zoungas (Principal Investigator)

sophia.zoungas@monash.edu

Phone: +61 (3) 9903 0711

Address: Level 5/99 Commercial Rd, Melbourne 3004

**Recruitment Status:** Recruiting



For peer review only - http://bmjopen.bmj.com/site/about/guidelines.xhtml

**Acronyms used in this protocol**:

**ABS**: Australian Bureau of Statistics

**ACAP**: Aged-care assessment dataset

**ACR**: Albumin to creatinine ratio

**ADR**: Adverse Drug Reaction

**ADCS-IADL**: Alzheimer's Disease Cooperative Study Instrumental Activities of Daily Living

**ADAS-Cog**: The Alzheimer's Disease Assessment Scale - Cognition

**AE**: Adverse events

**ASPREE**: ASPirin in Reducing Events in the Elderly

**CAM**: Confusion Assessment Method

**CES-D-10**: Centre for Epidemiologic Studies Depression Scale – 10 items

**COWAT**: Controlled Oral Word Association Test

**CRF**: Case Report Form

**DMC**: Data Management Committee

**DOHA**: Department of Health and Ageing

**DSMB**: Data Safety and Monitoring Board

**EAC**: End-point Adjudication Committee

**GCP**: Good Clinical Practice

**HVLT-R**: Hopkins Verbal Learning Test – Revised

**HDL**: High Density Lipoprotein

**HIPAA**: Health Insurance Portability and Accountability Act of 1996

**IDL**: Intermediate Density Lipoprotein

**IPAQ-E**: International Physical Activity Questionnaire modified for the elderly

**LDL**: Low Density Lipoprotein

**MI**: Myocardial Infarction

**NCD** : Neurocognitive Disorder

**SAE**: Serious Adverse Event

**SDMT**: Symbol Digit Modalities Test

**SOP**: Standard Operating Procedure

**STAREE**: STAtin therapy for Reducing Events in the Elderly

**3MS**: Modified Mini-Mental State test

**TICS-M**: Telephone Interview of Cognitive Status – modified version

**VLDL**: Very Low Density Lipoprotein

For peer review only


For peer review only - http://bmjopen.bmj.com/site/about/guidelines.xhtml

**REVISIONS**

| **No.** | **Changes** |
|---|---|
| **Version 1.2.2** | Original approved version |
| **Version 1.3** | Changes to Study Activity Schedule including addition of cognitive tests, CESD10, and biobank sample collection |
| **Version 1.3.1** | SF-12 changed to SF-36; Biobank Steering Committee added to Governance structure |
| **Version 1.3.2** | Study medication: change to post out details and addition of medication compliance questionnaire |
| **Version 1.3.3** | Addition of International Steering Committee; Updated exclusion criteria regarding cytochrome P450 3A4 inhibitors; Change in biobank study samples collected; |
| **Version 2.0** | Updates to align protocol with SPIRIT 2013 statement recommendations |
| | Updates and clarification of primary and secondary endpoints; including change in dementia definition from DSM IV to DSM V |
| | Updated exclusion criteria regarding cytochrome P450 3A4 inhibitors |
| **Version 3.0** | Response to COVID-19 – schedule update |
| | Changes to power calculations, sample size and follow up period |

For peer review only



For peer review only - http://bmjopen.bmj.com/site/about/guidelines.xhtml

Contents

Contents ... 4
1. Executive Summary ... 8
2. Introduction ... 9
2.1 Rationale ... 9
2.2 Statins ... 10
2.3 Other health outcomes ... 12
2.4 Statins in the Elderly: potential risks ... 12
2.4.1 Myopathy ... 12
2.4.2 Diabetes ... 13
2.4.3 Cancer ... 13
2.4.4 Cognitive impairment ... 13
2.4.5 Liver Injury ... 13
2.5 Utilisation ... 14
2.6 Implications of Guidelines on Treatment of Cholesterol in the Elderly ... 14
2.7 Summary of rationale ... 16
3. Study Objectives ... 17
3.1 Primary objectives ... 17
3.2 Secondary objectives ... 17
3.3 Tertiary objectives ... 17
4. Hypotheses ... 18
4.1 Primary null hypotheses ... 18
4.2 Primary alternative hypotheses ... 18
5. Study Design ... 19
5.1 Trial Design ... 19
5.2 Study Population ... 19
5.2.1 General considerations ... 19
5.2.2 Inclusion criteria ... 19
5.2.3 Exclusion criteria ... 19
5.2.4 Rescreening or reassessment ... 20
5.3 Study Intervention and Choice of Comparator ... 20
5.3.1 Intervention ... 20
5.3.2 Rationale for choice of atorvastatin ... 20
5.4 Participant recruitment ... 21
5.4.1 Recruitment ... 21

For peer review only


For peer review only - http://bmjopen.bmj.com/site/about/guidelines.xhtml

5.4.2 Setting ....................................................................................................21
5.4.3 Community participants ...................................................................................22
5.4.4 STAREE hub locations.......................................................................................22
5.5 Participant timeline and schedule of assessments..................................................22
5.6 Baseline Screening Phase.........................................................................................22
5.6.1 Screening call ......................................................................................................22
5.6.2 Lifestyle profile and screening visits (Baseline visit 1)..........................................26
5.6.3 Post screening GP/medical visit (Run-in phase between Baseline visits 1 and 2) .26
5.6.4 Post screening visit study activity (Run-in phase between Baseline visits 1 and 2) ....................................................................................................26
5.6.5 Determinations of Eligibility and Assessments visit (Baseline Visit 2)...................26
5.7 Randomisation and followup phase ..........................................................................27
5.7.1 Randomisation procedure ....................................................................................27
5.7.2 Post randomisation procedure .............................................................................27
5.7.3 Blinding ................................................................................................................28
5.7.4 Annual Follow up visit..........................................................................................28
5.7.5 Phone Contact......................................................................................................28
5.7.6 Other Actions .......................................................................................................29
5.8 Economic Analysis......................................................................................................29
6. Study Endpoints ..........................................................................................................29
6.1 Primary endpoints.......................................................................................................29
6.2 Secondary endpoints ..................................................................................................30
6.3 Endpoint ascertainment .............................................................................................30
6.4 Endpoint adjudication process ...................................................................................31
7. Sub-studies and ancillary studies..................................................................................31
7.1 STAREE-HEART ...........................................................................................................31
7.3 STAREE Healthy Ageing Biobank.................................................................................31
8. 8. Contingency plans for participant wellbeing ..............................................................32
8.1 Commencement of regular lipid lowering agents .......................................................32
8.2 Development of an indication for statins ....................................................................32
8.3 Post-trial care ..............................................................................................................32
9. Levels of participant involvement....................................................................................33
9.1 Levels of participant involvement................................................................................33
9.2 Withdrawal...................................................................................................................34
10. Study Medication and Supplies ....................................................................................35
10.1 Study treatments .......................................................................................................35


For peer review only - http://bmjopen.bmj.com/site/about/guidelines.xhtml

10.2 Administration of study medication ....35
10.3 Dispensing and randomisation ....35
10.4 Study medication compliance....36
10.5 Study medication storage ....36
10.6 Study medication accountability and dispensing logs....37
10.7 Study medication disposal ....37
10.8 Overdose of study medication....38
11. Concomitant Therapy ....38
11.1 Prescription medications ....38
12. Sample Size and Power calculations....39
13. Safety Monitoring and Reporting ....43
13.1 Introduction ....43
13.2 Safety Monitoring ....43
13.2.2 Adverse events (AE) ....43
13.2.3 Serious adverse events (SAE)....43
13.2.4 Suspected Unexpected Serious Adverse Events (SUSARs) ....44
13.3 Safety reporting ....45
13.4 Adverse Events of Interest....45
13.4.1 New diagnosis of diabetes ....45
13.4.2 New diagnosis of myopathy....45
13.4.3 Liver impairment....46
14. Adherence to Ethical, Regulatory and Administrative Considerations ....47
14.1 Ethical considerations....47
14.1.1 General....47
14.1.2 Ethics Committee Approval ....47
14.1.3 Information for participants ....47
14.1.4 Informed consent....47
14.1.5 Protocol Amendments....47
14.1.6 Confidentiality....48
14.2 Regulatory considerations ....48
14.2.1 Financing....48
14.2.2 Trial registration....48
14.2.3 Declaration of interest ....48
14.3 Indemnity....48
14.4 Data Access ....49
14.5 Governance....49


For peer review only - http://bmjopen.bmj.com/site/about/guidelines.xhtml

14.6 Administrative Organisation ........................................................................50
14.6.1 Individual and committee responsibilities........................................................50
14. Data Management and Training ........................................................................54
14.1 Case report forms ........................................................................54
14.2 Data Management ........................................................................54
14.3 Data analysis ........................................................................55
14.4 Training ........................................................................56
14.4.1 Research Support (Administrative) Officers ........................................................56
14.4.2 GP Liaison Officers ........................................................................56
14.4.3 Research Field Officers ........................................................................56
15. Study Monitoring and Auditing........................................................................57
16. Dissemination Plan........................................................................58
17. References ........................................................................59

For peer review only



For peer review only - http://bmjopen.bmj.com/site/about/guidelines.xhtml

## 1. Executive Summary

This protocol describes the background, rationale, study design, processes and governance arrangements for the Statins in Reducing Events in the Elderly (STAREE) trial and its sub-studies.

STAREE is a double blind, randomised placebo-controlled parallel group trial investigating whether statin therapy can prolong good health and maintain independence amongst older people. STAREE will recruit and randomise 9,631 Australian participants aged 70 years and over who are free from cardiovascular disease, diabetes and dementia and living independently in the community.

The STAREE trial will compare the effects of atorvastatin (2 x 20 mg/day) or identical placebo on two co-primary outcomes, a composite of all cause death, dementia or disability and a composite of cardiovascular death, non-fatal myocardial infarction or non-fatal stroke. Secondary outcomes include all cause death, cardiovascular death, dementia, other cognitive impairment, disability, all cause hospitalisation, need for permanent residential care, fatal and non-fatal myocardial infarction, fatal and non-fatal stroke (thromboembolic and haemorrhagic), fatal and non-fatal cancer, heart failure and quality of life. The cost effectiveness of statin therapy for primary prevention in people aged 70 years and over will also be assessed.

For peer review only


For peer review only - http://bmjopen.bmj.com/site/about/guidelines.xhtml

## 2. Introduction

### 2.1 Rationale

**Ageing Population**

The developed world is experiencing a rapid demographic transition towards an older population.[1] Increasing survival amongst older age groups is a major contributor to this shift, with almost twice as many older people surviving into their eighties and nineties compared to fifty years ago.[2]

In the US, the proportion of the population aged 65 and over is projected to increase from 40 million in 2009 to 89 million in 2050 with 19 million (20%) in the oldest age group (85+) by 2050.[3] Similar trends are anticipated in other developed nations such as Australia, Canada and the United Kingdom.[2]

As age advances quality of life is increasingly affected by frailty, cognitive decline/dementia and the consequences of a variety of chronic diseases including cardiovascular disease, arthritis, osteoporosis and malignancy. Although preventive therapies have reduced the burden of chronic diseases at younger ages there is limited evidence that these advances have improved the health of older people.[2] As a result, the majority of older individuals ultimately progress to residential care. In Australia, at 75 years of age the remaining lifetime need for residential nursing care is estimated at 72% for women and 53% for men, at a cost of approximately $7 billion Australian dollars per annum.[4]

The gains that have been made in increasing quantity of life have not been accompanied by similar gains in quality of life, for the additional later years of life lived. The critical question is therefore one of the balance of any benefits and harms of preventive therapies for older people.

One promising but inadequately tested preventive approach to extending life and maintaining independence in the elderly is statin therapy. In people with existing cardiovascular disease (secondary prevention), the benefits of statin therapy are clear with the risks of cardiovascular events reduced across all ages. In people without cardiovascular disease (primary prevention), the benefits of taking a statin are not clear for older people. Most national and international guidelines have not been able to make strong recommendations to guide prescribing for older people and any recommendations have generally been informed by trials of statins in younger people (<70 years) or trials of statins that have not studied the range of outcomes important to older people.[5]

**Cholesterol and Clinical Outcomes in the Elderly**

While high levels of total cholesterol or LDL cholesterol are known to increase the risk of mortality among young and middle-aged populations, the relationship between total cholesterol or LDL cholesterol and all-cause or cause-specific death in older populations is not consistent. Some observational studies have reported no association or a negative association between total cholesterol or LDL cholesterol levels and all-cause death[6-11] whilst others have reported a positive linear association between total cholesterol and LDL cholesterol levels and cardiovascular death.[12] One explanation for the discrepancy is “reverse causality,” whereby unquantified comorbid disease may be causing both lower


For peer review only - http://bmjopen.bmj.com/site/about/guidelines.xhtml

total cholesterol or LDL cholesterol levels and an increased risk of death, thereby creating an apparent lack of association or negative association.

Given the uncertainty around optimal total and LDL cholesterol levels in the elderly, the decision as to whether statins should be initiated or even continued must be guided by randomised controlled trials.

## 2.2 Statins

### Pharmacological effects

Statins (HMG-CoA reductase inhibitors) are a class of drug with the potential to delay the onset of several causes of incapacity in the elderly. Their best-recognised pharmacological action is to inhibit the mevalonate pathway and biosynthesis of cholesterol and isoprenoids by binding to HMG-CoA reductase and displacing the natural substrate HMG-CoA. This interaction prevents HMG-CoA reductase from attaining a functional conformation, stops the conversion of HMG-CoA to l-mevalonate and produces a reduction in intracellular cholesterol.[13] LDL-receptor gene expression and formation on the hepatocyte cell surface are then induced. The result is an increase in LDL cholesterol extraction (and its precursors, IDL and VLDL) from the blood and decrease in circulating LDL cholesterol levels (by 18-55%).[13] Beneficial effects on other lipid parameters have also been reported including increased HDL cholesterol (by 5-15%),[14] decreased levels of atherogenic lipoproteins, [15] decreased triglyceride concentrations (7-30%) and decreased synthesis and secretion of triglyceride-rich lipoproteins[15] as well as reduced susceptibility of LDL cholesterol to oxidation.[13]

The reduction in circulating lipid levels underpins the ability of statins to reduce the incidence of myocardial infarction and stroke and their long-term sequelae (cardiac failure, disability, cognitive decline). Statins also exert a number of pleiotropic effects including anti-oxidative, anti-thrombotic and a moderately powerful anti-inflammatory action.[16] The pleiotropic effects of statins have been attributed to the inhibition of isoprenoid metabolite formation (geranylgeranyl pyrophosphate and farnesyl pyrophosphate).[13] These metabolites are intermediates in the post-translational formation of several cell-signalling proteins that control multiple cell functions including maintenance of cell shape, motility, factor secretion, differentiation, and proliferation thus explaining improvements in endothelial function, vascular inflammation and oxidation, and atherosclerotic plaque stability.[13]

### Clinical effects on Major Vascular Events and Death

The impact of statin therapy on cardiovascular events has been studied in primary (mostly high risk) and secondary prevention settings. A recent meta-analysis of pooled individual participant data from 28 trials of statin versus control/placebo including participants of ages 55 years and over has reported a 21% reduction in risk of major vascular events per 1 mmol/L reduction in LDL cholesterol, attributed to reduced risks of major coronary events, major stroke events and vascular death.[17]

In an analysis of mixed populations (both primary and secondary prevention), there was a trend for decreasing reductions in major vascular events with increasing age. Analyses by age group indicated risk reductions of major vascular events of 19% and 13% per 1 mmol/L reduction in LDL cholesterol for participants aged >70 to ≤75 and >75 years respectively.


For peer review only - http://bmjopen.bmj.com/site/about/guidelines.xhtml

This compared to a risk reduction of 25% for participants ≤55 years. [17] The risk of all-cause death was also reduced, albeit to a much lesser extent (9% per 1 mmol/L reduction in LDL cholesterol). No effect on non-vascular deaths was observed. Of note, participants were predominantly high risk, middle-aged men with high baseline LDL cholesterol levels (mean weighted LDL cholesterol level 3.7 mmol/L) or known cardiovascular disease.[17]

In those with cardiovascular disease (secondary prevention), the benefits of statin were clear with risks of major vascular events and all-cause death reduced in people of all ages.[18] Moreover, the number needed to treat was sufficiently low to justify treatment (28 patients would need to be treated for 5 years to prevent one death).[19]

In analyses limited to primary prevention populations, there was a significant trend toward smaller proportional risk reductions as age increased. (Figure 1) Analyses by age group indicated risk reductions of major vascular events of 16% and 8% per 1 mmol/L reduction in LDL cholesterol for participants aged >70 to ≤75 and >75 years respectively. This compared to a risk reduction of 32% for participants ≤55 years.[17] No data was presented on the risk of all-cause death in older people without cardiovascular disease.

**Figure 1: Effects of statin therapy on major vascular events (MVE) per mmol/L reduction in LDL cholesterol by age at randomisation in participants without vascular disease.** [17]

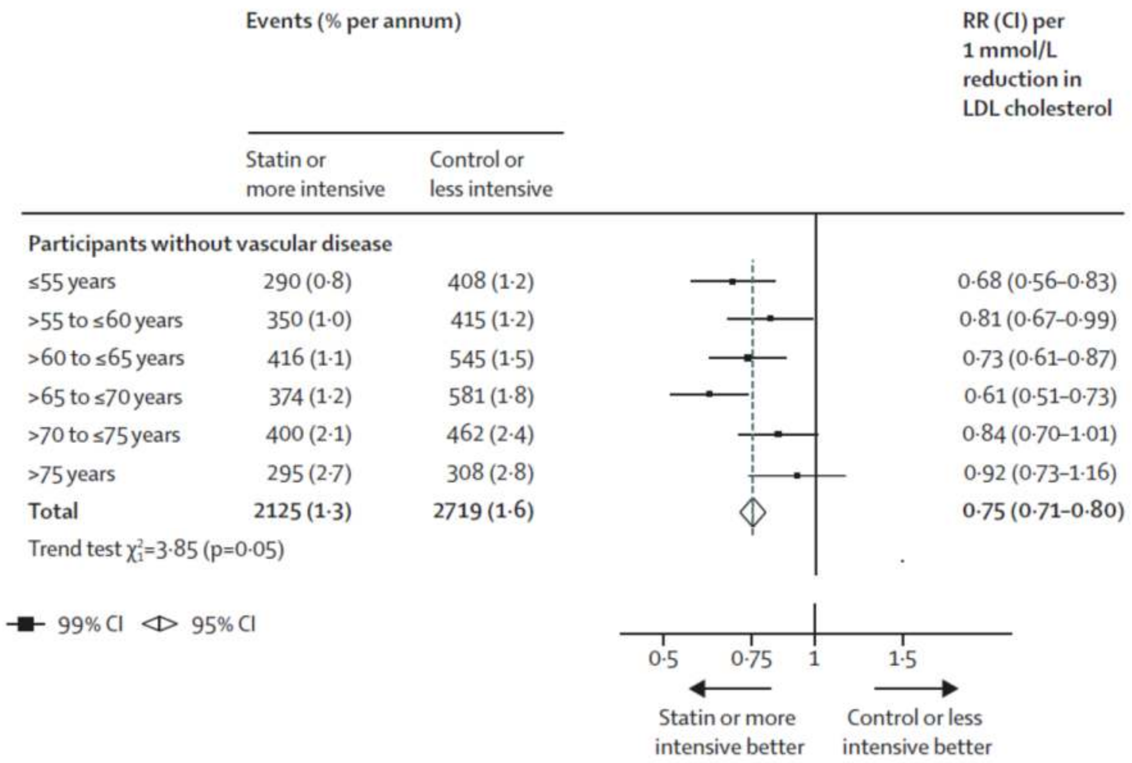


## Post hoc subgroup analyses of older participants in statin trials

Pravastatin in the PROspective Study of Pravastatin in the Elderly at Risk (PROSPER) trial, which followed 3,239 people aged 70 years or older for on average 3.2 years, did not significantly reduce cardiovascular events.[20] Pravastatin in the Antihypertensive and Lipid-Lowering Treatment to Prevent Heart Attack Trial (ALLHAT-LLT) trial which followed 2,867


For peer review only - http://bmjopen.bmj.com/site/about/guidelines.xhtml

people 65 years or older for over 4.5 years, did not significantly reduce cardiovascular events or mortality.[21] Rosuvastatin in the Justification for the Use of Statins in Prevention: an Intervention Trial Evaluating Rosuvastatin (JUPITER) trial, which followed 5695 people aged 70 years or older for on average 2 years, significantly reduced cardiovascular events (RRR 39% 95%CI 14 to 57%) but not mortality.[22] Rosuvastatin in the Heart Outcomes Prevention Evaluation (HOPE 3) Trial, which followed 3086 people aged 70 years or older for on average 4.6 years, did not significantly reduce cardiovascular events.[22] A pooling of data from the last 2 trials (JUPITER and HOPE 3) concluded that statins reduced cardiovascular events in those people aged 65-70 years (RRR 49% 95%CI 31 to 62%) and >70 years (RRR 26% 95%CI 9 to 39%).[23]

This demonstrates the degree of uncertainty about the effects of statins in the older age groups. Given the potential for widespread use of statins amongst older people, the limited data emphasises the need for more certainty about the scale of any likely benefit of this class of drugs.

### 2.3 Other health outcomes

Physical and cognitive function, independence, and health-related quality of life are important health outcomes for older people. These outcomes have not been comprehensively evaluated in previous statin trials.

Statins have been proposed to attenuate long term decline in physical and cognitive function and to preserve independent living by reducing the morbidity associated with coronary and cerebrovascular disease (including subclinical disease) in the elderly.[3]

### 2.4 Statins in the Elderly: potential risks

The likelihood of adverse effects of drug therapy increases in older people.[24] Much of the knowledge of specific adverse effects comes from spontaneous reports or from trials predominantly studying middle aged people. As with efficacy, the burden of statin adverse effects in older individuals has not been well studied. As a result, it is still not possible to know the balance of risks and benefits associated with the use of these drugs in this age-group.[25]

Amongst the elderly, the issues most likely to influence the risk/benefit balance are:

#### 2.4.1 Myopathy

All statins have been reported to cause myopathy with the severity ranging from asymptomatic increases in creatinine kinase to muscle aches and weakness to fatal rhabdomyolysis.[26] The precise mechanisms are not well-understood. Of note, these myopathies are dose dependent and may occur even after therapy has been tolerated for up to 1 year.[27] The estimated incidence of statin myopathy in trials has ranged from 1.4 to 5% with the lowest rates reported for atorvastatin at low or high dosage.[27,28] Older age, female gender and concomitant use of inhibitors of cytochrome P450 3A4 are recognised risk factors for the development of statin myopathy.[29] There may also be a higher risk of disability in older adults using statins due to age-related sarcopaenia.[30]


For peer review only - http://bmjopen.bmj.com/site/about/guidelines.xhtml

### 2.4.2 Diabetes

In a recently published meta-analysis of statin trials, statin use was associated with a 9% increased risk for incident diabetes with little heterogeneity between trials.[31] Meta-regression indicated that the risk of diabetes was highest in trials of older participants.[31] In light of this data, in 2012 the FDA modified the labelling of statins to include a warning for the potential risk of increased blood glucose levels and new type 2 diabetes.[32]

### 2.4.3 Cancer

One trial (PROSPER) reported an increased risk of cancer and cancer mortality with statin therapy among men and women older than 70 years.[20] However, this effect was not observed in other statin trials. A meta-analysis of all statin trials suggested no overall increased risk of cancer or cancer mortality over an average follow up of 4.8 years, albeit in a mostly middle-aged population.[33] Nonetheless, more data on the effects of statin therapy on risk of cancer in elderly patients is required and further follow up studies have been called for in such patients.[25] Longer follow up would determine whether new cancer events could occur with time. This is especially important if statin therapy is to be advocated for primary prevention.

### 2.4.4 Cognitive impairment

The FDA recently reported a review of the US spontaneous reporting database (AERS), the published medical literature (case reports and observational studies), and randomised clinical trials to evaluate the effect of statin therapy on cognition.[32] The post-marketing adverse event reports generally described ill-defined memory loss that was reversible upon discontinuation of statin therapy. Most reports were from individuals over the age of 50 years. Time to onset of the impairment was highly variable, ranging from one day to years after statin exposure. The review did not find an association between cognitive impairment and any specific statin, any specific age group, any particular statin dose or concomitant medication use. Furthermore, the cognitive impairment did not appear to be associated with fixed or progressive dementia, such as Alzheimer's disease. Nonetheless, in 2012 the FDA modified the labelling of statins to include a warning for the potential risk of cognitive effects such as memory loss and confusion.[32] A subsequent meta-analysis of data from 25 randomised trials reported no statistically significant effect of statins on a crude overall measure of cognition across all cognitive domains.[34] This analysis included 12 trials undertaken exclusively in younger populations (< 65 years) where subtle effects may not be measurable and only 4 trials that had more than 1-year follow up. Indeed, this meta-analysis could not address either long-term safety or efficacy particularly in older populations, largely because few such trials exist.

### 2.4.5 Liver Injury

Statins have been associated with mild elevations in liver enzymes in approximately 0.5-2.0% of people, particularly within the first 12 weeks of therapy being initiated.[35] Meta-analysis of trials has demonstrated a 50% increased risk of elevations in liver transaminase compared to placebo or control.[36] The response appeared dose dependent. However, the elevations were usually transient, rarely associated with symptoms and deemed unlikely to be clinically relevant.

Serious statin induced liver injury was rare. Post marketing reviews of statins conducted by the FDA between 2000 and 2009 indicated fewer than 2 cases of statin associated serious


For peer review only - http://bmjopen.bmj.com/site/about/guidelines.xhtml

liver injury per one-million patient years reported to the Adverse Event Reporting System.[37] Furthermore, although the use of statins has been rising over this period, no detectable increase in the annual rates of fatal or severe liver injury cases possibly or probably causally associated with statin use has been observed.[37]
Whether the risk of statin induced liver damage is increased in the elderly compared to younger people is currently unknown.

### 2.5 Utilisation

Statins are one of the most frequently dispensed drugs.[38] In the financial year ending 30 June 2012, over 10 million prescriptions for atorvastatin and 6 million prescriptions for rosuvastatin were dispensed in Australia.[39] In the period from April 2005 to March 2010, 86% of statin initiations came from general practitioners (GPs).[40] In 2011, the British Heart Foundation reported that 1 million statin prescriptions were provided each week in the United Kingdom.[41]

At present statin use in the elderly is lower than expected given the high absolute risk of this population. A UK survey of 41,250 GP medical records, reported that 23% of patients with no history of cardiovascular disease and aged over 75 were prescribed statin therapy compared to 29% in the 70-74 age group.[42] Amongst patients ≥75 years, for every 5 year increment in age the odds of being prescribed a statin decreased compared to the 40-44 year age group. One US study reported that 22% of people aged 80 years or older (in a primary prevention setting) were prescribed a statin[43] and another that those aged ≥70 years were less likely to use a statin than those aged 65-69 years (OR 0.57, 95% CI 0.39-1.09).[44] In an Australian survey of people aged 50 years and older, approximately 40% of respondents aged ≥75 years reported taking a statin.[45]

### 2.6 Implications of Guidelines on Treatment of Cholesterol in the Elderly

The potential for greater use of statin therapy amongst older people is illustrated by Figure 2 a) and b). This figure provides an approximation of the proportion of each age group where the estimated absolute cardiovascular risk exceeds a 15% likelihood of a new vascular event in the next five years (commonly regarded as the fundamental indication for statin therapy).[46] It is based on the cardiovascular death rate multiplied by four; in the WOSCOPS trial there were four incident cardiovascular events for each fatal event.[47] On this basis, most males over the age of 75 years and a high percentage of females would qualify for consideration of statin therapy.

Greater use of statins is also likely to be driven by the recent changes in guidelines on the treatment of cholesterol to reduce vascular events, which have caused controversy by now recommending moderate-to-high intensity statin therapy for people aged 40-75 years at much lower absolute risk (i.e. 10 year absolute cardiovascular risk of 7·5% or higher compared to 20% or higher in previous guidelines), with no requirement for monitoring of lipid levels.[48] In fact, guidelines that have moved from target-based to absolute cardiovascular risk based treatment strategies, controversially risk medicalisation of ‘healthy older populations’ as all elderly individuals (>70 years) have an estimated absolute cardiovascular risk greater than 10%.

For peer review only


For peer review only - http://bmjopen.bmj.com/site/about/guidelines.xhtml

**Figure 2: The proportion of each age group in each absolute cardiovascular risk stratum for a) males and b) females. The shaded section under the thick black line shows the approximate percentage of each age group with a risk of 15% of developing a cardiovascular event in the next 5 years. The different shades of blue (from lighter to darker) show the proportions with a 5%, 10%, 15% and 20% risk.**

## a) Males

| | | Age | | | | | | | | | | | | | | |
|---|---|---|---|---|---|---|---|---|---|---|---|---|---|---|---|---|
| | | 25-29 | 30-34 | 35-39 | 40-44 | 45-49 | 50-54 | 55-59 | 60-64 | 65-69 | 70-74 | 75-79 | 80-84 | 85+ | SUM | % of all CHD deaths |
| Percentile of 10-year CHD mortality risk | 2-5 | 0 | 0 | 1 | 2 | 4 | 7 | 9 | 13 | 21 | 38 | 54 | 54 | 102 | 305 | 2 |
| | 6-10 | 0 | 0 | 1 | 2 | 5 | 9 | 12 | 16 | 25 | 44 | 61 | 64 | 109 | 348 | 3 |
| | 11-15 | 0 | 0 | 1 | 3 | 6 | 10 | 14 | 18 | 29 | 50 | 69 | 73 | 116 | 389 | 3 |
| | 16-20 | 0 | 1 | 2 | 3 | 7 | 12 | 16 | 21 | 32 | 54 | 76 | 83 | 123 | 430 | 3 |
| | 21-25 | 0 | 1 | 2 | 4 | 7 | 13 | 18 | 23 | 36 | 57 | 82 | 91 | 128 | 462 | 3 |
| | 26-30 | 0 | 1 | 2 | 4 | 8 | 14 | 19 | 25 | 39 | 59 | 88 | 98 | 133 | 490 | 4 |
| | 31-35 | 0 | 1 | 2 | 5 | 9 | 15 | 21 | 27 | 41 | 62 | 93 | 104 | 140 | 520 | 4 |
| | 36-40 | 0 | 1 | 3 | 5 | 10 | 16 | 22 | 30 | 44 | 65 | 97 | 109 | 147 | 549 | 4 |
| | 41-45 | 0 | 1 | 3 | 6 | 11 | 17 | 24 | 32 | 45 | 68 | 101 | 114 | 154 | 576 | 4 |
| | 46-50 | 0 | 1 | 3 | 6 | 11 | 18 | 25 | 34 | 48 | 71 | 105 | 119 | 160 | 601 | 5 |
| | 41-55 | 0 | 1 | 3 | 7 | 12 | 19 | 27 | 37 | 51 | 75 | 110 | 124 | 166 | 632 | 5 |
| | 56-60 | 1 | 2 | 4 | 7 | 13 | 21 | 29 | 40 | 55 | 79 | 115 | 129 | 171 | 666 | 5 |
| | 61-65 | 1 | 2 | 4 | 8 | 14 | 22 | 31 | 43 | 58 | 84 | 120 | 133 | 176 | 696 | 5 |
| | 66-70 | 1 | 2 | 4 | 9 | 16 | 24 | 34 | 47 | 63 | 88 | 126 | 139 | 182 | 735 | 6 |
| | 71-75 | 1 | 2 | 5 | 10 | 18 | 26 | 37 | 51 | 67 | 94 | 133 | 145 | 189 | 778 | 6 |
| | 76-80 | 1 | 3 | 5 | 11 | 20 | 29 | 42 | 56 | 73 | 102 | 141 | 153 | 197 | 833 | 6 |
| | 81-85 | 1 | 3 | 6 | 13 | 23 | 33 | 48 | 62 | 79 | 111 | 150 | 162 | 210 | 901 | 7 |
| | 86-90 | 2 | 4 | 8 | 16 | 28 | 39 | 57 | 72 | 89 | 124 | 161 | 174 | 236 | 1010 | 8 |
| | 91-95 | 2 | 5 | 11 | 19 | 33 | 45 | 66 | 82 | 99 | 141 | 173 | 187 | 263 | 1126 | 8 |
| | 96-100 | 2 | 6 | 14 | 22 | 39 | 52 | 75 | 92 | 109 | 158 | 185 | 199 | 290 | 1243 | 9 |
| | SUM | 12 | 37 | 84 | 162 | 294 | 441 | 626 | 821 | 1103 | 1624 | 2240 | 2454 | 3392 | 13290 | |
| | % of all CHD deaths | 0 | 0 | 1 | 1 | 2 | 3 | 5 | 6 | 8 | 12 | 17 | 18 | 26 | | |

## b) Females

| | | Age | | | | | | | | | | | | | | |
|---|---|---|---|---|---|---|---|---|---|---|---|---|---|---|---|---|
| | | 25-29 | 30-34 | 35-39 | 40-44 | 45-49 | 50-54 | 55-59 | 60-64 | 65-69 | 70-74 | 75-79 | 80-84 | 85+ | SUM | % of all CHD deaths |
| Percentile of 10-year CHD mortality risk | 2-5 | 0 | 0 | 0 | 0 | 1 | 1 | 2 | 3 | 6 | 13 | 24 | 46 | 123 | 219 | 2 |
| | 6-10 | 0 | 0 | 0 | 0 | 1 | 1 | 2 | 4 | 8 | 15 | 28 | 53 | 150 | 262 | 2 |
| | 11-15 | 0 | 0 | 0 | 1 | 1 | 1 | 3 | 5 | 10 | 17 | 31 | 61 | 176 | 306 | 3 |
| | 16-20 | 0 | 0 | 0 | 1 | 1 | 2 | 3 | 5 | 11 | 20 | 35 | 69 | 202 | 349 | 3 |
| | 21-25 | 0 | 0 | 0 | 1 | 1 | 2 | 3 | 6 | 12 | 22 | 38 | 75 | 223 | 383 | 3 |
| | 26-30 | 0 | 0 | 0 | 1 | 1 | 2 | 4 | 7 | 13 | 24 | 42 | 82 | 239 | 415 | 4 |
| | 31-35 | 0 | 0 | 0 | 1 | 1 | 2 | 4 | 8 | 14 | 26 | 45 | 87 | 254 | 442 | 4 |
| | 36-40 | 0 | 0 | 0 | 1 | 2 | 3 | 4 | 8 | 15 | 28 | 47 | 91 | 268 | 467 | 4 |
| | 41-45 | 0 | 0 | 0 | 1 | 2 | 3 | 5 | 9 | 16 | 31 | 51 | 95 | 283 | 496 | 4 |
| | 46-50 | 0 | 0 | 0 | 1 | 2 | 3 | 5 | 9 | 16 | 33 | 55 | 100 | 301 | 525 | 4 |
| | 41-55 | 0 | 0 | 1 | 1 | 2 | 3 | 6 | 9 | 17 | 36 | 60 | 105 | 323 | 563 | 5 |
| | 56-60 | 0 | 0 | 1 | 1 | 3 | 4 | 6 | 10 | 18 | 37 | 67 | 112 | 346 | 605 | 5 |
| | 61-65 | 0 | 0 | 1 | 2 | 3 | 4 | 7 | 10 | 19 | 39 | 75 | 120 | 367 | 647 | 6 |
| | 66-70 | 0 | 0 | 1 | 2 | 3 | 5 | 7 | 11 | 21 | 41 | 84 | 129 | 382 | 686 | 6 |
| | 71-75 | 0 | 1 | 1 | 2 | 4 | 5 | 8 | 12 | 23 | 44 | 93 | 140 | 391 | 724 | 6 |
| | 76-80 | 0 | 1 | 1 | 2 | 4 | 6 | 9 | 14 | 25 | 49 | 103 | 152 | 397 | 763 | 6 |
| | 81-85 | 0 | 1 | 1 | 3 | 5 | 7 | 11 | 17 | 27 | 55 | 115 | 167 | 406 | 815 | 7 |
| | 86-90 | 0 | 1 | 2 | 4 | 6 | 10 | 14 | 20 | 33 | 65 | 134 | 194 | 430 | 913 | 8 |
| | 91-95 | 1 | 1 | 3 | 5 | 8 | 12 | 19 | 26 | 41 | 79 | 153 | 221 | 455 | 1024 | 9 |
| | 96-100 | 1 | 1 | 3 | 6 | 10 | 14 | 24 | 32 | 51 | 93 | 172 | 249 | 482 | 1138 | 10 |
| | SUM | 2 | 6 | 15 | 36 | 61 | 90 | 146 | 225 | 396 | 767 | 1452 | 2348 | 6198 | 11742 | |
| | % of all CHD deaths | 0 | 0 | 0 | 0 | 1 | 1 | 1 | 2 | 3 | 7 | 12 | 20 | 53 | | |


For peer review only - http://bmjopen.bmj.com/site/about/guidelines.xhtml

### 2.7 Summary of rationale

- Meta-analysis of trials highlights the uncertainty about the efficacy of statins in the prevention of morbidity and mortality in older populations (over 70 years). This may stem from inadequate numbers in this age group included in major trials.
- Warnings about adverse effects of statins have recently been added to the product information but their frequency is uncertain and appears higher in the elderly.
- A strong potential exists for greater promotion of statins amongst the over 70 age group on the basis of recent guidelines advocating use for primary prevention at lower absolute cardiovascular risk levels. Evidence in the elderly is currently insufficient to support this proposal.
- Real world trials examining the impact of statins on a range of clinical outcomes important to older people have not been conducted.

For peer review only


For peer review only - http://bmjopen.bmj.com/site/about/guidelines.xhtml

# 3. Study Objectives

## 3.1 Primary objectives

To determine in people aged ≥70 years the effect of statin therapy (40 mg atorvastatin) versus placebo, over an average 6-year treatment period, on two co-primary clinical endpoints:

(i) a composite of all cause death or dementia or development of disability and;
(ii) a composite of cardiovascular death, non-fatal myocardial infarction or non-fatal stroke.

## 3.2 Secondary objectives

(1) To determine the effects of statin therapy versus placebo in people aged ≥70 years on each of: all cause death, cardiovascular death, dementia, other cognitive impairment, disability, all cause hospitalisation, need for permanent residential care, fatal and non-fatal myocardial infarction, fatal and non-fatal stroke (thromboembolic and haemorrhagic), fatal and non-fatal cancer, atrial fibrillation, heart failure and quality of life.

(2) To assess the cost-effectiveness of statin therapy for primary prevention in people aged ≥70 years.

## 3.3 Tertiary objectives

These include effects of statin therapy on pathology measures such as fasting glucose/HbA1c, urine ACR and estimated glomerular filtration rate (eGFR).

For peer review only


For peer review only - http://bmjopen.bmj.com/site/about/guidelines.xhtml

# 4. Hypotheses

## 4.1 Primary null hypotheses

Treatment with statin (atorvastatin 40 mg) compared with placebo will not prolong overall disability free survival amongst a healthy elderly population (≥70 years).

Treatment with statin (atorvastatin 40 mg) compared with placebo will not reduce the incidence of major adverse cardiovascular events amongst a healthy elderly population (≥70 years).

## 4.2 Primary alternative hypotheses

Treatment with statin (atorvastatin 40 mg) will prolong overall disability free survival amongst a healthy elderly population (≥70 years).

Treatment with statin (atorvastatin 40 mg) will reduce the incidence of major adverse cardiovascular events amongst a healthy elderly population (≥70 years).

For peer review only


For peer review only - http://bmjopen.bmj.com/site/about/guidelines.xhtml

# 5. Study Design

## 5.1 Trial Design

STAREE is a double blind, randomised placebo-controlled parallel group trial that is investigating whether statin therapy can prolong good health and maintain independence amongst older people.

## 5.2 Study Population

### 5.2.1 General considerations

The study will enrol men and women 70 years of age and over who are free from cardiovascular disease, diabetes and dementia and living independently in the community.

The study sites are listed in Appendix 1.

### 5.2.2 Inclusion criteria

Potential participants must meet the following criteria to be included in the study:

- Men and women aged ≥70 years living independently in the community,
- Willing and able to provide informed consent and accept the study requirements (Note: competent physical ability to participate in the trial is assessed using the KATZ ADL questionnaire)

### 5.2.3 Exclusion criteria

Potential participants who meet any of the following criteria will be excluded from the study**:**

- A history of clinical cardiovascular disease (defined as myocardial infarction, angina, coronary artery angioplasty and/or stenting, coronary artery bypass grafting surgery, heart failure, stroke, transient ischaemic attack (TIA), carotid stenosis, abdominal aortic aneurysm and peripheral vascular disease),
- Clinical diagnosis of dementia, treatment with medications for dementia (e.g. Aricept (Donepezil hydrochloride), Exelon (Rivastigmine) or Galantyl (Galantamine)) or a 3MS score <78 on screening.
- A history of diabetes or evidence of diabetes on pathology results (WHO diagnostic criteria whereby HbA1c ≥ 6.5%/ 48 mmol/mol or fasting glucose ≥7.0 mmol/L on two consecutive tests),
- Total cholesterol >7.5 mmol/L, [49]
- Moderate or severe chronic kidney disease (persistent proteinuria whereby urine albumin: creatinine ratio >30 mg/mmol [50] and/or eGFR <45 ml/min/1.73m$^2$ on two consecutive tests), [49]
- Moderate or severe liver disease (persistent elevations of transaminases of more than 3 times the upper limit of the normal laboratory reference range),
- Serious inter-current illness likely to cause death within the next 5 years such as terminal cancer or obstructive airways disease,
- Current participation in another interventional clinical trial,
- Absolute contraindication to statin therapy,


For peer review only - http://bmjopen.bmj.com/site/about/guidelines.xhtml

- Current use of statin therapy or other lipid lowering therapy for primary prevention and unwilling to stop therapy,
- Current long term or permanent use of potent cytochrome P450 (CYP) 3A4 inhibitors as specified in Appendix 3.

### 5.2.4 Rescreening or reassessment

Potential participants who are excluded on the basis of either their 3MS total score (<78) or their responses on the KATZ ADL questionnaire (unable to perform items 9-14) may be re-screened if the result is likely to be related to a temporary issue that may resolve over time (e.g. temporary depressive symptoms as indicated by the CES-D 10 or temporary disability related to fracture).

To be eligible for re-screening, participants with a 3MS score below 78 that also score 8 or above on the CES-D 10 (indicating presence of depressive symptoms) may be re-screened in 3 months. There may be instances where rescreening or reassessment of certain tasks may be required, such as participants with a temporary disability, and these participants will be considered for such on a case-by-case basis.

## 5.3 Study Intervention and Choice of Comparator

### 5.3.1 Intervention

Two tablets of Atorvastatin (2 x 20 mg/day) or identical placebo.

### 5.3.2 Rationale for choice of atorvastatin

Atorvastatin is the most commonly prescribed statin in Australia.

Atorvastatin is well researched with wide practice experience. Importantly, relative to other statins, it has a favourable side effect profile and has been shown to be one of the most potent statins for reducing low density lipoprotein concentrations (LDL-C) and triglyceride levels. The dose of 40 mg is expected to produce a 30-50% reduction in LDL-C (see table below).

For peer review only


For peer review only - http://bmjopen.bmj.com/site/about/guidelines.xhtml

**Table 1: Average relative reduction in LDL cholesterol concentrations with different doses of commonly used statins.** [51]

| | Daily dose of different statins | | | | |
|---|---|---|---|---|---|
| | 5 mg | 10 mg | 20 mg | 40 mg | 80 mg |
| Pravastatin | 15% | 20% | 24% | 29% | 33% |
| Simvastatin | 23% | 27% | 32% | 37% | 42% |
| Atorvastatin | 31% | 37% | 43% | 49% | 55% |
| Rosuvastatin | 38% | 43% | 48% | 53% | 58% |

Shaded boxes indicate regimens that can produce about a halving or more in LDL cholesterol concentrations (largely irrespective of patient characteristics, including presenting concentrations of cholesterol). The 2016 cost for generic atorvastatin 40 mg daily in the UK is about £2 per 28 days of treatment;[184] rosuvastatin 20 mg daily currently costs about £25 per month,[185] but it became available as a generic in the USA during 2016.

Different statins have different potencies and newer therapies such as atorvastatin and rosuvastatin produce larger reductions in LDL-C per mg of drug than older therapies, such as simvastatin and pravastatin.[51] Adverse events associated with statin treatment may be more likely with higher doses of specific statins (i.e. simvastatin ⩾ 80 mg) and with combination therapy (i.e. statin and fibrate). [52, 53] However, the flexibility of reducing from 40 mg (standard dose) to 20mg (low dose) will assist with exploring dose-response medication-related side effects and up or down titration will assist with reducing study medication discontinuation rates.

A very important issue that has not been addressed is the balance between expected benefits and harms of statin therapy in people aged over 70 years. STAREE will provide evidence to address this.

## 5.4 Participant recruitment

### 5.4.1 Recruitment

First participant expected to be consented to the study in October 2015 and the first participant expected to be randomised to study medication in November 2015.

### 5.4.2 Setting

STAREE will be conducted in Australian primary care (GP) practices and the community.

Registered GP co-investigators will access their computerised practice databases, in association with STAREE GP Liaison Officers if requested, to identify all patients aged 70 years and over. Depending on the practice database, patients will be further screened for inclusion and exclusion criteria. Those who are physically able to attend the practice and considered suitable for the study by their GP, will be invited to participate in the study via a letter of invitation.


For peer review only - http://bmjopen.bmj.com/site/about/guidelines.xhtml

### 5.4.3 Community participants

Any participants whose usual GPs are not registered as STAREE co-investigators will be considered as community participants. They will be assessed by a STAREE medical officer for suitability to be in the study. Their usual GP will be notified of their involvement and will be provided a copy of any pathology and any abnormal assessments that are performed as part of the study. Baseline and annual visits for community participants will be conducted at community venues or in the STAREE site offices.

### 5.4.4 STAREE hub locations

The STAREE central coordinating site is located in Melbourne, Australia.

STAREE hubs will be located in regional Victoria, Queensland, Tasmania, Western Australia, South Australia and New South Wales (see Appendix 1). Hubs will service the immediate and surrounding areas and may assist with other locations when requested.

### 5.5 Participant timeline and schedule of assessments

Figure 3 shows the participant timeline including the baseline screening phase, randomisation and follow up phase.

Table 2 shows a summary of the schedule of assessments to be undertaken at each study visit. Additional details of the cognitive assessments, physical measures and laboratory tests are provided in Appendix 6.

### 5.6 Baseline Screening Phase

The baseline screening phase will comprise a screening phone call, baseline visit 1, medication run in, GP visit and baseline visit 2.

Eligibility for randomisation into the study will be assessed across the baseline screening and medication run-in phase and will be based on the inclusion and exclusion criteria as well as compliance with the run-in medication.

### 5.6.1 Screening call

Potential participants will phone the study centre after they have received an invitation to participate in the study. At this phone screening call, they will be screened for eligibility to be included in the study. Those who pass this first screening stage will then be invited to attend baseline visit 1.


For peer review only - http://bmjopen.bmj.com/site/about/guidelines.xhtml

**Figure 3: Participant Timeline**

**Call**

**Baseline Visit 1 (Face to face or phone visit)**

- Informed consent obtained
- Questionnaires, exclusion criteria and pathology requested

**4 week run-in**

- Participant to take a 4-week run-in of 1 tablet of placebo
- Participant to see GP for inclusion in study

**Baseline Visit 2 (Face to face or phone visit)**

- Questionnaires are administered
- Review inclusion/exclusion criteria

**Randomisation**

- Participants randomised to placebo or active statin
- Participants to receive their study medication via tracked post (annual send outs)

**4 week post randomisation**

- Participants to take 1 tablet / 20 mg of study medication

**Call 4 weeks after randomisation**

- Participants to increase dosage to 2 tablets / 40 mg of study medication

**Participant contact post-randomisation**

- Participants will have a study follow up visit every 6 months (these study visits will alternate as phone visits or clinic/phone visit).
- Participants will have a final close-out study visit (clinic/phone visit)


For peer review only - http://bmjopen.bmj.com/site/about/guidelines.xhtml

**Table 2: STAREE measurement and study activity schedule**. A single X (with no superscripts) indicates that all specified measures carried out. The presence of a superscript indicates that the corresponding specific assessment is performed at that time point.

| | Screening | Baseline visits (BV) | | | Post-randomisation | | | |
|---|---|---|---|---|---|---|---|---|
| **Measurement/ Activity** | **Screening call** | **BV 1** | **GP visit** | **BV 2** | **1 month call** | **Annual visits (without cognitive testing)** | **Annual visits (with cognitive testing)** | **Close out visit** |
| **Type of visit** | **phone** | **clinic or phone** | **clinic or telehealth** | **clinic or phone** | **phone** | **clinic or phone** | **clinic or phone** | **clinic or phone** |
| **Month in trial (week)** | **0** | **0** | **0** | **0** | **(4)** | | | |
| **Review inclusion/ Exclusion criteria** | X | X | X | X | | | | |
| **Obtain informed consent** | | X | | | | | | |
| **Dispense study medication (supply posted after annual study visit once compliance checked)** | | X (run-in med only) | | Posted (after randomisation) | | | | |
| **Medication titration** | | | | | X | | | |
| **Assess medication compliance** | | | | X | | X | X | X |
| **Concomitant medications** | | X | | | | X | X | X |
| **Blood pressure & heart rate/rhythm[a], height[b], weight[c], waist:hip ratio[c],** | | X[a,b,c] | | | | X [a, c,] | X [a, c,] | X |
| **Lifestyle profile & personal medical history[d], family medical history[e] & IPAQ-E[f], physical function** | | X[d, e] | | X | | X[d, f] | X[d] | X[d] |
| **Routine Laboratory testing, GP referral to local pathology** Bloods**: fasting glucose[1], fasting lipids (TC, HDL, LDL, triglyceride)[1], Hb[2], CK[2], creatinine[3], (eGFR)[3], ALT[3], AST[3], HbA1c[3], Urine: ACR[3] | | X[1,2,3] | | | | X[3] | X[3] | X[1,3] |
| **Measurement/ Activity** | **Screening call** | **BV 1** | **GP visit** | **BV 2** | **Post-randomisation** | | | |

For peer review only


For peer review only - http://bmjopen.bmj.com/site/about/guidelines.xhtml

| | Screening | Baseline visits (BV) | | | Post-randomisation | | | |
|---|---|---|---|---|---|---|---|---|
| | Screening call | BV 1 | GP visit | BV 2 | 1 month call | Annual visits (without cognitive testing) | Annual visits (with cognitive testing) | Close out visit |
| **Type of visit** | **phone** | **clinic or phone** | **clinic** | **clinic or phone** | **phone** | **clinic or phone** | **clinic or phone** | |
| **Month in trial (week)** | **0** | **0** | **0** | **0** | **(4)** | | | |
| **Quality of life** SF-36 | | | | X | | X | X | X |
| **Assess cognitive function** 3MS$^{\pm}$ (and CES-D 10 to exclude depression); CAM[1] | | X | | | | | X | X |
| **Detailed cognitive tests**$^{\beta£}$ COWAT, Stroop, HVLT-R, SDMT, ADAS-Cog, Lurian Overlapping Figures, Trail Making Test | | | | X | | | X | X |
| **Assess physical disability** Life disability KATZ ADL | | X | | | | X | X | X |
| **Biobank samples collected** | | | | X | | | | |
| **Clinical event reporting** Questionnaire & medical report review | | | | | | X | X | X |

**All study visits, including baseline and follow up visits, may be either conducted in person or by phone call. For visits conducted by phone call, physical measures (blood pressure, heart rate and weight) will be collected from medical records if available.

± Full 3MS is conducted at clinic visits and a partial 3MS is conducted at phone visits

$^{\beta}$ Annual visits with cognitive testing conducted by phone will not include SDMT, STROOP, ADAS-Cog and Lurian Overlapping Figures. These tests will only be conducted at in person visits.

$^{£}$B-ADL (Bayer Activities of Daily Living) is administered to the named 'study partner' of any participant who shows a pre-specified decline in cognitive tests at an annual visit. If the participant does not consent to a study partner undertaking the B-ADL, then it may be administered to the participant themselves.



For peer review only - http://bmjopen.bmj.com/site/about/guidelines.xhtml

For peer review only

### 5.6.2 Lifestyle profile and screening visits (Baseline visits)

STAREE staff will explain the study to participants. After informed consent is provided measurement and medical history data will be collected to further assess eligibility for the study. Participants will be given a copy of their consent form. Participants will be assigned a unique identification number, which will be used throughout the study.

The information to be collected and reviewed at the baseline visits will include the following:

a. Basic demographic and lifestyle factors
b. Past medical history – identified by the participant and GP practice medical record or patient health summary (as available)
c. Cognitive assessment – 3MS[54] (full or modified version if phone visit)
d. Depressive symptoms assessment – CES-D 10[55]
e. Physical disability – KATZ ADL using Life Ability questionnaire[34]
f. Blood pressure, heart rate and rhythm, weight, height and waist circumference
g. Concomitant medications
h. Pathology – referred to local pathology collection service for blood and urine tests

Note: Pathology testing results from participants' medical records may be used to assess eligibility if performed no more than 6 months before Baseline visit 1. HbA1c may be used if performed within the past 12 months.

Medication for the run-in phase will be handed out.

### 5.6.3 Post screening GP/medical visit (Run-in phase between Baseline visits 1 and 2)

Participants will see their GP who will be an enrolled co-investigator (or the STAREE medical officer if the usual GP is not an enrolled co-investigator) and will complete an Inclusion/Exclusion Criteria Worksheet. The baseline pathology testing results will be reviewed by the GP (or the STAREE medical officer). These results will help determine eligibility via the inclusion and exclusion criteria.

### 5.6.4 Post screening visit study activity (Run-in phase between Baseline visits 1 and 2)

Participants will enter a run-in phase of 4 weeks whereby they will be asked to take one tablet of study medication (placebo) a day.

### 5.6.5 Determinations of Eligibility and Assessments visit (Baseline Visit 2)

Participants will have a second visit with STAREE field officers after the run-in phase. This visit will be conducted within 4 to 6 weeks of the baseline visit 1.

Participants will be deemed eligible to participate if they are compliant with run-in medication pill count (>85% taken), have satisfactory pathology testing and their GP (or the STAREE medical officer) has provided approval using the GP screening form.

If deemed eligible to continue to baseline visit 2, participants will undergo the following assessments at this second screening visit:

a. Quality of life – Short Form (SF)--36 Questionnaire.[56]


For peer review only - http://bmjopen.bmj.com/site/about/guidelines.xhtml

b. Comprehensive assessment of cognition – COWAT[57], STROOP[58], HVLT-R[59], SDMT[60], ADAS-Cog[61], Lurian Overlapping Figures[62], Trail Making Test[63] (if funding available).

Participants' willingness to donate and have blood samples collected and stored in the STAREE biobank will be ascertained at the baseline visit 2 and offered at STAREE hubs with facilities for sample processing and storage.

The purpose of this sample collection is to allow identification and analysis of potential biomarkers that predict the onset of age-related diseases such as cardiovascular disease, dementia, cancer and diabetes.

### 5.7 Randomisation and follow up phase

The randomisation and follow up phases are illustrated in Figure 3.

#### 5.7.1 Randomisation procedure

Following the completion of data collection at baseline visit 2, the Randomisation Form will be completed, and participants will be randomised into the STAREE study via the study website.

An independent statistician will generate the allocation list, using the statistical package R. This arrangement will ensure that the randomisation code remains inaccessible to all study staff and investigators. The allocation list will be generated using Cox model randomly permuted blocks (of size either 2 or 4), with randomisation stratified for site and age group (<80y, 80+y). Blocks will ensure ongoing approximate balance of randomisation to statin and placebo within strata. Treatment allocation will be achieved through a web or interactive voice recognition telephone-based system.

Following the completion of the randomisation process the study medication will be posted to participants.

Participants' contact details will be confirmed to ensure the correct mailing address for study medication is recorded and the addresses of the closest relative, friend and local doctor provided.
Participants will be instructed to take one tablet of study medication a day.
Participants will be instructed to call the study centre if they have any adverse effects.

The distribution of study medication is detailed in Section 8.

A record of all participants undergoing screening and randomisation will be made in the appropriate tracking log.

#### 5.7.2 Post randomisation procedure

Participants will be contacted by phone one month after commencement of study medication to review tolerability and compliance with study medication and instruct them to increase the dose from one tablet to two tablets. The date of this phone contact will be taken from the drug receipt log as recorded in the STAREE database.


For peer review only - http://bmjopen.bmj.com/site/about/guidelines.xhtml

Participants reporting any tolerability issues with the study medication will be referred back to their GP for further advice and management.

**Table 3. Study medication**

| | 0 | 4 weeks |
|---|---|---|
| Number of tablets | 1 | 2 |
| | Receive study medication | Phone call to increase to 2 tablets |

### 5.7.3 Blinding

All STAREE staff including the investigator team, management, administration, medical officers, GP Liaison Officers, Field Officers, Data Officers and students will be blinded to treatment allocation through the randomisation procedure and study duration.
All GP co-investigators and practice staff will be blinded to treatment allocation.
All participants will be blinded to treatment allocation.

In the event of a clinical emergency, participants will be assumed to be on active treatment and the recommendation will be to cease treatment.

A process for emergency unblinding will be in place at Ramsay Healthcare. Selected Ramsay Healthcare staff will be granted access to the randomisation code. This information will be accessible and shared using a Secure File Transfer Portal (SFTP).

The need for unblinding of study participants will be determined on a case-by-case basis after referral to a senior STAREE medical officer.

### 5.7.4 Annual Follow up visit

Each year after randomisation, participants will participate in a study visit with a Field Officer. Visits may be conducted face-to-face or by phone. Annual measurements will include cognitive tests and disability assessments, lifestyle questionnaires as well as physical measures. The Quality of Life (SF-36), Life Ability and Annual medical history questionnaires will be provided in an online format or posted to participants who have agreed to this. For all other participants these surveys will be conducted at face-to-face visits. Pathology will be repeated (excluding lipids). Participants will be asked about their recent medical history and any concomitant medications they are taking. Additionally, participants will be asked about serious adverse events (SAEs) and potential study endpoints.

Following notification, clinical information relating to any SAE or endpoint will be sourced through: a) medical record review and b) hospital records and discharge summaries.

### 5.7.5 Phone Contact

After randomisation and the mail out of study medication, if receipt of study medication has not been registered a participant will be contacted by phone.


For peer review only - http://bmjopen.bmj.com/site/about/guidelines.xhtml

Annual phone calls (6, 18, 30, 42, 54, 66 months) will be made to ask participants about SAEs and potential study endpoints and to encourage compliance with study medication.

Data to be collected during the phone calls include:

- Compliance with study medication
- Serious adverse event and endpoint notification
- Concomitant medications
- Physical disability (Life disability KATZ ADL)

### 5.7.6 Other Actions

If a participant does not proceed to randomisation, then the reason for non-randomisation is to be documented and collected on all relevant hard copy and electronic (database) Case Report Forms (CRF) such as the Screening CRF and Assessment and Randomisation CRF Contact with the participant regarding the outcome of the screening phase will be recorded within the Participant Communication Log

### 5.8 Economic Analysis

In addition to the disability free survival and cardiovascular outcomes, STAREE will examine the cost effectiveness of statin treatment for prolonging disability free survival and preventing major adverse cardiovascular events in healthy individuals aged ≥70 years.

Decision analysis will be used to compare the downstream consequences of atorvastatin versus placebo. Incorporation of Markov and life-tabling techniques will allow for the modelling of outcomes beyond the 6-year duration of the trial.

The main output of interest in health economic modelling will be the incremental cost-effectiveness ratio in terms of net costs per unit of health gain. Net costs will comprise the costs of statin minus costs saved from the reduction in downstream health services utilisation. Health gains will be measured by years of life and quality-adjusted life years (QALYs) gained. Both will be enabled by the collection of time-to-outcome data and QALYS also by collection of quality of life data. Cost data will be estimated by applying published unit costs (from an Australian healthcare system perspective) to data on health services utilisation. All health economic analyses will be undertaken in accordance with recommended approaches, such as 5% discounting of estimated future costs and health gains. To account for any uncertainty in the data inputs for health economic modelling, sensitivity and uncertainty analyses will be undertaken via Monte Carlo simulation.

## 6. Study Endpoints

### 6.1 Primary endpoints

STAREE will have two co-primary endpoints, disability free-survival and major adverse cardiovascular events, which are both composite endpoints.

1. Disability-free survival - defined as survival free of dementia  or persistent physical disability (as derived from the endpoints of all-cause mortality, dementia and physical disability), and


For peer review only - http://bmjopen.bmj.com/site/about/guidelines.xhtml

2. Major adverse cardiovascular events – defined as the first occurrence of a non-fatal myocardial infarction, non-fatal stroke or cardiovascular death.

### 6.2 Secondary endpoints

Two types of outcome measures are pre-specified secondary endpoints in STAREE. The first group are components of the co-primary endpoints and additional cognitive and cardiovascular endpoints (heart failure, other acute coronary syndromes and revascularisations). The second group include non-cardiovascular endpoints (need for permanent residential care, hospitalisation and cancer).

1. All cause death
2. Cardiovascular death
3. Fatal and non-fatal myocardial infarction
4. Fatal and non-fatal stroke
   a. Haemorrhagic stroke
   b. Thromboembolic stroke
5. Persistent physical disability (Life Ability KATZ-ADL questionnaire[34])
6. Dementia
7. Other cognitive impairment (not meeting criteria for Major Neurocognitive Disorder)
8. Approved need for permanent residential care (based on reporting by an Aged Care Assessment Team)
9. All cause hospitalisation (reasons and length of stay)
10. Heart failure
11. Atrial fibrillation
12. Revascularisation procedure
13. Fatal and non-fatal cancer (excluding non-melanoma skin cancer)
14. Quality of life (SF-36 questionnaire[56])
15. Cost-effectiveness

Please refer to Appendix 2 for brief definitions of primary and secondary endpoints.

### 6.3 Endpoint ascertainment

At 6-month telephone calls and annual visits, participants will be asked whether they have been diagnosed with any new health events which are from a list of study endpoints. Notification of a potential study endpoint will trigger the collection of further information for endpoint confirmation and adjudication. Confirmation of endpoints will be ascertained by collecting information from any of the following sources within 90 days of learning of the event:

- Details from medical records from the usual treating physician or practice held medical record.
- Medical records obtained by letter/fax/email contact with other treating specialist physicians or secondary/tertiary medical care centres.
- Case Report Forms.
- Hospital records/discharge summaries, pathology reports.
- Death Certification: The National Death Index will be routinely sourced for notification of death not identified through the above processes for up to 5 years after the completion of study.

For peer review only


For peer review only - http://bmjopen.bmj.com/site/about/guidelines.xhtml

- Review of Commonwealth Department of Health and Ageing (DOHA) database of aged care assessments (ACAP dataset).
- Review of cancer registries for up to 5 years after the completion of study.
- Aged Care Assessment Service (ACAS report): A copy is provided to the GP and assesses the needs and level of care recommended for the individual.

### 6.4 Endpoint adjudication process

An end-point adjudication committee (EAC) will be appointed with the task of assessing the clinical data relating to all endpoints. Data collected from GPs, hospitals, specialists and other sources by the research staff will be presented to the EAC. The information available to the EAC will not only include the clinical source documentation obtained from the GP records but also death certificates and, if available, autopsy reports blinded to participant identity. The EAC will adjudicate through an online system with meetings conducted to resolve discordant adjudications and to develop decision rules.

## 7. Sub-studies and ancillary studies

### 7.1 STAREE-HEART

STAREE-HEART will conduct a suite of additional cardiac assessments in a subset of up to 500 STAREE participants before they are randomised to study medication. It aims to determine the effect of statin treatment over a 3 year period compared with placebo on markers of cardiac ageing. These include an echocardiogram which measures global longitudinal strain (GLS), which may predict incident atrial fibrillation (AF) and heart failure, and twice daily handheld ECG recordings over a 2-week period which may improve AF detection 4-fold compared to usual screening. Additionally, a new energy waveform 12-lead ECG will be performed. The assessments will take place prior to randomisation and will be repeated at the year 3 follow up.

### 7.2 STAREE-MIND

STAREE-MIND will conduct neuroimaging assessments in a subset of up to 300 STAREE participants. STAREE-MIND aims to determine the effect of statin treatment on longitudinal measures of brain health including grey matter volume, white matter integrity and white matter hyperintensities, cerebral perfusion and metabolism, and iron loading. It will also examine the relationship between imaging changes in the brain and cognitive impairment and dementia. The imaging will be conducted prior to commencement of the study medication and will be repeated at 4 years of follow up. The detailed STAREE MIND protocol is shown in appendix 10.

### 7.3 STAREE Healthy Ageing Biobank

Up to 2000 STAREE participants located near STAREE study sites with facilities for processing and storage of biospecimens (Melbourne, Perth and Hobart) will be invited to provide a blood and urine sample for the STAREE Healthy Ageing Biobank. This study aims to create a collection of biospecimens which can be used in future research to identify new biomarkers that are linked with development of clinical outcomes such as stroke, myocardial infarction, cancer and dementia.


For peer review only - http://bmjopen.bmj.com/site/about/guidelines.xhtml

## 8. Contingency plans for participant wellbeing

Participants will be advised to seek care from their usual treating GP (or other health care providers) for any medical condition arising during the course of the study. Any test measurement result that is outside the normal range will trigger a notification to the participant's GP.

Treating GPs will be co-investigators and provided with information about the study to aid the participant's involvement in the study (wherever possible).

### 8.1 Commencement of regular lipid lowering agents

A prescription for a lipid lowering agent will be reason to stop the study medication. The participant will cease study medication but continue in the study for observation (routine follow up visits).

### 8.2 Development of an indication for statins

Any participant who develops a recognised clinical indication (such as a myocardial infarction or stroke) for statin treatment may commence routine statin therapy. The participant will cease study medication but continue in the study for observation (routine follow up visits).

### 8.3 Post-trial care

At the end of the interventional phase of the trial, it is anticipated that participants will be followed passively for an additional 5 years, contingent on funding being obtained. During this period, study participants will continue to be treated by their usual GPs and follow up will be conducted annually by face to face or phone contact. Study participants will be asked to provide informed consent for the extended post-trial follow up.

For peer review only


For peer review only - http://bmjopen.bmj.com/site/about/guidelines.xhtml

# 9. Levels of participant involvement

## 9.1 Levels of participant involvement

The aim of the study is to assess outcomes on an intention to treat basis, i.e. to test the decision to prescribe a statin and the consequences of that decision. Therefore, every effort will be made to minimise loss to follow up with respect to determining mortality, dementia, disability and major adverse cardiovascular events. Loss to follow up will be minimised by recording three contacts for each participant including their GP, close relative living apart, and close friend.

Full participation in STAREE means that a participant is currently taking study medication and attending for study visits and/or phone calls. If participants request to change their level of involvement or withdraw from the study it will be explained to them that there are number of options. These may include the following:

*Changes to study medication*

Participants may decide to interrupt or discontinue the study medication for a number or reasons but regardless of this decision, they can continue to be active participants in STAREE:

*On Medication:* Participants are taking study medication (one or two tablets daily).

*Medication interrupted:* Participants have temporarily stopped taking study medication due to circumstances such as illness, injury or holidays or for other personal reasons. Participants who have interrupted their medication intend to re-start medication at a future date when the circumstances that have led to the interruption are resolved.

*Medication discontinued:* Participants have stopped taking study medication permanently. This may be due to adverse effects or for other personal reasons, or because they have developed a clinical indication and been prescribed an open label statin. In all cases, when a participant discontinues study medication, the reason for discontinuation and date of the last dose of study medication will be recorded

Participants who have stopped taking study medication and are deemed by a STAREE medical officer as suitable for medication re-challenge will also be considered as "interrupted" until they either re-start or are deemed to have permanently discontinued medication.

*Changes to study visits (clinic and/or phone calls)*

Regardless of whether participants are taking study medication or not, they will still participate in study visits (clinic and/or phone calls). If participants do not want to participate in study visits (clinic and/or phone calls), they may elect to participate by review of their medical records corresponding with the trial's duration (such as GP and hospital records) and national morbidity and mortality databases). All participants will be asked to provide approval for access to this information as part of the study enrolment procedures.



For peer review only - http://bmjopen.bmj.com/site/about/guidelines.xhtml

### 9.2 Withdrawal

Participants will be considered to have withdrawn from the study if they revoke their consent. This means that they are not taking study medication, are not participating in study visits (clinic and/or phone calls), and that medical record review is not being conducted. All data that participants have contributed to the study up to the date of withdrawal will remain in the study database, and review of medical records dated earlier than the withdrawal date may be completed. Publicly available data such as death (e.g. from death notices) may also be used in such cases.

For peer review only


For peer review only - http://bmjopen.bmj.com/site/about/guidelines.xhtml

# 10. Study Medication and Supplies

## 10.1 Study treatments

*Run-in placebo*

The 4 week placebo medication will be provided in either a labelled box or bottle (one box or bottle of 40 tablets). It will be given to participants at the completion of baseline visit 1.

*Double-blind Study Medication*

Study medication will be provided by Ramsay Healthcare in boxed blister packs (3 sheets of 10 tablets), with the STAREE logo printed on the box. Participants will be randomly allocated to one of two treatments; a) atorvastatin (2 x 20 mg tablets) or b) placebo (2 x 20 mg tablets with identical appearance). Study medication will be posted regularly throughout follow-up, at least every 3-9 months (usually at 6-month intervals).

*Post randomisation*

After randomisation, study medication will be posted directly to the participant's home or provided to the participant at an annual visit. Participants will be directed to start taking one tablet of study medication from a labelled first box containing 30 tablets. Subsequently participants will be directed to take two tablets from boxes containing 30 tablets.

## 10.2 Administration of study medication

*Run-in period*

Participants will be directed to take 1 tablet of run-in medication at the same time every day.

*Double blind period*

Participants will be directed to take 1 tablet (20 mg) at the same time every day for the first 30 days of the trial. After 30 days, participants will be directed to take 2 tablets (40 mg) at the same time every day.

If a suspected intolerance of the full dose (2 tablets) of study medication (atorvastatin or placebo) develops, the participants' GP may reduce the dose to 1 tablet at their own discretion.

*Administration of study medication for those taking allowed Cytochrome P450 3A4 inhibitor at study entry and during follow up*

Participants identified as taking certain (allowed) cytochrome P450 3A4 inhibitors will be directed to take 1 tablet (20 mg) of study medication per day for the duration of the study. The list of allowed cytochrome P450 3A4 can be found in Appendix 3.

## 10.3 Dispensing and randomisation

The number of authorised dispensing staff will be limited to assure proper adherence with established accountability and dispensing procedures. All procedures are described in the STAREE Drug Accountability Manual.

Medication for the run-in phase will be dispensed to the participant by the study staff at baseline visit 1. Each participant will be dispensed 40 placebo tablets and be directed to


For peer review only - http://bmjopen.bmj.com/site/about/guidelines.xhtml

take one tablet at the same time daily. When the participant returns for the final assessments and eligibility visit (baseline visit 2), medication compliance will be checked prior to randomisation.

Following the completion of the randomisation process, study medication will be posted to the participant at regular intervals, as required, to ensure continuity of study medication supply for each participant. The participant will be asked to confirm receipt of the study medication by calling the STAREE coordinating centre. If confirmation of receipt has not been made within 10 days of dispatch of the study medication research, staff will contact the participant to ensure receipt. Attempts to contact the participant will continue until receipt has been confirmed. In the rare instance of missing or undelivered study medication, the study medication will be re-issued and sent to the home of the participant. Note, the postage of study medication to participants will be tracked with the delivery or courier service with the signature of the recipient being required upon delivery.

The Medication ID number assigned to the participant will remain the same for the duration of the study with each separate batch being distinguished by a batch number appended to the medication ID number. The participant will receive study medication with the same Medication ID number at each visit.

A '*Study Drug Log'* will record the medication dispensed and the medication returned from the participant.

The *Study Drug Log* will record information on the dispensing of study drug and will include the following:

- Participant ID number and participant acrostic
- Medication ID number
- Date dispensed (initials of dispenser)
- Date of medication return (initialled by research staff)
- Number of tablets returned
- Comments section to allow for explanation of discrepancy, if applicable.

These processes are further detailed in the drug accountability manual.

### 10.4 Study medication compliance

Compliance with study medication will be checked at six-monthly telephone calls and annual visits. Questions regarding study medication use will also be asked at annual visits to encourage medication compliance (see Appendix 4). The questions are based on the Brief Medication Questionnaire [64] and Morisky questionnaire[65]. Participants will be asked to count and report any unused tablets at annual visits.

### 10.5 Study medication storage

Study medication storage will be the responsibility of Ramsay Healthcare. Study medication will be predominantly stored at a secure storage centre in a temperature-controlled environment. Placebo run-in medication is released to STAREE periodically, in-bulk, and stored at the coordinating centre. All placebo medication for the run-in phase will be stored


For peer review only - http://bmjopen.bmj.com/site/about/guidelines.xhtml

in a secured area with restricted access, at room temperature under the supervision of the coordinating centre.

The receipt, storage, dispensing, accountability and study medication collection, for both the run-in and randomisation phases, will be the responsibility of both Ramsay Healthcare and the STAREE coordinating centre. Each party will be responsible for maintaining appropriate delivery, storage, and transfer records.

### 10.6 Study medication accountability and dispensing logs

All study medication dispensed in the trial will be accounted for by confirming the delivery, receipt and commencement dates of each study medication supply. Study medication compliance will be assessed and confirmed by collection, counting and documentation of unused study medication from participants and/or by confirmation of the completion and/or cessation dates of each individual supply with participants by phone.

All unused study medication will be collected to allow for proper destruction of the medication. Unused tablets and packaging will be returned by participants to trial staff at face to face study visits. If unused study medication is not able to be collected at study visits, attempts will be made to confirm safe destruction (see section 8.7 below). Any lost medication packs will be documented, re-dispensed and posted to the participant, with all the details recorded to allow an audit trail. Tablet counts will be made of all returned medication packs. The coordinating centre will make all reasonable attempts to verify that any unused or partially used medication supplies are returned by the study participant and that no remaining supplies are in the participant's possession.

The coordinating centre will maintain records which adequately document that study participants were provided study medication supplies and doses as specified by the protocol and document and reconcile all study medication received from the participants (see section 8.3).

### 10.7 Study medication disposal

Unused study medication will be collected at annual visits by study staff whenever possible and disposed. Unused study medication will then be relinquished for safe destruction at a local pharmacy or destruction service. Each batch of destroyed study medication will be documented, along with the date, location and the pharmacy staff name, in the STAREE Study Medication Tracking Log (more detailed information is provided in the STAREE Drug Accountability Manual).

If unused study medication is not able to be collected at an annual study visit, participants will be given the option of disposing their supply at the local pharmacy (i.e. through the Return of Unwanted Medication or RUM scheme) or return via a reply-paid envelope satchel. Destruction of unused study medication by participants at pharmacies or other safe medical waste service will be documented on the STAREE Disposal Form, which will be returned to the STAREE trial for record keeping.


For peer review only - http://bmjopen.bmj.com/site/about/guidelines.xhtml

### 10.8 Overdose of study medication

There is no specific treatment for atorvastatin overdose. Should an overdose occur, the participant should seek emergency medical care and be treated symptomatically with supportive measures instituted as required. The Poisons Information Centre on 13 11 26 (Australia) may also be contacted for advice on the management of an overdose.

## 11. Concomitant Therapy

### 11.1 Prescription medications

All prescription medicine will be recorded on a Concomitant Medications Form including indication for use and start/stop dates. Concomitant medications will be checked at annual study visits where participants will be asked to bring in any medications/vitamins/ supplements they are taking and by review of GP medical records.

For peer review only


For peer review only - http://bmjopen.bmj.com/site/about/guidelines.xhtml

## 12. Sample Size and Power calculations

**Table 4. Sample size calculations**

| | ITT effect size (RR) | Power | Required number of events | Required number of participants | On treatment (true) effect |
|---|---|---|---|---|---|
| For disability free survival (Death or Dementia or Disability) | **14.5%** | **83.1%** | **1,397** | **9,631** | **18%** |
| For major adverse cardiovascular events (CV death, non-fatal MI or non-fatal stroke) | **20.2%** | **80.0%** | **619** | **9,631** | **25%** |

Based on these sample size calculations 9,631 participants will be recruited and a total of 58,135 person-years accrued.

These calculations assume:

Loss to follow up: 2% per annum (which is expected to be conservative, i.e. the true loss to follow up is expected to be very small assuming good retention of participants and the ability to access medical records or perform record linkage for those who cease regular follow up). This translates to expecting an average of 6.04 years of follow up time per participant.

A two-sided significance level of 5% (alpha rate).

An anticipated age structure of STAREE participants of 56%: 27%: 12%: 5% in the respective age bands 70-74 years: 75-79 years: 80-84 years: 85+ years. These proportions are based on recruitment of the initial 7000 participants to the study and expectations about future recruitment.

Treatment cross-over: In year 1 of follow up we assume 8% of participants who have been randomised to statin will cease taking their study medication. Thereafter, we assume 3% of participants per annum in the statin group will cease taking study medication. In year 1 of follow up we assume 1% of participants in the placebo group will commence statin. Thereafter we assume 3% of participants per annum in the placebo group will commence statin therapy. Further we assume that there are no subsequent treatment "re-cross-overs", i.e. assume a worst-case scenario for the hazard ratio that will be observed in the intention to treat analysis.

The following event rates:

**Death, dementia or persistent disability (disability-free survival) - co-primary endpoint A**

*All cause death*


For peer review only - http://bmjopen.bmj.com/site/about/guidelines.xhtml

We anticipate death rates in STAREE randomised participants will be less than half those in the general population. Specifically, the STAREE anticipated age-specific death rates are as follows:

| | |
|---|---|
| 70-74y | 10.0 per 1000 person-years |
| 75-79y | 17.7 per 1000 person-years |
| 80-84y | 33.6 per 1000 person-years |
| 85+y | 66.6 per 1000 person-years |

These event rates have been informed by corresponding mortality rates from the ASPREE (Aspirin in Reducing Events in the Elderly) randomised clinical trial and from underlying Australian population death rates for 2011, obtained from http://www.abs.gov.au/AUSSTATS/abs@.nsf/DetailsPage/3302.02011?OpenDocument

*Dementia*

Population rates of dementia have been estimated as 11 per 1000 person years in the 75-59 year old age group although fluctuations have been observed. [66-68] For Australia, there is a paucity of national data to inform incidence rates, as noted by AIHW in 2012. The ASPREE trial provides a highly relevant contemporary source of information. A dementia rate of 6.8 per 1000 person years was observed in ASPREE (median age 74 years) although almost no cases occurred in the first year of follow up so the rate was higher in later years. In ASPREE, dementia was screened for using a 3MS reduction of at least 10 points and there was a 60% conversion of the screen-positives to confirmed cases of dementia. In STAREE a 3MS reduction of at least 5 points will be used, which is expected to approximately double the number of screen positives but lower the conversion rate to confirmed cases of dementia to 50%. From these considerations, 3MS screening in STAREE is expected to yield 33% more cases of dementia than in ASPREE. Given this, and detailed age-specific data from ASPREE, the dementia rates in the later years of follow up in STAREE are expected to be as follows, using participant age at randomisation:

| | |
|---|---|
| 70-74y | 5.8 per 1000 person-years |
| 75-79y | 12.4 per 1000 person-years |
| 80-84y | 26.9 per 1000 person-years |
| 85+y | 36.6 per 1000 person-years |

*Physical Disability (defined as persistent loss of an activity of daily living)*

This component is defined by a persistent “a lot of difficulty” or “unable to do the activity” rating, or “Yes” answer to receiving help to perform the same Katz activity of daily living (ADL) domain/s (items 9 to 14), which is confirmed at least 6 months previously as loss of the same ADL. If it is not possible to obtain a Katz ADL assessment, notification of admission to aged care or community assessment will initiate collection of relevant clinical records to substantiate a disability endpoint. The rate of this endpoint was negligible in the first year of ASPREE. The following rates are expected to be seen in later years of follow up in STAREE and are based on observed rates in ASPREE, using participant age at randomisation:


For peer review only - http://bmjopen.bmj.com/site/about/guidelines.xhtml

70-74y 4.5 per 1000 person-years
75-79y 7.6 per 1000 person-years
80-84y 12.5 per 1000 person-years
85+y 25.0 per 1000 person-years

*Overlap of death, dementia and persistent physical disability*

The overlap between these three components will be assumed to be 10%. This is consistent with the observed overlap between dementia and physical disability occurring prior to death in ASPREE. Hence the composite STAREE event rate is estimated to be the sum of the 3 component event rates less 10% of that sum.

The calculations of expected primary endpoint event rates in years 3-5 of follow up after randomisation are as follows:

70-74y: (10.0 + 5.8 + 4.5)*0.9 per 1000 person-years = 18.3 per 1000 person-years
75-79y: (17.7 + 12.4 + 7.6)*0.9 per 1000 person-years = 33.9 per 1000 person-years
80-84y: (33.6 + 26.9 + 12.5)*0.9 per 1000 person-years = 65.7 per 1000 person-years
85+y: (66.6 + 36.6 + 25.0)*0.9 per 1000 person-years = 115.4 per 1000 person-years

*Consideration of healthy volunteer effect*

It is anticipated that the event rates for disability-free survival calculated above will be reached by STAREE participants in years 3-5 following randomisation. In the initial 2 years of follow up participants are assumed to be healthy relative to the population, i.e. in the first 2 years of follow up STAREE participants are assumed to experience lower event rates. An event rate of 0 (zero) per 1000 person years is assumed in the first year of follow up for dementia and physical disability and a rate of 4 per 1000 person years is assumed for mortality. In the second year of follow up it is assumed that the primary endpoint event rate will be equal to half of that anticipated above in years 3-5.

**Major adverse cardiovascular events (cardiovascular death, non-fatal myocardial infarction or non-fatal stroke) – co-primary endpoint B**

This composite endpoint is defined as the first occurrence of cardiovascular death or non-fatal myocardial infarction or non-fatal stroke. In ASPREE this endpoint has been evaluated to the extent possible (of reported non-fatal myocardial infarction/angina events reported approximately 45% will be adjudicated as a non-fatal myocardial infarction). The figure below shows the estimated rate of major fatal and non-fatal cardiovascular events in ASPREE participants by age group. Of note, the underlying event rate seems fairly constant over the time from randomisation, i.e. the initial “healthy volunteer” effect that is seen in mortality and can be anticipated for admission to care, dementia and loss of activity of daily living, does not seem to apply to major adverse cardiovascular events. The three components, non-fatal myocardial infarction: non-fatal stroke: cardiovascular death, contribute events to the composite endpoint in the approximate ratio of 2:2:1 respectively.

For peer review only


For peer review only - http://bmjopen.bmj.com/site/about/guidelines.xhtml

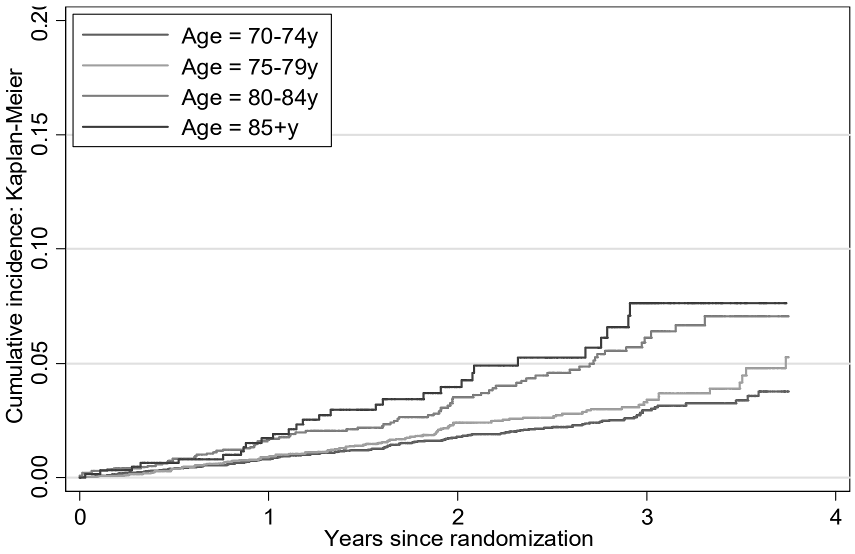


Based on the rates seen in ASPREE the following rates are anticipated in STAREE:

Years 1-5:

| | |
|---|---|
| 70-74y | 9.5 per 1000 person-years |
| 75-79y | 11.5 per 1000 person-years |
| 80-84y | 18.5 per 1000 person-years |
| 85+y | 24.5 per 1000 person-years |


For peer review only - http://bmjopen.bmj.com/site/about/guidelines.xhtml

## 13. Safety Monitoring and Reporting

### 13.1 Introduction

In STAREE the primary responsibility for the safety of individual participants will rest with the participant's usual general practitioner who is a co-investigator in the study. Additionally, an independent Data and Safety Monitoring Board (DSMB) will be appointed and will have primary responsibility for monitoring the study data for adverse trends and drug-related serious adverse events (SAEs). The responsibilities of the DSMB are outlined in the STAREE DSMB charter and DSMB plan.

### 13.2 Safety Monitoring

Several types of safety issues and SAEs may occur in STAREE participants. At each point of contact with participants, STAREE staff will specifically query participants for adverse events. Information on adverse events may also be reported to study staff between study visits by participants through contact with the trial centre by phone or email.

Participants will be encouraged to discuss any adverse events with their usual GP. GPs will be notified by STAREE staff of any abnormal test results and the STAREE medical officer will be available to discuss any issues with participants or their GPs.

### 13.2.2 Adverse events (AE)

The definition of an Adverse Event (AE) in the STAREE trial has been adapted from the National Institute of Aging definition. At the designated intervals for event collection, participants will be asked about specific events of interest as well as any new diseases or conditions. AEs will be obtained by self-report and some may require supporting documentation. AEs will be reported biannually to the Data Safety Monitoring Board (DSMB) by blinded treatment group.

AEs identified during monitoring will be classified by a trained medical officer or senior investigator as either serious or non-serious, and as potentially related or unrelated to study medication. If the AE is deemed related, therefore an adverse drug reaction (ADR or AR), further classification will occur regarding whether it is an expected or unexpected AR (see Appendix 5).

As the potential adverse effects of the active study drug in STAREE are well documented, AEs that are expected will not be considered as SAEs unless they meet the criteria for an SAE (section 11.2.3)

Source: AE definition adapted from the National Institute of Aging AE and SAE 2012 guidelines:
https://www.nia.nih.gov/sites/default/files/niaaeandsaeguidelinesfinal011012_0.doc

### 13.2.3 Serious adverse events (SAE)

Serious adverse events (SAEs) or Serious Adverse Drug Reactions (Serious ADRs) are defined as any unfavourable medical occurrence that:

- is fatal


For peer review only - http://bmjopen.bmj.com/site/about/guidelines.xhtml

- is life threatening [i.]
- requires in-patient-hospitalisation or prolongation of existing hospitalisation [ii.]
- causes persistent or significant disability/incapacity
- is a congenital anomaly /birth defect [iii.]
- is a medically important event or reaction [iv.] or
- is an accidental or intentional overdose.

Source: National Statement on Ethical Conduct in Human Research 2007; National Health and Medical Research Council Safety Monitoring and Reporting in Clinical Trials involving Therapeutic Goods November 2016

i. Life Threatening SAE: An SAE is considered life threatening when it places the participant at immediate risk of death from the event as it occurred.

ii. Hospitalisation: A hospitalisation is to be considered an SAE only if it is an official admission (inpatient) with a duration of more than 24hr or a minimum of 2 calendar days where exact time of stay is unavailable.

iii. Not applicable, since STAREE participants are aged 70 years or over at study entry

iv. In the opinion of the responsible investigator (i.e. an event that is not immediately life threatening, does not result in death or hospitalisation, but which may jeopardise the participant or require intervention to prevent one of the other SAEs listed above)

### 13.2.4 Suspected Unexpected Serious Adverse Events (SUSARs)

A SUSAR is defined as a SAE that is:

- not a predefined cardiovascular endpoint (see section 5),
- is unexpected (taking into account drug labelling and the participant history),
- is causally associated with the study medication based on a determination by a medical officer of 'definite', 'probable' or 'possible' using the Naranjo scale.

All SUSARs will be adjudicated by a study medical officer to determine if the event is unexpected in terms of both medication labelling and the participant's history.

A non-endpoint event will be deemed to be a SUSAR if:

- Evidence exists to support the possible association of the event with either or both of the medications (i.e. either the project office or the investigator has assessed the event as possibly causally related to blinded study medication); and
- The event is unexpected in terms of the product labelling for either product; and
- The event is unexpected in terms of the subject's history; and
- The event is serious as defined in section 11.2.

For peer review only


For peer review only - http://bmjopen.bmj.com/site/about/guidelines.xhtml

### 13.3 Safety reporting

In general, only AEs pre-specified to be of specific interest and those that are deemed to be serious will be reportable. AEs of interest are diabetes, myopathy (muscle symptoms) and liver impairment.

All SAEs will be reported from the study sites directly to the study medical officer within 24 hours of their notification to the clinical sites (study centres).

SAEs will be reviewed by the medical officer and adjudicated as related or unrelated to study medication, and as expected or unexpected serious adverse events.

All SAEs and SUSARS will be reported by the study centre to the ethics committees and DSMB according to the following schedule:

- Related and expected SAEs, will be reported to the Human Research and Ethics Committee (HREC)
- Related and unexpected SUSARs will be reported to the HREC within 72 hours and to the RACGP and TGA within 15 days and followed up with detailed reports when available
- Unrelated SAEs will be reported to the STAREE DSMB bi-annually

All SAEs and SUSARs will be reported to the DSMB in summary format by blinded treatment arm (A and B).

The data and safety monitoring reports will be prepared by an independent statistician.

### 13.4 Adverse Events of Interest

#### 13.4.1 New diagnosis of diabetes

Diabetes as diagnosed on the basis of:

1) an annual HbA1c or fasting plasma glucose test using the WHO criteria (a HbA1c value of 6.5% or higher or a fasting plasma glucose of 7.0 mmol/L or higher, with confirmation by a second test within 4 weeks; or

2) community-based testing during usual clinical care (GP or other clinicians e.g. hospitalisation with a hyperglycaemic crisis or classic symptoms of hyperglycaemia and a random plasma glucose test of 11.1 mmol/L and/or commencement of glucose lowering medication for glucose control).

#### 13.4.2 New diagnosis of myopathy

All participants will be advised to discuss a new diagnosis of myopathy with their usual treating GP. The decision to cease or reduce the study medication will be made by the GP in consultation with the participant. The participant will continue in the study on a reduced dose or remain off study medication and have routine follow up (study visits and phone calls) for observational purposes (see section 7.1).


For peer review only - http://bmjopen.bmj.com/site/about/guidelines.xhtml

### 13.4.3 Liver impairment

Liver impairment defined as:

1) an elevated ALT and/or AST across two or more tests (elevations greater than 3x ULN and elevated bilirubin (2x ULN)) and

2) no other demonstrable cause.

For peer review only


For peer review only - http://bmjopen.bmj.com/site/about/guidelines.xhtml

# 14. Adherence to Ethical, Regulatory and Administrative Considerations

## 14.1 Ethical considerations

### 14.1.1 General

This study will be conducted in accordance with the Declaration of Helsinki 1964 as revised in Edinburgh in 2000 and with the National Health & Medical Research Council (NHMRC) Guidelines on Human Experimentation.

### 14.1.2 Ethics Committee Approval

The primary ethics committee for the study is the Monash University Human Research Ethics Committee (Project ID 2787 and 21528).

The study has also been reviewed and approved by the following ethics committees:
Curtin University Human Research Ethics Committee (HR113/2015)
RACGP National Research and Evaluation Ethics Committee (14 - 017)
Tasmanian Health and Medical Research Ethics Committee (H0014918)
University of Newcastle Human Research Ethics Committee (H-2016-0266)

### 14.1.3 Information for participants

Before obtaining consent from the participant they must be informed of the objectives, benefits, risks and requirements of the study, as well as the nature of the study medication. A participant consent form will be provided to every participant prior to baseline visit 1 (posted if time permits) or at baseline visit 1.

### 14.1.4 Informed consent

a) All participants must give their written informed consent at baseline visit 1 to continue in the study.
b) Informed consent is obtained from the participant by the STAREE Field Officers. The study staff should fully inform the participant of all pertinent aspects of the STAREE study by reviewing the study information and consent form. All participants should be informed to the fullest extent possible about the study, in language and terms they are able to understand.
c) Prior to a participant's involvement in the study, the written Informed Consent Form should be signed, name filled in and personally dated by the participant and by the staff member who conducted the informed consent discussion. A copy of the signed and dated written Informed Consent Form will be provided to the participant. The original consent form is to be stored in the participant's individual study file, held by the investigator.
d) The form used for obtaining the participant's informed consent must be the current version that has been reviewed and approved by the appropriate Ethics Committee.

### 14.1.5 Protocol Amendments

All amendments and revisions to the trial protocol will be submitted to the Monash University Human Research Ethics Committee and on approval will be communicated to STAREE investigators and relevant staff through internal processes such as email and group meetings. All updates will be chronologically documented in the current version of the protocol.


For peer review only - http://bmjopen.bmj.com/site/about/guidelines.xhtml

### 14.1.6 Confidentiality

All study-related information will be stored securely at the study sites. All participant information will be stored in locked filing cabinets in areas accessible only by STAREE personnel. All participant CRFs will contain a coded identification number only to maintain participant confidentiality. All records that contain names or other personal identifiers, such as informed consent forms, will be stored separately from study records identified by code number. All local databases will be secured with password-protected access systems. Other forms and lists, that link participant ID numbers to other identifying information will be stored in a separate, locked file in an area with limited access. Hardcopy information will be uploaded into a secure study database housed in the high security Monash University Data Centre at Clayton, Melbourne. All information collected for this project will be retained for a period of no less than 15 years following the completion of the project. Hardcopy information will be shredded and destroyed, and electronic data will be deleted from the secure STAREE database after a period of no less than 15 years after the completion of the project, in accordance with the Monash University Human Research Ethics Committee recommendations for drug trials and Good Clinical Practice Guidelines.

## 14.2 Regulatory considerations

### 14.2.1 Financing

Monash University is the sponsor of the STAREE trial.

STAREE has been awarded project grants from the NHMRC (Australia) (APP1068146 and APP1161503) and from the Heart Foundation (#101663). STAREE MIND and STAREE HEART have been awarded NHMRC grant funding (#2006611 and #1165440).

### 14.2.2 Trial registration

STAREE is registered with clinicaltrials.gov (Identifier NCT02099123). First posted March 28, 2014.

### 14.2.3 Declaration of interest

The STAREE investigators have established a policy regarding Conflict of Interest. The policy aims to protect the integrity of the project's decision-making process and to manage risk. The policy requires full disclosure by all of the investigators of their, and their immediate families, financial relationships with all pharmaceutical and biomedical companies judged to have an active or potential interest in the conduct and outcome of the study. The disclosures of each project investigator will be documented in all publications.

## 14.3 Indemnity

Monash University shall at all times indemnify the study investigators and their staff from claims that may be made against them for any injury sustained by a study participant as a consequence of the follow up for this study as outlined in this protocol. GPs insurers will be asked to indemnify the GP co-investigators who are participating in the study. Ramsay Healthcare will indemnify the study medication.

For peer review only


For peer review only - http://bmjopen.bmj.com/site/about/guidelines.xhtml

### 14.4 Data Access

The Data Access and Publications Committee will oversee the intra-study data sharing process, with input from the STAREE Executive Committee.

Access to STAREE data for analysis, preparation and submission of publications and presentations will be decided according to the STAREE Data Access Policy and the STAREE Publications Policy.

The policies are based on the following principles:

- STAREE data is to be used for the purposes of furthering the public good in relation to health.
- STAREE data is a collaborative resource. Data elements cannot be reserved by researchers for exclusive use.
- STAREE data is to be used for projects that are scientifically, methodologically and ethically sound.
- The privacy of STAREE participants is to be protected.

### 14.5 Governance

*Central Coordinating Centre*

The STAREE Central Coordinating Centre is located at the School of Public Health and Preventive Medicine, Monash University, Melbourne.

*Steering Committee (SC)*

STAREE is overseen and coordinated by a steering committee comprised of Prof Sophia Zoungas (Chair), Monash University; Prof John McNeil, Monash University; Prof Andrew Tonkin, Monash University; Prof Rory Wolfe, Monash University; Prof Christopher Reid, Monash University; Prof Mark Nelson, University of Tasmania; Prof Lawrie Beilin, University of Western Australia; Prof Stephen Nicholls, Monash University; Associate Professor Trevor Chong, Monash University, Prof Geoffrey Cloud, Monash University, Dr Stephanie Ward, Monash University and Prof Anthony Wierzbicki (King's College, UK).

The Steering Committee will provide oversight to the overall policy and direction of the project. It will be responsible for protocol generation and, where appropriate, modification, budgeting and all funding applications. The Steering Committee will meet regularly throughout the planning, execution and data analysis phases of the project, when it will consider reports from the Study Director and the EAC. The Steering Committee will be responsible for all the publications and communications relating to the study.


For peer review only - http://bmjopen.bmj.com/site/about/guidelines.xhtml

**STAREE Governance Structure**

## 14.6 Administrative Organisation

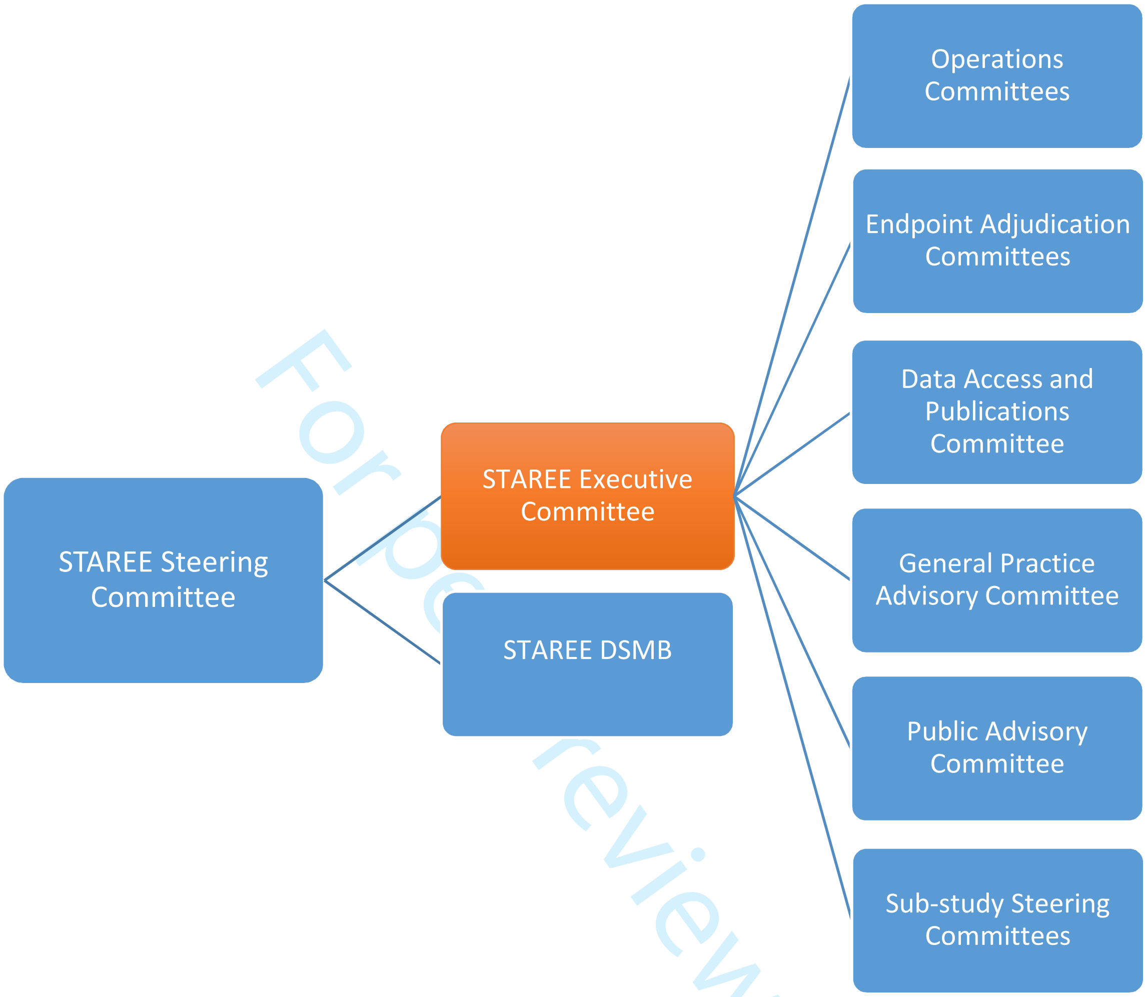


### 14.6.1 Individual and committee responsibilities

*Principal Investigators:* Prof Sophia Zoungas and Prof John McNeil

***STAREE Executive Committee***

The STAREE Executive Committee will be responsible for the overall design, management and conduct of STAREE including preparation of the protocol and revisions, preparation of investigators brochure and case report forms (CRFs), publication of study reports, approving the operational plan for the study, and financial management of the trial. The committee will monitor the progress of recruitment against targets, and if necessary, institute remedial actions. It will also review the rates of adherence to randomised treatments and the data from the quality assurance program.

***National Operations Committee***

The National Operations Committee will be responsible for implementing the study, monitoring the progress of recruitment and ensuring adherence and standardisation through the study. It will also need to ensure the coordination, consistency and transfer of data to the Data Management Centre. The committee will ensure quality control review of


For peer review only - http://bmjopen.bmj.com/site/about/guidelines.xhtml

laboratory data, clinical measurements and data collection, completeness and entry times of data, site monitoring and source documentation. These committees may consist of the Principal Investigator or Project Managers and other key research personnel who will report directly to the Steering Committee.

***Internal Data Monitoring Committee (DMC)***

The STAREE Internal Data Monitoring Committee (DMC) is responsible for reviewing information related to the conduct of the trial and monitoring activity as per STAREE's Monitoring Plan, Monash University's Research Data Management Guidelines and the NHMRC's Australian Code for the Responsible Conduct of Research. The STAREE Internal DMC shall make recommendations to the Monash University STAREE Executive Committee. Recommendations shall include all aspects of Data Monitoring as per the STAREE Monitoring Plan.

DMC members will be appointed by the Executive and Management of the STAREE Clinical Trial and may be involved in the study's collection and management of data, including data monitoring. Appointed committee members will include a Committee Chair, Data Statistician and an individual who has had previous experience with a DMC or experience with trials of medicinal investigational products.

***Data Safety and Monitoring Board***

The Data Safety and Monitoring Board (DSMB) will monitor the establishment and implementation of the STAREE Data Management System and review all safety, data and quality assurance reports for the study. The DSMB chair will report to the Steering Committee of STAREE on data management issues associated with the study and will provide recommendations to the Steering Committee in relation to subject recruitment, event rate tracking, monitoring adverse events and conducting interim analyses. The committee will operate in accordance with the DSMB Charter and Plan.

The DSMB will have responsibility for monitoring quality control of the data, progress of recruitment and safety aspects of the trial. The DMC will present data for review by the DSMB by a blinded treatment arm (e.g., 'A' and 'B'). The DSMB will monitor all-cause mortality rates in the two randomised groups. Additionally one pre-specified interim analysis may occur when an average of 3.25 years of follow up per participant has accrued and >50% of the primary endpoints have occurred. The DSMB will be responsible for reviewing any interim analysis on unblinded data and providing recommendations to the trial executive committee. The DSMB will review deaths, serious adverse events and other endpoint data on a periodic basis. Administrative support will be provided by an independent biostatistician and research assistant.

The DSMB will be provided with data every 6 months or as requested. The DSMB will meet annually for the first 2 years and thereafter will meet twice annually, by teleconference or in-person where possible to review study progress, data quality control, address policy issues and review total mortality data, adverse events and all safety data and monitor the study progress and data quality. The DSMB has expertise related to the conduct of clinical trials *per se* and in the primary care sector, the elderly, epidemiology, biostatistics, clinical pharmacology, clinicians, and cardiovascular disease. Confidentiality will be maintained


For peer review only - http://bmjopen.bmj.com/site/about/guidelines.xhtml

during all phases of the trial including monitoring, preparation of interim results, review, and response to monitoring recommendations.

***Endpoint Adjudication Committees (EACs)***

The Endpoint Adjudication Committees (EACs) will be responsible for developing adjudication criteria and processes to capture key data required for the identification and adjudication of the following study endpoints: All-cause mortality, myocardial infarction, heart failure, stroke, and dementia. The EACs will operated in accordance with the Endpoint Adjudication Committee Charter.

The following EACs will be responsible for the assessment of suspected endpoints through examination of relevant clinical information and for their adjudication according to the pre-specified endpoint definitions.

Death Subcommittee
Dementia Subcommittee
Heart Disease (Myocardial Infarction) Subcommittee
Heart Disease (Heart Failure) Subcommittee
Stroke Subcommittee

The EAC subcommittees will be chaired by specialist clinicians with expertise to oversee adjudication of the relevant endpoints. All EAC members will be selected by the STAREE Executive Committee and will be appointed for the duration of the study. Administrative support, training and source documentation required for EAC members to carry out their roles will be provided by the EAC Coordinator and STAREE study staff.

***Data Access and Publications Committee***

The Data Access and Publications Committee will be responsible for developing and implementing the data access, sub-studies and ancillary studies, and publication policies. It will oversee the processes of receiving, reviewing and approving applications from researchers for STAREE baseline data, for conduct of sub-studies and ancillary studies, and requests for publication, approval for abstracts and submission to conferences. Authorship of publications will be guided by the STAREE data access and publications policies.

***General Practice Advisory Committee***

The General Practice Advisory Committee will advise the National Operations Committee and other committees on aspects of the trial related to family practice.

***Public Advisory Group***

The STAREE Public Advisory Group will be a forum to facilitate the flow of information from the STAREE clinical trial to the public. It will also provide opportunities for members of the group to provide feedback and input from the community perspective on how information is disseminated to the public about the STAREE trial.

***STAREE Healthy Ageing Biobank Steering committee***

Access and utilisation of the biospecimens stored in the STAREE Healthy Ageing Biobank will be governed by the STAREE Healthy Ageing Biobank steering committee. The STAREE

For peer review only


For peer review only - http://bmjopen.bmj.com/site/about/guidelines.xhtml

Healthy Ageing Biobank steering committee will review detailed proposals outlining the research to be undertaken, and access to biospecimens will only be granted if the proposals are assessed to have scientific merit and are adequately funded. Accurate records of the aliquots of each stored biospecimen will be achieved through the use of a Nunc Next Generation CryoTube Scanner and software. The software will be utilised to track each sample which will include storage or retrieval of a specimen by an approved researcher. Any secondary research which uncovers significant genetic information will be notified to the Biobank Steering committee, which will determine who will be notified about these results.

***STAREE MIND Steering committee***

The STAREE MIND Steering Committee will oversee integration of STAREE MIND with the parent trial. It will be responsible for design, management and conduct of STAREE MIND including preparation of the protocol, investigators brochures and case report forms (CRFs), publication of study reports, approval of the operational plan, financial management and will monitor the progress of recruitment. The STAREE MIND committee will report on progress to the STAREE Steering and Executive committees.

***STAREE HEART Steering committee***

The STAREE HEART Steering Committee will oversee integration of STAREE HEART with the parent trial. It will be responsible for design, management and conduct of STAREE HEART including preparation of the protocol, investigators brochures and case report forms (CRFs), publication of study reports, approval of the operational plan, financial management and will monitor the progress of recruitment. The STAREE HEART committee will report on progress to the STAREE Steering and Executive committees.

For peer review only


For peer review only - http://bmjopen.bmj.com/site/about/guidelines.xhtml

## 14. Data Management and Training

### 14.1 Case report forms

All data from assessments conducted during the study will be recorded on an appropriate case report form (CRF) for each participant. Electronic CRFs will be accessible from the STAREE website. The CRFs must be completed during or after each participant visit (or as soon as all data is available, e.g. once pathology results are obtained) and uploaded onto the STAREE web-based portal. Original hard-copy CRFs and other source documents will be stored in the participant's file at the local study site.

CRFs will be kept current to reflect the participant's course throughout the study. Participants will not be identified on the CRF by name. Appropriately coded identification (GP, Practice and Subject Identification Numbers) and an acrostic of the participant's initials will be used.

The data from the CRFs will be uploaded onto the STAREE web-based data management portal. All information collected will be treated in accordance with professional and ethical guidelines. All corrections and alterations of data on the CRFs must be made according to the instructions provided and must be dated and initialled.

### 14.2 Data Management

Data collection and management will be centralised at the STAREE data management centre, Department of Epidemiology and Preventive Medicine, Monash University, Melbourne. Study data collected at local study sites will be entered on electronic CRFs with source documentation retained in the participant file. All information related to potential endpoints will be copied from the original documents sourced through clinical notes and hospital medical records and sent de-identified to the STAREE data management centre. Serious adverse event and endpoint data will be accepted as either a) photocopied clinical records or b) scanned or photographed image files of clinical records. The clinic will retain all original information related to the clinical event or endpoint in the subject files.

Electronic study records will be accessible through the internet at the STAREE web-portal. Access to this website will be made available to registered study staff via password protected log-in procedures.

Monash University implements a defence in depth approach to information security and employs a multitude of controls to protect the study's infrastructure and data. These controls are regularly audited to ensure they meet global best practices and are aligned with ISO 27001 security practices. Data collected as part of the STAREE clinical trial will be stored on University managed secure and resilient infrastructure located in Australia that complies with all applicable data protection and privacy obligations. Some specialist data collected as part of the STAREE clinical trial may be entered into applications that are operated by external third parties. This will only be permitted where all applicable data protection and privacy obligations are in place with those external third parties.

Monash University values the privacy of every individual's personal information and is committed to the protection of that information from unauthorised use and disclosure,


For peer review only - http://bmjopen.bmj.com/site/about/guidelines.xhtml

except where permitted by law. Monash University is subject to and ensures that personal and health information it holds or has access to, is handled in accordance with data protection and privacy legislation, including the Privacy and Data Protection Act 2014 (Vic) and the Health Records Act 2001 (Vic) and where required, the Privacy Act 1988 (Cth) and applicable international regulations.

### 14.3 Data analysis

The data will be analysed by statisticians from the Biostatistics Unit, Department of Epidemiology and Preventive Medicine, Monash University.

Baseline characteristics of the two treatment groups will be tabulated and imbalance defined as a 0.25 standard deviation difference in means (quantitative characteristics) or an odds ratio of 1.5 (binary characteristics).

All comparisons between treatment arms will be on an intention-to-treat basis i.e. according to the group to which participants were randomised and without reference to their actual compliance with assigned treatment.

Each of the co-primary endpoints will be analysed separately in time-to-event analyses. Event rates (time to first event within each endpoint definition) will be compared between groups using a hazard ratio, 95% confidence interval and two-sided p-value from a Cox proportional hazards regression model fitted to the endpoint with censoring for non-events at their most recent study visit and a single covariate being an indicator of the group to which the individual was randomised, statin or placebo. The proportional hazards assumption will be tested for each model.

A gate-keeping procedure will be used to adjust for the multiple testing created by the presence of two co-primary endpoints. First, MACE will be tested at alpha=0.05 and, if the MACE p-value is <0.05 then second, disability-free survival will be tested at alpha=0.05.

Secondary analyses of the co-primary endpoints will be performed using extended Cox proportional hazards regression models based on time(s) to any event within the endpoint definition (for relevant endpoints that can occur more than once).

No statistical adjustment will be made for the multiple secondary endpoints but the reporting of all secondary endpoint analyses will make clear whether any of the co-primary endpoints were statistically significant and will state the number of secondary endpoints proposed a priori in the study protocol.

In the survival analyses, loss to follow up will be considered a censoring event. This equates to an assumption that data is missing at random given the participant’s treatment group and the timing of their loss to follow up. The adequacy of this assumption will be checked in sensitivity analyses that will include both imputation approaches and adjustment for baseline covariates predictive of propensity for dropout. The analyses will be performed using commercial statistical software packages (Stata or SAS).


For peer review only - http://bmjopen.bmj.com/site/about/guidelines.xhtml

### 14.4 Training

All STAREE staff will be trained by senior study staff and study investigators and undertake a competency assessment prior to commencing their specific role.

All Staff will be provided with background information on the rationale of the STAREE trial and the study protocol. They will also be required to familiarise themselves with the key principles of Good Clinical Practice (GCP) and will attend an accredited GCP training course.

All study activities are described in standard operating procedures (SOPs), and staff will be regularly encouraged to refer to SOPs and SOP updates for guidance.

#### 14.4.1 Research Officers

STAREE Research Officers are responsible for and will be trained in the conduct of phone screening for potential study participants, in use of the STAREE database, booking and confirming participant visits, conducting 6 month participant phone calls and labelling and dispatch of medication, receipt of study medication and follow up missing documents for randomisation and endpoint ascertainment.

#### 14.4.2 GP Liaison Officers

STAREE GP Liaison Officers are responsible for engaging with general practices and registering GPs as STAREE co-investigators. They will be provided with practical guidance in conducting GP practice visits to recruit GPs, in data entry, and in searching GP practice databases for potential participants.

#### 14.4.3 Research Field Officers

STAREE Field Officers are responsible for conducting baseline screening visits and annual follow up visits with study participants. They will receive training in database entry, conducting informed consent discussions with potential participants, visit conduct and performing specific study assessments (e.g. weight, height and blood pressure measurement and taking personal and medical histories) and assessing the suitability of a participant to participate in the trial.

Field Officers will observe baseline 1 and 2 and annual visits that are being conducted by a trained Field Officer to become familiar with the visit procedures. Training in the conduct of cognitive assessments will be provided in the form of training videos, SOPs and role plays. Before Field Officers can conduct independent study visits, they will undertake specific training in cognitive assessments with a Senior Field Officer and specialist STAREE Investigator and conduct 3-6 study visits under the supervision from a Senior Field Officer.

Accreditation to perform study visits will be provided subject to satisfactory performance in both the cognitive assessment training and supervised visit conduct. To maintain accreditation, Field Officers will be required to conduct a minimum of 4 visits per month (at least 2 with cognitive assessments) and performance will be reviewed annually.


For peer review only - http://bmjopen.bmj.com/site/about/guidelines.xhtml

## 15. Study Monitoring and Auditing

Monitoring and auditing in STAREE will include monitoring of investigative sites and visit conduct by study staff.  This will involve monitoring of all study documentation and activities including participant files, data quality, administrative activities and endpoint collection as detailed further in the STAREE monitoring plan.

All STAREE study sites will be monitored annually by trained STAREE clinical research Site Monitors, following GCP guidelines. Site monitoring will be undertaken at all STAREE sites to ensure that the study is being conducted according to the protocol and appropriate regulatory requirements, that protocol deviations are appropriately documented and reported, the site complies with safety regulations and that personnel at each site meet GCP obligations.

All field staff involved in the STAREE study will have their activity reviewed by the Visit Conduct Monitor.  Monitoring will ensure that all visit activity is conducted in accordance with the study protocol, relevant SOPs and test administration SOPs.

Study monitors will submit written reports summarising their findings to the internal Data Monitoring Committee (DMC). Where monitoring identifies risks or issues for participant safety or trial processes, appropriate steps will be taken to remedy the issue including re-training of staff and repeat monitoring.

For peer review only



For peer review only - http://bmjopen.bmj.com/site/about/guidelines.xhtml

## 16. Dissemination Plan

The STAREE dissemination plan will ensure that the outputs from the research inform general practice and maximise the benefit to older people.

The key audiences for the STAREE results are:

- General practitioners
- Physicians and other health professionals/clinicians
- STAREE participants
- The general public (including community groups such as Council of the Older Australian, Probus groups etc.)
- Professional organisations (e.g. RACGP)
- Funding organisations (e.g. NHMRC, Monash University)
- Scientific and medical research communities

Representatives of these key stakeholder groups will be invited to take part in discussion of the STAREE findings in relation to current clinical practice and policy and how to best translate the research findings into recommendations for wider audiences.

Multiple channels of communication will be used to disseminate STAREE findings to the wider community. These will include the following:

- Written feedback to STAREE participants and GP co-investigators on the study results and recommendations arising from the study.
- Published academic journal articles, plain English summaries, research summaries for professional journals, newsletters, presentations, and frequently asked questions section will be made accessible on the STAREE website.
- Workshops and presentations at scientific meetings, GP conferences and community and public venues.


For peer review only - http://bmjopen.bmj.com/site/about/guidelines.xhtml

## 17. References

1. Bongaarts J. Human population growth and the demographic transition. Philosophical transactions of the Royal Society of London Series B, Biological sciences. 2009;364(1532):2985-90.
2. Christensen K, Doblhammer G, Rau R, Vaupel JW. Ageing populations: the challenges ahead. Lancet. 2009;374(9696):1196-208.
3. Jacobsen L, Mather M, Lee M, Kent M. America's Ageing Population. Population Bulletin. 2011;66:2-3.
4. Productivity Commission 2011, Caring for Older Australians: Overview, Report No. 53, Final Inquiry Report, Canberra.
5. Zoungas S, Curtis A, Tonkin A, McNeil J. Statins in the elderly: an answered question? Current opinion in cardiology. 2014;29(4):372-80.
6. Jonsson A, Sigvaldason H, Sigfusson N. Total cholesterol and mortality after age 80 years. Lancet. 1997;350(9093):1778-9.
7. Schatz IJ, Masaki K, Yano K, Chen R, Rodriguez BL, Curb JD. Cholesterol and all-cause mortality in elderly people from the Honolulu Heart Program: a cohort study. Lancet. 2001;358(9279):351-5.
8. Volpato S, Leveille SG, Corti MC, Harris TB, Guralnik JM. The value of serum albumin and high-density lipoprotein cholesterol in defining mortality risk in older persons with low serum cholesterol. Journal of the American Geriatrics Society. 2001;49(9):1142-7.
9. Weverling-Rijnsburger AW, Jonkers IJ, van Exel E, Gussekloo J, Westendorp RG. High-density vs low-density lipoprotein cholesterol as the risk factor for coronary artery disease and stroke in old age. Archives of internal medicine. 2003;163(13):1549-54.
10. Foody JM, Wang Y, Kiefe CI, Ellerbeck EF, Gold J, Radford MJ, et al. Long-term prognostic importance of total cholesterol in elderly survivors of an acute myocardial infarction: the Cooperative Cardiovascular Pilot Project. Journal of the American Geriatrics Society. 2003;51(7):930-6.
11. Schupf N, Costa R, Luchsinger J, Tang MX, Lee JH, Mayeux R. Relationship between plasma lipids and all-cause mortality in nondemented elderly. Journal of the American Geriatrics Society. 2005;53(2):219-26.
12. Lewington S, Whitlock G, Clarke R, Sherliker P, Emberson J, Halsey J, et al. Blood cholesterol and vascular mortality by age, sex, and blood pressure: a meta-analysis of individual data from 61 prospective studies with 55,000 vascular deaths. Lancet. 2007;370(9602):1829-39.
13. Stancu C, Sima A. Statins: mechanism of action and effects. Journal of cellular and molecular medicine. 2001;5(4):378-87.
14. Nicholls SJ, Tuzcu EM, Sipahi I, Grasso AW, Schoenhagen P, Hu T, et al. Statins, high-density lipoprotein cholesterol, and regression of coronary atherosclerosis. JAMA : the journal of the American Medical Association. 2007;297(5):499-508.
15. Maron DJ, Fazio S, Linton MF. Current perspectives on statins. Circulation. 2000;101(2):207-13.
16. Greenwood J, Mason JC. Statins and the vascular endothelial inflammatory response. Trends in immunology. 2007;28(2):88-98.
17. Efficacy and safety of statin therapy in older people: a meta-analysis of individual participant data from 28 randomised controlled trials. Lancet. 2019;393(10170):407-15.
18. Cholesterol Treatment Trialists C, Mihaylova B, Emberson J, Blackwell L, Keech A, Simes J, et al. The effects of lowering LDL cholesterol with statin therapy in people at low

For peer review only


For peer review only - http://bmjopen.bmj.com/site/about/guidelines.xhtml

risk of vascular disease: meta-analysis of individual data from 27 randomised trials. Lancet. 2012;380(9841):581-90.
19. Afilalo J, Duque G, Steele R, Jukema JW, de Craen AJ, Eisenberg MJ. Statins for secondary prevention in elderly patients: a hierarchical bayesian meta-analysis. Journal of the American College of Cardiology. 2008;51(1):37-45.
20. Shepherd J, Blauw GJ, Murphy MB, Bollen EL, Buckley BM, Cobbe SM, et al. Pravastatin in elderly individuals at risk of vascular disease (PROSPER): a randomised controlled trial. Lancet. 2002;360(9346):1623-30.
21. Han BH, Sutin D, Williamson JD, Davis BR, Piller LB, Pervin H, et al. Effect of Statin Treatment vs Usual Care on Primary Cardiovascular Prevention Among Older Adults: The ALLHAT-LLT Randomized Clinical Trial. JAMA internal medicine. 2017;177(7):955-65.
22. Glynn RJ, Koenig W, Nordestgaard BG, Shepherd J, Ridker PM. Rosuvastatin for primary prevention in older persons with elevated C-reactive protein and low to average low-density lipoprotein cholesterol levels: exploratory analysis of a randomized trial. Annals of internal medicine. 2010;152(8):488-96, w174.
23. Ridker PM, Lonn E, Paynter NP, Glynn R, Yusuf S. Primary Prevention With Statin Therapy in the Elderly: New Meta-Analyses From the Contemporary JUPITER and HOPE-3 Randomized Trials. Circulation. 2017;135(20):1979-81.
24. Routledge PA, O'Mahony MS, Woodhouse KW. Adverse drug reactions in elderly patients. British journal of clinical pharmacology. 2004;57(2):121-6.
25. Brugts JJ, Yetgin T, Hoeks SE, Gotto AM, Shepherd J, Westendorp RG, et al. The benefits of statins in people without established cardiovascular disease but with cardiovascular risk factors: meta-analysis of randomised controlled trials. Bmj. 2009;338:b2376.
26. Bellosta S, Paoletti R, Corsini A. Safety of statins: focus on clinical pharmacokinetics and drug interactions. Circulation. 2004;109(23 Suppl 1):III50-7.
27. Graham DJ, Staffa JA, Shatin D, Andrade SE, Schech SD, La Grenade L, et al. Incidence of hospitalized rhabdomyolysis in patients treated with lipid-lowering drugs. JAMA : the journal of the American Medical Association. 2004;292(21):2585-90.
28. Davidson MH, Robinson JG. Safety of aggressive lipid management. Journal of the American College of Cardiology. 2007;49(17):1753-62.
29. Sathasivam S. Statin induced myotoxicity. European journal of internal medicine. 2012;23(4):317-24.
30. Hilmer S, Gnjidic D. Statins in older adults. Australian Prescriber. 2013;36(3):79-82.
31. Sattar N, Preiss D, Murray HM, Welsh P, Buckley BM, de Craen AJ, et al. Statins and risk of incident diabetes: a collaborative meta-analysis of randomised statin trials. Lancet. 2010;375(9716):735-42.
32. FDA drug safety communication: Important safety label changes to cholesterol-lowering statin drugs 2012.
33. Cholesterol Treatment Trialists C, Emberson JR, Kearney PM, Blackwell L, Newman C, Reith C, et al. Lack of effect of lowering LDL cholesterol on cancer: meta-analysis of individual data from 175,000 people in 27 randomised trials of statin therapy. PloS one. 2012;7(1):e29849.
34. Ott BR, Daiello LA, Dahabreh IJ, Springate BA, Bixby K, Murali M, et al. Do Statins Impair Cognition? A Systematic Review and Meta-Analysis of Randomized Controlled Trials. Journal of general internal medicine. 2015.


For peer review only - http://bmjopen.bmj.com/site/about/guidelines.xhtml

35. Catapano AL, Graham I, De Backer G, Wiklund O, Chapman MJ, Drexel H, et al. 2016 ESC/EAS Guidelines for the Management of Dyslipidaemias. European heart journal. 2016;37(39):2999-3058.
36. Naci H, Brugts J, Ades T. Comparative tolerability and harms of individual statins: a study-level network meta-analysis of 246 955 participants from 135 randomized, controlled trials. Circulation Cardiovascular quality and outcomes. 2013;6(4):390-9.
37. Bays H, Cohen DE, Chalasani N, Harrison SA. An assessment by the Statin Liver Safety Task Force: 2014 update. Journal of clinical lipidology. 2014;8(3):S47-S57.
38. Lindsley CW. 2012 trends and statistics for prescription medications in the United States: CNS therapeutics continue to hold leading positions. ACS chemical neuroscience. 2013;4(8):1133-5.
39. PBS Information Management Section. Pharmaceutical Policy Branch. Expenditure and prescriptions twelve months to 30 June 2014.
40. Simons LA, Ortiz M, Calcino G. Long term persistence with statin therapy -- experience in Australia 2006-2010. Australian family physician. 2011;40(5):319-22.
41. Trusler D. Statin prescriptions in UK now total a million each week. Bmj. 2011;343:d4350.
42. Sheppard JP, Singh S, Fletcher K, McManus RJ, Mant J. Impact of age and sex on primary preventive treatment for cardiovascular disease in the West Midlands, UK: cross sectional study. Bmj. 2012;345:e4535.
43. Chokshi NP, Messerli FH, Sutin D, Supariwala AA, Shah NR. Appropriateness of statins in patients aged >/=80 years and comparison to other age groups. The American journal of cardiology. 2012;110(10):1477-81.
44. Robinson JG, Booth B. Statin use and lipid levels in older adults: National Health and Nutrition Examination Survey, 2001 to 2006. Journal of clinical lipidology. 2010;4(6):483-90.
45. Morgan TK, Williamson M, Pirotta M, Stewart K, Myers SP, Barnes J. A national census of medicines use: a 24-hour snapshot of Australians aged 50 years and older. The Medical journal of Australia. 2012;196(1):50-3.
46. National Vascular Disease Prevention Alliance. Guidelines for the management of absolute cardiovascular disease risk. 2012.
47. Shepherd J, Cobbe SM, Ford I, Isles CG, Lorimer AR, MacFarlane PW, et al. Prevention of coronary heart disease with pravastatin in men with hypercholesterolemia. West of Scotland Coronary Prevention Study Group. The New England journal of medicine. 1995;333(20):1301-7.
48. Stone NJ, Robinson JG, Lichtenstein AH, Bairey Merz CN, Blum CB, Eckel RH, et al. 2013 ACC/AHA guideline on the treatment of blood cholesterol to reduce atherosclerotic cardiovascular risk in adults: a report of the American College of Cardiology/American Heart Association Task Force on Practice Guidelines. Journal of the American College of Cardiology. 2014;63(25 Pt B):2889-934.
49. East Melbourne: Royal Australian College of General Practitioners. Guidelines for preventative activities in general practice. (2012).
50. NICE. Chronic Kidney Disease - Early identification and management of chronic kidney disease in adults in primary and secondary care. (2014).
51. Collins R, Reith C, Emberson J, Armitage J, Baigent C, Blackwell L, et al. Interpretation of the evidence for the efficacy and safety of statin therapy. The Lancet. 2016;388(10059):2532-61.

For peer review only



For peer review only - http://bmjopen.bmj.com/site/about/guidelines.xhtml

52. Ridker PM, Pradhan A, MacFadyen JG, Libby P, Glynn RJ. Cardiovascular benefits and diabetes risks of statin therapy in primary prevention: an analysis from the JUPITER trial. Lancet. 2012;380(9841):565-71.
53. Ganga HV, Slim HB, Thompson PD. A systematic review of statin-induced muscle problems in clinical trials. American heart journal. 2014;168(1):6-15.
54. Bland RC, Newman SC. Mild dementia or cognitive impairment: the Modified Mini-Mental State examination (3MS) as a screen for dementia. Canadian journal of psychiatry Revue canadienne de psychiatrie. 2001;46(6):506-10.
55. Radloff LS. The CES-D Scale: A Self-Report Depresion Scale for Research in the General Population. Applied Psychological Measurement. 1977;1(3):385-401.
56. Ware JE, Jr., Sherbourne CD. The MOS 36-item short-form health survey (SF-36). I. Conceptual framework and item selection. Medical care. 1992;30(6):473-83.
57. Ross TP. The reliability of cluster and switch scores for the Controlled Oral Word Association Test. Archives of clinical neuropsychology : the official journal of the National Academy of Neuropsychologists. 2003;18(2):153-64.
58. Koss E, Ober BA, Delis DC, Friedland RP. The Stroop color-word test: indicator of dementia severity. The International journal of neuroscience. 1984;24(1):53-61.
59. Shapirol AM, Benedict RHB, Schretlen D, Brandt J. Construct and Concurrent Validity of the Hopkins Verbal Learning Test-Revised. The Clinical Neuropsychologist. 1999;13(3):348-58.
60. Smith A. Symbol Digit Modalities Test (SDMT). Manual (Revised). Western Psychological Services, Los Angeles, 1982.
61. Rosen WG, Mohs RC, Davis KL. A new rating scale for Alzheimer's disease. The American journal of psychiatry. 1984;141(11):1356-64.
62. Lezak MD. Neuropsychological assessment, 3rd ed. New York: Oxford University Press. 1995.
63. Mitrushina M, Boone KB, Razani J, D'Elia LF. Handbook of Normative Data for Neuropsychological Assessment. 2 edn. ed: Oxford University Press Inc.; 2005.
64. Svarstad BL, Chewning BA, Sleath BL, Claesson C. The Brief Medication Questionnaire: a tool for screening patient adherence and barriers to adherence. Patient education and counseling. 1999;37(2):113-24.
65. Morisky DE, Green LW, Levine DM. Concurrent and predictive validity of a self-reported measure of medication adherence. Medical care. 1986;24(1):67-74.
66. Langa KM, Larson EB, Crimmins EM, Faul JD, Levine DA, Kabeto MU, et al. A Comparison of the Prevalence of Dementia in the United States in 2000 and 2012. JAMA internal medicine. 2017;177(1):51-8.
67. Ahmadi-Abhari S, Guzman-Castillo M, Bandosz P, Shipley MJ, Muniz-Terrera G, Singh-Manoux A, et al. Temporal trend in dementia incidence since 2002 and projections for prevalence in England and Wales to 2040: modelling study. Bmj. 2017;358:j2856.
68. Kawas CH, Katzman R. Epidemiology of Dementia and Alzheimer Disease. . Alzheimer Disease. 2nd Ed ed. Philadelphia:: Lippincott Williams & Wilkins; 1999. p. 95-116.
69. Association AP. Diagnostic and Statistical Manual of Mental Disorders. 5th Edition. 5th Edn ed: American Psychiatric Association; 2013.


For peer review only - http://bmjopen.bmj.com/site/about/guidelines.xhtml

# A randomised clinical trial of STAtin therapy for Reducing Events in the Elderly (STAREE)

**PROTOCOL**

**Version 3.0**

**APPENDICES 1-10**

For peer review only

## Appendix 1: STAREE hub locations (update July 2019)

| Site location | STATE | HREC | Start Date | Office location |
|---|---|---|---|---|
| Melbourne and surrounds | VIC | MUHREC | 26/08/2014 | Monash University, 553 St Kilda Rd, Melbourne |
| Geelong | VIC | MUHREC | 05/09/2016 | Monash University, 553 St Kilda Rd, Melbourne |
| Mornington | VIC | MUHREC | 14/09/2016 | Monash University, 553 St Kilda Rd, Melbourne |
| Mildura | VIC | MUHREC | 16/01/2017 | School of Rural Health, Monash, Mildura (closed 2018) |
| Bendigo | VIC | MUHREC | 20/03/2017 | School of Rural Health, Monash, Bendigo |
| Gippsland | VIC | MUHREC | 20/03/2017 | Monash University, 553 St Kilda Rd, Melbourne |
| East Gippsland | VIC | MUHREC | 1/4/2019 | Monash University, 553 St Kilda Rd, Melbourne |
| Warrnambool | VIC | MUHREC | 26/04/2018 | Monash University, 553 St Kilda Rd, Melbourne |
| Albury/Wodonga | VIC | MUHREC | 19/04/2018 | Monash University, 553 St Kilda Rd, Melbourne |
| Hobart | TAS | HREC (Tasmania) network | 01/06/2015 | Menzies Institute for Medical research, University of Tasmania, Hobart |
| Burnie | TAS | HREC (Tasmania) network, | 01/01/2017 | Menzies Institute for Medical research, University of Tasmania, Launceston |
| Launceston | TAS | HREC (Tasmania) network | 01/01/2017 | Menzies Institute for Medical research, University of Tasmania, Launceston |
| Perth and regional WA | WA | HREC (Curtin), | 16/06/2015 | Curtin University, Perth |
| Newcastle | NSW | HREC (Uni Newcastle) | 11/09/2017 | University of Newcastle, Newcastle |
| Tamworth | NSW | HREC (Uni Newcastle) | 28/05/2018 | University of Newcastle, Newcastle |
| Port Macquarie | NSW | HREC (Uni Newcastle) | 22/07/2019 | University of Newcastle, Newcastle |
| Sydney | NSW | HREC (Uni Newcastle) | 21/05/2018 | University of Newcastle, Newcastle |
| Brisbane | Qld | MUHREC | 17/07/2017 | Corporate House, Greenslopes, Brisbane |
| Sunshine coast/Hervey Bay | Qld | MUHREC | 17/07/2017 | Corporate House, Greenslopes, Brisbane |
| Gold Coast | Qld | MUHREC | 17/07/2017 | Corporate House, Greenslopes, Brisbane |
| Toowoomba | Qld | MUHREC | 17/07/2017 | Corporate House, Greenslopes, Brisbane |
| Adelaide | SA | MUHREC | 24/04/2018 | Victoria Park, Dulwich, Adelaide |

For peer review only

## Appendix 2: Brief definitions of STAREE endpoints

| | |
|---|---|
| **PRIMARY ENDPOINTS** | |
| Death, Dementia and Disability | Survival free of dementia or persistent physical disability (as derived from the endpoints of all-cause death, dementia and persistent physical disability) |
| Major Adverse Cardiovascular Event (MACE) | Major Adverse Cardiac Event (MACE); cardiovascular death or acute and non-fatal myocardial infarction (MI) or stroke. |
| **SECONDARY ENDPOINTS** | |
| All-cause mortality | Any cause for death; will be classified based on primary cause such as stroke, MI, heart failure, cancer or other.<br>(All sudden deaths are presumed to be caused by a major cardiovascular event unless confirmed otherwise). |
| Cardiovascular death | Death resulting from a cardiovascular event (eg. MI, stroke, heart failure). |
| Myocardial Infarction | Fatal: Death resulting from MI<br>Non-fatal: Evidence of myocardial necrosis in a clinical setting consistent with myocardial ischemia |
| Stroke | Fatal: Death resulting from stroke<br>Non-fatal: Acute episode of focal or global neurological dysfunction lasting more than 24 hours (unless interrupted by surgery or death) with no apparent cause other than of vascular origin. Imaging is required for classification. |

For peer review only

| | |
|---|---|
| | OR<br><br>Acute episode of focal or global neurological dysfunction lasting less than 24 hours and with no apparent cause other than of vascular origin AND imaging supporting stroke diagnosis. |
| Persistent Physical Disability | Persistent disability or loss of physical function as reflected on any one of the KATZ ADL items (Q9-14) as either "a lot of difficulty" or "unable to do this activity" and/or "Yes" to receiving help, across 2 or more records (> 6 months duration).<br><br>OR<br><br>If it is not possible to obtain a Katz ADL assessment, relevant clinical records such as notification of admission to aged care or community assessment. |
| Dementia (Major Neurocognitive Disorder) | An acquired impairment in cognitive function that is significant (≥2 SD decline relative to population norms) and interferes with independence in everyday activities. [69]<br><br>OR<br><br>Dementia on medical record, without any supporting cognitive data available. |
| Other Cognitive Impairment | All other instances of cognitive impairment (not meeting criteria for "Dementia" above). |
| Approval for permanent residential care | Approval for permanent residential care (based on reporting by an Aged Care Assessment Team). |
| Diagnosis requiring hospitalisation | Hospitalisation extending more than 24 hours or across a minimum of two calendar days. Excludes elective procedures. |
| Heart Failure | A heart failure event, treated in the emergency room or being admitted to hospital and defined as including at least one criterion for heart failure symptoms, at least two for heart failure signs, and imaging or biomarker evidence. |

For peer review only

| | |
|---|---|
| Atrial Fibrillation | Any ECG rhythm strip showing AFib verified by a medical professional<br><br>OR<br><br>Medical professional diagnosed AFib<br><br>OR<br><br>Procedures undertaken for AFib (e.g. cardio version, ablation)<br><br>OR<br><br>Possible AFib<br><br>Irregular heart rhythm during heart rate measurement (e.g. irregular pulse on BP measure )<br><br>AND<br><br>Taking medication used to manage AF (oral anticoagulant agents without other indication (ADD ATC CODE), cardiac glycosides (ATC-Code C01A), antiarrhythmics, Class I and III (ATC-Code C01B)) |
| Hospitalisation for Arterial Revascularisation Procedures | Treatment or surgical intervention to relieve symptoms associated with coronary, arterial disease. Coronary, cerebral or peripheral revascularisation procedures, including percutaneous coronary intervention (PCI), coronary artery bypass graft surgery(CABG), carotid endarterectomy or carotid bypass, other arterial surgery (with or without stenting), other arterial surgery (aortic aneurysm repair, aortobifemoral bypass, femoropopliteal bypass, femoral-tibial bypass and cerebral aneurysm repair) |
| Fatal and non-fatal cancer | Malignant neoplasm confirmed by histopathology or imaging (non-melanoma skin cancers excluded). |
| Quality of life | Determined by the score (0-100) in each of the 8 domains of the SF-36; physical functioning, role limitations due to physical problems, bodily pain, general health perceptions, vitality, social functioning, role-limitations due to emotional problems, and mental health [56]. Based on population norms, a score of 50 is average, 10 point variation is one standard deviation (15 points = 1.5SD). Summary score collapses domains |

For peer review only

| | |
|---|---|
| | into two scores – age bracket specific |
| Cost-effectiveness of statin | To be derived from health economic analysis |

For peer review only

| ADVERSE EVENTS OF INTEREST | BRIEF DEFINITION |
|---|---|
| New diabetes | Diabetes as diagnosed on the basis of:<br>1) an annual HbA1c or fasting plasma glucose test using the WHO criteria (a HbA1c value of 6.5% or higher or a fasting plasma glucose of 7.0 mmol/L or higher, with confirmation by a second test within 4 weeks; or<br>2) community-based testing during usual clinical care (GP or other clinicians e.g. hospitalisation with a hyperglycaemic crisis or classic symptoms of hyperglycaemia and a random plasma glucose test of 11.1 mmol/L and/or commencement of glucose lowering medication for glucose control). |
| Myopathy | Participant description of any muscle symptoms including type, duration and effect of exercise. |
| Liver impairment | Elevated ALT and/or AST across two or more tests (elevations greater than 3x ULN and elevated bilirubin (2x ULN)) and no other demonstrable cause |

For further information see also supplement document *Endpoint Definitions*

For peer review only

# Appendix 3 : Cytochrome P4503 A4 inhibitors clinical recommendations

(updated November 2020)

| CYP450 3A4 Inhibitors | | | | |
|---|---|---|---|---|
| **Brand Name** | **Generic Name** | **Indication** | **Drug Class** | **Study Recommendations** |
| Amiodarone<br>Aratac<br>Cardinorm<br>Cordarone<br>Rithmik | Amiodarone | Angina/Arrhythmia | Antiarrhythmic agent | **Limit study medication to one tablet (20 mg) for the duration of amiodarone therapy.** |
| Magicul<br>Tagamet | Cimetidine | Gastroesophageal reflux disease | Histamine receptor antagonist | **EXCLUDE/ withhold study medication for the duration of cimetidine therapy** |
| Cyclosporin<br>Neoral | Cyclosporin | Transplant rejection, rheumatoid arthritis | Immunosuppresant | **EXCLUDE/ withhold study medication for the duration of cyclosporin therapy** |
| Azol | Danazol | Endometriosis | Gonadotropin Inhibitor | **EXCLUDE/ withhold study medication for the duration of danazol therapy** |
| Cardizem<br>Coras<br>Diltahexal<br>Diltiazem<br>Dilzem<br>Vasocardol | Diltiazem | Angina/Hypertension | $Ca^{+}$ Channel Blocker | **Limit study medication to one tablet (20 mg) for the duration of diltiazem therapy.** |
| Telzir | Fosamprenavir | HIV | Protease Inhibitor | **EXCLUDE** |
| Kaletra | Lopinavir/ Ritonavir | HIV/AIDS | Protease Inhibitor | **EXCLUDE** |
| Anpec<br>Cordilox<br>Isoptin<br>Tarka<br>Veracaps | Verapamil | Angina/Hypertension/Arrhythmia | Ca+ Channel Blocker | **Limit study medication to one tablet (20 mg) for the duration of verapamil therapy.** |
| **CYP450 3A4 Inhibitors** | | | | |

For peer review only

| Brand Name | Generic Name | Indication | Drug Class | Study Recommendations |
|---|---|---|---|---|
| Norvir | Ritonavir | HIV/AIDS | Protease inhibitor and potent CYP3A4 inhibitor | **EXCLUDE** |
| Included in:<br>Prezcobix<br>Evotaz<br>Stribild<br>Genvoya<br>Tybost | Cobicistat | HIV/AIDS | Potent CYP3A4 inhibitor | **EXCLUDE** |
| Maviret | Glecaprevir/ Pibrentasvir | Hepatitis C | Protease inhibitor | **EXCLUDE/ withhold study drug for the duration of protease inhibitor therapy (usually 12 weeks)** |
| Vosevi | Sofosbuvir/ Velpatasvir/ Voxilaprevir | Hepatitis C | Protease inhibitor | **EXCLUDE/ withhold study drug for the duration of protease inhibitor therapy (usually 12 weeks)** |
| Prevymis | Letermovir | Cytomegalovirus (CMV) prevention | CMV DNA terminase complex inhibitor | **EXCLUDE/ withhold study drug for the duration of letermovir therapy** |

**Antibiotics and Antifungals for Long Term Oral Use Only**

| Brand Name | Generic Name | Indication | Drug Class | Study Recommendations |
|---|---|---|---|---|
| Clarac<br>Clarihexal<br>Clarithro<br>Clarithromycin<br>Kalixocin<br>Klacid<br>Nexium Hp7 Combination pack | Clarithromycin | Bacterial infection | Macrolide antibiotic | **Limit study medication to one tablet (20 mg) for the duration of clarithromycin therapy.** |
| EES<br>E-mycin<br>Eryc<br>Erythromycin | Erythromycin | Bacterial infection | Macrolide antibiotic | **Limit study medication to one tablet (20 mg) for the duration of erythromycin therapy.** |

For peer review only

| | | | | |
|---|---|---|---|---|
| Diflucan<br>Fluconazole | Fluconazole | Fungal infection | Antifungal | **Limit study medication to one tablet (20 mg) for the duration of fluconazole therapy.** |
| Lozanoc<br>Sporanox | Itraconazole | Fungal infection | Antifungal | **EXCLUDE/ withhold study medication for the duration of itraconazole therapy** |
| Nizora | Ketoconazole | Fungal infection | Antifungal | **EXCLUDE/ withhold study medication for the duration of ketoconazole therapy** |
| Fucidin | Fusidic acid or sodium fusidate | Bacterial infection | Antibiotic | **EXCLUDE/ withhold study medication for the duration of and one week after completion of fusidic acid therapy** |


1. www.drugbank.ca
2. www.medicines.org.uk
3. www.drugs.com/pro/atorvastatin.html
4. www.medsafe.govt.nz
5. Stockley's Drug Interactions


For peer review only

## Appendix 4: Medication compliance questions

1. Have you been taking study medication? Yes / No
2. If Yes, how may study tablets do you take each day? 1 tablet / 2 tablets / other___
3. In an average week, how many days do you take study medication? _____ days
4. Do you ever miss taking your study medications? Yes / No
5. Comments (i.e. reasons for occasionally missing or altering dose) ________

OR, if they answer NO to question 1, then:

2. What reason(s) did you stop taking study medication? ______________
3. What date did you stop taking study medication?
4. Who made this decision? GP or PCP / Self / Other

For peer review only

## Appendix 5: Reporting of AEs and SAEs

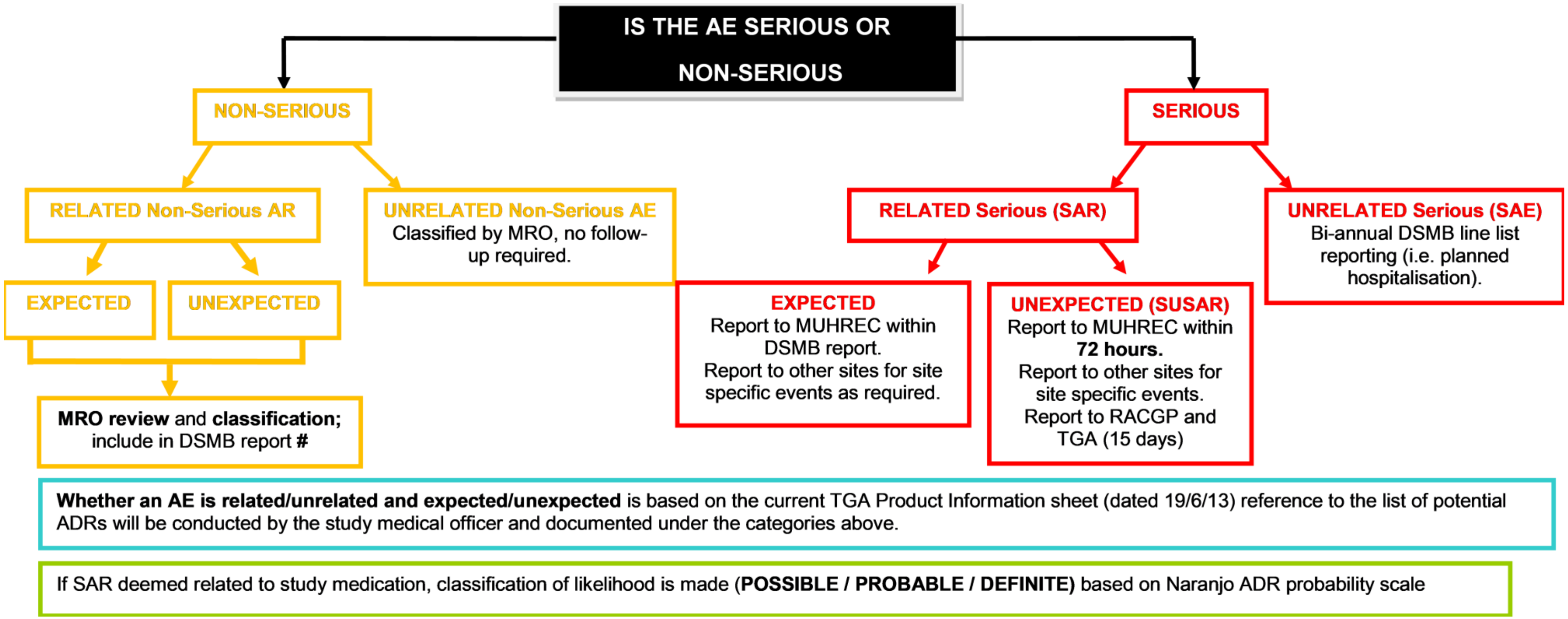

| | |
|---|---|
| **AE** Adverse Event | Any unfavourable medical occurrence |
| **SAE** Serious Adverse Event | An event that:<br>❖ requires hospitalisation/prolongation of existing hospitalisation<br>❖ results in death<br>❖ is life threatening<br>❖ results in persistent or significant disability/incapacity<br>❖ is a congenital anomaly/birth defect; or<br>❖ is a medically important event or reaction |
| **SAR** Serious Adverse Drug Reaction | A SAE that is deemed related to study medication (note serious ADR also used; also non-serious ADR) |
| **SUSAR** Serious, Unexpected, Suspected Adverse Reaction | An **unexpected** SAE where there is **some degree of probability** that the event is related to the study medication. |
| **PI** Principal Investigator | Principal Investigator or a co-investigator delegated this responsibility |
| **Comment from the PI** | A statement about the significance of the information, the possible impact on study participants, and action taken or recommended |

***DSMB report: submitted six-monthly: includes line list of non-serious AEs of interest # (muscle symptoms, changes to memory) and summary of related SAEs, both expected and unexpected; the former based on the current Product Information sheet; Reports from DSMB to be forwarded to all committees & ADR select section to TGA***

For peer review only

# Appendix 6: Assessment tools

| Outcome Measure | Tool | | Type | Reference/s |
|---|---|---|---|---|
| Global cognitive function | 3MS | Modified Mini Mental State Test | Questionnaire | Tombaugh TN et al. (2005). Test-retest reliable coefficients and 5-year change scores for the MMSE and 3MS. Arch Clin Neuropsychol. 2005;20:485-503<br><br>Ryan J, Woods RL, Britt C, Murray AM, Shah RC, Reid CM, Kirpach B, Wolfe RS, Nelson MR, Lockery JE, Orchard SG, Trevaks RE, McNeil JJ, Storey E; ASPREE Investigator Group. Normative performance of healthy older individuals on the Modified Mini-Mental State (3MS) examination according to ethno-racial group, gender, age, and education level. Clin Neuropsychol. 2019 May;33(4):779-797. doi: 10.1080/13854046.2018.1488996. Epub 2018 Jul 5. PMID: 29976121; PMCID: PMC6810645. |
| Verbal fluency + language | COWAT | Controlled Oral Word Association Test | Questionnaire | Ross, T.P. The reliability of cluster and switch scores for the Controlled Oral Word Association Test. Archives of clinical neuropsychology. 2003;18(2);153-164 |
| Executive functioning + short term memory | Stroop | Victoria Stroop Color-Word Test | Questionnaire | Koss E, et al. The Stroop color-word test. Int J Neurosc. 1984;24(1):53-61.<br><br>Carone, D. A. (2007). E. Strauss, E. M. S. Sherman, & O. Spreen, A Compendium of Neuropsychological Tests: Administration, Norms, and Commentary: A Review of: "(3rd ed.), Oxford University Press, New York, 2006." [Review of E. Strauss, E. M. S. Sherman, & O. Spreen, A Compendium of Neuropsychological Tests: Administration, Norms, and Commentary: A Review of: "(3rd ed.), Oxford University Press, New York, 2006. |
| Memory function | HLVT-R<br><br>Or<br>Hopkins | Hopkins Verbal Learning Test Revised | Questionnaire | Shapirol, A.M. Benedict, R.H.B. Schretlen, D. Brandt, J. Construct and Concurrent Validity of the Hopkins Verbal Learning Test-Revised. The Clinical Neuropsychologist. 1999; 13(3): 348-358.<br><br>Ryan, J., Woods, R. L., Murray, A. M., Shah, R. C., Britt, C. J., Reid, C. M., Wolfe, R., Nelson, M. R., Lockery, J. E., Orchard, S. G., Trevaks, R. E., Chong, T. J., McNeil, J. J., & Storey, E. (2020). Normative performance of older individuals on the Hopkins Verbal Learning Test-Revised (HVLT-R) according to ethno-racial group, gender, age and education level. The Clinical Neuropsychologist, ahead-of-print(ahead-of-print), 1–17 |
| Executive Functioning | TMT | Trail Making Test | Questionnaire | Reitan RM. Validity of the Trail Making test as an indicator of organic brain damage. Percept Motor Skills 1958; 8: 271-276.<br><br>Lezak MD (1995) Neuropsychological assessment, 3rd edn. New York: Oxford University Press.<br><br>Corrigan JD, Hinkeldey MS. Relationships between Parts A and B of the Trail Making Test. J Clin Psychol 1987;43:402- |

For peer review only

| | | | | |
|---|---|---|---|---|
| | | | | 9.<br><br>Mitrushina, M., Boone, K.B., Razani, J. & D'Elia, L.F. Handbook of Normative Data for Neuropsychological Assessment, Appendix 4M (Page 653), (Oxford University Press Inc., 2005). |
| Processing speed | SDMT | Symbol Digit Modalities Test | Questionnaire | Smith A. Symbol Digit Modalities Test (SDMT). Manual (Revised). Western Psychological Services, Los Angeles, 1982.<br><br>Kiely, K.M., Butterworth, P., Watson, N. & Wooden, M. The Symbol Digit Modalities Test: Normative data from a large nationally representative sample of Australians. Archives of clinical neuropsychology : the official journal of the National Academy of Neuropsychologists 29, 767-775 (2014).<br><br>Ryan J, Woods RL, Britt CJ, Murray AM, Shah RC, Reid CM, Wolfe R, Nelson MR, Orchard SG, Lockery JE, Trevaks RE, Storey E; behalf of the ASPREE Investigator Group on. Normative Data for the Symbol Digit Modalities Test in Older White Australians and Americans, African-Americans, and Hispanic/Latinos. J Alzheimers Dis Rep. 2020 Aug 4;4(1):313-323. doi: 10.3233/ADR-200194. PMID: 33024939; PMCID: PMC7504980. |
| Visual agnosia | Visual Agnosia | Lurian Overlapping figures | Questionnaire | Lezak, M.D. (1995) Neuropsychological assessment, 3rded. New York: Oxford University Press. |
| Delirium | CAM | Confusion Assessment Method | Questionnaire | Inouye, S.K., van Dyk, C.H., Alessi, C.A., Balkin, S., Siegal, A.P., & Horwitz, R.I. Clarifying confusion: The Confusion Assessment Method. American College of Physicians 1990; 113: 941-948.<br><br>The Confusion Assessment Method (CAM). Training Manual and Coding Guide (2003). Yale University School of Medicine. |
| Quality of Life | SF-36 | Short Form-36 item Health Survey | Questionnaire | Ware, J.E., Jr., & Sherbourne, C.D. "The MOS 36-Item Short-Form Health Survey (SF-36): I. Conceptual Framework and Item Selection,". Medical Care, 30:473-483, 1992. |
| Physical disability | Katz ADL | Katz Index of Independence in Activities of Daily Living | Questionnaire | Katz S, Apkon CA. A measure of primary sociobiological functions. Int J Health Serv 1976;6:493-507. |
| Functional impairment | ADCS-IADL or B-ADL | Activities of Daily Living | Questionnaire | Hindmarch I, Lehfeld H, de Jongh P & Erzigkeit H. Dement Geriatr Cogn Disord 1998;9(suppl 2):20-26<br><br>Lawton, M.P. Gerontologist, 1969. **9**(3): p. 179-186.<br><br>Njegovan, V. Journal of Gerontology, 2001. **56A**(10): p. M638–M643. |
| Memory impairment<br><br>Part A – | ADAS-Cog | the Alzheimer Disease Assessment Scale – Cognitive (ADAS-Cog) | Questionnaire | Diagnostic and statistical manual of mental disorders. 4th ed. Text Revision. 2000, Washington, DC: American Psychiatric Association.<br><br>Rosen, W.G., Mohs, R.C., & Davis, K.L. A new rating scale for Alzheimer's disease. Am J Psychiatry 984;141(11):1356- |

For peer review only

| | | | | |
|---|---|---|---|---|
| Constructional Praxis<br><br>Part B - Ideational Praxis | | subscale | | 1364.<br><br>Fillenbaum, Gerda G, Burchett, Bruce M, Unverzagt, Frederick W, Rexroth, Daniel F, & Welsh-Bohmer, Kathleen. (2011). Norms for CERAD Constructional Praxis Recall. Clinical Neuropsychologist, 25(8), 1345–1358. https://doi.org/10.1080/13854046.2011.614962 |
| Depressive symptoms (score > 8 trigger) | CES-D-10 | Center for Epidemiological Studies Depression Scale – 10 item | Questionnaire | Radloff, L.S. The CES-D Scale: A Self-Report Depression Scale for Research in the General Population. Applied Psychological Measurement. 1977; 1(3): 385-401<br><br>Irwin M, Artin KH, Oxman MN. Screening for depression in the older adult: criterion validity of the 10-item Center for Epidemiological Studies Depression Scale (CES-D). Arch Intern Med. 1999; 159(15):1701-1704. |
| Performance of everyday activities | B-ADL | Bayer Activities of Daily Living | | Hindmarch I, Lehfeld H, de Jongh P, Erzigkeit H: The Bayer Activities of Daily Living Scale (B-ADL). Dement Geriatr Cogn Disord 1998;9(suppl 2):20-26. doi: 10.1159/000051195 |
| Physical activity | IPAQ-E | International Physical Activity Questionnaire modified for the elderly | Questionnaire | Hurtig-Wennlöf A, Hagströmer M, Olsson LA. The International Physical Activity Questionnaire modified for the elderly: aspects of validity and feasibility. Public Health Nutr. 2010 Nov;13(11):1847-54. doi: 10.1017/S1368980010000157. Epub 2010 Mar 3. |

For peer review only

# PARTICIPANT CONSENT FORM

*MONASH UNIVERSITY*

## Appendix 7: STAREE participant information sheet/consent form

For peer review only


For peer review only - http://bmjopen.bmj.com/site/about/guidelines.xhtml

# PARTICIPANT CONSENT FORM

*MONASH UNIVERSITY*

For peer review only


For peer review only - http://bmjopen.bmj.com/site/about/guidelines.xhtml

# PARTICIPANT CONSENT FORM

*MONASH UNIVERSITY*

# PARTICIPANT INFORMATION SHEET/CONSENT FORM

*MONASH UNIVERSITY*

**Full Project Title:** A randomised clinical trial of STAtin therapy for Reducing Events in the Elderly (STAREE)

For peer review only


For peer review only - http://bmjopen.bmj.com/site/about/guidelines.xhtml

# Participant Consent Form

*Monash University*

This participant information and consent form is 8 pages long. Please make sure you have all the pages.

This document tells you about the research project. It explains the tests and treatments involved. Knowing what is involved will help you decide if you want to take part.

Please read this information carefully. Ask questions about anything that you do not understand or want to know more about. Before deciding whether or not to take part, you may want to talk about it with a relative, friend or your local doctor.

If you decide to take part in the research project, you will be asked to sign the consent section. By signing it you are telling us that you:

- Have read the participant information sheet and consent form;
- Understand what you have read;
- Agree to take part in the research project;
- Agree to take the study medication as instructed for an average of 5 years;
- Agree to undergo the study assessments as described in this document;
- Agree to attend clinic visits annually;
- Agree to your medical records being accessed; and
- Agree to having your data accessed via data linkage (bringing together information from different sources such as registries, hospitals etc.).

You will be given a copy of this Participation Information and Consent Form to keep.

For any queries, information on the project or to speak to the research team please call **1800 770 664**.

### Why are you being given this Participant Information and Consent Form?

We would like to invite you to participate in this research project as you are aged 70 or older and do not have current serious disease or disability.

### Who is running this study?

The study is run by the School of Public Health and Preventive Medicine, Monash University. Your general practitioner (GP) may be a co-investigator and may receive up to $100 per patient for the use of their practice facilities.

### What is the study about?

Statins (HMG-CoA reductase inhibitors) are a class of drug that block the action of a chemical (HMG-CoA reductase) in the liver that is needed to make cholesterol. This action lowers cholesterol and lipid levels in the blood. The health benefits of statins may include reducing the number of new cases of heart disease, stroke and dementia. However, there



For peer review only - http://bmjopen.bmj.com/site/about/guidelines.xhtml

# PARTICIPANT CONSENT FORM

*MONASH UNIVERSITY*

are side-effects from statins that are still being debated. We are investigating if the benefits of statins outweigh the risks in healthy people aged 70 and over. We aim to determine if taking statins prevents a decline in the physical and mental abilities associated with ageing which may lead to long term residential care and a loss of independence.

STAREE will be conducted in general practices across Australia and aims to recruit at least 10,000 participants nationally. Half of the participants will receive statin therapy and half will receive a placebo tablet (tablet with inactive ingredients). We will then observe the 2 groups over an average 5 year period to see what differences there are for levels of physical disability, death, heart attacks, diabetes, dementia and general quality of life. We will collect this data from you, your GP's records, and any hospital or specialist you visit.

You will also be asked to fill out a separate consent form authorising access to your complete Medicare, Pharmaceutical Benefits Scheme (PBS), Repatriation Pharmaceutical Benefits Scheme (RPBS) data and various health data sets. Medicare collects information on your doctor visits and the associated costs, while the PBS/RPBS collects information on the prescription medications you have filled at pharmacies. This consent form is sent securely to the Department of Human Services which holds this information confidentially. We also ask for your consent to access other health data information such as hospital discharge summaries and national registry health information (i.e. National Death Index).

Providing us with this information helps us to assess your health status throughout the study.

### WHAT ARE WE ASKING YOU TO DO?

**Baseline Visit 1**: If you agree to participate, you will be asked to give your consent by signing this form. After you give your consent to enter this study, research staff will briefly look at your medical records to ensure there are no underlying medical conditions that would make you ineligible for the study, and to check any pathology results from the previous 6 months to ensure no tests are repeated unnecessarily. We will then measure your blood pressure, heart rate and ask you some questions about your general health and medical history, your family medical history, current medications, and your daily activities. We will ask you to complete other questionnaires that will assess your mood and involve some thinking tasks. You will have a brief physical examination including measuring your weight, height, waist and hip circumference.

We will then ask you to take a placebo tablet of study medication once daily for four weeks. This will help get you in the routine of taking a tablet every day.

The research staff will also ask you to attend a local pathology service for a fasting blood test and a urine test. Approximately 12 ml (about 2 teaspoons) of blood will be taken and this sample will be used to check your cholesterol, liver and kidney function and sugar levels. Approximately 20 ml of urine will also be collected. This will provide information about your kidney function and cardiovascular health.


For peer review only - http://bmjopen.bmj.com/site/about/guidelines.xhtml

# PARTICIPANT CONSENT FORM

*MONASH UNIVERSITY*

Based on these test results, the physical examination and your medical and medication history, we will ask you to see your GP to discuss if it is appropriate for you to continue in the study and attend baseline visit 2.

**Baseline Visit 2**: At the second visit, if you have taken your study medication, and you satisfy all other eligibility criteria, you will be randomly assigned to take two tablets of either statin (atorvastatin 40 mg) or placebo (a dummy tablet), at the same time every day with a drink of water for an average of 5 years. You will need to commit to taking the study medication regularly as well as attend all the study visits.

The medication you receive will be determined by a computer program. Neither you, research staff, nor your GP will know if you are taking the active (statin) or placebo medication. You will have equal chance of receiving either statin or placebo.

In the event of an emergency, any doctor can find out what treatment you are taking if they need to know.

During this visit, you will be asked to complete tasks that measure thinking and memory function and a questionnaire on your health and physical ability.

**Annual clinic visit:** Each year, you will be asked to attend a study visit or a phone call where staff will conduct similar activities as in your baseline visits. You will be asked to attend this visit, even if you stop taking the study medication.

**6 monthly phone call**: Every six months the research staff will phone you and ask you about your recent medical history, medications and how you are going on the trial.

### CAN I LEAVE THE STUDY?

Participation in any research project is voluntary. If you do not wish to take part, you do not have to. If you decide to take part and later change your mind, you can change your level of involvement with the project at any stage.

While full participation in the study means that you are taking the study medication and having study visits and phone calls, there are other levels of participation that you may consider. Stopping or discontinuing study medication (even for a short time period) does not mean you have to stop your involvement in the trial.

The other levels of involvement are:

- Stopping your study medication but still attending study visits and receiving phone calls
- Stopping your study medication and also stopping study visits and phone calls
- Stopping your study medication, stopping study visits and phone calls and stopping collection of data from your medical records


For peer review only - http://bmjopen.bmj.com/site/about/guidelines.xhtml

# PARTICIPANT CONSENT FORM

*MONASH UNIVERSITY*

Please note, if you decide to not continue your participation in the study, the data that has been collected up to this point will remain part of the study data. It is important that this data remains in the study because if it is excluded the safety of the research project may be compromised. Retention of this data is also important to ensure appropriate reporting to the study Data and Safety Monitoring Board.

If you do decide to stop your involvement from the study, we may ask you about your reasons. This information is important for the conduct of future trials.

Regardless of your level of participation or if you stop your involvement completely from the study, it will not affect your routine treatment, your relationship with your GP or your relationship with Monash University.

**WHAT ARE THE POSSIBLE BENEFITS OF TAKING PART?**

We cannot guarantee that you will receive any health benefits from this research. However, some participants may experience health benefits from being placed on atorvastatin. These benefits may include the following:

- Reductions in the levels of cholesterol in the blood,
- Improved health of blood vessels throughout the body,
- A reduced risk of cardiovascular disease (e.g.) heart attack or stroke).

In addition, some scientific studies have suggested that statins may be associated with reduced risk of dementia, depression, and cancer. However, these effects are less certain.

If the study shows that there are net health benefits from statin therapy, it may enable this treatment to be available to more people in the future. We will also inform your doctor of any abnormal findings from your measurements, and memory and thinking tasks, so that they can discuss this with you.

**WHAT ARE THE POSSIBLE RISKS AND DISADVANTAGES OF TAKING PART?**

Atorvastatin, the statin used in this study, is an established safe and well-tolerated medication to lower cholesterol. However, like all medications, atorvastatin can cause side-effects. These side-effects do not affect everyone. You may experience no side effects. If you do experience side effects from the study medication they may be mild, moderate or severe. Many side effects are temporary or reversible, however, some side effects can be serious, long lasting or permanent. If this occurs, your doctor may ask you to stop taking the medication.

You could experience side effects that researchers do not expect or do not know about and these side effects could be serious.


For peer review only - http://bmjopen.bmj.com/site/about/guidelines.xhtml

# PARTICIPANT CONSENT FORM

*MONASH UNIVERSITY*

Tell your doctor immediately about any new or unusual symptoms that you experience. Your doctor will monitor any side effects that do occur and will discuss the best way of managing them with you.

The reported side effects of atorvastatin:

- Common side effects (occur in 1-10% of patients): headache, muscle pain, constipation, feeling sick and increase in blood sugar levels.
- Uncommon side effects (0.1-1%): skin rash, itching and hives.
- Rare side effects (0.01-0.1%): hypersensitivity (allergic) reactions and severe muscle disorders (myopathy).

You may also refer to the Product Information Sheet provided to you at your first study visit.

You should not drink more than one or two small glasses of grapefruit juice each day as large quantities of grapefruit juice can change the effects of atorvastatin. You should also avoid consuming excessive amounts of alcohol on a regular basis while taking the study medication.

If you suffer any injuries or complications as a result of this research project, you should contact the study team as soon as possible so they can assist with arranging appropriate medical treatment. If you are eligible for Medicare, you can receive any medical treatment required, free of charge, as a public patient in any Australian public hospital.

### WHAT IF NEW INFORMATION ARISES DURING THE RESEARCH PROJECT?

Sometimes during the course of a research project, new information becomes available about the treatment being studied. If this happens, your doctor will tell you about it and discuss with you whether you want to continue in the research project. If you decide to withdraw, your doctor will continue with your regular health care. If you decide to continue in the research project you will be asked to sign an updated consent form.

Also, on receiving new information, your doctor may consider it to be in your best interest to withdraw you from the research project. If this happens they will explain the reasons and arrange for your regular health care to continue.

### WHAT WILL HAPPEN TO INFORMATION ABOUT ME?

Any information collected in this study will not be published in any manner that could identify you as an individual, during or after the conclusion of this project. We will only publish group data. Your personal and health information which we will collect as part of this study is reported on special forms by the research staff. Your name is not recorded on these forms. You are only identified by your initials and a study-specific number assigned by research staff.

By signing the attached consent form, you are agreeing to the release of your medical records held by your doctor or hospital. This information will be collected, stored and


For peer review only - http://bmjopen.bmj.com/site/about/guidelines.xhtml

# PARTICIPANT CONSENT FORM

*MONASH UNIVERSITY*

analysed only for the purposes of this study, and will be limited to the medical details related to this study. Any information collected from your clinic about you will be overseen by the clinic GPs.

Medical tests and results collected as part of the study may reveal an underlying condition. Any results outside the normal range (such as elevated blood pressure or abnormal pathology) will be communicated to your GP.

Final results of the project and the arm of the trial you were part of, will be communicated to you at the conclusion of the trial.

Monash University implements a defence in-depth approach (multiple layers of security) to information security and employs a multitude of controls to protect our infrastructure and data. These controls are regularly audited to ensure they meet global best practices and are aligned with ISO 27001 security practices. Data collected as part of the STAREE clinical trial will be stored on University-managed, secure and resilient infrastructure located in Australia that complies with all applicable data protection and privacy obligations. Some specialist data collected as part of the STAREE clinical trial may be entered into applications that are operated by external third parties. This will only be permitted where all applicable data protection and privacy obligations are in place with those external third parties.

All information collected for this project will be retained for a period of no less than 15 years following the completion of the project. Hardcopy information will be shredded and destroyed and electronic data will be deleted from the secure STAREE database after a period of no less than 15 years after the completion of the project, in accordance with the Monash University Human Research Ethics Committee recommendations for drug trials and Good Clinical Practice Guidelines.

### WHO IS FUNDING THE RESEARCH?

The main funding body of STAREE is the National Health and Medical Research Council of Australia.

### WHO HAS REVIEWED THE RESEARCH PROJECT?

All research in Australia involving humans is reviewed by an independent group of people called a Human Research Ethics Committee (HREC). The ethical aspects of this project have been approved by the Monash University Human Research Ethics Committee (MUHREC) and The Royal Australian College of General Practitioners National Research and Evaluation Ethics Committee (NREEC).

This project will be carried out according to the *National Statement on Ethical Conduct in Human Research (2007)*. This statement has been developed to protect the interests of people who agree to participate in human research studies.

### FURTHER INFORMATION AND WHO TO CONTACT



For peer review only - http://bmjopen.bmj.com/site/about/guidelines.xhtml

# Participant Consent Form

*Monash University*

Please ask the research staff member if you have any further questions. You can also consult your own doctor if you have any concerns during the course of the research.

Specific enquiries related to this study can also be made to the following person at STAREE:

Name: Professor Sophia Zoungas (Monash University):
Phone: +61 3 9903 0711
E-mail: sophia.zoungas@monash.edu

For matters relating to research at the site at which you are participating, or if you have any complaints about any aspect of the project, the way it is being conducted or any questions about being a research participant in general, you may contact the Executive Officer, Monash University Human Research Ethics (MUHREC) quoting project 21528:

Executive Officer
Monash University Human Research Ethics Committee (MUHREC)
Room 111, Building 3e

Research Office
Monash University VIC 3800
Tel: +61 3 9905 2052
Email: muhrec@monash.edu
Fax: +61 3 9905 3831


For peer review only - http://bmjopen.bmj.com/site/about/guidelines.xhtml

# Participant Consent Form

*Monash University*

**Title** A randomised clinical trial of STAtin therapy for Reducing Events in the Elderly (STAREE)

## Declaration by participant

I have read the Participation Information Sheet or someone has read it to me in a language that I understand.

I understand the purposes, procedures and risks of the research described in the project.

I understand I will be asked to take a study medication for the duration of the project, and be contacted on a regular basis by phone or in person.

I give permission for my doctors, other health professionals, hospitals or laboratories to release information to Monash University concerning my health and treatment for the purposes of this project. I understand that such information will remain confidential.

I give permission for information that I provide for this research project to be linked to various health datasets for the purposes of the project's outcomes and future research.

I have had an opportunity to ask questions and I am satisfied with the answer I have received.

I freely agree to participate in this research project as described and understand that I am free to change my level of participation or withdraw at any time during the study without affecting my future health care.

I understand that if I decide to discontinue the study medication, I may be asked to attend follow-up visits to allow collection of information regarding my health status.

I understand that if I choose to withdraw from the project that any data that has been collected prior to my withdrawal will be retained in the study database.

I understand I will be given a signed copy of this document to keep.

Name of participant (please print)________________________________________

Signature____________________________________Date__________________

## Declaration by researcher

I have given a verbal explanation of the research project, its procedures and risks and I believe that the participant has understood that explanation.

Name of researcher (please print)________________________________________

Signature____________________________________Date__________________



For peer review only - http://bmjopen.bmj.com/site/about/guidelines.xhtml

# PARTICIPANT CONSENT FORM

*MONASH UNIVERSITY*

## Appendix 8: List of relevant documents.

- DSMB Charter and Plan
- Endpoint Adjudication Committee Charter
- Monitoring plan
- Register of interests for STAREE Investigators and DSMB members

For peer review only


For peer review only - http://bmjopen.bmj.com/site/about/guidelines.xhtml

# PARTICIPANT CONSENT FORM

*MONASH UNIVERSITY*

## Appendix 9: COVID pandemic response

**As a result of government and workplace restrictions due to the COVID-19 pandemic and to ensure safety of staff and participants, the following changes were made to the STAREE clinical trial following ethics approval:**

13th March 2020: Risk management plan formed and approved by STAREE Executive committee

16th March 2020: Invitation letters to participants suspended

20th March 2020: Face to face visits ceased. GP recruitment suspended.

23rd March 2020: Annual Visit 1 was modified to be conducted as a phone call visit

24th April 2020: Baseline Visit 2 was modified to be conducted as a phone call visit

18th May 2020: Annual Visits 2 and 4 were modified to be conducted as phone call visits

27th May 2020: Resumed sending invitation letters to participants. Baseline Consent visit (BV-C) instituted as a phone/zoom call.

July 2020: Physically distanced face to face BV1 visits (SD-BV1) commenced (phone visits offered as alternative if participant preference).

**Changes to the schedule:**

To provide a physically distanced or phone visit the following modifications were made to the visit schedule:

Physically distanced BV1 (SD-BV1): The consent procedure was moved to a separate phone/zoom visit called Baseline Consent (BV-C). Some sections of the usual Baseline 1 visit were moved to the Baseline 2 visit.

3MS: to be completed in full during in-person SD-BV1 or to a modified version used for phone based BV1s.

SD-BV2: All BV2 visits will be conducted by phone.

Annual Visits: The annual visit schedule was modified so that all annual visits can be conducted over the phone or in the clinic.

For peer review only



For peer review only - http://bmjopen.bmj.com/site/about/guidelines.xhtml

# PARTICIPANT CONSENT FORM

MONASH UNIVERSITY

## Appendix 10: Detailed protocol revisions

| No. | Section | Section | Initials/Date |
|---|---|---|---|
| Version 1.2.2 June 2014 | Original approved version (drafts leading up to next version) | | |
| Version 1.3 March 2015 | Cognitive tests added | 5.2<br>7.1.5<br>Study Activity Schedule | SS 27/4/2015 |
| | CES-D-10 added | 5.2<br>7.1.2<br>Study Activity Schedule | |
| | Change from 2 yearly annual visits to yearly annual visits | 7.1.8<br>Study Activity Schedule | |
| | Biobank sub-study – sample collection | 7.1.5<br>Study Activity Schedule | |
| | One month follow up visit added | 7.1.7 | |
| Version 1.3.1 July 2015 | SF-12 changed to SF-36 | 5.2 | SS 17/8/2015 |
| | Biobank Steering Committee added to Governance structure | 12.5.1 | |
| Version 1.3.2 January 2016 | Endpoint definitions expanded | 5.2, 5.3, 11.1, 11.2<br>Appendix 2 | FOH 27/1/2016 |
| | Study medication post out change | | |
| | Study medication compliance questionnaire added | Appendix 4 | |
| | Table of contents added | | |
| | Expansion of SAE and SUSAR reporting | 11.2 | |
| Version 1.3.3 July 2016 | Updated exclusion criteria regarding cytochrome P450 3A4 inhibitors | 6.2<br>8.2.1 (added)<br>Appendix 3 | SS 13/7/2016 |
| | Community participant follow up added | 6.3.1 | |

For peer review only



For peer review only - http://bmjopen.bmj.com/site/about/guidelines.xhtml

# PARTICIPANT CONSENT FORM

MONASH UNIVERSITY

| | | | |
|---|---|---|---|
| | International Steering Committee added | 12.5.1 | FO 14/7/2016 |
| | Change in biobank study samples collected | 7.1.5 | SS 13/9/2016 |
| | Addition of reporting requirements for AEs and SAEs | 11.1.0 Appendix 5 | SS 22/3/2017 |
| Version 2.0 July 2018 | Updates to align protocol with SPIRIT 2013 statement recommendations | Across protocol | AC/SZ/SS 18/7/2018 |
| | Addition of STAREE hubs | Appendix 1 | SS 18/7/2018 |
| | Addition of Data access policy | Section 12.4 | AC 12/3/2019 |
| | Addition of secondary endpoints and clarification of both primary and secondary endpoints | Section 5 | AC 12/3/2019 |
| | Addition of new Forest plot | Section 1.2 | AC 12/3/2019 |
| | Addition of single-lead ECG, IPAQ-E and TICS-M to measurement and study schedule | Table 2 | AC/SS 13/9/2019 |
| | Updated exclusion criteria regarding cytochrome P450 3A4 inhibitors | Appendix 3 | AC/IH/SS 9/9/2019 |
| | Updated dementia definition from DSM IV to DSM V | Appendix 2 | ES/SZ/SS 13/9/2019 |
| Version 3.0 | Removal of Bictegravir from table | Appendix 3 | AK 10/2/20 |
| | Addition of information on data security | Section 12.4 | AC/SS 10/2/20 |
| | Clarification of definition of disability free survival | Section 5.1 p 27 | AC 10/2/20 |
| | Modification of dementia and cognitive decline endpoint brief definitions | Appendix 2 | AC 13/2/20 & July 2022 |

For peer review only


For peer review only - http://bmjopen.bmj.com/site/about/guidelines.xhtml

# PARTICIPANT CONSENT FORM

*MONASH UNIVERSITY*

| | | | |
|---|---|---|---|
| | COVID response – schedule update | Appendix 9/ Table 2 | SS 27/05/2020 |
| | Modification of heart failure endpoint brief definition | Appendix 2 | AC 1/07/2020 |
| | Changes to power calculations, sample size and follow-up period | Section 10 | |
| | Addition of Executive Summary | Section 1 | AC 1/06/2021 |
| | Addition of STAREE MIND protocol | Appendix 10 | AC 1/06/2021 |
| | Extended Revisions table moved to appendices and replaced with summary revision table | Appendix 11 | AC 1/06/2021 |

For peer review only


For peer review only - http://bmjopen.bmj.com/site/about/guidelines.xhtml

# PARTICIPANT CONSENT FORM

*MONASH UNIVERSITY*

# PARTICIPANT INFORMATION SHEET/CONSENT FORM

*MONASH UNIVERSITY*

**Full Project Title:** The STAREE Healthy Ageing Biobank

This participant information and consent form is 9 pages long. Please make sure you have all the pages. For any queries, information on the project or to speak to the research team please call **1800 770 664**.

**INTRODUCTION**

You are being invited to provide a blood and urine sample for the STAREE Healthy Ageing Biobank because you are a participant in the STAREE Clinical Trial. The sample you provide for the STAREE Healthy Ageing Biobank is in addition to any blood and urine that you have taken for the STAREE clinical trial. This is because research using blood and urine samples may help us to find out more about what causes disease, how to prevent it, and how to treat it. The research project aims to create a collection of blood and urine samples which scientists can then use in the future to identify biomarkers or genes associated with conditions such as heart attacks, strokes, cancer and Alzheimer's disease.

This Participant Information and Consent Form tells you about the research project. It explains what is involved to help you decide if you want to take part.

Please read this information carefully. Ask questions about anything that you don't understand or want to know more about. Before deciding whether or not to take part, you might want to talk about it with a relative, friend or your local health worker.

Participation in this research is voluntary. If you don't wish to take part, you don't have to. Note that consent for this additional research is separate to the informed consent you have already provided for participation in the STAREE Clinical Trial. If you do not wish to take part in this additional project you may still participate in the STAREE Clinical Trial. Additionally, consent can be given for different parts of the study, as indicated by the checked boxes at the end of this consent form.

If you decide you want to take part in the research project, you will be asked to sign the consent section. By signing it you are telling us that you:

- understand what you have read;
- consent to take part in the research project;


For peer review only - http://bmjopen.bmj.com/site/about/guidelines.xhtml

# PARTICIPANT CONSENT FORM

MONASH UNIVERSITY

- consent to be involved in the procedures described;
- consent to the use of your personal and health information as described.

You will be given a copy of this Participant Information and Consent Form to keep.

**WHAT IS THE PURPOSE OF THIS RESEARCH PROJECT?**

- *What are biomarkers and genes, and why are they important?*

Biomarkers, including proteins, which are found in the blood and urine, are molecules that are needed for the structure, function and regulation of cells that make up the body's tissues and organs. We are still learning about the proteins that are present in healthy people such as yourself.

In addition to proteins, genetic material (or DNA) can also be collected from blood and urine. The unique way in which our many genes are arranged, is what makes us all different. However there are some sections of DNA which will almost always cause some diseases, or at least increase the risk of someone suffering from a condition or disease. We still have much to learn about these critical sections of DNA. For example, it is estimated that the genetic predisposition for heart disease may involve more than 200 genes. However the exact role is known for only a small number of these genes.

Previous studies have shown that changes in certain proteins and genetic material can be detected in people with cardiovascular disease, forms of cancer, and in Alzheimer's disease. However much more work needs to be carried out before we know which particular proteins and genes play the biggest role in actually causing these diseases.

- What is this study about?

We would like to see whether we can identify known or new proteins and/or genes in the blood and urine that may be linked with the incidence of strokes, heart attacks, bleeding episodes, changes in mental function and development of cancer.

We are seeking to collect blood and urine samples from participants who decide to take part in the STAREE Clinical Trial.

This research has been funded by Monash University.


For peer review only - http://bmjopen.bmj.com/site/about/guidelines.xhtml

# Participant Consent Form

*Monash University*

### What does participation in this research project involve?

**Procedures**: We are asking you to provide a blood and urine sample for the STAREE Biobank.

At a STAREE Biobank visit, either a research nurse, or other trained professional, will collect the blood sample for the STAREE Biobank. You will be provided with a collection cup for the urine sample.

The sample collection for the Biobank involves taking 30–40 ml (just over 2 tablespoons) of your blood and collecting up to 70mls of your urine, and will add about 15 minutes to the length of your second visit or take about 15 minutes at a convenient location near you.

You are **not** required to fast before providing the additional blood and urine sample for the STAREE Biobank. After you have provided the blood and urine sample, it will have your name and any identifying features removed, and replaced with a code number. That code number will be the only information attached to your sample.

Your blood and urine samples will be processed and transported (if necessary) to the STAREE Healthy Ageing Biobank laboratory at the Clinical Trials Centre, Monash University, for storage (and other locations, such as the Alfred hospital, may be used as long-term storage sites in future). Research staff at this laboratory will not be able to link the code number to your identity and therefore will not know to whom the sample belongs.

**Follow-up:** If you agree to provide a blood and urine sample for the Biobank, but do not ultimately continue on in the STAREE Clinical Trial for whatever reason, you can still choose to remain a participant in the Biobank. This will mean that your stored blood and urine samples are kept in the STAREE Biobank, and we will contact you on an annual basis for up to 5 years to ask you a few questions about your health. We will also continue to have access to your medical records (your doctor's records, and those of any hospital or specialist whom you may visit).

We also ask you for permission to obtain information about you from a government database known as the National Death Index. Providing us with this information greatly facilitates us knowing about your health status.

(If you remain in the STAREE Clinical Trial, follow-up will consist of the above, but annual contact will occur via face-to-face visits with the research staff).

You may be asked to provide another 30–40 ml of your blood and up to 70mls of urine at another time during the study, such as at the 3 year time point and/or at the end of the study (in about 5 years).


For peer review only - http://bmjopen.bmj.com/site/about/guidelines.xhtml

# Participant Consent Form

*Monash University*

**Reimbursement:** You will not be paid for your participation in this research.

Although knowledge acquired through medical research may lead to discoveries that are of commercial value, there will be no financial benefit to yourself, your family or the STAREE Biobank.

### What are the possible benefits?

You will not benefit directly from the results of this research, but it is hoped that the results of research on your blood and urine, and those from other participants, may help doctors gain further understanding of how to reduce the risk of death, heart attacks, strokes, heart failure, cancer or dementia in people like yourself. Your blood and urine will be helpful whether you develop a major disease or not.

### What are the possible risks?

**Blood collection:** When blood samples are taken from a vein in your arm you may experience some discomfort. A small number of participants may faint, have some bruising, or a minor infection or blood clot form under the skin. These are generally minor discomforts which should resolve themselves in a few days.

If you should feel light-headed or dizzy during or after the blood sample collection procedure you will be encouraged to remain at the Clinical Trials Centre (or alternative blood collection site) until you feel comfortable leaving.

**Confidentiality:** The code number assigned to your blood and urine sample will be linked to your personal information that is stored on a separate database held at Monash University, Department of Epidemiology & Preventive Medicine. Therefore, the possibility exists that your blood and urine sample could be re-identified, however this is most unlikely to occur – see the section **What will happen to information about me?**

**Genetic testing:** Genetic testing of samples may occur in the future if additional funding is available. Any future projects must be approved by a Human Research Ethics Committee. If any future research uncovers any significant information specific to your health, our STAREE Steering Committee may decide to authorise someone to contact you and offer you access to this information. You may decline the information. "Significant" information is any findings that are deemed very serious or highly significant, that have been validated and to which there is known medical support available. If you wish to be given this information a qualified person will explain it to you. You should also consider whether this information should be made known to your family members. Sharing these findings could help avoid similar medical problems in your family. Before deciding whether or not to receive these results, you may want to discuss this with a relative or a significant other first. By signing this consent, you will


For peer review only - http://bmjopen.bmj.com/site/about/guidelines.xhtml

# PARTICIPANT CONSENT FORM

MONASH UNIVERSITY

be asked to select if you wish to be informed or not informed of any serious or highly significant genetic results in the future that has any specific health implications for you or your genetic relatives – see page 9.

### DO I HAVE TO TAKE PART IN THIS RESEARCH PROJECT?

Participation in any research project is voluntary. If you do not wish to take part, you do not have to. If you decide to take part and later change your mind, you are free to withdraw from the project at a later stage.

If you decide to withdraw, please notify a member of the STAREE research team in writing. This will allow that person or the research supervisor to inform you if there are any special requirements linked to withdrawing.

If you decide to leave the project, the researchers would like to keep the blood and urine samples and the personal and health information about you that has already been collected. This is to help them make sure that the results of the research can be measured properly. However, you may elect to have samples destroyed and health and personal information removed from the study. If you choose to have your samples and information removed you must tell the researchers before you withdraw from the research project.

Your decision whether to take part or not, or to take part and then withdraw, will not affect your relationship with the researchers or your doctor.

### HOW WILL I BE INFORMED OF THE FINAL RESULTS OF THIS RESEARCH PROJECT?

The establishment of the STAREE Healthy Ageing Biobank is purely for research purposes only. At this formative stage, the Biobank cannot be used for the diagnosis of diseases because we do not yet know which particular biomarkers or genes will emerge, in the years to come, as being the most significant in terms of predicting disease onset. The ongoing nature of the development of the Biobank means that the final results of this project will not be available for some considerable time. If however in the future, genetic testing is undertaken and you consent to take part in this study, you will be asked at the end of this information sheet to consider being notified or not notified of any significant result (see also page 9).

Eventually, results of group data from the research that is conducted will be published in medical journals, and presented at national and international conferences.

### WHAT WILL HAPPEN TO INFORMATION ABOUT ME?

The STAREE Biobank laboratory is equipped to store and handle blood and urine samples appropriately for future tests. Your potentially re-identifiable blood and urine samples will be


For peer review only - http://bmjopen.bmj.com/site/about/guidelines.xhtml

# PARTICIPANT CONSENT FORM

*MONASH UNIVERSITY*

stored for a long time, possibly for many years after the completion of the study. When all the samples are no longer required they will be destroyed by the STAREE Biobank laboratory, or the laboratory in which the research has been conducted, in a safe manner according to local laboratory procedures.

**Who will make use of my blood and urine samples?**

The STAREE Biobank is a non-profit service dedicated to providing a resource to scientists involved in medical research. Researchers from universities, hospitals, and other health organizations conduct research using blood and urine. They may contact the STAREE Biobank and request samples for their studies. The researcher must have funding to carry out the study, and must have the study reviewed scientifically, before they are permitted to access the blood and urine samples from the STAREE Biobank. All studies must have approval by a Human Research Ethics Committee. Your samples will NOT be sold. The STAREE Biobank may charge researchers a fee to recover some of the costs of storing and administering its collection of samples.

Your blood and urine sample will not be linked to your name, initials, date of birth or any other information that may identify you.

**What types of tests will be performed?**

The tests performed on your blood and urine may include tests for inflammation, nutrition, insulin, hormones and genes that are thought to be related to heart disease, diabetes, dementia, cancer or other major diseases. Some researchers may develop new tests to identify diseases. Others may develop new ways to treat or even cure diseases. In the future, some of the research may help develop new products, such as tests and drugs.

Some research looks at diseases that are passed on in families (called familial or hereditary genetic research). Research done with your blood and urine may look for genetic causes and signs of disease.

Rapid advances in technology make it impossible to predict what new tests or studies may be possible in the future. The selection of tests will be guided by current and future scientific information about heart disease, diabetes, dementia, cancer or other major diseases.

**You may consent to having your blood and urine sample tested for biomarkers, genetic markers, or both; see Consent Form, Page 9.**

Any information obtained in connection with this research project that can identify you will remain confidential and will only be used for the purpose of this research project. It will only be disclosed with your permission, except as required by law.


For peer review only - http://bmjopen.bmj.com/site/about/guidelines.xhtml

# Participant Consent Form

*Monash University*

In any publication and/or presentation, information will be provided in such a way that you cannot be identified. The STAREE Biobank and the STAREE Co-ordinating Centre (Monash University) are responsible for ensuring the information about you is kept private. They will take every precaution to safeguard your identity, and prevent misuse of records. Only authorised people who have agreed in writing to protect your identity will have access to your linked information. Therefore, all other researchers working with your samples and clinical information will not know your identity. This will make it very difficult for any research results (and importantly, those relating to genetic information) to be linked to you or your family. Also, people outside the research process will not have access to results about any one person, which will help to protect your privacy.

**Can I access research information kept about me?**

A report of the individual results of any tests done on your blood and urine samples will not routinely be made available to you, your family members or any other person. This is because research can take a long time and must use blood and/or urine samples from many people before results are known. Results from research using your samples may not be ready for many years and will not affect your care right now, but they may be helpful to people like you in the future.

In accordance with relevant Australian and/or Victorian privacy and other relevant laws, you have the right to access the information collected and stored by the researchers about you. Please contact one of the researchers named at the end of this document if you would like to access your information.

Further, in accordance with regulatory guidelines, the information collected in this research project will be kept indefinitely. You must be aware that the information may become de-identified at some point and access to information about you after this point will not be possible.

**Is this research approved?**

The ethical aspects of this research project have been approved by the Alfred Hospital Ethics Committee.

This project will be carried out according to the *National Statement on Ethical Conduct in Human Research (2007)* produced by the National Health and Medical Research Council of Australia. This statement has been developed to protect the interests of people who agree to participate in human research.


For peer review only - http://bmjopen.bmj.com/site/about/guidelines.xhtml

# PARTICIPANT CONSENT FORM

*MONASH UNIVERSITY*

## FURTHER INFORMATION AND WHO TO CONTACT

Please ask the research staff member if you have any further questions.

For specific enquiries related to this study, or matters relating to research at the site at which you are participating, or any questions about being a research participant in general, you may contact:

Name: Associate Professor Sophia Zoungas (Monash University):
Phone: +61 3 9903 0711
E-mail: sophia.zoungas@monash.edu

If you have any complaints about any aspect of the project or the way it is being conducted, you may contact:
Complaints Officer
Office of Ethics & Research Governance
Alfred Health

Tel: (03) 9076 3619

You will need to quote the following Alfred Health project number: 266/15.

For peer review only


For peer review only - http://bmjopen.bmj.com/site/about/guidelines.xhtml

# Participant Consent Form

*Monash University*

**Title** The STAREE Healthy Ageing Biobank

## Declaration by Participant

I have read, or have had read to me, the *"The STAREE Healthy Ageing Biobank" Patient Information, Version 1.6.1, Dated 17 April 2018* in a language that I understand, and I understand the purposes, procedures and risks of this research project as described within it.

I have had an opportunity to ask questions and I am satisfied with the answers I have received.

I freely agree to participate in this research project, as described.

I understand that I will be given a signed copy of this document to keep.

The researcher has agreed not to reveal my identity and personal details if information about this project is published or presented in any public form.

I agree to my blood and urine samples being stored and tested for **biomarkers** in the future

☐ YES ☐ NO

I agree to my blood and urine samples being stored and tested for **genetic markers** in the future

☐ YES ☐ NO

I agree to be **informed** of any significant genetic results that have specific health implications for me and/or my genetic relatives

☐ YES ☐ NO

Name of participant (please print)______________________________

Signature______________________________Date_______________

## Declaration by Researcher

I have given a verbal explanation of the research project, its procedures and risks and I believe that the participant has understood that explanation.

Name of researcher (please print)______________________________

Signature______________________________Date_______________


For peer review only - http://bmjopen.bmj.com/site/about/guidelines.xhtml

# Reporting checklist for protocol of a clinical trial.

Based on the SPIRIT guidelines.

## Instructions to authors

Complete this checklist by entering the page numbers from your manuscript where readers will find each of the items listed below.

Your article may not currently address all the items on the checklist. Please modify your text to include the missing information. If you are certain that an item does not apply, please write "n/a" and provide a short explanation.

Upload your completed checklist as an extra file when you submit to a journal.

In your methods section, say that you used the SPIRITreporting guidelines, and cite them as:

Chan A-W, Tetzlaff JM, Gøtzsche PC, Altman DG, Mann H, Berlin J, Dickersin K, Hróbjartsson A, Schulz KF, Parulekar WR, Krleža-Jerić K, Laupacis A, Moher D. SPIRIT 2013 Explanation and Elaboration: Guidance for protocols of clinical trials. BMJ. 2013;346:e7586

| | | Reporting Item | Page Number |
|---|---|---|---|
| **Administrative information** | | | |
| Title | #1 | Descriptive title identifying the study design, population, interventions, and, if applicable, trial acronym | 1 |
| Trial registration | #2a | Trial identifier and registry name. If not yet | 4 |

For peer review only

| | | | |
|---|---|---|---|
| | | registered, name of intended registry | |
| Trial registration: data set | #2b | All items from the World Health Organization Trial Registration Data Set | Appendix 2 |
| Protocol version | #3 | Date and version identifier | Appendix 3: STAREE protocol V3.0 |
| Funding | #4 | Sources and types of financial, material, and other support | 25 |
| Roles and responsibilities: contributorship | #5a | Names, affiliations, and roles of protocol contributors | 1 & 25 |
| Roles and responsibilities: sponsor contact information | #5b | Name and contact information for the trial sponsor | 25 |
| Roles and responsibilities: sponsor and funder | #5c | Role of study sponsor and funders, if any, in study design; collection, management, analysis, and interpretation of data; writing of the report; and the decision to submit the report for publication, including whether they will have ultimate authority over any of these activities | 25 |
| Roles and responsibilities: | #5d | Composition, roles, and responsibilities of the coordinating centre, steering committee, endpoint | 15, 20, 25, Appendix 1 |

For peer review only

| | | | |
|---|---|---|---|
| committees | | adjudication committee, data management team, and other individuals or groups overseeing the trial, if applicable (see Item 21a for data monitoring committee) | |
| **Introduction** | | | |
| Background and rationale | #6a | Description of research question and justification for undertaking the trial, including summary of relevant studies (published and unpublished) examining benefits and harms for each intervention | 5 to 9 |
| Background and rationale: choice of comparators | #6b | Explanation for choice of comparators | 5 to 9 |
| Objectives | #7 | Specific objectives or hypotheses | 9 |
| Trial design | #8 | Description of trial design including type of trial (eg, parallel group, crossover, factorial, single group), allocation ratio, and framework (eg, superiority, equivalence, non-inferiority, exploratory) | 10 |
| **Methods: Participants, interventions, and outcomes** | | | |
| Study setting | #9 | Description of study settings (eg, community clinic, academic hospital) and list of countries where data | 10 |

For peer review only

| | | | |
|---|---|---|---|
| | | will be collected. Reference to where list of study sites can be obtained | |
| Eligibility criteria | #10 | Inclusion and exclusion criteria for participants. If applicable, eligibility criteria for study centres and individuals who will perform the interventions (eg, surgeons, psychotherapists) | 11 |
| Interventions: description | #11a | Interventions for each group with sufficient detail to allow replication, including how and when they will be administered | 12 |
| Interventions: modifications | #11b | Criteria for discontinuing or modifying allocated interventions for a given trial participant (eg, drug dose change in response to harms, participant request, or improving / worsening disease) | 12, 13 |
| Interventions: adherance | #11c | Strategies to improve adherence to intervention protocols, and any procedures for monitoring adherence (eg, drug tablet return; laboratory tests) | 12 & 13 |
| Interventions: concomitant care | #11d | Relevant concomitant care and interventions that are permitted or prohibited during the trial | 13 |
| Outcomes | #12 | Primary, secondary, and other outcomes, including the specific measurement variable (eg, systolic blood pressure), analysis metric (eg, change from baseline, final value, time to event), method of aggregation (eg, median, proportion), and time point for each outcome. Explanation of the clinical | 9, 14-16 |

For peer review only

| | | | |
|---|---|---|---|
| | | relevance of chosen efficacy and harm outcomes is strongly recommended | |
| Participant timeline | #13 | Time schedule of enrolment, interventions (including any run-ins and washouts), assessments, and visits for participants. A schematic diagram is highly recommended (see Figure) | Appendix 3 |
| Sample size | #14 | Estimated number of participants needed to achieve study objectives and how it was determined, including clinical and statistical assumptions supporting any sample size calculations | 16-18, Appendix 4 |
| Recruitment | #15 | Strategies for achieving adequate participant enrolment to reach target sample size | 9-10 |
| **Methods: Assignment of interventions (for controlled trials)** | | | |
| Allocation: sequence generation | #16a | Method of generating the allocation sequence (eg, computer-generated random numbers), and list of any factors for stratification. To reduce predictability of a random sequence, details of any planned restriction (eg, blocking) should be provided in a separate document that is unavailable to those who | 11 |

For peer review only

| | | | |
|---|---|---|---|
| | | enrol participants or assign interventions | |
| Allocation concealment mechanism | #16b | Mechanism of implementing the allocation sequence (eg, central telephone; sequentially numbered, opaque, sealed envelopes), describing any steps to conceal the sequence until interventions are assigned | 11 |
| Allocation: implementation | #16c | Who will generate the allocation sequence, who will enrol participants, and who will assign participants to interventions | 11 |
| Blinding (masking) | #17a | Who will be blinded after assignment to interventions (eg, trial participants, care providers, outcome assessors, data analysts), and how | 14 |
| Blinding (masking): emergency unblinding | #17b | If blinded, circumstances under which unblinding is permissible, and procedure for revealing a participant's allocated intervention during the trial | 14 |
| **Methods: Data collection, management, and analysis** | | | |
| Data collection plan | #18a | Plans for assessment and collection of outcome, baseline, and other trial data, including any related processes to promote data quality (eg, duplicate measurements, training of assessors) and a description of study instruments (eg, | 12, Appendix 3 STAREE protocol V 3.0 |

For peer review only

| | | | |
|---|---|---|---|
| | | questionnaires, laboratory tests) along with their reliability and validity, if known. Reference to where data collection forms can be found, if not in the protocol | |
| Data collection plan: retention | #18b | Plans to promote participant retention and complete follow-up, including list of any outcome data to be collected for participants who discontinue or deviate from intervention protocols | Appendix 3: STAREE protocol V 3.0 |
| Data management | #19 | Plans for data entry, coding, security, and storage, including any related processes to promote data quality (eg, double data entry; range checks for data values). Reference to where details of data management procedures can be found, if not in the protocol | Appendix 3: STAREE Protocol V3.0 |
| Statistics: outcomes | #20a | Statistical methods for analysing primary and secondary outcomes. Reference to where other details of the statistical analysis plan can be found, if not in the protocol | 18-20 |
| Statistics: additional analyses | #20b | Methods for any additional analyses (eg, subgroup and adjusted analyses) | 20 |
| Statistics: analysis population and missing data | #20c | Definition of analysis population relating to protocol non-adherence (eg, as randomised analysis), and any statistical methods to handle missing data (eg, multiple imputation) | 19 |

For peer review only

**Methods: Monitoring**

| | | | |
|---|---|---|---|
| Data monitoring: formal committee | #21a | Composition of data monitoring committee (DMC); summary of its role and reporting structure; statement of whether it is independent from the sponsor and competing interests; and reference to where further details about its charter can be found, if not in the protocol. Alternatively, an explanation of why a DMC is not needed | 20-21 |
| Data monitoring: interim analysis | #21b | Description of any interim analyses and stopping guidelines, including who will have access to these interim results and make the final decision to terminate the trial | 21 |
| Harms | #22 | Plans for collecting, assessing, reporting, and managing solicited and spontaneously reported adverse events and other unintended effects of trial interventions or trial conduct | 13 |
| Auditing | #23 | Frequency and procedures for auditing trial conduct, if any, and whether the process will be independent from investigators and the sponsor | Appendix 3: Study Protocol V3.0 |

**Ethics and dissemination**

| | | | |
|---|---|---|---|
| Research ethics approval | #24 | Plans for seeking research ethics committee / institutional review board (REC / IRB) approval | 21 |

For peer review only

| | | | |
|---|---|---|---|
| Protocol amendments | #25 | Plans for communicating important protocol modifications (eg, changes to eligibility criteria, outcomes, analyses) to relevant parties (eg, investigators, REC / IRBs, trial participants, trial registries, journals, regulators) | Appendix 3 STAREE protocol v3.0 |
| Consent or assent | #26a | Who will obtain informed consent or assent from potential trial participants or authorised surrogates, and how (see Item 32) | 10 |
| Consent or assent: ancillary studies | #26b | Additional consent provisions for collection and use of participant data and biological specimens in ancillary studies, if applicable | Appendix 4 |
| Confidentiality | #27 | How personal information about potential and enrolled participants will be collected, shared, and maintained in order to protect confidentiality before, during, and after the trial | Appendix 3 STAREE Protocol |
| Declaration of interests | #28 | Financial and other competing interests for principal investigators for the overall trial and each study site | 26 |
| Data access | #29 | Statement of who will have access to the final trial dataset, and disclosure of contractual agreements that limit such access for investigators | 22 |
| Ancillary and post trial care | #30 | Provisions, if any, for ancillary and post-trial care, and for compensation to those who suffer harm from trial participation | 13 |

For peer review only

| | | | |
|---|---|---|---|
| Dissemination policy: trial results | #31a | Plans for investigators and sponsor to communicate trial results to participants, healthcare professionals, the public, and other relevant groups (eg, via publication, reporting in results databases, or other data sharing arrangements), including any publication restrictions | 21 |
| Dissemination policy: authorship | #31b | Authorship eligibility guidelines and any intended use of professional writers | 22 |
| Dissemination policy: reproducible research | #31c | Plans, if any, for granting public access to the full protocol, participant-level dataset, and statistical code | 22 |
| **Appendices** | | | |
| Informed consent materials | #32 | Model consent form and other related documentation given to participants and authorised surrogates | Appendix 3 |
| Biological specimens | #33 | Plans for collection, laboratory evaluation, and storage of biological specimens for genetic or molecular analysis in the current trial and for future use in ancillary studies, if applicable | 16 (manuscript); Appendix 3: STAREE protocol V3.0 |

None The SPIRIT Explanation and Elaboration paper is distributed under the terms of the Creative Commons Attribution License CC-BY-NC. This checklist can be completed online using https://www.goodreports.org/, a tool made by the EQUATOR Network in collaboration with Penelope.ai

For peer review only

# Statins for extension of disability free survival and primary prevention of cardiovascular events among older people: protocol for a randomised controlled trial in primary care (STAREE trial)



| | |
|---|---|
| Journal: | *BMJ Open* |
| Manuscript ID | bmjopen-2022-069915.R1 |
| Article Type: | Protocol |
| Date Submitted by the Author: | 01-Mar-2023 |





| | |
|---|---|
| Complete List of Authors: | Zoungas, Sophia; Monash University, School of Public Health and Preventive Medicine<br>Curtis, Andrea; Monash University, School of Public Health & Preventive Medicine<br>Spark, Simone; Monash University, School of Public Health and Preventive Medicine<br>Wolfe, Rory; Monash University, School of Public Health and Preventive Medicine<br>McNeil, John; Monash University, School of Public Health and Preventive Medicine<br>Beilin, Lawrence; University of Western Australia, School of Medicine<br>Chong, Trevor T-J; Monash University, Turner Institute for Brain and Mental Health; Alfred Health, Department of Neurology<br>Cloud, Geoffrey; Monash University, Department of Neuroscience; Alfred Health, Department of Neurology<br>Hopper, Ingrid; Monash University, School of Public Health and Preventive Medicine; Alfred Health, Department of Cardiology and General Medicine Unit<br>Kost, Alissia; Monash University, School of Public Health and Preventive Medicine<br>Nelson, Mark; University of Tasmania, Menzies Research Institute<br>Nicholls, Stephen; Monash University, School of Public Health and Preventive Medicine; Monash University, Victorian Heart Institute<br>Reid, Christopher; Monash University, School of Public Health and Preventive Medicine; Curtin University, School of Public Health<br>Ryan, Joanne; Monash University, School of Public Health and Preventive Medicine<br>Tonkin, Andrew; Monash University, School of Public Health and Preventive Medicine<br>Ward, Stephanie; Monash University, School of Public Health and Preventive Medicine; University of New South Wales, Centre for Healthy Brain Ageing, School of Psychiatry<br>Wierzbicki, Anthony; Guy's and St Thomas' Hospitals, Metabolic Medicine/Chemical Pathology |



| | |
|---|---|
| <b>Primary Subject Heading</b>: | Cardiovascular medicine |

| | |
|---|---|
| Secondary Subject Heading: | Cardiovascular medicine |
| Keywords: | Ischaemic heart disease < CARDIOLOGY, PREVENTIVE MEDICINE, Dementia |
| | |

SCHOLARONE™
Manuscripts

**Statins for extension of disability free survival and primary prevention of cardiovascular events among older people: protocol for a randomised controlled trial in primary care (STAREE trial)**

**Authors:** Sophia Zoungas[1], Andrea J Curtis[1], Simone Spark[1], Rory Wolfe[1], John J McNeil[1], Lawrence Beilin[2], Trevor T-J Chong[3,4,5], Geoffrey C Cloud[4,6], Ingrid Hopper[1,7], Alissia Kost[1], Mark R Nelson[8], Stephen J Nicholls[1,9], Christopher M Reid[10], Joanne Ryan[1], Andrew Tonkin[1], Stephanie A Ward[1,11,12], Anthony S Wierzbicki[13] and the STAREE investigator group

Affiliations:

[1] School of Public Health and Preventive Medicine, Monash University, Melbourne, Australia.

[2] School of Medicine, Royal Perth Hospital, University of Western Australia, Perth, Western Australia.

[3] Turner Institute for Brain and Mental Health, Monash University, Victoria, Australia.

[4] Department of Neurology, Alfred Health, Melbourne, Victoria, Australia.

[5] Department of Clinical Neurosciences, St Vincent's Hospital, Melbourne, Victoria, Australia.

[6] Department of Neuroscience, Central Clinical School, Monash University, Melbourne, Australia.

[7] Department of Cardiology and General Medicine Unit, Alfred Health, Melbourne, Australia.

[8] Menzies Institute for Medical Research, University of Tasmania, Hobart, TAS, Australia.


For peer review only - http://bmjopen.bmj.com/site/about/guidelines.xhtml

[9] Victorian Heart Institute, Monash University, Clayton, Australia.

[10] School of Population Health, Curtin University, Perth, WA, Australia.

[11] Centre for Healthy Brain Ageing, School of Psychiatry, UNSW, Sydney, NSW.

[12] Department of Geriatric Medicine, The Prince of Wales Hospital, Randwick, NSW

[13] Department of Metabolic Medicine/Chemical Pathology, Guy's & St Thomas' Hospitals, London.

Corresponding author:

Professor Sophia Zoungas MBBS(Hons) PhD FRACP

School of Public Health and Preventive Medicine

Monash University

Melbourne, Australia

Email: sophia.zoungas@monash.edu

Phone: +61 3 9903 0711

Word Count: 4967

Keywords: statin, primary prevention, ageing, dementia, cardiovascular disease

For peer review only



For peer review only - http://bmjopen.bmj.com/site/about/guidelines.xhtml

## ABSTRACT

**Introduction:** The world is undergoing a demographic transition to an older population. Preventive healthcare has reduced the burden of chronic illness at younger ages but there is limited evidence that these advances can improve health at older ages. Statins are one class of drug with the potential to prevent or delay the onset of several causes of incapacity in older age, particularly major cardiovascular disease (CVD). This paper presents the protocol for the STAtins in Reducing Events in the Elderly (STAREE) trial, a randomised double-blind placebo-controlled trial (RCT) examining the effects of statins in community dwelling older people without CVD, diabetes or dementia.

**Methods and Analysis:** We will conduct a double blind, randomised placebo-controlled trial among people aged 70 years and over, recruited through Australian general practice and no history of clinical CVD, diabetes or dementia. Participants will be randomly assigned to oral atorvastatin (40 mg daily) or matching placebo (1:1 ratio). The co-primary endpoints are disability-free survival defined as survival free of dementia and persistent physical disability and major cardiovascular events (cardiovascular death or non-fatal myocardial infarction or stroke). Secondary endpoints are all-cause death, dementia and other cognitive decline, persistent physical disability, fatal and non-fatal myocardial infarction, fatal and non-fatal stroke, heart failure, atrial fibrillation, fatal and non-fatal cancer, all-cause hospitalisation, need for permanent residential care and quality of life. Comparisons between assigned treatment arms will be on an intention-to-treat basis with each of the co-primary endpoints analysed separately in time-to-first-event analyses using Cox proportional hazards regression models.

**Ethics and Dissemination:** STAREE will address uncertainties about the preventive effects of statins on a range of clinical outcomes important to older people. Institutional ethics approval has been obtained. All research outputs will be disseminated to GP co-investigators



For peer review only - http://bmjopen.bmj.com/site/about/guidelines.xhtml

and participants, published in peer-reviewed journals and presented at national and international conferences.

**Trial registration number**

ClinicalTrials.gov Identifier: NCT02099123. Date of registration: March 28, 2014.

## ARTICLE SUMMARY

### Strengths and limitations

- STAtins in Reducing Events in the Elderly (STAREE) is a double blind, placebo-controlled randomised clinical trial among a primary prevention population of people aged 70 years and over that will evaluate the effect of statin therapy on two co-primary outcomes including disability-free survival and major cardiovascular events.
- STAREE is a pragmatic trial run through general practice across metropolitan and regional/rural Australia with usual care provided by general practitioners.
- STAREE is one of the largest statin trials to carry out a comprehensive assessment of several causes of incapacity including dementia and disability.
- Complete vital status ascertainment will be facilitated by linkage to the National Death Index.
- STAREE will be examining the effects of starting statins but not withdrawing statins or de-escalating therapy in older age.

For peer review only


For peer review only - http://bmjopen.bmj.com/site/about/guidelines.xhtml

## INTRODUCTION

The world is experiencing a rapid demographic transition towards an older population.[1] Increasing survival amongst older age groups is a major contributor to this shift, with almost twice as many older people surviving into their eighties and nineties compared to fifty years ago. [2] In the US, the proportion of the population aged 65 and over is projected to increase from 40 million in 2009 to 89 million in 2050 with 19 million (20%) in the oldest age group (85+) by 2050. [3] Similar trends are seen in other high income countries such as Australia, Canada, Japan, Europe and the United Kingdom. [2] However, over the same time period healthy life expectancy has not increased to the same extent as life expectancy. [4, 5] While preventive therapies have reduced the burden of chronic diseases at younger ages, age remains an important determinant of cardiovascular disease (CVD), dementia and frailty. [6, 7, 8] The net impact of an ageing population is that more people are living with chronic diseases for longer, and these can lead to disability and loss of independence.[9, 10] Loss of independence is not only one of the biggest concerns for older people [11] but has significant societal and economic impacts. [12-14] Identifying preventive interventions that can extend life free of dementia and disability (disability-free survival) is an important public health goal. One promising but insufficiently tested preventive approach to extending disability-free survival among older people is statin therapy.

### The debate about optimal LDL cholesterol levels, use of statin therapy and clinical outcomes in older people.

While high levels of LDL cholesterol (LDL-C) are known to increase the risk of CVD events and mortality among young and middle-aged populations [15, 16], the relationship between LDL-C and clinical outcomes including all-cause or cause-specific death in older populations


For peer review only - http://bmjopen.bmj.com/site/about/guidelines.xhtml

is not consistent. Previous observational studies have reported a mix of no association, positive association or U-curve associations between LDL-C levels and all-cause death [17-20], cardiovascular events [21, 22] and dementia. [16, 23] One explanation for lack of an association or negative association is "reverse causality," whereby unquantified comorbid disease may be causing both lower LDL-C levels and an increased risk of death or dementia. [24] Given the uncertainty around the relationship of LDL-C levels and outcomes in older people, the decision as to whether lipid lowering therapy such as statins should be initiated must be guided by randomised controlled trials. Furthermore, absolute CVD risk calculators, whose use is recommended to assist prescribing decisions for primary prevention, are principally driven by age such that universal treatment becomes the default option without certainty of the risks and benefit for older populations. [25]

Statins have the potential to delay the onset of several causes of incapacity in older people. Their best-recognised pharmacological action is to inhibit the mevalonate pathway and biosynthesis of cholesterol and isoprenoids by binding to HMG-CoA reductase. The reduction in circulating lipid levels, especially LDL-C, underpins their ability to reduce the incidence of acute myocardial infarction and stroke (including subclinical CVD) and their potential long-term sequelae (heart failure, cognitive decline). [26] Statins also exert a number of pleiotropic effects including anti-oxidative, anti-thrombotic and a moderately powerful anti-inflammatory action, [27] which could also help explain their reported beneficial effects in reducing disability and the risk of dementia. [28] The pleiotropic effects of statins have been attributed to the inhibition of isoprenoid metabolite formation (geranylgeranyl pyrophosphate and farnesyl pyrophosphate). [29] These metabolites are intermediates in the post-translational formation of several cell-signalling proteins that control multiple cell functions including maintenance of cell shape, motility, factor secretion,


For peer review only - http://bmjopen.bmj.com/site/about/guidelines.xhtml

differentiation, and proliferation thus explaining improvements in endothelial function, vascular inflammation and oxidation, and atherosclerotic plaque stability. [29] Statins also attenuate neuroinflammation, an enduring feature of neurodegenerative diseases through potent suppression of microglia (neuroimmune cells), activation and inhibition of cytokine production, and have been shown to mitigate the aggregation of amyloid β, a key factor in the development of Alzheimer's disease. [30 31]

In people with known CVD, the benefits of statin therapy are clear, with the risks of cardiovascular events reduced across all ages. In people without CVD, the benefits of taking a statin are not as clear at older ages and continue to be debated. [32, 33] Most national and international guidelines have not been able to make strong recommendations to guide prescribing for older people, and any recommendations have generally been informed by subgroups within trials that have not studied the range of health outcomes meaningful to older people and that consider both beneficial and adverse effects. [34, 35]

A meta-analysis of pooled individual participant data (aged 55 years +) from 28 statin trials that included participants with and without known CVD, reported a 21% reduction in risk of major vascular events per 1 mmol/L reduction in LDL-C, attributed to reduced risks of major coronary events, major stroke events and vascular death. [36] Analyses by age group indicated the greatest risk reductions (25% per 1 mmol/L reduction in LDL-C) among participants aged 55 years or less. This compared to risk reductions of 19% and 13% for older participants aged >70 to ≤75 and >75 years respectively. [36] The risk of all-cause death was also reduced, albeit to a much lesser extent (9% per 1 mmol/L reduction in LDL-C). There was no effect on non-vascular deaths including cancer deaths. Of note, participants were predominantly high risk, middle-aged men with high baseline LDL-C levels (mean weighted


For peer review only - http://bmjopen.bmj.com/site/about/guidelines.xhtml
For peer review only

LDL-C level 3.7 mmol/L) or known CVD. [36] In analyses limited to participants with no history of vascular disease, there was a trend toward smaller proportional risk reductions in major vascular events as age increased. [36] However, a *post hoc* pooling of subgroup data from 2 trials of rosuvastatin (the Justification for the Use of Statins in Prevention: an Intervention Trial Evaluating Rosuvastatin (JUPITER) trial, and the Heart Outcomes Prevention Evaluation (HOPE 3) trial, concluded that the effect on major vascular events in people aged ≥70 years (HR 0.74; 95% CI 0.61 to 0.91) was similar to those < 65 years (HR 0.75; 95% CI 0.57 to 0.97). [37] Importantly, the effects on dementia and physical disability were not comprehensively investigated by these studies. [38]

Yet statin treatment is not without potential risk of harm. [39] Observational studies have reported muscle weakness/pain, liver impairment, new-onset diabetes and haemorrhagic stroke [15, 40, 41] but the incidence of these adverse effects has been much lower in clinical trials. [42] Importantly, statins may interact with other medications, contributing to adverse drug reactions and avoidable hospitalisations. [43] Older adults exposed to polypharmacy are more vulnerable to adverse reactions and increased risks of adverse effects. As with efficacy, the burden of statin adverse effects in older people has not been well studied. As a result, it is still not possible to know the balance of risks and benefits in this age group. [44]

In conclusion, the gains made in increasing lifespan may not have been accompanied by similar gains in healthy life, for the additional later years of life lived. A strong potential exists for greater use of statins for primary prevention to extend healthy life and maintain independence amongst the ageing population however, current evidence for starting statins among older people is insufficient to support this approach. The STAREE trial is a large scale RCT specifically designed to address this need.


For peer review only - http://bmjopen.bmj.com/site/about/guidelines.xhtml

## Study Objectives and Hypotheses

Primary objectives

To determine in people aged ≥70 years the effect of statin therapy (40 mg atorvastatin) versus placebo, on two co-primary clinical endpoints:

(i) disability-free survival defined as survival free of dementia and persistent physical disability (a composite of all cause death or development of dementia or persistent physical disability); and

(ii) major cardiovascular events (a composite of cardiovascular death, non-fatal myocardial infarction or non-fatal stroke).

We hypothesise that treatment with a statin (atorvastatin 40 mg) will:

a) prolong overall disability free survival, and

b) reduce the incidence of major cardiovascular events amongst a primary prevention population aged ≥70 years.

Secondary objectives

To determine the effects of statin therapy (atorvastatin 40mg) versus placebo on each of all-cause death, dementia and other cognitive decline, persistent physical disability, fatal and non-fatal myocardial infarction, fatal and non-fatal stroke and cardiovascular death, fatal and non-fatal cancer, heart failure hospitalisation, atrial fibrillation, all-cause hospitalisation, need for permanent residential care, and quality of life.

Tertiary objectives


For peer review only - http://bmjopen.bmj.com/site/about/guidelines.xhtml

To determine the effects of statin therapy (atorvastatin 40mg) versus placebo on each of fasting glucose/HbA1c, urine ACR, estimated glomerular rate (eGFR), frailty phenotype and functional independence (instrumental ADLs).

## METHODS AND ANALYSIS

### Study design

STAREE is a double blind, randomised, placebo-controlled primary prevention trial. The WHO Trial Registration Data Set is shown in Appendix 1 and Appendix 2a and 2b detail the study protocol.

### Setting, participant recruitment, screening, consent and follow up

Recruitment is being conducted through general practices across metropolitan and regional/rural Australia with participating general practitioners (GPs) registered as STAREE co-investigators. General practice clinical database reviews create a list of potential participants aged ≥70 years without a history of clinical CVD, diabetes, dementia or other life-limiting conditions. Each GP screens the list for suitability and approves the sending of an invitation letter through the mail. Potential participants call the study centre and undergo an initial phone screening for trial inclusion and exclusion criteria. Those who are eligible are invited to take part in two baseline screening visits, either as a face-to-face visit at their usual GP clinic or as a remote phone visit with a trained study team member.

At the first baseline screening visit informed consent is obtained (Appendix 2a) and then further eligibility assessments (described below) are completed. Demographic information, lifestyle factors, past medical history, concomitant medications, and other physical measurements (blood pressure, heart rate, height, weight, and waist circumference) are


For peer review only - http://bmjopen.bmj.com/site/about/guidelines.xhtml

collected. Eligible participants then enter a 4-week run-in period and asked to take 1 tablet of run-in medication (matching placebo) each day to ascertain medication compliance. Placebo has been chosen for run in instead of active drug, so participants are not excluded on the basis of statin intolerance and the results are generalisable to the real-world use of statins. Eligible participants also attend their GP clinic for GP review of the trial inclusion and exclusion criteria, as well as any pathology testing.

At the second baseline screening visit after the run-in period, participants who achieve run-in medication compliance of >85% and have GP approval to participate in the trial, complete a cognitive battery to ascertain baseline cognitive performance (see Appendix 2a).

See Appendix 2a and b for a detailed description of the schedule of visits and all tests performed.

**Inclusion and exclusion criteria**

Individuals are deemed eligible if they; i) are aged ≥70 years; ii) live independently in the community; iii) are willing and able to provide informed consent and accept the study requirements and iv) do not have a history of clinical CVD events, diabetes or dementia. Exclusion criteria are a Modified Mini-Mental State Examination (3MS) score <78 on screening,  moderate or severe chronic kidney disease, moderate or severe liver disease, or serious inter-current illness likely to cause death within the next 5 years; total cholesterol >7.5 mmol/L; current participation in another interventional clinical trial; and an absolute contraindication to statin therapy, current use of statin therapy or other lipid lowering therapy for primary prevention and unwilling to stop therapy or current long term or permanent use of potent cytochrome P450 (CYP) 3A4 inhibitors.


For peer review only - http://bmjopen.bmj.com/site/about/guidelines.xhtml

## Randomisation to study treatment

Participants are randomly allocated to either atorvastatin or placebo treatment through a web-based system according to a randomisation schedule generated by an independent statistician. The allocation list was generated using randomly permuted blocks (of size either 2 or 4) with randomisation stratified by state (Victoria, Tasmania, South Australia, Western Australia, Queensland and New South Wales) and age group (70-80 years, ≥80 years). Blocks ensure ongoing approximate balance of randomisation to statin and placebo within strata.

## Study medication

Study medication comprises active treatment with atorvastatin tablets (20mg) or identical placebo. Participants are asked to take 1 tablet of study medication (20 mg of atorvastatin or placebo) per day for 4 weeks. They are then contacted by phone to assess study medication tolerability and instructed to increase the dose to two tablets per day (2x20 mg). Dose reduction of study medication to 1 tablet per day (20 mg) or temporary interruption is permitted throughout trial follow up to assist in managing tolerability and to reduce study medication discontinuation.

Atorvastatin has been chosen as one of the most commonly prescribed statins in Australia and many other countries with extensive GP experience in its use. [40] Importantly, relative to other statins, it is one of the most potent statins for reducing LDL-C with the dose of 40 mg expected to produce a 30-50% reduction. [45] The 2x20mg tablet formulation allows for dose adjustments and simplicity of supply of study product.


For peer review only - http://bmjopen.bmj.com/site/about/guidelines.xhtml

Medication is posted to participants at approximately 6 monthly intervals. Both active and placebo treatments are supplied by Ramsay Community Pharmacy and dispensed by an unblinded and independent pharmacist through a Monash University-affiliated central pharmacy dispensing centre.

**Study measures and post-randomisation follow up**

The trial activity schedule is summarized in Table 1. Participants are followed via phone or face to face visits every 6 months. At follow up visits, participants are asked about use of concomitant medications including commencement of new medications, adverse events and potential study endpoints. Supporting clinical information relating to any serious adverse events or endpoints is sourced from: a) medical record review; b) hospital records and discharge summaries; and c) linkage with national administrative death, hospitalisation and disease-specific registry datasets where possible.

A random subset of participants will also be offered the opportunity to attend STAREE sites with facilities for processing and storing biospecimens for collection of whole blood and/or urine samples, as outlined in Appendix 2a and 3.

**Adherence and retention strategies**

Adherence to medication is encouraged with multiple strategies including review of study medication use at follow up phone and face-to-face visits and through reminders included in quarterly participant newsletters and annual face-to-face or web-based study updates.

**Participant wellbeing**


For peer review only - http://bmjopen.bmj.com/site/about/guidelines.xhtml

Participants are advised to seek care from their usual treating GP (or other health care providers) for any medical condition arising during the study.  Any tests or measurements performed as part of the trial trigger a notification to the participant's GP if outside the normal range or deemed clinically significant.

**Commencement of lipid lowering therapy**

Any participants who develop a recognised clinical indication (such as a myocardial infarction or stroke) for statin treatment can take open label statin as per usual practice by their treating clinician or clinical teams and will cease study medication whilst continuing in the study for observation (routine follow-up visits).

**Study conduct amendments in response to COVID-19**

As a result of government and workplace restrictions implemented during the COVID-19 pandemic, and to ensure safety of staff and participants, amendments to the trial protocol were implemented in 2020 to enable remote screening and follow up visits by phone instead of in-person visits. After easing of COVID-19 restrictions, participants were given the option of continuing with remote screening and follow up visits or face-to-face clinic visits depending on their risk profile, personal preference and location.

**Blinding and emergency unblinding**

All STAREE staff including the investigator team, management, administration, medical and research officers and students are blinded to treatment allocation through the randomisation procedure and study duration. All GP co-investigators and practice staff will be blinded to treatment allocation. All participants will be blinded to treatment allocation for the study


For peer review only - http://bmjopen.bmj.com/site/about/guidelines.xhtml

duration. GP co-investigators are requested to not measure lipid levels in participants after randomisation, to minimise the risk of unmasking.

In the event of a clinical emergency, participants will be assumed to be on active treatment and the recommendation will be to cease study treatment if that were a requirement for statin use. The need for unblinding of study participants will be determined on a case-by-case basis after referral to a senior STAREE medical investigator who will discuss the clinical need for unblinding with the requesting clinician and then authorise the request. The process of emergency unblinding will be overseen by the Monash University-affiliated, independent pharmacist who will have access to the randomisation code.

**Endpoint definitions and adjudication**

Ascertainment of primary endpoints will be performed by Endpoint Adjudication Committees (EAC) of expert clinicians. These Committees will adjudicate events related to possible myocardial infarction, stroke, dementia, disability, heart failure, and trajectory and mode of death. Each subcommittee will have a chair and a minimum of two other members. Two members will conduct adjudications for each event and discordant cases will be resolved via consensus of all committee members.

Disability free survival is defined as survival free of dementia or physical disability as derived from the endpoints of all-cause death, dementia, and persistent physical disability. It is a composite endpoint that has been used in recent clinical trials and enables an evaluation of the net benefit of statin by capturing both beneficial and adverse effects. [35]

Dementia is defined as a significant impairment in cognitive function (≥ 2 SD below population norms; Appendix 2b) that interferes with independence in everyday activities [46]


For peer review only - http://bmjopen.bmj.com/site/about/guidelines.xhtml

or as a clinical diagnosis based on the medical record. Persistent physical disability is defined as a loss of a basic activity of daily living (ADL) as defined by Katz [47] at two consecutive visits separated by a period of 6 months. A loss of an ADL (mobility, bathing, transferring, toileting, dressing, or feeding) will equate to a self-report of "a lot of difficulty", "unable to do this activity" or a requirement for help in performing the activity. If it is not possible to obtain a Katz ADL assessment, notification of assessment for aged care services will initiate collection of relevant clinical records.

A major cardiovascular event is defined as the first occurrence of cardiovascular death or non-fatal myocardial infarction (MI) or non-fatal stroke. Cardiovascular death is defined as all deaths of cardiovascular cause including fatal MI, fatal stroke, sudden death and death due to other circulatory causes. MI is any ST and non-ST elevation event based on the fourth universal definition of MI. [48] Stroke is any ischaemic or non-ischaemic event defined by ICD-11 criteria [49] (not including transient ischaemic attack or subarachnoid haemorrhage and classified as disabling or non-disabling using the Modified Rankin Scale. Brain imaging will determine aetiology with ischaemic stroke further delineated according to the TOAST criteria. [50]

**Statistical considerations**

The number of participants needed in the trial was originally based on estimated event rates, using the ASPREE trial population which has similar demographic characteristics to the STAREE population, and on the magnitude of the treatment effect anticipated as a result of the LDL-C lowering effect of atorvastatin. [51]

**Modifications triggered by COVID-19**


For peer review only - http://bmjopen.bmj.com/site/about/guidelines.xhtml

The recruitment rate in Australia was impacted by the COVID-19 pandemic. In addition, STAREE was originally planned as an international endeavour with proposed funding and recruitment across multiple regions including Australia, however in late 2020 it became evident that the proposed funding for recruitment outside of Australia would not be secured. These developments were considered in July 2021 in finalisation of the trial design, reduction in the sample size from an original target of n=18,000 and corresponding power calculation to allow for feasible completion of the trial in Australia alone.

**Sample size**

The sample size requirements are summarised in Table 1 for detecting relative risk as hazard ratios with a two-sided significance level of 5% and adopting a closed testing procedure of the two co-primary endpoints. The key assumptions of the power calculation are:

- Loss to follow-up will occur at a rate of 2% of study participants per annum (loss to follow-up includes those due to death and is expected to be minimised through good retention of participants and the ability to access medical records or perform record linkage for participants who cease in-person assessment). Hence an average of 6.0 years of follow-up time per participant is expected by the anticipated end of the trial in December 2025, at which time the required number of co-primary endpoints are expected to have accrued.
- An age structure of STAREE participants of 56%: 27%: 12%: 5% in the respective age bands 70-74 years: 75-79 years: 80-84 years: 85+ years. These proportions are based on recruitment of the initial approximately 7,500 participants to late 2020.
- With treatment adherence statins will reduce the rate of MACE by 25% and the rate of disability-free survival by 18%.


For peer review only - http://bmjopen.bmj.com/site/about/guidelines.xhtml

- Treatment cross-over will occur and impact the hazard ratio that will be observed in intention to treat analyses. In the first year of follow-up, we assume 8% of participants randomised to statin will cease taking their study medication. Thereafter we assume 3% of participants per annum in the statin group will cease taking study medication. In the first year of follow-up, we assume 1% of participants in the placebo group will commence open-label statin. Thereafter, we assume 3% of participants per annum in the placebo group will commence statin therapy. Further, we assume that there will be no subsequent treatment "re-cross-overs”. It is assumed that the effects of statin use and non-use outside use of study medication is the same as the effects of randomised statin and placebo respectively.
- Rates of the co-primary endpoint death, dementia, or persistent physical disability (DFS) in the placebo group of 18.3 per 1000 person-years (70-74y), 33.9 per 1000 person-years (75-79y), 65.7 per 1000 person-years (80-84y), 115.4 per 1000 person-years (85+y) (See Appendix 4). To allow for a healthy participant effect at recruitment, it is assumed that these event rates will be observed from the start of the third year of an individuals’ follow-up and that in the first and second year of follow-up the corresponding rates respectively will be zero and half of that of the third year of follow-up.
- Major cardiovascular event rates in the placebo group, according to age at randomisation, of 9.5 per 1000 person-years (70-74y), 11.5 per 1000 person-years (75-79y), 18.5 per 1000 person-years (80-84y), 24.5 per 1000 person-years (85+y).

Table 1: Sample size requirement and power justification for the STAREE trial’s co-primary endpoints


For peer review only - http://bmjopen.bmj.com/site/about/guidelines.xhtml

| Co-primary endpoint | Effect size (relative reduction in risk for statin group versus placebo in intention to treat analysis based on treatment adherence assumptions) | Power | Required number of events | Required number of participants based on assumed event rates and follow-up |
|---|---|---|---|---|
| Major cardiovascular events (first occurrence of CV death, non-fatal MI or non-fatal stroke) | **20.2%** | **80.0%** | **619** | **9,631** |
| Disability free survival (first occurrence of dementia, persistent physical disability or death) | **14.5%** | **83.1%** | **1,397** | **9,631** |

**Statistical analysis**

Baseline characteristics of the two treatment groups will be tabulated and imbalance defined as a 0.25 standard deviation difference in means (quantitative characteristics) or an odds ratio of 1.5 (binary characteristics). Given the large sample size, randomisation is anticipated to adequately balance baseline characteristics of participants in the two treatment groups. Hence unadjusted analyses will be considered primary. All primary comparisons between treatment arms will be on an intention-to-treat basis i.e., according to the group to which participants were randomised and without reference to their actual compliance with assigned treatment. Each of the co-primary endpoints will be analysed separately in time-to-event analyses. Event rates (time to first event within each endpoint definition) will be compared between groups using a hazard ratio and 95% confidence interval from a Cox proportional hazards regression model fitted to the endpoint, with censoring for individuals not experiencing an endpoint event at their most recent study visit, and a single covariate being an indicator of the group to which the individual was randomised, statin or placebo. The proportional hazards assumption will be tested for each model. Loss to follow-up will be considered a censoring event. This

For peer review only


For peer review only - http://bmjopen.bmj.com/site/about/guidelines.xhtml

equates to an assumption that data is missing at random given the participant's treatment group and the timing of their loss to follow-up. The adequacy of this assumption will be checked in sensitivity analyses that will include both imputation approaches and adjustment for baseline covariates predictive of propensity for dropout.

A closed testing procedure will be used to allow for the multiple testing arising from two co-primary endpoints. This approach is based on the expectation that cardiovascular benefit will be the main contributor to improved disability free survival and that a substantial effect of statins on the latter is unlikely in the absence of an effect on the former. First, major cardiovascular events will be tested at alpha=0.05 and, if the major cardiovascular events p-value is <0.05 then second, disability-free survival will be tested at alpha=0.05. If the major cardiovascular events p-value is not <0.05 than a p-value for disability-free survival will not be presented.

Secondary analyses of the co-primary endpoints will be performed using extended Cox proportional hazards regression models based on time(s) to any event within the endpoint definition (for relevant endpoints that can occur more than once). No statistical adjustment will be made for the multiple secondary endpoints and no p-values will be presented for the statin versus placebo comparison for these endpoints. The reporting of all secondary endpoint analyses will make clear whether either of the co-primary endpoints were statistically significant.

Subgroup analyses will be undertaken by sex (female, male), age (70-74, ≥75years), systolic and diastolic blood pressure levels (terciles), body mass index (<25, 25-29.9, 30+ kg/m$^2$),


For peer review only - http://bmjopen.bmj.com/site/about/guidelines.xhtml

smoking status (never, past and current), 3MS score (above, below median), estimated GFR (<60, ≥60 mL/min per $1.73m^2$), and LDL-C and HDL-C levels (terciles).

The impacts of COVID-19 will be investigated in sensitivity analyses that include examination of participant event rates by whether their randomisation occurred pre-, peri- or post-pandemic, and consider COVID-19 vaccination status (which will be an intercurrent event for those randomised pre-pandemic and a baseline covariate for those randomised post-pandemic), following emerging guidance on analytical approaches and estimand definitions. [52]

**Data and Safety Monitoring Board (DSMB)**

An independent committee comprising experts in clinical trials, cardiology, and statistics are overseeing data accrual and safety. As outlined in the DSMB Charter (Appendix 5), the DSMB will monitor participant deaths, serious adverse events, endpoints, data quality and progress of the study. The DSMB will also review any interim analyses on unblinded data in accordance with stopping rules and will provide recommendations to the STAREE Executive Committee.

**Patient and public involvement**

STAREE has embedded mechanisms for ongoing participant and public involvement. These include a participant advisory committee, which reviewed study processes and documents, and a formal study that assessed participant preferences about the delivery of informed consent information. [53 54] Presentations to relevant community, social and professional groups have also offered the opportunity for public involvement.

For peer review only



For peer review only - http://bmjopen.bmj.com/site/about/guidelines.xhtml

During the trial, participants may provide feedback via access to a free phone line. Participants are also invited to annual trial updates and "question and answer" sessions. At the completion of the trial, participants will receive a summary of the study results and information about which treatment arm they were allocated to.

**Ethics and Dissemination**

STAREE is being conducted in accordance with the Declaration of Helsinki (1964, revised in 2008), the NHMRC Guidelines on Human Experimentation, the federal patient privacy (HIPAA) law and ICH-GCP guidelines and the International Conference of Harmonisation Guidelines for Good Clinical Practice. Institutional ethics approval has been obtained from the following:

Monash University Human Research Ethics Committee (Project ID 2787 and 21528).

Curtin University Human Research Ethics Committee (HR113/2015)

RACGP National Research and Evaluation Ethics Committee (14 - 017)

Tasmanian Health and Medical Research Ethics Committee (H0014918)

University of Newcastle Human Research Ethics Committee (H-2016-0266)

The trial is managed and co-ordinated by the STAREE steering committee (Appendix 6 which is responsible for developing and approving the protocol and operational plan. The trial central coordinating centre is based at the School of Public Health and Preventive Medicine, Monash University, 553 St Kilda Road, Melbourne 3004.

All research outputs will be disseminated to GP co-investigators and participants, published in peer-reviewed journals and presented at national and international conferences. Authorship of papers will be according to the criteria of the International Committee of Medical Journal


For peer review only - http://bmjopen.bmj.com/site/about/guidelines.xhtml

Editors and all STAREE publications will list the lead authors and writing group, and the STAREE Investigator group.

On completion of the trial, and after publication of the primary and secondary outcomes of the study, requests for access to de-identified data (to be provided through a secure online environment) may be submitted to the researchers located at the School of Public Health and Preventive Medicine, Monash University, Melbourne, Australia.

**Trial status**

Recruitment commenced in November 2015 and was expected to be completed in 2021. However, COVID-19 public health directives and work restrictions resulted in reduced access to general practices across Australia such that recruitment had to be extended for a further 18 months.

**DISCUSSION**

There is continuing debate on the utility of statins for primary prevention in older people primarily due to the lack of RCT evidence in this population. [33] While no new RCTs of statins have reported since the conception and commencement of STAREE in 2015 and completion of HOPE-3 in 2016, new analyses of existing RCT data have added to the debate. Age specific analyses of pooled data from the HOPE 3 and JUPITER RCTs have suggested significant CVD benefits for all age groups above 65 years including a 26% risk reduction in CVD events in individuals aged 70 years and over. [37] A meta-analysis of efficacy and safety of individual participant data from 28 RCTs of statin therapy that included participants over 75 years indicated non-significant 16% and 8% reductions in major vascular events per 1.0 mml/L reduction in LDL-C amongst 70 to 75 and >75 year age groups, respectively,


For peer review only - http://bmjopen.bmj.com/site/about/guidelines.xhtml

without known vascular disease. [36] However, no significant effects on vascular death, non-vascular death, cancer death or all-cause death were observed in either of these older age groups. Guidelines for statin prescription reflect this lack of robust trial evidence, with varying recommendations for apparently healthy older adults over 70 or 75 years, which have led to confusion amongst clinicians and consumers alike. In contrast, specific data exist for the use of aspirin and anti-hypertensive drugs in this population which has informed guideline committees in making recommendations for intervention. [3, 51, 55] STAREE will provide new trial evidence to address this critical gap for statins.

STAREE is a large primary prevention trial that is assessing whether starting statins in adults 70 years and older will extend independent and healthy lifespan. While STAREE captures the CVD endpoints that are the traditional primary outcomes of statin trials, we consider that a wider range of outcomes beyond major CVD events is important to evaluate the overall benefit of statins in an older population. To this end, our disability free survival outcome including detailed ascertainment of dementia using well validated measures of cognition, such as those used in the ASPREE trial [35] and advocated by others [33], will allow us to determine whether statins reduce major cardiovascular events and prolong survival free of dementia or physical disability; health outcomes that are highly valued by older people and the general population.

For peer review only


For peer review only - http://bmjopen.bmj.com/site/about/guidelines.xhtml

## Author contributions

SZ, JJM, RW, LB, GCC, TT-JC, MRN, SJN, CMR, AT, ASW and SAW developed the concept and design of the STAREE trial. SZ, AJC, SS and RW developed and wrote the study protocol. SZ, AJC, SS and RW wrote the initial drafts of the manuscript. All authors (SZ, AJC, SS, RW, JJM, LB, TT-JC, GCC, IH, AK, MRN, SJN, CMR, JR, AT, SAW, ASW) reviewed and revised further drafts of the manuscript and read and approved the final manuscript.

## Funding statement

STAREE is sponsored by Monash University and has received funding from the National Health and Medical Research Council (NHMRC APP1068146 and APP1161503) and the Heart Foundation of Australia (HF Stroke Prevention grant). STAREE MIND and STAREE HEART have been awarded NHMRC grant funding (#2006611 and #1165440). The funders have had no role in the study design, collection, management, analysis and interpretation of data or in writing or submission of reports or publications.

For peer review only


For peer review only - http://bmjopen.bmj.com/site/about/guidelines.xhtml

**Competing Interests**

SZ has received NHMRC and Australian Heart Foundation research funding as the principal investigator of the STAREE trial; and payment to the institution (Monash University) from Amgen Australia, AstraZeneca, Boehringer-Ingelheim, Eli Lilly Australia, Merck Sharp & Dohme Australia, Novo Nordisk, Sanofi, and Servier for consultancy work outside the submitted work.

IH has received research funding from NHMRC and Royal Australasian College of Physicians and honoraria for lectures or advisory board participation from Boehringer Ingelheim, Vifor and Eli Lilly.

JJM is supported by a NHMRC Leadership Fellowship (IG1173690).

MRN has served on a Novartis advisory board on lipid management in 2020.

CMR is supported through a NHMRC Principal Research Fellowship (APP 1136372).

SJN has received research support from AstraZeneca, New Amsterdam Pharma, Amgen, Anthera, Eli Lilly, Esperion, Novartis, Cerenis, The Medicines Company, Resverlogix, InfraReDx, Roche, Sanofi-Regeneron and LipoScience and is a consultant for AstraZeneca, Amarin, Akcea, Eli Lilly, Anthera, Omthera, Merck, Takeda, Resverlogix, Sanofi-Regeneron, CSL Behring, Esperion, Boehringer Ingelheim and Vaxxinity.

TC has received honoraria for lectures from Roche.

ASW has been a clinical trial investigator for Akcea, Amgen, Regeneron and Silence Therapeutics. He has served as a guideline chair and done consultancy work for the National Institute of Health and Care Excellence in the UK.

AT has received research support or honoraria for lectures, advisory board or data monitoring committee participation from Amgen, The Medicines Group, Merck, Novartis and Pfizer.

SW has received payment to attend an advisory meeting on dementia for Roche.

For peer review only


For peer review only - http://bmjopen.bmj.com/site/about/guidelines.xhtml

## References

1. Bongaarts J. Human population growth and the demographic transition. *Philos Trans R Soc Lond B Biol Sci* 2009;364(1532):2985-90. doi: 10.1098/rstb.2009.0137 [published Online First: 2009/09/23]
2. Christensen K, Doblhammer G, Rau R, et al. Ageing populations: the challenges ahead. *Lancet* 2009;374(9696):1196-208. doi: 10.1016/S0140-6736(09)61460-4 [published Online First: 2009/10/06]
3. Jacobsen L, Kent M, Lee M, et al. America's aging population. *Population Buletin* 2011;66(1)
4. Spiers GF, Kunonga TP, Beyer F, et al. Trends in health expectancies: a systematic review of international evidence. *BMJ Open* 2021;11(5):e045567. doi: 10.1136/bmjopen-2020-045567 [published Online First: 2021/05/27]
5. Welsh CE, Matthews FE, Jagger C. Trends in life expectancy and healthy life years at birth and age 65 in the UK, 2008-2016, and other countries of the EU28: An observational cross-sectional study. *Lancet Reg Health Eur* 2021;2:100023. doi: 10.1016/j.lanepe.2020.100023 [published Online First: 2021/04/20]
6. North BJ, Sinclair DA. The intersection between aging and cardiovascular disease. *Circ Res* 2012;110(8):1097-108. doi: 10.1161/CIRCRESAHA.111.246876 [published Online First: 2012/04/14]
7. van der Flier WM, Scheltens P. Epidemiology and risk factors of dementia. *J Neurol Neurosurg Psychiatry* 2005;76 Suppl 5:v2-7. doi: 10.1136/jnnp.2005.082867 [published Online First: 2005/11/18]
8. Gale CR, Cooper C, Sayer AA. Prevalence of frailty and disability: findings from the English Longitudinal Study of Ageing. *Age Ageing* 2015;44(1):162-5. doi: 10.1093/ageing/afu148 [published Online First: 2014/10/15]
9. Fong JH. Disability incidence and functional decline among older adults with major chronic diseases. *BMC Geriatr* 2019;19(1):323. doi: 10.1186/s12877-019-1348-z [published Online First: 2019/11/23]
10. Maresova P, Javanmardi E, Barakovic S, et al. Consequences of chronic diseases and other limitations associated with old age - a scoping review. *BMC Public Health* 2019;19(1):1431. doi: 10.1186/s12889-019-7762-5 [published Online First: 2019/11/05]
11. Quine S, Morrell S. Fear of loss of independence and nursing home admission in older Australians. *Health Soc Care Community* 2007;15(3):212-20. doi: 10.1111/j.1365-2524.2006.00675.x [published Online First: 2007/04/21]
12. Fried TR, Bradley EH, Williams CS, et al. Functional disability and health care expenditures for older persons. *Arch Intern Med* 2001;161(21):2602-7. doi: 10.1001/archinte.161.21.2602 [published Online First: 2001/12/26]
13. Wolff JL, Starfield B, Anderson G. Prevalence, expenditures, and complications of multiple chronic conditions in the elderly. *Arch Intern Med* 2002;162(20):2269-76. doi: 10.1001/archinte.162.20.2269 [published Online First: 2002/11/07]
14. El-Hayek YH, Wiley RE, Khoury CP, et al. Tip of the iceberg: Assessing the global socioeconomic costs of Alzheimer's Disease and related dementias and strategic implications for stakeholders. *J Alzheimers Dis* 2019;70(2):323-41. doi: 10.3233/JAD-190426 [published Online First: 2019/07/01]
15. Cholesterol Treatment Trialists C, Mihaylova B, Emberson J, et al. The effects of lowering LDL cholesterol with statin therapy in people at low risk of vascular disease: meta-analysis of individual data from 27 randomised trials. *Lancet*


For peer review only - http://bmjopen.bmj.com/site/about/guidelines.xhtml

2012;380(9841):581-90. doi: 10.1016/S0140-6736(12)60367-5 [published Online First: 2012/05/23]
16. Prospective Studies C, Lewington S, Whitlock G, et al. Blood cholesterol and vascular mortality by age, sex, and blood pressure: a meta-analysis of individual data from 61 prospective studies with 55,000 vascular deaths. *Lancet* 2007;370(9602):1829-39. doi: 10.1016/S0140-6736(07)61778-4 [published Online First: 2007/12/07]
17. Jonsson A, Sigvaldason H, Sigfusson N. Total cholesterol and mortality after age 80 years. *Lancet* 1997;350(9093):1778-9. doi: 10.1016/S0140-6736(05)63609-4 [published Online First: 1997/12/31]
18. Schatz IJ, Masaki K, Yano K, et al. Cholesterol and all-cause mortality in elderly people from the Honolulu Heart Program: a cohort study. *Lancet* 2001;358(9279):351-5. doi: 10.1016/S0140-6736(01)05553-2 [published Online First: 2001/08/15]
19. Volpato S, Leveille SG, Corti MC, et al. The value of serum albumin and high-density lipoprotein cholesterol in defining mortality risk in older persons with low serum cholesterol. *J Am Geriatr Soc* 2001;49(9):1142-7. doi: 10.1046/j.1532-5415.2001.49229.x [published Online First: 2001/09/18]
20. Schupf N, Costa R, Luchsinger J, et al. Relationship between plasma lipids and all-cause mortality in nondemented elderly. *J Am Geriatr Soc* 2005;53(2):219-26. doi: 10.1111/j.1532-5415.2005.53106.x [published Online First: 2005/01/28]
21. Weverling-Rijnsburger AW, Jonkers IJ, van Exel E, et al. High-density vs low-density lipoprotein cholesterol as the risk factor for coronary artery disease and stroke in old age. *Arch Intern Med* 2003;163(13):1549-54. doi: 10.1001/archinte.163.13.1549 [published Online First: 2003/07/16]
22. Foody JM, Wang Y, Kiefe CI, et al. Long-term prognostic importance of total cholesterol in elderly survivors of an acute myocardial infarction: the Cooperative Cardiovascular Pilot Project. *J Am Geriatr Soc* 2003;51(7):930-6. doi: 10.1046/j.1365-2389.2003.51305.x [published Online First: 2003/07/02]
23. Zingel R, Bohlken J, Riedel-Heller S, et al. Association between low-density lipoprotein cholesterol levels, statin use, and dementia in patients followed in German general practices. *J Alzheimers Dis* 2021;79(1):37-46. doi: 10.3233/JAD-201176 [published Online First: 2020/11/21]
24. Liang Y, Vetrano DL, Qiu C. Serum total cholesterol and risk of cardiovascular and non-cardiovascular mortality in old age: a population-based study. *BMC Geriatr* 2017;17(1):294. doi: 10.1186/s12877-017-0685-z [published Online First: 2017/12/29]
25. Pencina MJ, Navar AM, Wojdyla D, et al. Quantifying importance of major risk factors for coronary heart disease. *Circulation* 2019;139(13):1603-11. doi: 10.1161/CIRCULATIONAHA.117.031855 [published Online First: 2018/12/28]
26. Silverman MG, Ference BA, Im K, et al. Association between lowering LDL-C and cardiovascular risk reduction among different therapeutic interventions: A systematic review and meta-analysis. *JAMA* 2016;316(12):1289-97. doi: 10.1001/jama.2016.13985 [published Online First: 2016/09/28]
27. Greenwood J, Mason JC. Statins and the vascular endothelial inflammatory response. *Trends Immunol* 2007;28(2):88-98. doi: 10.1016/j.it.2006.12.003 [published Online First: 2007/01/02]
28. Zhou Z, Ofori-Asenso R, Curtis AJ, et al. Association of statin use with disability-free survival and cardiovascular disease among healthy older adults. *J Am Coll Cardiol* 2020;76(1):17-27. doi: 10.1016/j.jacc.2020.05.016 [published Online First: 2020/07/04]


For peer review only - http://bmjopen.bmj.com/site/about/guidelines.xhtml

29. Stancu C, Sima A. Statins: mechanism of action and effects. *J Cell Mol Med* 2001;5(4):378-87. doi: 10.1111/j.1582-4934.2001.tb00172.x [published Online First: 2002/06/18]
30. Menge T, Hartung HP, Stuve O. Statins--a cure-all for the brain? *Nat Rev Neurosci* 2005;6(4):325-31. doi: 10.1038/nrn1652 [published Online First: 2005/04/02]
31. Bagheri H, Ghasemi F, Barreto GE, et al. The effects of statins on microglial cells to protect against neurodegenerative disorders: A mechanistic review. *Biofactors* 2020;46(3):309-25. doi: 10.1002/biof.1597 [published Online First: 2019/12/18]
32. Gurwitz JH, Go AS, Fortmann SP. Statins for primary prevention in older adults: Uncertainty and the Need for More Evidence. *JAMA* 2016;316(19):1971-72. doi: 10.1001/jama.2016.15212 [published Online First: 2016/11/14]
33. Singh S, Zieman S, Go AS, et al. Statins for primary prevention in older adults-moving toward evidence-based decision-making. *J Am Geriatr Soc* 2018;66(11):2188-96. doi: 10.1111/jgs.15449 [published Online First: 2018/10/03]
34. Zoungas S, Curtis A, Tonkin A, et al. Statins in the elderly: an answered question? *Curr Opin Cardiol* 2014;29(4):372-80. doi: 10.1097/HCO.0000000000000082 [published Online First: 2014/05/29]
35. McNeil JJ, Woods RL, Nelson MR, et al. Effect of aspirin on disability-free survival in the healthy elderly. *N Engl J Med* 2018;379(16):1499-508. doi: 10.1056/NEJMoa1800722 [published Online First: 2018/09/18]
36. Cholesterol Treatment Trialists C. Efficacy and safety of statin therapy in older people: a meta-analysis of individual participant data from 28 randomised controlled trials. *Lancet* 2019;393(10170):407-15. doi: 10.1016/S0140-6736(18)31942-1 [published Online First: 2019/02/05]
37. Ridker PM, Lonn E, Paynter NP, et al. Primary prevention with statin therapy in the elderly: New meta-analyses from the contemporary JUPITER and HOPE-3 randomized trials. *Circulation* 2017;135(20):1979-81. doi: 10.1161/CIRCULATIONAHA.117.028271 [published Online First: 2017/04/08]
38. McGuinness B, O'Hare J, Craig D, et al. Cochrane review on 'Statins for the treatment of dementia'. *Int J Geriatr Psychiatry* 2013;28(2):119-26. doi: 10.1002/gps.3797 [published Online First: 2012/04/05]
39. Routledge PA, O'Mahony MS, Woodhouse KW. Adverse drug reactions in elderly patients. *Br J Clin Pharmacol* 2004;57(2):121-6. doi: 10.1046/j.1365-2125.2003.01875.x [published Online First: 2004/01/30]
40. Buettner C, Davis RB, Leveille SG, et al. Prevalence of musculoskeletal pain and statin use. *J Gen Intern Med* 2008;23(8):1182-6. doi: 10.1007/s11606-008-0636-7 [published Online First: 2008/05/02]
41. Collins R, Reith C, Emberson J, et al. Interpretation of the evidence for the efficacy and safety of statin therapy. *Lancet* 2016;388(10059):2532-61. doi: 10.1016/S0140-6736(16)31357-5 [published Online First: 2016/09/13]
42. Zhou Z, Albarqouni L, Curtis AJ, et al. The safety and tolerability of statin therapy in primary prevention in older adults: A systematic review and meta-analysis. *Drugs Aging* 2020;37(3):175-85. doi: 10.1007/s40266-019-00736-y [published Online First: 2020/01/11]
43. Pirmohamed M, James S, Meakin S, et al. Adverse drug reactions as cause of admission to hospital: prospective analysis of 18 820 patients. *BMJ* 2004;329(7456):15-9. doi: 10.1136/bmj.329.7456.15 [published Online First: 2004/07/03]
44. Brugts JJ, Yetgin T, Hoeks SE, et al. The benefits of statins in people without established cardiovascular disease but with cardiovascular risk factors: meta-analysis of

For peer review only


For peer review only - http://bmjopen.bmj.com/site/about/guidelines.xhtml

randomised controlled trials. *BMJ* 2009;338:b2376. doi: 10.1136/bmj.b2376 [published Online First: 2009/07/02]

45. Naci H, Brugts JJ, Fleurence R, et al. Dose-comparative effects of different statins on serum lipid levels: a network meta-analysis of 256,827 individuals in 181 randomized controlled trials. *Eur J Prev Cardiol* 2013;20(4):658-70. doi: 10.1177/2047487313483600 [published Online First: 2013/03/27]
46. American Psychiatric A. Neurocognitive Disorders. In *Diagnostic and statistical manual of mental disorders : DSM-5-TR*. Fifth edition, text revision. ed: Washington, DC : American Psychiatric Association Publishing 2022.
47. Katz, S. Assessing self-maintenance: Activities of daily living, mobility, and instrumental activities of daily living. *Journal of the American Geriatrics Society.* 1983. 31(12), 721–727.
48. Thygesen K, Alpert JS, Jaffe AS, et al. Fourth universal definition of myocardial infarction (2018). *J Am Coll Cardiol* 2018;72(18):2231-64. doi: 10.1016/j.jacc.2018.08.1038 [published Online First: 2018/08/30]
49. Feigin V, Norrving B, Sudlow CLM, et al. Updated Criteria for Population-Based Stroke and Transient Ischemic Attack Incidence Studies for the 21st Century. *Stroke.* 2018;49(9):2248-2255. doi: 10.1161/STROKEAHA.118.022161.
50. Adams HP, Jr., Bendixen BH, Kappelle LJ, et al. Classification of subtype of acute ischemic stroke. Definitions for use in a multicenter clinical trial. TOAST. Trial of Org 10172 in Acute Stroke Treatment. *Stroke* 1993;24(1):35-41. doi: 10.1161/01.str.24.1.35 [published Online First: 1993/01/01]
51. McNeil JJ, Wolfe R, Woods RL, et al. Effect of aspirin on cardiovascular events and bleeding in the healthy elderly. *N Engl J Med* 2018;379(16):1509-18. doi: 10.1056/NEJMoa1805819 [published Online First: 2018/09/18]
52. Van Lancker K, Tarima S, Bartlett J, et al. Estimands and their Estimators for Clinical Trials Impacted by the COVID-19 Pandemic: A Report from the NISS Ingram Olkin Forum Series on Unplanned Clinical Trial Disruptions. Statistics in Biopharmaceutical Research, DOI: 10.1080/19466315.2022.2094459
53. O'Hare F, Flanagan Z, Nelson M, et al. Comparing two methods for delivering clinical trial informed consent information to older adults: singular versus stepped approach. *Clin Trials* 2018;15(6):610-15. doi: 10.1177/1740774518793377 [published Online First: 2018/08/04]
54. O'Hare F, Spark S, Flanagan Z, et al. Impact of informed consent content and length on recruitment of older adults into a community based primary prevention trial. *Contemp Clin Trials Commun* 2018;11:89-94. doi: 10.1016/j.conctc.2018.05.003 [published Online First: 2018/07/14]
55. Beckett NS, Peters R, Fletcher AE, et al. Treatment of hypertension in patients 80 years of age or older. *N Engl J Med* 2008;358(18):1887-98. doi: 10.1056/NEJMoa0801369 [published Online First: 2008/04/02]

For peer review only


For peer review only - http://bmjopen.bmj.com/site/about/guidelines.xhtml

**Appendix 1. WHO Trial Registration Data Set**

| | Data Category | Information |
|---|---|---|
| 1 | Primary Registry and Trial Identifying Number | ClinicalTrials.gov<br>NCT02099123 |
| 2 | Date of Registration in Primary Registry | 28 March 2014 |
| 3 | Secondary Identifying Numbers | |
| 4 | Source(s) of Monetary or Material Support | National Health and Medical Research Council of Australia<br>Heart Foundation (Australia) |
| 5 | Primary Sponsor | Monash University |
| 6 | Secondary Sponsor(s) | Not applicable |
| 7 | Contact for Public Queries | staree@monash.edu |
| 8 | Contact for Scientific Queries | Professor Sophia Zoungas (Principal Investigator)<br>sophia.zoungas@monash.edu<br>Phone: +61 (3) 9903 0711<br>Address: Level 5/99 Commercial Rd, Melbourne 3004 |
| 9 | Public Title | STAtin therapy for Reducing Events in the Elderly (STAREE) trial |
| 10 | Scientific Title | A randomised clinical trial of STAtin therapy for Reducing Events in the Elderly |
| 11 | Countries of Recruitment | Australia |
| 12 | Health Condition(s) or Problem(s) Studied | Prevention of cardiovascular disease, dementia and disability |
| 13 | Intervention(s) | Atorvastatin (2 x 20 mg/day) |
| | | Identical Placebo (2 tablets/day) |
| 14 | Key Inclusion and Exclusion Criteria | Inclusion criteria: Age ≥70 years, living independently in the community, willing and able to provide informed consent and accept the study requirements |
| | | Exclusion criteria: History of clinical cardiovascular events, diabetes or dementia; Modified Mini-Mental State Examination (3MS) score <78 on screening; moderate or severe chronic kidney disease; moderate or severe liver disease; serious inter-current illness likely to cause death within the next 5 years; total cholesterol >7.5 mmol/L; current participation in another interventional clinical trial; and an absolute contraindication to statin therapy; current use of statin therapy or other lipid lowering therapy for primary prevention and unwilling to stop therapy; or current long term or permanent use of potent cytochrome P450 (CYP) 3A4 inhibitors. |
| 15 | Study Type | Interventional |
| | | Allocation: randomised |
| | | Double blind |
| | | Primary purpose: Phase IV study |
| 16 | Date of First Enrolment | November 2015 |
| 17 | Sample Size | 9631 |
| 18 | Recruitment Status | Recruiting |

For peer review only

| | | |
|---|---|---|
| 19 | Primary Outcome(s) | i) a composite of cardiovascular death, non-fatal myocardial infarction, or non-fatal stroke; and<br>ii) a composite of all-cause death or dementia or development of persistent physical disability. |
| 20 | Key Secondary Outcomes | All cause death, dementia, other cognitive impairment, persistent physical disability, fatal and non-fatal myocardial infarction, fatal and non-fatal stroke, cardiovascular death, fatal and non-fatal cancer, heart failure hospitalisation, atrial fibrillation, all-cause hospitalisation, need for permanent residential care, quality of life |
| 21 | Ethics Review | Monash University Human Research Ethics Committee (Project ID 2787 and 21528)<br>Curtin University Human Research Ethics Committee (HR113/2015)<br>RACGP National Research and Evaluation Ethics Committee (14 - 017)<br>Tasmanian Health and Medical Research Ethics Committee (H0014918)<br>University of Newcastle Human Research Ethics Committee (H-2016-0266) |
| 22 | Completion date | Anticipated end 2025 |
| 23 | Summary Results | Not applicable |
| 24 | IPD sharing statement | Requests for access to de-identified data (to be provided through a secure online environment) may be submitted to the researchers located at the School of Public Health and Preventive Medicine, Monash University, Melbourne, Australia. |

For peer review only

# A randomised clinical trial of STAtin therapy for Reducing Events in the Elderly (STAREE)

PROTOCOL

Version 3.1

**Protocol authors:**

Professor Sophia Zoungas, Dr Andrea Curtis, Dr Simone Spark, Professor Rory Wolfe

**Affiliation:**

School of Public Health and Preventive Medicine, Monash University, VIC, Australia

For peer review only

**Contact for public enquiries**

Phone: 1800 770 664

Email: staree@monash.edu

Fax: +61 (3) 9903 0737

**Contact for scientific enquiries**

Professor Sophia Zoungas (Principal Investigator)

sophia.zoungas@monash.edu

Phone: +61 (3) 9903 0711

Address: Level 5/99 Commercial Rd, Melbourne 3004

**Recruitment Status:** Recruiting


For peer review only - http://bmjopen.bmj.com/site/about/guidelines.xhtml

**Acronyms used in this protocol**:

**ABS**: Australian Bureau of Statistics

**ACAP**: Aged-care assessment dataset

**ACR**: Albumin to creatinine ratio

**ADR**: Adverse Drug Reaction

**ADCS-IADL**: Alzheimer's Disease Cooperative Study Instrumental Activities of Daily Living

**AE**: Adverse events

**ASPREE**: ASPirin in Reducing Events in the Elderly

**CAM**: Confusion Assessment Method

**CES-D-10**: Centre for Epidemiologic Studies Depression Scale – 10 items

CERAD: Consortium to Establish a Registry for Alzheimer's Disease

**COWAT**: Controlled Oral Word Association Test

**CRF**: Case Report Form

**DMC**: Data Management Committee

**DOHA**: Department of Health and Ageing

**DSMB**: Data Safety and Monitoring Board

**EAC**: End-point Adjudication Committee

**GCP**: Good Clinical Practice

**HVLT-R**: Hopkins Verbal Learning Test – Revised

**HDL**: High Density Lipoprotein

**HIPAA**: Health Insurance Portability and Accountability Act of 1996

**IDL**: Intermediate Density Lipoprotein

**IPAQ-E**: International Physical Activity Questionnaire modified for the elderly

**LDL**: Low Density Lipoprotein

**MI**: Myocardial Infarction

**NCD** : Neurocognitive Disorder

**SAE**: Serious Adverse Event

**SDMT**: Symbol Digit Modalities Test

**SOP**: Standard Operating Procedure

**STAREE**: STAtin therapy for Reducing Events in the Elderly

**3MS**: Modified Mini-Mental State test

**TICS-M**: Telephone Interview of Cognitive Status – modified version

**VLDL**: Very Low Density Lipoprotein

For peer review only


For peer review only - http://bmjopen.bmj.com/site/about/guidelines.xhtml

REVISIONS

| No. | Changes |
|---|---|
| Version 1.2.2 | Original approved version |
| Version 1.3 | Changes to Study Activity Schedule including addition of cognitive tests, CESD10, and biobank sample collection |
| Version 1.3.1 | SF-12 changed to SF-36; Biobank Steering Committee added to Governance structure |
| Version 1.3.2 | Study medication: change to post out details and addition of medication compliance questionnaire |
| Version 1.3.3 | Addition of International Steering Committee; Updated exclusion criteria regarding cytochrome P450 3A4 inhibitors; Change in biobank study samples collected; |
| Version 2.0 | Updates to align protocol with SPIRIT 2013 statement recommendations |
| | Updates and clarification of primary and secondary endpoints; including change in dementia definition from DSM IV to DSM V |
| | Updated exclusion criteria regarding cytochrome P450 3A4 inhibitors |
| Version 3.0 | Response to COVID-19 – schedule update |
| | Changes to power calculations, sample size and follow up period |
| Version 3.1 | Addition of Frailty phenotype and functional independence as tertiary endpoints |


For peer review only - http://bmjopen.bmj.com/site/about/guidelines.xhtml

Contents

Contents ....................................................................................................4
1. Executive Summary ....................................................................................8
2. Introduction ...............................................................................................9
2.1 Rationale ....................................................................................................9
2.2 Statins.......................................................................................................10
2.3 Other health outcomes ...........................................................................12
2.4 Statins in the Elderly: potential risks ....................................................12
2.4.1 Myopathy ..............................................................................................12
2.4.2 Diabetes ................................................................................................13
2.4.3 Cancer ...................................................................................................13
2.4.4 Cognitive impairment ..........................................................................13
2.4.5 Liver Injury ...........................................................................................13
2.5 Utilisation .................................................................................................14
2.6 Implications of Guidelines on Treatment of Cholesterol in the Elderly ........14
2.7 Summary of rationale ..............................................................................16
3. Study Objectives .......................................................................................17
3.1 Primary objectives ...................................................................................17
3.2 Secondary objectives ...............................................................................17
3.3 Tertiary objectives ...................................................................................17
4. Hypotheses ................................................................................................18
4.1 Primary null hypotheses ..........................................................................18
4.2 Primary alternative hypotheses ...............................................................18
5. Study Design...............................................................................................19
5.1 Trial Design...............................................................................................19
5.2 Study Population.......................................................................................19
5.2.1 General considerations ........................................................................19
5.2.2 Inclusion criteria....................................................................................19
5.2.3 Exclusion criteria ...................................................................................19
5.2.4 Rescreening or reassessment ..............................................................20
5.3 Study Intervention and Choice of Comparator........................................20
5.3.1 Intervention ..........................................................................................20
5.3.2 Rationale for choice of atorvastatin ....................................................20
5.4 Participant recruitment ...........................................................................21


For peer review only - http://bmjopen.bmj.com/site/about/guidelines.xhtml

5.4.1 Recruitment ...... 21
5.4.2 Setting ...... 21
5.4.3 Community participants ...... 22
5.4.4 STAREE hub locations ...... 22
5.5 Participant timeline and schedule of assessments ...... 22
5.6 Baseline Screening Phase ...... 22
5.6.1 Screening call ...... 22
5.6.2 Lifestyle profile and screening visits (Baseline visits) ...... 26
5.6.3 Post screening GP/medical visit (Run-in phase between Baseline visits 1 and 2) . 26
5.6.4 Post screening visit study activity (Run-in phase between Baseline visits 1 and 2) ...... 26
5.6.5 Determinations of Eligibility and Assessments visit (Baseline Visit 2) ...... 26
5.7 Randomisation and follow up phase ...... 27
5.7.1 Randomisation procedure ...... 27
5.7.2 Post randomisation procedure ...... 27
5.7.3 Blinding ...... 28
5.7.4 Annual Follow up visit ...... 28
5.7.5 Phone Contact ...... 28
5.7.6 Other Actions ...... 29
5.8 Economic Analysis ...... 29
6. Study Endpoints ...... 29
6.1 Primary endpoints ...... 29
6.2 Secondary endpoints ...... 30
6.3 Tertiary endpoints ...... 30
6.3 Endpoint ascertainment ...... 30
6.4 Endpoint adjudication process ...... 31
7. Sub-studies and ancillary studies ...... 31
7.1 STAREE-HEART ...... 31
7.3 STAREE Healthy Ageing Biobank ...... 32
8. Contingency plans for participant wellbeing ...... 33
8.1 Commencement of regular lipid lowering agents ...... 33
8.2 Development of an indication for statins ...... 33
8.3 Post-trial care ...... 33
9. Levels of participant involvement ...... 34
9.1 Levels of participant involvement ...... 34
9.2 Withdrawal ...... 35

For peer review only



For peer review only - http://bmjopen.bmj.com/site/about/guidelines.xhtml

10. Study Medication and Supplies ....36
10.1 Study treatments ....36
10.2 Administration of study medication ....36
10.3 Dispensing and randomisation ....36
10.4 Study medication compliance ....37
10.5 Study medication storage ....37
10.6 Study medication accountability and dispensing logs ....38
10.7 Study medication disposal ....38
10.8 Overdose of study medication ....38
11. Concomitant Therapy ....39
11.1 Prescription medications ....39
12. Sample Size and Power calculations ....40
13. Safety Monitoring and Reporting ....44
13.1 Introduction ....44
13.2 Safety Monitoring ....44
13.2.2 Adverse events (AE) ....44
13.2.3 Serious adverse events (SAE) ....44
13.2.4 Suspected Unexpected Serious Adverse Events (SUSARs) ....45
13.3 Safety reporting ....46
13.4 Adverse Events of Interest ....46
13.4.1 New diagnosis of diabetes ....46
13.4.2 New diagnosis of myopathy ....46
13.4.3 Liver impairment ....47
14. Adherence to Ethical, Regulatory and Administrative Considerations ....48
14.1 Ethical considerations ....48
14.1.1 General ....48
14.1.2 Ethics Committee Approval ....48
14.1.3 Information for participants ....48
14.1.4 Informed consent ....48
14.1.5 Protocol Amendments ....48
14.1.6 Confidentiality ....49
14.2 Regulatory considerations ....49
14.2.1 Financing ....49
14.2.2 Trial registration ....49
14.2.3 Declaration of interest ....49

For peer review only


For peer review only - http://bmjopen.bmj.com/site/about/guidelines.xhtml

14.3 Indemnity ........ 49
14.4 Data Access ........ 50
14.5 Governance ........ 50
14.6 Administrative Organisation ........ 51
14.6.1 Individual and committee responsibilities ........ 51
14. Data Management and Training ........ 55
14.1 Case report forms ........ 55
14.2 Data Management ........ 55
14.3 Data analysis ........ 56
14.4 Training ........ 57
14.4.1 Research Officers ........ 57
14.4.2 GP Liaison Officers ........ 57
14.4.3 Research Field Officers ........ 57
15. Study Monitoring and Auditing ........ 58
16. Dissemination Plan ........ 59
17. References ........ 60



For peer review only - http://bmjopen.bmj.com/site/about/guidelines.xhtml

## 1. Executive Summary

This protocol describes the background, rationale, study design, processes and governance arrangements for the Statins in Reducing Events in the Elderly (STAREE) trial and its sub-studies.

STAREE is a double blind, randomised placebo-controlled parallel group trial investigating whether statin therapy can prolong good health and maintain independence amongst older people. STAREE will recruit and randomise 9,631 Australian participants aged 70 years and over who are free from cardiovascular disease, diabetes and dementia and living independently in the community.

The STAREE trial will compare the effects of atorvastatin (2 x 20 mg/day) or identical placebo on two co-primary outcomes, a composite of all cause death, dementia or disability and a composite of cardiovascular death, non-fatal myocardial infarction or non-fatal stroke. Secondary outcomes include all cause death, cardiovascular death, dementia, other cognitive impairment, disability, all cause hospitalisation, need for permanent residential care, fatal and non-fatal myocardial infarction, fatal and non-fatal stroke (thromboembolic and haemorrhagic), fatal and non-fatal cancer, heart failure and quality of life. The cost effectiveness of statin therapy for primary prevention in people aged 70 years and over will also be assessed.

For peer review only


For peer review only - http://bmjopen.bmj.com/site/about/guidelines.xhtml

# 2. Introduction

## 2.1 Rationale

### Ageing Population

The developed world is experiencing a rapid demographic transition towards an older population.[1] Increasing survival amongst older age groups is a major contributor to this shift, with almost twice as many older people surviving into their eighties and nineties compared to fifty years ago.[2]

In the US, the proportion of the population aged 65 and over is projected to increase from 40 million in 2009 to 89 million in 2050 with 19 million (20%) in the oldest age group (85+) by 2050.[3] Similar trends are anticipated in other developed nations such as Australia, Canada and the United Kingdom.[2]

As age advances quality of life is increasingly affected by frailty, cognitive decline/dementia and the consequences of a variety of chronic diseases including cardiovascular disease, arthritis, osteoporosis and malignancy. Although preventive therapies have reduced the burden of chronic diseases at younger ages there is limited evidence that these advances have improved the health of older people.[2] As a result, the majority of older individuals ultimately progress to residential care. In Australia, at 75 years of age the remaining lifetime need for residential nursing care is estimated at 72% for women and 53% for men, at a cost of approximately $7 billion Australian dollars per annum.[4]

The gains that have been made in increasing quantity of life have not been accompanied by similar gains in quality of life, for the additional later years of life lived. The critical question is therefore one of the balance of any benefits and harms of preventive therapies for older people.

One promising but inadequately tested preventive approach to extending life and maintaining independence in the elderly is statin therapy. In people with existing cardiovascular disease (secondary prevention), the benefits of statin therapy are clear with the risks of cardiovascular events reduced across all ages. In people without cardiovascular disease (primary prevention), the benefits of taking a statin are not clear for older people. Most national and international guidelines have not been able to make strong recommendations to guide prescribing for older people and any recommendations have generally been informed by trials of statins in younger people (<70 years) or trials of statins that have not studied the range of outcomes important to older people.[5]

### Cholesterol and Clinical Outcomes in the Elderly

While high levels of total cholesterol or LDL cholesterol are known to increase the risk of mortality among young and middle-aged populations, the relationship between total cholesterol or LDL cholesterol and all-cause or cause-specific death in older populations is not consistent. Some observational studies have reported no association or a negative association between total cholesterol or LDL cholesterol levels and all-cause death[6-11] whilst others have reported a positive linear association between total cholesterol and LDL cholesterol levels and cardiovascular death.[12] One explanation for the discrepancy is "reverse


For peer review only - http://bmjopen.bmj.com/site/about/guidelines.xhtml

causality," whereby unquantified comorbid disease may be causing both lower total cholesterol or LDL cholesterol levels and an increased risk of death, thereby creating an apparent lack of association or negative association.

Given the uncertainty around optimal total and LDL cholesterol levels in the elderly, the decision as to whether statins should be initiated or even continued must be guided by randomised controlled trials.

## 2.2 Statins

### Pharmacological effects

Statins (HMG-CoA reductase inhibitors) are a class of drug with the potential to delay the onset of several causes of incapacity in the elderly. Their best-recognised pharmacological action is to inhibit the mevalonate pathway and biosynthesis of cholesterol and isoprenoids by binding to HMG-CoA reductase and displacing the natural substrate HMG-CoA. This interaction prevents HMG-CoA reductase from attaining a functional conformation, stops the conversion of HMG-CoA to l-mevalonate and produces a reduction in intracellular cholesterol.[13] LDL-receptor gene expression and formation on the hepatocyte cell surface are then induced. The result is an increase in LDL cholesterol extraction (and its precursors, IDL and VLDL) from the blood and decrease in circulating LDL cholesterol levels (by 18-55%).[13] Beneficial effects on other lipid parameters have also been reported including increased HDL cholesterol (by 5-15%),[14] decreased levels of atherogenic lipoproteins, [15] decreased triglyceride concentrations (7-30%) and decreased synthesis and secretion of triglyceride-rich lipoproteins[15] as well as reduced susceptibility of LDL cholesterol to oxidation.[13]

The reduction in circulating lipid levels underpins the ability of statins to reduce the incidence of myocardial infarction and stroke and their long-term sequelae (cardiac failure, disability, cognitive decline). Statins also exert a number of pleiotropic effects including anti-oxidative, anti-thrombotic and a moderately powerful anti-inflammatory action.[16] The pleiotropic effects of statins have been attributed to the inhibition of isoprenoid metabolite formation (geranylgeranyl pyrophosphate and farnesyl pyrophosphate).[13] These metabolites are intermediates in the post-translational formation of several cell-signalling proteins that control multiple cell functions including maintenance of cell shape, motility, factor secretion, differentiation, and proliferation thus explaining improvements in endothelial function, vascular inflammation and oxidation, and atherosclerotic plaque stability.[13]

### Clinical effects on Major Vascular Events and Death

The impact of statin therapy on cardiovascular events has been studied in primary (mostly high risk) and secondary prevention settings. A recent meta-analysis of pooled individual participant data from 28 trials of statin versus control/placebo including participants of ages 55 years and over has reported a 21% reduction in risk of major vascular events per 1 mmol/L reduction in LDL cholesterol, attributed to reduced risks of major coronary events, major stroke events and vascular death.[17]

In an analysis of mixed populations (both primary and secondary prevention), there was a trend for decreasing reductions in major vascular events with increasing age. Analyses by age group indicated risk reductions of major vascular events of 19% and 13% per 1 mmol/L


For peer review only - http://bmjopen.bmj.com/site/about/guidelines.xhtml

reduction in LDL cholesterol for participants aged >70 to ≤75 and >75 years respectively. This compared to a risk reduction of 25% for participants ≤55 years. [17] The risk of all-cause death was also reduced, albeit to a much lesser extent (9% per 1 mmol/L reduction in LDL cholesterol). No effect on non-vascular deaths was observed. Of note, participants were predominantly high risk, middle-aged men with high baseline LDL cholesterol levels (mean weighted LDL cholesterol level 3.7 mmol/L) or known cardiovascular disease.[17]

In those with cardiovascular disease (secondary prevention), the benefits of statin were clear with risks of major vascular events and all-cause death reduced in people of all ages.[18] Moreover, the number needed to treat was sufficiently low to justify treatment (28 patients would need to be treated for 5 years to prevent one death).[19]

In analyses limited to primary prevention populations, there was a significant trend toward smaller proportional risk reductions as age increased. (Figure 1) Analyses by age group indicated risk reductions of major vascular events of 16% and 8% per 1 mmol/L reduction in LDL cholesterol for participants aged >70 to ≤75 and >75 years respectively. This compared to a risk reduction of 32% for participants ≤55 years.[17] No data was presented on the risk of all-cause death in older people without cardiovascular disease.

For peer review

**Figure 1: Effects of statin therapy on major vascular events (MVE) per mmol/L reduction in LDL cholesterol by age at randomisation in participants without vascular disease.** [17]

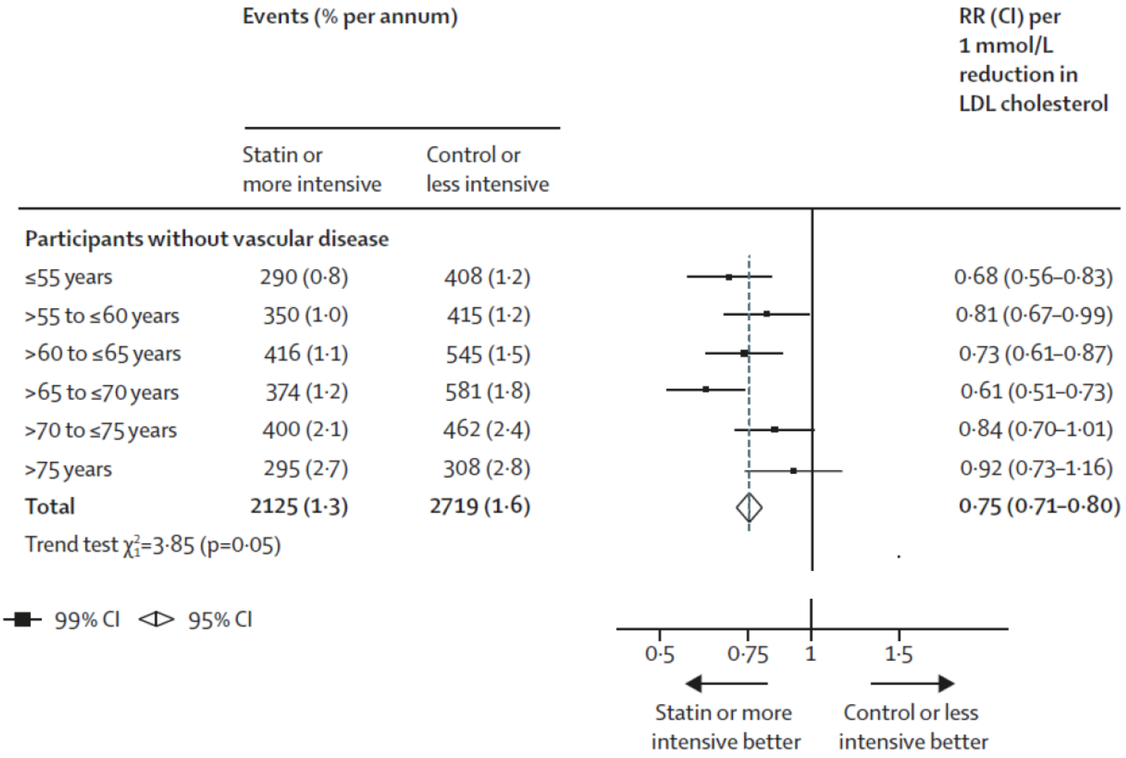


## Post hoc subgroup analyses of older participants in statin trials

Pravastatin in the PROspective Study of Pravastatin in the Elderly at Risk (PROSPER) trial, which followed 3,239 people aged 70 years or older for on average 3.2 years, did not


For peer review only - http://bmjopen.bmj.com/site/about/guidelines.xhtml

significantly reduce cardiovascular events.[20] Pravastatin in the Antihypertensive and Lipid-Lowering Treatment to Prevent Heart Attack Trial (ALLHAT-LLT) trial which followed 2,867 people 65 years or older for over 4.5 years, did not significantly reduce cardiovascular events or mortality.[21] Rosuvastatin in the Justification for the Use of Statins in Prevention: an Intervention Trial Evaluating Rosuvastatin (JUPITER) trial, which followed 5695 people aged 70 years or older for on average 2 years, significantly reduced cardiovascular events (RRR 39% 95%CI 14 to 57%) but not mortality.[22] Rosuvastatin in the Heart Outcomes Prevention Evaluation (HOPE 3) Trial, which followed 3086 people aged 70 years or older for on average 4.6 years, did not significantly reduce cardiovascular events.[22] A pooling of data from the last 2 trials (JUPITER and HOPE 3) concluded that statins reduced cardiovascular events in those people aged 65-70 years (RRR 49% 95%CI 31 to 62%) and >70 years (RRR 26% 95%CI 9 to 39%).[23]

This demonstrates the degree of uncertainty about the effects of statins in the older age groups. Given the potential for widespread use of statins amongst older people, the limited data emphasises the need for more certainty about the scale of any likely benefit of this class of drugs.

### 2.3 Other health outcomes

Physical and cognitive function, independence, and health-related quality of life are important health outcomes for older people. These outcomes have not been comprehensively evaluated in previous statin trials.

Statins have been proposed to attenuate long term decline in physical and cognitive function and to preserve independent living by reducing the morbidity associated with coronary and cerebrovascular disease (including subclinical disease) in the elderly.[3]

### 2.4 Statins in the Elderly: potential risks

The likelihood of adverse effects of drug therapy increases in older people.[24] Much of the knowledge of specific adverse effects comes from spontaneous reports or from trials predominantly studying middle aged people. As with efficacy, the burden of statin adverse effects in older individuals has not been well studied. As a result, it is still not possible to know the balance of risks and benefits associated with the use of these drugs in this age-group.[25]

Amongst the elderly, the issues most likely to influence the risk/benefit balance are:

#### 2.4.1 Myopathy

All statins have been reported to cause myopathy with the severity ranging from asymptomatic increases in creatinine kinase to muscle aches and weakness to fatal rhabdomyolysis.[26] The precise mechanisms are not well-understood. Of note, these myopathies are dose dependent and may occur even after therapy has been tolerated for up to 1 year.[27] The estimated incidence of statin myopathy in trials has ranged from 1.4 to 5% with the lowest rates reported for atorvastatin at low or high dosage.[27,28] Older age, female gender and concomitant use of inhibitors of cytochrome P450 3A4 are recognised risk factors for the development of statin myopathy.[29] There may also be a higher risk of disability in older adults using statins due to age-related sarcopaenia.[30]


For peer review only - http://bmjopen.bmj.com/site/about/guidelines.xhtml
For peer review only

### 2.4.2 Diabetes

In a recently published meta-analysis of statin trials, statin use was associated with a 9% increased risk for incident diabetes with little heterogeneity between trials.[31] Meta-regression indicated that the risk of diabetes was highest in trials of older participants.[31] In light of this data, in 2012 the FDA modified the labelling of statins to include a warning for the potential risk of increased blood glucose levels and new type 2 diabetes.[32]

### 2.4.3 Cancer

One trial (PROSPER) reported an increased risk of cancer and cancer mortality with statin therapy among men and women older than 70 years.[20] However, this effect was not observed in other statin trials. A meta-analysis of all statin trials suggested no overall increased risk of cancer or cancer mortality over an average follow up of 4.8 years, albeit in a mostly middle-aged population.[33] Nonetheless, more data on the effects of statin therapy on risk of cancer in elderly patients is required and further follow up studies have been called for in such patients.[25] Longer follow up would determine whether new cancer events could occur with time. This is especially important if statin therapy is to be advocated for primary prevention.

### 2.4.4 Cognitive impairment

The FDA recently reported a review of the US spontaneous reporting database (AERS), the published medical literature (case reports and observational studies), and randomised clinical trials to evaluate the effect of statin therapy on cognition.[32] The post-marketing adverse event reports generally described ill-defined memory loss that was reversible upon discontinuation of statin therapy. Most reports were from individuals over the age of 50 years. Time to onset of the impairment was highly variable, ranging from one day to years after statin exposure. The review did not find an association between cognitive impairment and any specific statin, any specific age group, any particular statin dose or concomitant medication use. Furthermore, the cognitive impairment did not appear to be associated with fixed or progressive dementia, such as Alzheimer's disease. Nonetheless, in 2012 the FDA modified the labelling of statins to include a warning for the potential risk of cognitive effects such as memory loss and confusion.[32] A subsequent meta-analysis of data from 25 randomised trials reported no statistically significant effect of statins on a crude overall measure of cognition across all cognitive domains.[34] This analysis included 12 trials undertaken exclusively in younger populations (< 65 years) where subtle effects may not be measurable and only 4 trials that had more than 1-year follow up. Indeed, this meta-analysis could not address either long-term safety or efficacy particularly in older populations, largely because few such trials exist.

### 2.4.5 Liver Injury

Statins have been associated with mild elevations in liver enzymes in approximately 0.5-2.0% of people, particularly within the first 12 weeks of therapy being initiated.[35] Meta-analysis of trials has demonstrated a 50% increased risk of elevations in liver transaminase compared to placebo or control.[36] The response appeared dose dependent. However, the elevations were usually transient, rarely associated with symptoms and deemed unlikely to be clinically relevant.


For peer review only - http://bmjopen.bmj.com/site/about/guidelines.xhtml

Serious statin induced liver injury was rare. Post marketing reviews of statins conducted by the FDA between 2000 and 2009 indicated fewer than 2 cases of statin associated serious liver injury per one-million patient years reported to the Adverse Event Reporting System.[37] Furthermore, although the use of statins has been rising over this period, no detectable increase in the annual rates of fatal or severe liver injury cases possibly or probably causally associated with statin use has been observed.[37]
Whether the risk of statin induced liver damage is increased in the elderly compared to younger people is currently unknown.

## 2.5 Utilisation

Statins are one of the most frequently dispensed drugs.[38] In the financial year ending 30 June 2012, over 10 million prescriptions for atorvastatin and 6 million prescriptions for rosuvastatin were dispensed in Australia.[39] In the period from April 2005 to March 2010, 86% of statin initiations came from general practitioners (GPs).[40] In 2011, the British Heart Foundation reported that 1 million statin prescriptions were provided each week in the United Kingdom.[41]

At present statin use in the elderly is lower than expected given the high absolute risk of this population. A UK survey of 41,250 GP medical records, reported that 23% of patients with no history of cardiovascular disease and aged over 75 were prescribed statin therapy compared to 29% in the 70-74 age group.[42] Amongst patients ≥75 years, for every 5 year increment in age the odds of being prescribed a statin decreased compared to the 40-44 year age group. One US study reported that 22% of people aged 80 years or older (in a primary prevention setting) were prescribed a statin[43] and another that those aged ≥70 years were less likely to use a statin than those aged 65-69 years (OR 0.57, 95% CI 0.39-1.09).[44] In an Australian survey of people aged 50 years and older, approximately 40% of respondents aged ≥75 years reported taking a statin.[45]

## 2.6 Implications of Guidelines on Treatment of Cholesterol in the Elderly

The potential for greater use of statin therapy amongst older people is illustrated by Figure 2 a) and b). This figure provides an approximation of the proportion of each age group where the estimated absolute cardiovascular risk exceeds a 15% likelihood of a new vascular event in the next five years (commonly regarded as the fundamental indication for statin therapy).[46] It is based on the cardiovascular death rate multiplied by four; in the WOSCOPS trial there were four incident cardiovascular events for each fatal event.[47] On this basis, most males over the age of 75 years and a high percentage of females would qualify for consideration of statin therapy.

Greater use of statins is also likely to be driven by the recent changes in guidelines on the treatment of cholesterol to reduce vascular events, which have caused controversy by now recommending moderate-to-high intensity statin therapy for people aged 40-75 years at much lower absolute risk (i.e. 10 year absolute cardiovascular risk of 7·5% or higher compared to 20% or higher in previous guidelines), with no requirement for monitoring of lipid levels.[48] In fact, guidelines that have moved from target-based to absolute cardiovascular risk based treatment strategies, controversially risk medicalisation of ‘healthy older populations’ as all elderly individuals (>70 years) have an estimated absolute cardiovascular risk greater than 10%.


For peer review only - http://bmjopen.bmj.com/site/about/guidelines.xhtml

Figure 2: The proportion of each age group in each absolute cardiovascular risk stratum for a) males and b) females. The shaded section under the thick black line shows the approximate percentage of each age group with a risk of 15% of developing a cardiovascular event in the next 5 years. The different shades of blue (from lighter to darker) show the proportions with a 5%, 10%, 15% and 20% risk.

a) Males

| Percentile of 10-year CHD mortality risk | Age | | | | | | | | | | | | | | |
|---|---|---|---|---|---|---|---|---|---|---|---|---|---|---|---|
| | 25-29 | 30-34 | 35-39 | 40-44 | 45-49 | 50-54 | 55-59 | 60-64 | 65-69 | 70-74 | 75-79 | 80-84 | 85+ | SUM | % of all CHD deaths |
| 2-5 | 0 | 0 | 1 | 2 | 4 | 7 | 9 | 13 | 21 | 38 | 54 | 54 | 102 | 305 | 2 |
| 6-10 | 0 | 0 | 1 | 2 | 5 | 9 | 12 | 16 | 25 | 44 | 61 | 64 | 109 | 348 | 3 |
| 11-15 | 0 | 0 | 1 | 3 | 6 | 10 | 14 | 18 | 29 | 50 | 69 | 73 | 116 | 389 | 3 |
| 16-20 | 0 | 1 | 2 | 3 | 7 | 12 | 16 | 21 | 32 | 54 | 76 | 83 | 123 | 430 | 3 |
| 21-25 | 0 | 1 | 2 | 4 | 7 | 13 | 18 | 23 | 36 | 57 | 82 | 91 | 128 | 462 | 3 |
| 26-30 | 0 | 1 | 2 | 4 | 8 | 14 | 19 | 25 | 39 | 59 | 88 | 98 | 133 | 490 | 4 |
| 31-35 | 0 | 1 | 2 | 5 | 9 | 15 | 21 | 27 | 41 | 62 | 93 | 104 | 140 | 520 | 4 |
| 36-40 | 0 | 1 | 3 | 5 | 10 | 16 | 22 | 30 | 44 | 65 | 97 | 109 | 147 | 549 | 4 |
| 41-45 | 0 | 1 | 3 | 6 | 11 | 17 | 24 | 32 | 45 | 68 | 101 | 114 | 154 | 576 | 4 |
| 46-50 | 0 | 1 | 3 | 6 | 11 | 18 | 25 | 34 | 48 | 71 | 105 | 119 | 160 | 601 | 5 |
| 41-55 | 0 | 1 | 3 | 7 | 12 | 19 | 27 | 37 | 51 | 75 | 110 | 124 | 166 | 632 | 5 |
| 56-60 | 1 | 2 | 4 | 7 | 13 | 21 | 29 | 40 | 55 | 79 | 115 | 129 | 171 | 666 | 5 |
| 61-65 | 1 | 2 | 4 | 8 | 14 | 22 | 31 | 43 | 58 | 84 | 120 | 133 | 176 | 696 | 5 |
| 66-70 | 1 | 2 | 4 | 9 | 16 | 24 | 34 | 47 | 63 | 88 | 126 | 139 | 182 | 735 | 6 |
| 71-75 | 1 | 2 | 5 | 10 | 18 | 26 | 37 | 51 | 67 | 94 | 133 | 145 | 189 | 778 | 6 |
| 76-80 | 1 | 3 | 5 | 11 | 20 | 29 | 42 | 56 | 73 | 102 | 141 | 153 | 197 | 833 | 6 |
| 81-85 | 1 | 3 | 6 | 13 | 23 | 33 | 48 | 62 | 79 | 111 | 150 | 162 | 210 | 901 | 7 |
| 86-90 | 2 | 4 | 8 | 16 | 28 | 39 | 57 | 72 | 89 | 124 | 161 | 174 | 236 | 1010 | 8 |
| 91-95 | 2 | 5 | 11 | 19 | 33 | 45 | 66 | 82 | 99 | 141 | 173 | 187 | 263 | 1126 | 8 |
| 96-100 | 2 | 6 | 14 | 22 | 39 | 52 | 75 | 92 | 109 | 158 | 185 | 199 | 290 | 1243 | 9 |
| SUM | 12 | 37 | 84 | 162 | 294 | 441 | 626 | 821 | 1103 | 1624 | 2240 | 2454 | 3392 | 13290 | |
| % of all CHD deaths | 0 | 0 | 1 | 1 | 2 | 3 | 5 | 6 | 8 | 12 | 17 | 18 | 26 | | |

b) Females

| Percentile of 10-year CHD mortality risk | Age | | | | | | | | | | | | | | |
|---|---|---|---|---|---|---|---|---|---|---|---|---|---|---|---|
| | 25-29 | 30-34 | 35-39 | 40-44 | 45-49 | 50-54 | 55-59 | 60-64 | 65-69 | 70-74 | 75-79 | 80-84 | 85+ | SUM | % of all CHD deaths |
| 2-5 | 0 | 0 | 0 | 0 | 1 | 1 | 2 | 3 | 6 | 13 | 24 | 46 | 123 | 219 | 2 |
| 6-10 | 0 | 0 | 0 | 0 | 1 | 1 | 2 | 4 | 8 | 15 | 28 | 53 | 150 | 262 | 2 |
| 11-15 | 0 | 0 | 0 | 1 | 1 | 1 | 3 | 5 | 10 | 17 | 31 | 61 | 176 | 306 | 3 |
| 16-20 | 0 | 0 | 0 | 1 | 1 | 2 | 3 | 5 | 11 | 20 | 35 | 69 | 202 | 349 | 3 |
| 21-25 | 0 | 0 | 0 | 1 | 1 | 2 | 3 | 6 | 12 | 22 | 38 | 75 | 223 | 383 | 3 |
| 26-30 | 0 | 0 | 0 | 1 | 1 | 2 | 4 | 7 | 13 | 24 | 42 | 82 | 239 | 415 | 4 |
| 31-35 | 0 | 0 | 0 | 1 | 1 | 2 | 4 | 8 | 14 | 26 | 45 | 87 | 254 | 442 | 4 |
| 36-40 | 0 | 0 | 0 | 1 | 2 | 3 | 4 | 8 | 15 | 28 | 47 | 91 | 268 | 467 | 4 |
| 41-45 | 0 | 0 | 0 | 1 | 2 | 3 | 5 | 9 | 16 | 31 | 51 | 95 | 283 | 496 | 4 |
| 46-50 | 0 | 0 | 0 | 1 | 2 | 3 | 5 | 9 | 16 | 33 | 55 | 100 | 301 | 525 | 4 |
| 41-55 | 0 | 0 | 1 | 1 | 2 | 3 | 6 | 9 | 17 | 36 | 60 | 105 | 323 | 563 | 5 |
| 56-60 | 0 | 0 | 1 | 1 | 3 | 4 | 6 | 10 | 18 | 37 | 67 | 112 | 346 | 605 | 5 |
| 61-65 | 0 | 0 | 1 | 2 | 3 | 4 | 7 | 10 | 19 | 39 | 75 | 120 | 367 | 647 | 6 |
| 66-70 | 0 | 0 | 1 | 2 | 3 | 5 | 7 | 11 | 21 | 41 | 84 | 129 | 382 | 686 | 6 |
| 71-75 | 0 | 1 | 1 | 2 | 4 | 5 | 8 | 12 | 23 | 44 | 93 | 140 | 391 | 724 | 6 |
| 76-80 | 0 | 1 | 1 | 2 | 4 | 6 | 9 | 14 | 25 | 49 | 103 | 152 | 397 | 763 | 6 |
| 81-85 | 0 | 1 | 1 | 3 | 5 | 7 | 11 | 17 | 27 | 55 | 115 | 167 | 406 | 815 | 7 |
| 86-90 | 0 | 1 | 2 | 4 | 6 | 10 | 14 | 20 | 33 | 65 | 134 | 194 | 430 | 913 | 8 |
| 91-95 | 1 | 1 | 3 | 5 | 8 | 12 | 19 | 26 | 41 | 79 | 153 | 221 | 455 | 1024 | 9 |
| 96-100 | 1 | 1 | 3 | 6 | 10 | 14 | 24 | 32 | 51 | 93 | 172 | 249 | 482 | 1138 | 10 |
| SUM | 2 | 6 | 15 | 36 | 61 | 90 | 146 | 225 | 396 | 767 | 1452 | 2348 | 6198 | 11742 | |
| % of all CHD deaths | 0 | 0 | 0 | 0 | 1 | 1 | 1 | 2 | 3 | 7 | 12 | 20 | 53 | | |



For peer review only - http://bmjopen.bmj.com/site/about/guidelines.xhtml

## 2.7 Summary of rationale

- Meta-analysis of trials highlights the uncertainty about the efficacy of statins in the prevention of morbidity and mortality in older populations (over 70 years). This may stem from inadequate numbers in this age group included in major trials.
- Warnings about adverse effects of statins have recently been added to the product information but their frequency is uncertain and appears higher in the elderly.
- A strong potential exists for greater promotion of statins amongst the over 70 age group on the basis of recent guidelines advocating use for primary prevention at lower absolute cardiovascular risk levels. Evidence in the elderly is currently insufficient to support this proposal.
- Real world trials examining the impact of statins on a range of clinical outcomes important to older people have not been conducted.

For peer review only


For peer review only - http://bmjopen.bmj.com/site/about/guidelines.xhtml

# 3. Study Objectives

## 3.1 Primary objectives

To determine in people aged ≥70 years the effect of statin therapy (40 mg atorvastatin) versus placebo, over an average 6-year treatment period, on two co-primary clinical endpoints:

(i) a composite of all cause death or dementia or development of disability and;
(ii) a composite of cardiovascular death, non-fatal myocardial infarction or non-fatal stroke.

## 3.2 Secondary objectives

(1) To determine the effects of statin therapy versus placebo in people aged ≥70 years on each of: all cause death, cardiovascular death, dementia, other cognitive impairment, disability, all cause hospitalisation, need for permanent residential care, fatal and non-fatal myocardial infarction, fatal and non-fatal stroke (thromboembolic and haemorrhagic), fatal and non-fatal cancer, atrial fibrillation, heart failure and quality of life.

(2) To assess the cost-effectiveness of statin therapy for primary prevention in people aged ≥70 years.

## 3.3 Tertiary objectives

These include effects of statin therapy on pathology measures such as fasting glucose/HbA1c, urine ACR and estimated glomerular filtration rate (eGFR) as well as frailty phenotype and functional independence.


For peer review only - http://bmjopen.bmj.com/site/about/guidelines.xhtml

# 4. Hypotheses

## 4.1 Primary null hypotheses

Treatment with statin (atorvastatin 40 mg) compared with placebo will not prolong overall disability free survival amongst a healthy elderly population (≥70 years).

Treatment with statin (atorvastatin 40 mg) compared with placebo will not reduce the incidence of major adverse cardiovascular events amongst a healthy elderly population (≥70 years).

## 4.2 Primary alternative hypotheses

Treatment with statin (atorvastatin 40 mg) will prolong overall disability free survival amongst a healthy elderly population (≥70 years).

Treatment with statin (atorvastatin 40 mg) will reduce the incidence of major adverse cardiovascular events amongst a healthy elderly population (≥70 years).

For peer review only


For peer review only - http://bmjopen.bmj.com/site/about/guidelines.xhtml

# 5. Study Design

## 5.1 Trial Design

STAREE is a double blind, randomised placebo-controlled parallel group trial that is investigating whether statin therapy can prolong good health and maintain independence amongst older people.

## 5.2 Study Population

### 5.2.1 General considerations

The study will enrol men and women 70 years of age and over who are free from cardiovascular disease, diabetes and dementia and living independently in the community.

The study sites are listed in Appendix 1.

### 5.2.2 Inclusion criteria

Potential participants must meet the following criteria to be included in the study:

- Men and women aged ≥70 years living independently in the community,
- Willing and able to provide informed consent and accept the study requirements (Note: competent physical ability to participate in the trial is assessed using the Life Ability ADL questionnaire)

### 5.2.3 Exclusion criteria

Potential participants who meet any of the following criteria will be excluded from the study:

- A history of clinical cardiovascular disease (defined as myocardial infarction, angina, coronary artery angioplasty and/or stenting, coronary artery bypass grafting surgery, heart failure, stroke, transient ischaemic attack (TIA), carotid stenosis, abdominal aortic aneurysm and peripheral vascular disease),
- Clinical diagnosis of dementia, treatment with medications for dementia (e.g. donepezil (Aricept), rivastigmine (Exelon) or Galantamine (Galantyl) or a 3MS score <78 on screening.
- A history of diabetes or evidence of diabetes on pathology results (WHO diagnostic criteria whereby HbA1c ≥ 6.5%/ 48 mmol/mol or fasting glucose ≥7.0 mmol/L on two consecutive tests),
- Total cholesterol >7.5 mmol/L, [49]
- Moderate or severe chronic kidney disease (persistent proteinuria whereby urine albumin: creatinine ratio >30 mg/mmol [50] and/or eGFR <45 ml/min/1.73m$^2$ on two consecutive tests), [49]
- Moderate or severe liver disease (persistent elevations of transaminases of more than 3 times the upper limit of the normal laboratory reference range),
- Serious inter-current illness likely to cause death within the next 5 years such as terminal cancer or obstructive airways disease,
- Current participation in another interventional clinical trial,
- Absolute contraindication to statin therapy,
- Current use of statin therapy or other lipid lowering therapy for primary prevention and unwilling to stop therapy,


For peer review only - http://bmjopen.bmj.com/site/about/guidelines.xhtml

- Current long term or permanent use of potent cytochrome P450 (CYP) 3A4 inhibitors as specified in Appendix 3.

### 5.2.4 Rescreening or reassessment

Potential participants who are excluded on the basis of either their 3MS total score (<78) or their responses on the Life Ability questionnaire (unable to perform items 9-14) may be re-screened if the result is likely to be related to a temporary issue that may resolve over time (e.g. temporary depressive symptoms as indicated by the CES-D 10 or temporary disability related to fracture).

To be eligible for re-screening, participants with a 3MS score below 78 that also score 8 or above on the CES-D 10 (indicating presence of depressive symptoms) may be re-screened in 3 months. There may be instances where rescreening or reassessment of certain tasks may be required, such as participants with a temporary disability, and these participants will be considered for such on a case-by-case basis.

## 5.3 Study Intervention and Choice of Comparator

### 5.3.1 Intervention

Two tablets of Atorvastatin (2 x 20 mg/day) or identical placebo.

### 5.3.2 Rationale for choice of atorvastatin

Atorvastatin is the most commonly prescribed statin in Australia.

Atorvastatin is well researched with wide practice experience. Importantly, relative to other statins, it has a favourable side effect profile and has been shown to be one of the most potent statins for reducing low density lipoprotein concentrations (LDL-C) and triglyceride levels. The dose of 40 mg is expected to produce a 30-50% reduction in LDL-C (see table below).

For peer review only


For peer review only - http://bmjopen.bmj.com/site/about/guidelines.xhtml

Table 1: Average relative reduction in LDL cholesterol concentrations with different doses of commonly used statins. (51)

| | Daily dose of different statins | | | | |
|---|---|---|---|---|---|
| | 5 mg | 10 mg | 20 mg | 40 mg | 80 mg |
| Pravastatin | 15% | 20% | 24% | 29% | 33% |
| Simvastatin | 23% | 27% | 32% | 37% | 42% |
| Atorvastatin | 31% | 37% | 43% | 49% | 55% |
| Rosuvastatin | 38% | 43% | 48% | 53% | 58% |

Shaded boxes indicate regimens that can produce about a halving or more in LDL cholesterol concentrations (largely irrespective of patient characteristics, including presenting concentrations of cholesterol). The 2016 cost for generic atorvastatin 40 mg daily in the UK is about £2 per 28 days of treatment;[184] rosuvastatin 20 mg daily currently costs about £25 per month,[185] but it became available as a generic in the USA during 2016.

Different statins have different potencies and newer therapies such as atorvastatin and rosuvastatin produce larger reductions in LDL-C per mg of drug than older therapies, such as simvastatin and pravastatin.[51] Adverse events associated with statin treatment may be more likely with higher doses of specific statins (i.e. simvastatin ≥ 80 mg) and with combination therapy (i.e. statin and fibrate). [52, 53] However, the flexibility of reducing from 40 mg (standard dose) to 20mg (low dose) will assist with exploring dose-response medication-related side effects and up or down titration will assist with reducing study medication discontinuation rates.

A very important issue that has not been addressed is the balance between expected benefits and harms of statin therapy in people aged over 70 years. STAREE will provide evidence to address this.

## 5.4 Participant recruitment

### 5.4.1 Recruitment

First participant expected to be consented to the study in October 2015 and the first participant expected to be randomised to study medication in November 2015.

### 5.4.2 Setting

STAREE will be conducted in Australian primary care (GP) practices and the community.

Registered GP co-investigators will access their computerised practice databases, in association with STAREE GP Liaison Officers if requested, to identify all patients aged 70 years and over. Depending on the practice database, patients will be further screened for inclusion and exclusion criteria. Those who are physically able to attend the practice and considered suitable for the study by their GP, will be invited to participate in the study via a letter of invitation.


For peer review only - http://bmjopen.bmj.com/site/about/guidelines.xhtml

### 5.4.3 Community participants

Any participants whose usual GPs are not registered as STAREE co-investigators will be considered as community participants. They will be assessed by a STAREE medical officer for suitability to be in the study. Their usual GP will be notified of their involvement and will be provided a copy of any pathology and any abnormal assessments that are performed as part of the study. Baseline and annual visits for community participants will be conducted at community venues or in the STAREE site offices.

### 5.4.4 STAREE hub locations

The STAREE central coordinating site is located in Melbourne, Australia.

STAREE hubs will be located in regional Victoria, Queensland, Tasmania, Western Australia, South Australia and New South Wales (see Appendix 1). Hubs will service the immediate and surrounding areas and may assist with other locations when requested.

## 5.5 Participant timeline and schedule of assessments

Figure 3 shows the participant timeline including the baseline screening phase, randomisation and follow up phase.

Table 2 shows a summary of the schedule of assessments to be undertaken at each study visit. Additional details of the cognitive assessments, physical measures and laboratory tests are provided in Appendix 6.

## 5.6 Baseline Screening Phase

The baseline screening phase will comprise a screening phone call, baseline visit 1, medication run in, GP visit and baseline visit 2.

Eligibility for randomisation into the study will be assessed across the baseline screening and medication run-in phase and will be based on the inclusion and exclusion criteria as well as compliance with the run-in medication.

### 5.6.1 Screening call

Potential participants will phone the study centre after they have received an invitation to participate in the study.  At this phone screening call, they will be screened for eligibility to be included in the study. Those who pass this first screening stage will then be invited to attend baseline visit 1.


For peer review only - http://bmjopen.bmj.com/site/about/guidelines.xhtml

Figure 3: Participant Timeline

**Call**

**Baseline Visit 1 (Face to face or phone visit)**

- Informed consent obtained
- Questionnaires, exclusion criteria and pathology requested

**4 week run-in**

- Participant to take a 4-week run-in of 1 tablet of placebo
- Participant to see GP for inclusion in study

**Baseline Visit 2 (Face to face or phone visit)**

- Questionnaires are administered
- Review inclusion/exclusion criteria

**Randomisation**

- Participants randomised to placebo or active statin
- Participants to receive their study medication via tracked post (annual send outs)

**4 week post randomisation**

- Participants to take 1 tablet / 20 mg of study medication

**Call 4 weeks after randomisation**

- Participants to increase dosage to 2 tablets / 40 mg of study medication

**Participant contact post-randomisation**

- Participants will have a study follow up visit every 6 months (these study visits will alternate as phone visits or clinic/phone visit).
- Participants will have a final close-out study visit (clinic/phone visit)


For peer review only - http://bmjopen.bmj.com/site/about/guidelines.xhtml

**Table 2: STAREE measurement and study activity schedule**. A single X (with no superscripts) indicates that all specified measures carried out. The presence of a superscript indicates that the corresponding specific assessment is performed at that time point.

| | Screening | Baseline visits (BV) | | | Post-randomisation | | | |
|---|---|---|---|---|---|---|---|---|
| Measurement/ Activity | Screening call | BV 1 | GP visit | BV 2 | 1 month call | Annual visits (without cognitive testing) | Annual visits (with cognitive testing) | Close out visit |
| Type of visit | phone | clinic or phone | clinic or telehealth | clinic or phone | phone | clinic or phone | clinic or phone | clinic or phone |
| Month in trial (week) | 0 | 0 | 0 | 0 | (4) | | | |
| Review inclusion/ Exclusion criteria | X | X | X | X | | | | |
| Obtain informed consent | | X | | | | | | |
| Dispense study medication (supply posted after annual study visit once compliance checked) | | X (run-in med only) | | Posted (after randomis ation) | | | | |
| Medication titration | | | | | X | | | |
| Assess medication compliance | | | | X | | X | X | X |
| Concomitant medications | | X | | | | X | X | X |
| Blood pressure & heart rate/rhythm[a], height[b], weight[c], waist:hip ratio[c], | | X[a,b,c] | | | | X [a, c,] | X [a, c,] | X |
| Lifestyle profile & personal medical history[d], family medical history[e] & IPAQ-E[f], physical function | | X[d, e] | | X | | X[d, f] | X[d] | X[d] |
| Routine Laboratory testing, GP referral to local pathology Bloods**: fasting glucose[1], fasting lipids (TC, HDL, LDL, triglyceride)[1], Hb[2], CK[2], creatinine[3], (eGFR)[3], ALT[3], AST[3], HbA1c[3], Urine: ACR[3] | | X[1,2,3] | | | | X[3] | X[3] | X[1,3] |
| Measurement/ Activity | Screening call | BV 1 | GP visit | BV 2 | Post-randomis ation | | | |

For peer review only



For peer review only - http://bmjopen.bmj.com/site/about/guidelines.xhtml

| | Screening | Baseline visits (BV) | | | Post-randomisation | | | |
|---|---|---|---|---|---|---|---|---|
| | Screening call | BV 1 | GP visit | BV 2 | 1 month call | Annual visits (without cognitive testing) | Annual visits (with cognitive testing) | Close out visit |
| Type of visit | phone | clinic or phone | clinic | clinic or phone | phone | clinic or phone | clinic or phone | |
| Month in trial (week) | 0 | 0 | 0 | 0 | (4) | | | |
| **Quality of life**<br>SF-36 | | | | X | | X | X | X |
| **Assess cognitive function**<br>3MS± (and CES-D 10 to exclude depression); CAM[1] | | X | | | | | X | X |
| **Detailed cognitive tests**[β£]<br>COWAT, Stroop, HVLT-R, SDMT, CERAD Constructional Praxis, Lurian Overlapping Figures, Trail Making Test | | | | X | | | X | X |
| **Assess physical disability**<br>Life disability ADL | | X | | | | X | X | X |
| **Biobank samples collected** | | | | X | | | | |
| **Clinical event reporting**<br>Questionnaire & medical report review | | | | | | X | X | X |

**All study visits, including baseline and follow up visits, may be either conducted in person or by phone call. For visits conducted by phone call, physical measures (blood pressure, heart rate and weight) will be collected from medical records if available.

± Full 3MS is conducted at clinic visits and a partial 3MS is conducted at phone visits

β Annual visits with cognitive testing conducted by phone will not include SDMT, STROOP, CERAD Constructional Praxis subscale and Lurian Overlapping Figures. These tests will only be conducted at in person visits.

£B-ADL (Bayer Activities of Daily Living) is administered to the named 'study partner' of any participant who shows a pre-specified decline in cognitive tests at an annual visit. If the participant does not consent to a study partner undertaking the B-ADL, then it may be administered to the participant themselves.


For peer review only - http://bmjopen.bmj.com/site/about/guidelines.xhtml
For peer review only

### 5.6.2 Lifestyle profile and screening visits (Baseline visits)

STAREE staff will explain the study to participants. After informed consent is provided measurement and medical history data will be collected to further assess eligibility for the study. Participants will be given a copy of their consent form. Participants will be assigned a unique identification number, which will be used throughout the study.

The information to be collected and reviewed at the baseline visits will include the following:

a. Basic demographic and lifestyle factors
b. Past medical history – identified by the participant and GP practice medical record or patient health summary (as available)
c. Cognitive assessment – 3MS[54] (full or modified version if phone visit)
d. Depressive symptoms assessment – CES-D 10(55)
e. Physical disability – ADL using Life Ability questionnaire[56]
f. Blood pressure, heart rate and rhythm, weight, height and waist circumference
g. Concomitant medications
h. Pathology – referred to local pathology collection service for blood and urine tests

Note: Pathology testing results from participants' medical records may be used to assess eligibility if performed no more than 6 months before Baseline visit 1. HbA1c may be used if performed within the past 12 months.

Medication for the run-in phase will be handed out.

### 5.6.3 Post screening GP/medical visit (Run-in phase between Baseline visits 1 and 2)

Participants will see their GP who will be an enrolled co-investigator (or the STAREE medical officer if the usual GP is not an enrolled co-investigator) and will complete an Inclusion/Exclusion Criteria Worksheet. The baseline pathology testing results will be reviewed by the GP (or the STAREE medical officer). These results will help determine eligibility via the inclusion and exclusion criteria.

### 5.6.4 Post screening visit study activity (Run-in phase between Baseline visits 1 and 2)

Participants will enter a run-in phase of 4 weeks whereby they will be asked to take one tablet of study medication (placebo) a day.

### 5.6.5 Determinations of Eligibility and Assessments visit (Baseline Visit 2)

Participants will have a second visit with STAREE field officers after the run-in phase. This visit will be conducted within 4 to 6 weeks of the baseline visit 1.

Participants will be deemed eligible to participate if they are compliant with run-in medication pill count (>85% taken), have satisfactory pathology testing and their GP (or the STAREE medical officer) has provided approval using the GP screening form.

If deemed eligible to continue to baseline visit 2, participants will undergo the following assessments at this second screening visit:

a. Quality of life – Short Form (SF)--36 Questionnaire.(57)


For peer review only - http://bmjopen.bmj.com/site/about/guidelines.xhtml

b. Comprehensive assessment of cognition – COWAT[58], STROOP[59], HVLT-R[60], SDMT[61], CERAD Constructional Praxis subscale[62], Lurian Overlapping Figures[63], Trail Making Test[64] (if funding available).

Participants' willingness to donate and have blood samples collected and stored in the STAREE biobank will be ascertained at the baseline visit 2 and offered at STAREE hubs with facilities for sample processing and storage.

The purpose of this sample collection is to allow identification and analysis of potential biomarkers that predict the onset of age-related diseases such as cardiovascular disease, dementia, cancer and diabetes.

### 5.7 Randomisation and follow up phase

The randomisation and follow up phases are illustrated in Figure 3.

#### 5.7.1 Randomisation procedure

Following the completion of data collection at baseline visit 2, the Randomisation Form will be completed, and participants will be randomised into the STAREE study via the study website.

An independent statistician will generate the allocation list, using the statistical package R. This arrangement will ensure that the randomisation code remains inaccessible to all study staff and investigators. The allocation list will be generated using Cox model randomly permuted blocks (of size either 2 or 4), with randomisation stratified for site and age group (<80y, 80+y). Blocks will ensure ongoing approximate balance of randomisation to statin and placebo within strata. Treatment allocation will be achieved through a web or interactive voice recognition telephone-based system.

Following the completion of the randomisation process the study medication will be posted to participants.

Participants' contact details will be confirmed to ensure the correct mailing address for study medication is recorded and the addresses of the closest relative, friend and local doctor provided.
Participants will be instructed to take one tablet of study medication a day.
Participants will be instructed to call the study centre if they have any adverse effects.

The distribution of study medication is detailed in Section 8.

A record of all participants undergoing screening and randomisation will be made in the appropriate tracking log.

#### 5.7.2 Post randomisation procedure

Participants will be contacted by phone one month after commencement of study medication to review tolerability and compliance with study medication and instruct them to increase the dose from one tablet to two tablets. The date of this phone contact will be taken from the drug receipt log as recorded in the STAREE database.



For peer review only - http://bmjopen.bmj.com/site/about/guidelines.xhtml

Participants reporting any tolerability issues with the study medication will be referred back to their GP for further advice and management.

Table 3. Study medication

| | 0 | 4 weeks |
|---|---|---|
| Number of tablets | 1 | 2 |
| | Receive study medication | Phone call to increase to 2 tablets |

### 5.7.3 Blinding

All STAREE staff including the investigator team, management, administration, medical officers, GP Liaison Officers, Field Officers, Data Officers and students will be blinded to treatment allocation through the randomisation procedure and study duration.
All GP co-investigators and practice staff will be blinded to treatment allocation.
All participants will be blinded to treatment allocation.

In the event of a clinical emergency, participants will be assumed to be on active treatment and the recommendation will be to cease treatment.

A process for emergency unblinding will be in place at Ramsay Healthcare. Selected Ramsay Healthcare staff will be granted access to the randomisation code. This information will be accessible and shared using a Secure File Transfer Portal (SFTP).

The need for unblinding of study participants will be determined on a case-by-case basis after referral to a senior STAREE medical officer.

### 5.7.4 Annual Follow up visit

Each year after randomisation, participants will participate in a study visit with a Field Officer. Visits may be conducted face-to-face or by phone. Annual measurements will include cognitive tests and disability assessments, lifestyle questionnaires as well as physical measures. The Quality of Life (SF-36), Life Ability and Annual medical history questionnaires will be provided in an online format or posted to participants who have agreed to this. For all other participants these surveys will be conducted at face-to-face visits. Pathology will be repeated (excluding lipids). Participants will be asked about their recent medical history and any concomitant medications they are taking. Additionally, participants will be asked about serious adverse events (SAEs) and potential study endpoints.

Following notification, clinical information relating to any SAE or endpoint will be sourced through: a) medical record review and b) hospital records and discharge summaries.

### 5.7.5 Phone Contact

After randomisation and the mail out of study medication, if receipt of study medication has not been registered a participant will be contacted by phone.


For peer review only - http://bmjopen.bmj.com/site/about/guidelines.xhtml

Annual phone calls (6, 18, 30, 42, 54, 66 months) will be made to ask participants about SAEs and potential study endpoints and to encourage compliance with study medication.

Data to be collected during the phone calls include:

- Compliance with study medication
- Serious adverse event and endpoint notification
- Concomitant medications
- Physical disability (Life disability ADL)

### 5.7.6 Other Actions

If a participant does not proceed to randomisation, then the reason for non-randomisation is to be documented and collected on all relevant hard copy and electronic (database) Case Report Forms (CRF) such as the Screening CRF and Assessment and Randomisation CRF Contact with the participant regarding the outcome of the screening phase will be recorded within the Participant Communication Log

### 5.8 Economic Analysis

In addition to the disability free survival and cardiovascular outcomes, STAREE will examine the cost effectiveness of statin treatment for prolonging disability free survival and preventing major adverse cardiovascular events in healthy individuals aged ≥70 years.

Decision analysis will be used to compare the downstream consequences of atorvastatin versus placebo. Incorporation of Markov and life-tabling techniques will allow for the modelling of outcomes beyond the 6-year duration of the trial.

The main output of interest in health economic modelling will be the incremental cost-effectiveness ratio in terms of net costs per unit of health gain. Net costs will comprise the costs of statin minus costs saved from the reduction in downstream health services utilisation. Health gains will be measured by years of life and quality-adjusted life years (QALYs) gained. Both will be enabled by the collection of time-to-outcome data and QALYS also by collection of quality of life data. Cost data will be estimated by applying published unit costs (from an Australian healthcare system perspective) to data on health services utilisation. All health economic analyses will be undertaken in accordance with recommended approaches, such as 5% discounting of estimated future costs and health gains. To account for any uncertainty in the data inputs for health economic modelling, sensitivity and uncertainty analyses will be undertaken via Monte Carlo simulation.

## 6. Study Endpoints

### 6.1 Primary endpoints

STAREE will have two co-primary endpoints, disability free-survival and major adverse cardiovascular events, which are both composite endpoints.

1. Disability-free survival - defined as survival free of dementia or persistent physical disability (as derived from the endpoints of all-cause mortality, dementia and physical disability), and


For peer review only - http://bmjopen.bmj.com/site/about/guidelines.xhtml

2. Major adverse cardiovascular events – defined as the first occurrence of a non-fatal myocardial infarction, non-fatal stroke or cardiovascular death.

### 6.2 Secondary endpoints

Two types of outcome measures are pre-specified secondary endpoints in STAREE. The first group are components of the co-primary endpoints and additional cognitive and cardiovascular endpoints (heart failure, other acute coronary syndromes and revascularisations). The second group include non-cardiovascular endpoints (need for permanent residential care, hospitalisation and cancer).

1. All cause death
2. Cardiovascular death
3. Fatal and non-fatal myocardial infarction
4. Fatal and non-fatal stroke
   a. Haemorrhagic stroke
   b. Thromboembolic stroke
5. Persistent physical disability (in ADL based on Life Ability questionnaire[56])
6. Dementia
7. Other cognitive impairment (not meeting criteria for Dementia)
8. Approved need for permanent residential care (based on reporting by an Aged Care Assessment Team)
9. All cause hospitalisation (reasons and length of stay)
10. Heart failure
11. Atrial fibrillation
12. Revascularisation procedure
13. Fatal and non-fatal cancer (excluding non-melanoma skin cancer)
14. Quality of life (SF-36 questionnaire[57])
15. Cost-effectiveness

### 6.3 Tertiary endpoints

Pre-specified tertiary endpoints are:

1. Fasting glucose/HbA1c
2. Urine ACR
3. Estimated glomerular filtration rate (eGFR)
4. Frailty
5. Loss of functional independence (instrumental ADLs)

Please refer to Appendix 2 for brief definitions of primary and secondary endpoints.

### 6.3 Endpoint ascertainment

At 6-month telephone calls and annual visits, participants will be asked whether they have been diagnosed with any new health events which are from a list of study endpoints. Notification of a potential study endpoint will trigger the collection of further information for endpoint confirmation and adjudication. Confirmation of endpoints will be ascertained by collecting information from any of the following sources within 90 days of learning of the event:


For peer review only - http://bmjopen.bmj.com/site/about/guidelines.xhtml

- Details from medical records from the usual treating physician or practice held medical record.
- Medical records obtained by letter/fax/email contact with other treating specialist physicians or secondary/tertiary medical care centres.
- Case Report Forms.
- Hospital records/discharge summaries, pathology reports.
- Death Certification: The National Death Index will be routinely sourced for notification of death not identified through the above processes for up to 5 years after the completion of study.
- Review of Commonwealth Department of Health and Ageing (DOHA) database of aged care assessments (ACAP dataset).
- Review of cancer registries for up to 5 years after the completion of study.
- Aged Care Assessment Service (ACAS report): A copy is provided to the GP and assesses the needs and level of care recommended for the individual.

### 6.4 Endpoint adjudication process

An end-point adjudication committee (EAC) will be appointed with the task of assessing the clinical data relating to all endpoints. Data collected from GPs, hospitals, specialists and other sources by the research staff will be presented to the EAC. The information available to the EAC will not only include the clinical source documentation obtained from the GP records but also death certificates and, if available, autopsy reports blinded to participant identity. The EAC will adjudicate through an online system with meetings conducted to resolve discordant adjudications and to develop decision rules.

## 7. Sub-studies and ancillary studies

### 7.1 STAREE-HEART

**STAREE-HEART will conduct a suite of additional cardiac assessments in a subset of up to 500 STAREE participants before they are randomised to study medication. It aims to determine the effect of statin treatment over a 3 year period compared with placebo on markers of cardiac ageing. These include an echocardiogram which measures global longitudinal strain (GLS), which may predict incident atrial fibrillation (AF) and heart failure, and twice daily handheld ECG recordings over a 2-week period which may improve AF detection 4-fold compared to usual screening. Additionally, a new energy waveform 12-lead ECG will be performed. The assessments will take place prior to randomisation and will be repeated at the year 3 follow up.**

### 7.2 STAREE-MIND

STAREE-MIND will conduct neuroimaging assessments in a subset of up to 300 STAREE participants. STAREE-MIND aims to determine the effect of statin treatment on longitudinal measures of brain health including grey matter volume, white matter integrity and white matter hyperintensities, cerebral perfusion and metabolism, and iron loading. It will also examine the relationship between imaging changes in the brain and cognitive impairment and dementia. The imaging will be conducted prior to commencement of the study medication and will be repeated at 4 years of follow up. The detailed STAREE MIND protocol is shown in appendix 10.



For peer review only - http://bmjopen.bmj.com/site/about/guidelines.xhtml

## 7.3 STAREE Healthy Ageing Biobank

Up to 2000 STAREE participants located near STAREE study sites with facilities for processing and storage of biospecimens (Melbourne, Perth and Hobart) will be invited to provide a blood and urine sample for the STAREE Healthy Ageing Biobank. This study aims to create a collection of biospecimens which can be used in future research to identify new biomarkers that are linked with development of clinical outcomes such as stroke, myocardial infarction, cancer and dementia.

For peer review only


For peer review only - http://bmjopen.bmj.com/site/about/guidelines.xhtml

## 8. Contingency plans for participant wellbeing

Participants will be advised to seek care from their usual treating GP (or other health care providers) for any medical condition arising during the course of the study. Any test measurement result that is outside the normal range will trigger a notification to the participant's GP.

Treating GPs will be co-investigators and provided with information about the study to aid the participant's involvement in the study (wherever possible).

### 8.1 Commencement of regular lipid lowering agents

A prescription for a lipid lowering agent will be reason to stop the study medication. The participant will cease study medication but continue in the study for observation (routine follow up visits).

### 8.2 Development of an indication for statins

Any participant who develops a recognised clinical indication (such as a myocardial infarction or stroke) for statin treatment may commence routine statin therapy. The participant will cease study medication but continue in the study for observation (routine follow up visits).

### 8.3 Post-trial care

At the end of the interventional phase of the trial, it is anticipated that participants will be followed passively for an additional 5 years, contingent on funding being obtained. During this period, study participants will continue to be treated by their usual GPs and follow up will be conducted annually by face to face or phone contact. Study participants will be asked to provide informed consent for the extended post-trial follow up.

For peer review only


For peer review only - http://bmjopen.bmj.com/site/about/guidelines.xhtml

# 9. Levels of participant involvement

## 9.1 Levels of participant involvement

The aim of the study is to assess outcomes on an intention to treat basis, i.e. to test the decision to prescribe a statin and the consequences of that decision. Therefore, every effort will be made to minimise loss to follow up with respect to determining mortality, dementia, disability and major adverse cardiovascular events. Loss to follow up will be minimised by recording three contacts for each participant including their GP, close relative living apart, and close friend.

Full participation in STAREE means that a participant is currently taking study medication and attending for study visits and/or phone calls. If participants request to change their level of involvement or withdraw from the study it will be explained to them that there are number of options. These may include the following:

*Changes to study medication*
Participants may decide to interrupt or discontinue the study medication for a number or reasons but regardless of this decision, they can continue to be active participants in STAREE:

> *On Medication:* Participants are taking study medication (one or two tablets daily).
>
> *Medication interrupted:* Participants have temporarily stopped taking study medication due to circumstances such as illness, injury or holidays or for other personal reasons. Participants who have interrupted their medication intend to re-start medication at a future date when the circumstances that have led to the interruption are resolved.
>
> *Medication discontinued:* Participants have stopped taking study medication permanently. This may be due to adverse effects or for other personal reasons, or because they have developed a clinical indication and been prescribed an open label statin. In all cases, when a participant discontinues study medication, the reason for discontinuation and date of the last dose of study medication will be recorded

Participants who have stopped taking study medication and are deemed by a STAREE medical officer as suitable for medication re-challenge will also be considered as "interrupted" until they either re-start or are deemed to have permanently discontinued medication.

*Changes to study visits (clinic and/or phone calls)*
Regardless of whether participants are taking study medication or not, they will still participate in study visits (clinic and/or phone calls). If participants do not want to participate in study visits (clinic and/or phone calls), they may elect to participate by review of their medical records corresponding with the trial's duration (such as GP and hospital records) and national morbidity and mortality databases). All participants will be asked to provide approval for access to this information as part of the study enrolment procedures.


For peer review only - http://bmjopen.bmj.com/site/about/guidelines.xhtml

## 9.2 Withdrawal

Participants will be considered to have withdrawn from the study if they revoke their consent. This means that they are not taking study medication, are not participating in study visits (clinic and/or phone calls), and that medical record review is not being conducted. All data that participants have contributed to the study up to the date of withdrawal will remain in the study database, and review of medical records dated earlier than the withdrawal date may be completed. Publicly available data such as death (e.g. from death notices) may also be used in such cases.

For peer review only


For peer review only - http://bmjopen.bmj.com/site/about/guidelines.xhtml

# 10. Study Medication and Supplies

## 10.1 Study treatments

*Run-in placebo*
The 4 week placebo medication will be provided in either a labelled box or bottle (one box or bottle of 40 tablets). It will be given to participants at the completion of baseline visit 1.

*Double-blind Study Medication*
Study medication will be provided by Ramsay Healthcare in boxed blister packs (3 sheets of 10 tablets), with the STAREE logo printed on the box. Participants will be randomly allocated to one of two treatments; a) atorvastatin (2 x 20 mg tablets) or b) placebo (2 x 20 mg tablets with identical appearance). Study medication will be posted regularly throughout follow-up, at least every 3-9 months (usually at 6-month intervals).

*Post randomisation*
After randomisation, study medication will be posted directly to the participant's home or provided to the participant at an annual visit. Participants will be directed to start taking one tablet of study medication from a labelled first box containing 30 tablets. Subsequently participants will be directed to take two tablets from boxes containing 30 tablets.

## 10.2 Administration of study medication

*Run-in period*
Participants will be directed to take 1 tablet of run-in medication at the same time every day.

*Double blind period*
Participants will be directed to take 1 tablet (20 mg) at the same time every day for the first 30 days of the trial. After 30 days, participants will be directed to take 2 tablets (40 mg) at the same time every day.

If a suspected intolerance of the full dose (2 tablets) of study medication (atorvastatin or placebo) develops, the participants' GP may reduce the dose to 1 tablet at their own discretion.

*Administration of study medication for those taking allowed Cytochrome P450 3A4 inhibitor at study entry and during follow up*
Participants identified as taking certain (allowed) cytochrome P450 3A4 inhibitors will be directed to take 1 tablet (20 mg) of study medication per day for the duration of the study. The list of allowed cytochrome P450 3A4 can be found in Appendix 3.

## 10.3 Dispensing and randomisation

The number of authorised dispensing staff will be limited to assure proper adherence with established accountability and dispensing procedures. All procedures are described in the STAREE Drug Accountability Manual.

Medication for the run-in phase will be dispensed to the participant by the study staff at baseline visit 1. Each participant will be dispensed 40 placebo tablets and be directed to take one tablet at the same time daily. When the participant returns for the final assessments and


For peer review only - http://bmjopen.bmj.com/site/about/guidelines.xhtml

eligibility visit (baseline visit 2), medication compliance will be checked prior to randomisation.

Following the completion of the randomisation process, study medication will be posted to the participant at regular intervals, as required, to ensure continuity of study medication supply for each participant. The participant will be asked to confirm receipt of the study medication by calling the STAREE coordinating centre. If confirmation of receipt has not been made within 10 days of dispatch of the study medication research, staff will contact the participant to ensure receipt. Attempts to contact the participant will continue until receipt has been confirmed. In the rare instance of missing or undelivered study medication, the study medication will be re-issued and sent to the home of the participant. Note, the postage of study medication to participants will be tracked with the delivery or courier service with the signature of the recipient being required upon delivery.

The Medication ID number assigned to the participant will remain the same for the duration of the study with each separate batch being distinguished by a batch number appended to the medication ID number. The participant will receive study medication with the same Medication ID number at each visit.

A '*Study Drug Log'* will record the medication dispensed and the medication returned from the participant.

The *Study Drug Log* will record information on the dispensing of study drug and will include the following:

- Participant ID number and participant acrostic
- Medication ID number
- Date dispensed (initials of dispenser)
- Date of medication return (initialled by research staff)
- Number of tablets returned
- Comments section to allow for explanation of discrepancy, if applicable.

These processes are further detailed in the drug accountability manual.

### 10.4 Study medication compliance

Compliance with study medication will be checked at six-monthly telephone calls and annual visits. Questions regarding study medication use will also be asked at annual visits to encourage medication compliance (see Appendix 4). The questions are based on the Brief Medication Questionnaire [65] and Morisky questionnaire[66]. Participants will be asked to count and report any unused tablets at annual visits.

### 10.5 Study medication storage

Study medication storage will be the responsibility of Ramsay Healthcare. Study medication will be predominantly stored at a secure storage centre in a temperature-controlled environment. Placebo run-in medication is released to STAREE periodically, in-bulk, and stored at the coordinating centre. All placebo medication for the run-in phase will be stored in a secured area with restricted access, at room temperature under the supervision of the coordinating centre.


For peer review only - http://bmjopen.bmj.com/site/about/guidelines.xhtml

The receipt, storage, dispensing, accountability and study medication collection, for both the run-in and randomisation phases, will be the responsibility of both Ramsay Healthcare and the STAREE coordinating centre. Each party will be responsible for maintaining appropriate delivery, storage, and transfer records.

## 10.6 Study medication accountability and dispensing logs

All study medication dispensed in the trial will be accounted for by confirming the delivery, receipt and commencement dates of each study medication supply. Study medication compliance will be assessed and confirmed by collection, counting and documentation of unused study medication from participants and/or by confirmation of the completion and/or cessation dates of each individual supply with participants by phone.

All unused study medication will be collected to allow for proper destruction of the medication. Unused tablets and packaging will be returned by participants to trial staff at face to face study visits. If unused study medication is not able to be collected at study visits, attempts will be made to confirm safe destruction (see section 8.7 below). Any lost medication packs will be documented, re-dispensed and posted to the participant, with all the details recorded to allow an audit trail. Tablet counts will be made of all returned medication packs. The coordinating centre will make all reasonable attempts to verify that any unused or partially used medication supplies are returned by the study participant and that no remaining supplies are in the participant's possession.

The coordinating centre will maintain records which adequately document that study participants were provided study medication supplies and doses as specified by the protocol and document and reconcile all study medication received from the participants (see section 8.3).

## 10.7 Study medication disposal

Unused study medication will be collected at annual visits by study staff whenever possible and disposed. Unused study medication will then be relinquished for safe destruction at a local pharmacy or destruction service. Each batch of destroyed study medication will be documented, along with the date, location and the pharmacy staff name, in the STAREE Study Medication Tracking Log (more detailed information is provided in the STAREE Drug Accountability Manual).

If unused study medication is not able to be collected at an annual study visit, participants will be given the option of disposing their supply at the local pharmacy (i.e. through the Return of Unwanted Medication or RUM scheme) or return via a reply-paid envelope satchel. Destruction of unused study medication by participants at pharmacies or other safe medical waste service will be documented on the STAREE Disposal Form, which will be returned to the STAREE trial for record keeping.

## 10.8 Overdose of study medication

There is no specific treatment for atorvastatin overdose. Should an overdose occur, the participant should seek emergency medical care and be treated symptomatically with


For peer review only - http://bmjopen.bmj.com/site/about/guidelines.xhtml

supportive measures instituted as required. The Poisons Information Centre on 13 11 26 (Australia) may also be contacted for advice on the management of an overdose.

## 11. Concomitant Therapy

### 11.1 Prescription medications

All prescription medicine will be recorded on a Concomitant Medications Form including indication for use and start/stop dates. Concomitant medications will be checked at annual study visits where participants will be asked to bring in any medications/vitamins/ supplements they are taking and by review of GP medical records.

For peer review only



For peer review only - http://bmjopen.bmj.com/site/about/guidelines.xhtml

## 12. Sample Size and Power calculations

Table 4. Sample size calculations

| | ITT effect size (RR) | Power | Required number of events | Required number of participants | On treatment (true) effect |
|---|---|---|---|---|---|
| For disability free survival (Death or Dementia or Disability) | 14.5% | 83.1% | 1,397 | 9,631 | 18% |
| For major adverse cardiovascular events (CV death, non-fatal MI or non-fatal stroke) | 20.2% | 80.0% | 619 | 9,631 | 25% |

Based on these sample size calculations 9,631 participants will be recruited and a total of 58,135 person-years accrued.

These calculations assume:

Loss to follow up: 2% per annum (which is expected to be conservative, i.e. the true loss to follow up is expected to be very small assuming good retention of participants and the ability to access medical records or perform record linkage for those who cease regular follow up). This translates to expecting an average of 6.04 years of follow up time per participant.

A two-sided significance level of 5% (alpha rate).

An anticipated age structure of STAREE participants of 56%: 27%: 12%: 5% in the respective age bands 70-74 years: 75-79 years: 80-84 years: 85+ years. These proportions are based on recruitment of the initial 7000 participants to the study and expectations about future recruitment.

Treatment cross-over: In year 1 of follow up we assume 8% of participants who have been randomised to statin will cease taking their study medication. Thereafter, we assume 3% of participants per annum in the statin group will cease taking study medication. In year 1 of follow up we assume 1% of participants in the placebo group will commence statin. Thereafter we assume 3% of participants per annum in the placebo group will commence statin therapy. Further we assume that there are no subsequent treatment "re-cross-overs", i.e. assume a worst-case scenario for the hazard ratio that will be observed in the intention to treat analysis.

The following event rates:

### Death, dementia or persistent disability (disability-free survival) - co-primary endpoint A

*All cause death*


For peer review only - http://bmjopen.bmj.com/site/about/guidelines.xhtml

We anticipate death rates in STAREE randomised participants will be less than half those in the general population. Specifically, the STAREE anticipated age-specific death rates are as follows:

| | |
|---|---|
| 70-74y | 10.0 per 1000 person-years |
| 75-79y | 17.7 per 1000 person-years |
| 80-84y | 33.6 per 1000 person-years |
| 85+y | 66.6 per 1000 person-years |

These event rates have been informed by corresponding mortality rates from the ASPREE (Aspirin in Reducing Events in the Elderly) randomised clinical trial and from underlying Australian population death rates for 2011, obtained from http://www.abs.gov.au/AUSSTATS/abs@.nsf/DetailsPage/3302.02011?OpenDocument

*Dementia*

Population rates of dementia have been estimated as 11 per 1000 person years in the 75-59 year old age group although fluctuations have been observed. [67-69] For Australia, there is a paucity of national data to inform incidence rates, as noted by AIHW in 2012. The ASPREE trial provides a highly relevant contemporary source of information. A dementia rate of 6.8 per 1000 person years was observed in ASPREE (median age 74 years) although almost no cases occurred in the first year of follow up so the rate was higher in later years. In ASPREE, dementia was screened for using a 3MS reduction of at least 10 points and there was a 60% conversion of the screen-positives to confirmed cases of dementia. In STAREE a 3MS reduction of at least 5 points will be used, which is expected to approximately double the number of screen positives but lower the conversion rate to confirmed cases of dementia to 50%. From these considerations, 3MS screening in STAREE is expected to yield 33% more cases of dementia than in ASPREE. Given this, and detailed age-specific data from ASPREE, the dementia rates in the later years of follow up in STAREE are expected to be as follows, using participant age at randomisation:

| | |
|---|---|
| 70-74y | 5.8 per 1000 person-years |
| 75-79y | 12.4 per 1000 person-years |
| 80-84y | 26.9 per 1000 person-years |
| 85+y | 36.6 per 1000 person-years |

*Physical Disability (defined as persistent loss of an activity of daily living)*

This component is defined by a persistent “a lot of difficulty” or “unable to do the activity” rating, or “Yes” answer to receiving help to perform the same activity of daily living (ADL) domain/s (items 9 to 14), which is confirmed at least 6 months previously as loss of the same ADL. If it is not possible to obtain an ADL assessment, notification of admission to aged care or community assessment will initiate collection of relevant clinical records to substantiate a disability endpoint. The rate of this endpoint was negligible in the first year of ASPREE. The following rates are expected to be seen in later years of follow up in STAREE and are based on observed rates in ASPREE, using participant age at randomisation:

For peer review only



For peer review only - http://bmjopen.bmj.com/site/about/guidelines.xhtml

70-74y 4.5 per 1000 person-years
75-79y 7.6 per 1000 person-years
80-84y 12.5 per 1000 person-years
85+y 25.0 per 1000 person-years

*Overlap of death, dementia and persistent physical disability*

The overlap between these three components will be assumed to be 10%. This is consistent with the observed overlap between dementia and physical disability occurring prior to death in ASPREE. Hence the composite STAREE event rate is estimated to be the sum of the 3 component event rates less 10% of that sum.

The calculations of expected primary endpoint event rates in years 3-5 of follow up after randomisation are as follows:

70-74y: (10.0 + 5.8 + 4.5)*0.9 per 1000 person-years = 18.3 per 1000 person-years
75-79y: (17.7 + 12.4 + 7.6)*0.9 per 1000 person-years = 33.9 per 1000 person-years
80-84y: (33.6 + 26.9 + 12.5)*0.9 per 1000 person-years = 65.7 per 1000 person-years
85+y: (66.6 + 36.6 + 25.0)*0.9 per 1000 person-years = 115.4 per 1000 person-years

*Consideration of healthy volunteer effect*

It is anticipated that the event rates for disability-free survival calculated above will be reached by STAREE participants in years 3-5 following randomisation. In the initial 2 years of follow up participants are assumed to be healthy relative to the population, i.e. in the first 2 years of follow up STAREE participants are assumed to experience lower event rates. An event rate of 0 (zero) per 1000 person years is assumed in the first year of follow up for dementia and physical disability and a rate of 4 per 1000 person years is assumed for mortality. In the second year of follow up it is assumed that the primary endpoint event rate will be equal to half of that anticipated above in years 3-5.

### Major adverse cardiovascular events (cardiovascular death, non-fatal myocardial infarction or non-fatal stroke) – co-primary endpoint B

This composite endpoint is defined as the first occurrence of cardiovascular death or non-fatal myocardial infarction or non-fatal stroke. In ASPREE this endpoint has been evaluated to the extent possible (of reported non-fatal myocardial infarction/angina events reported approximately 45% will be adjudicated as a non-fatal myocardial infarction). The figure below shows the estimated rate of major fatal and non-fatal cardiovascular events in ASPREE participants by age group. Of note, the underlying event rate seems fairly constant over the time from randomisation, i.e. the initial “healthy volunteer” effect that is seen in mortality and can be anticipated for admission to care, dementia and loss of activity of daily living, does not seem to apply to major adverse cardiovascular events. The three components, non-fatal myocardial infarction: non-fatal stroke: cardiovascular death, contribute events to the composite endpoint in the approximate ratio of 2:2:1 respectively.


For peer review only - http://bmjopen.bmj.com/site/about/guidelines.xhtml

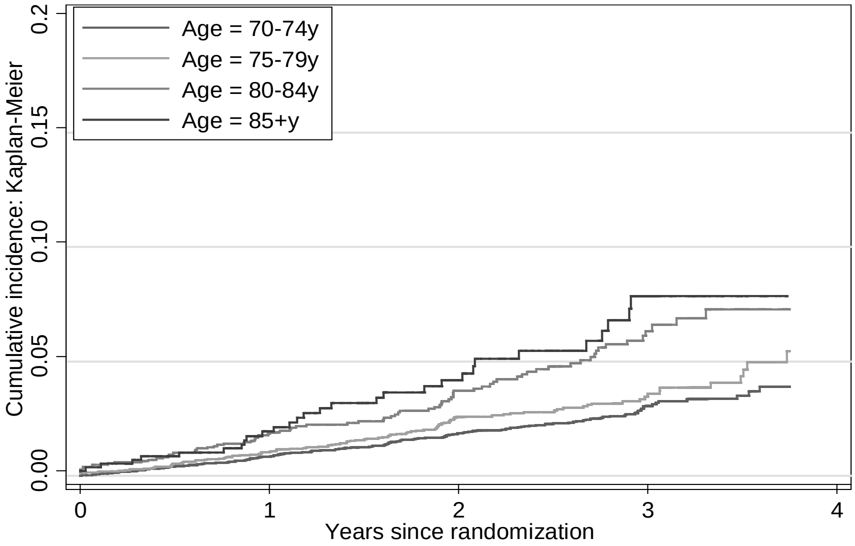


Based on the rates seen in ASPREE the following rates are anticipated in STAREE:

Years 1-5:

| | |
|---|---|
| 70-74y | 9.5 per 1000 person-years |
| 75-79y | 11.5 per 1000 person-years |
| 80-84y | 18.5 per 1000 person-years |
| 85+y | 24.5 per 1000 person-years |


For peer review only - http://bmjopen.bmj.com/site/about/guidelines.xhtml

## 13. Safety Monitoring and Reporting

### 13.1 Introduction

In STAREE the primary responsibility for the safety of individual participants will rest with the participant's usual general practitioner who is a co-investigator in the study. Additionally, an independent Data and Safety Monitoring Board (DSMB) will be appointed and will have primary responsibility for monitoring the study data for adverse trends and drug-related serious adverse events (SAEs). The responsibilities of the DSMB are outlined in the STAREE DSMB charter and DSMB plan.

### 13.2 Safety Monitoring

Several types of safety issues and SAEs may occur in STAREE participants. At each point of contact with participants, STAREE staff will specifically query participants for adverse events. Information on adverse events may also be reported to study staff between study visits by participants through contact with the trial centre by phone or email.

Participants will be encouraged to discuss any adverse events with their usual GP. GPs will be notified by STAREE staff of any abnormal test results and the STAREE medical officer will be available to discuss any issues with participants or their GPs.

### 13.2.2 Adverse events (AE)

The definition of an Adverse Event (AE) in the STAREE trial has been adapted from the National Institute of Aging definition. At the designated intervals for event collection, participants will be asked about specific events of interest as well as any new diseases or conditions. AEs will be obtained by self-report and some may require supporting documentation. AEs will be reported biannually to the Data Safety Monitoring Board (DSMB) by blinded treatment group.

AEs identified during monitoring will be classified by a trained medical officer or senior investigator as either serious or non-serious, and as potentially related or unrelated to study medication. If the AE is deemed related, therefore an adverse drug reaction (ADR or AR), further classification will occur regarding whether it is an expected or unexpected AR (see Appendix 5).

As the potential adverse effects of the active study drug in STAREE are well documented, AEs that are expected will not be considered as SAEs unless they meet the criteria for an SAE (section 11.2.3)

Source: AE definition adapted from the National Institute of Aging AE and SAE 2012 guidelines:
https://www.nia.nih.gov/sites/default/files/niaaeandsaeguidelinesfinal011012_0.doc

### 13.2.3 Serious adverse events (SAE)

Serious adverse events (SAEs) or Serious Adverse Drug Reactions (Serious ADRs) are defined as any unfavourable medical occurrence that:

- is fatal

For peer review only


For peer review only - http://bmjopen.bmj.com/site/about/guidelines.xhtml

- is life threatening [i.]
- requires in-patient-hospitalisation or prolongation of existing hospitalisation [ii.]
- causes persistent or significant disability/incapacity
- is a congenital anomaly /birth defect [iii.]
- is a medically important event or reaction [iv.] or
- is an accidental or intentional overdose.

Source: National Statement on Ethical Conduct in Human Research 2007; National Health and Medical Research Council Safety Monitoring and Reporting in Clinical Trials involving Therapeutic Goods November 2016

i. Life Threatening SAE: An SAE is considered life threatening when it places the participant at immediate risk of death from the event as it occurred.

ii. Hospitalisation: A hospitalisation is to be considered an SAE only if it is an official admission (inpatient) with a duration of more than 24hr or a minimum of 2 calendar days where exact time of stay is unavailable.

iii. Not applicable, since STAREE participants are aged 70 years or over at study entry

iv. In the opinion of the responsible investigator (i.e. an event that is not immediately life threatening, does not result in death or hospitalisation, but which may jeopardise the participant or require intervention to prevent one of the other SAEs listed above)

### 13.2.4 Suspected Unexpected Serious Adverse Events (SUSARs)

A SUSAR is defined as a SAE that is:

- not a predefined cardiovascular endpoint (see section 5),
- is unexpected (taking into account drug labelling and the participant history),
- is causally associated with the study medication based on a determination by a medical officer of 'definite', 'probable' or 'possible' using the Naranjo scale.

All SUSARs will be adjudicated by a study medical officer to determine if the event is unexpected in terms of both medication labelling and the participant's history.

A non-endpoint event will be deemed to be a SUSAR if:

- Evidence exists to support the possible association of the event with either or both of the medications (i.e. either the project office or the investigator has assessed the event as possibly causally related to blinded study medication); and
- The event is unexpected in terms of the product labelling for either product; and
- The event is unexpected in terms of the subject's history; and
- The event is serious as defined in section 11.2.


For peer review only - http://bmjopen.bmj.com/site/about/guidelines.xhtml

## 13.3 Safety reporting

In general, only AEs pre-specified to be of specific interest and those that are deemed to be serious will be reportable. AEs of interest are diabetes, myopathy (muscle symptoms) and liver impairment.

All SAEs will be reported from the study sites directly to the study medical officer within 24 hours of their notification to the clinical sites (study centres).

SAEs will be reviewed by the medical officer and adjudicated as related or unrelated to study medication, and as expected or unexpected serious adverse events.

All SAEs and SUSARS will be reported by the study centre to the ethics committees and DSMB according to the following schedule:

- Related and expected SAEs, will be reported to the Human Research and Ethics Committee (HREC)
- Related and unexpected SUSARs will be reported to the HREC within 72 hours and to the RACGP and TGA within 15 days and followed up with detailed reports when available
- Unrelated SAEs will be reported to the STAREE DSMB bi-annually

All SAEs and SUSARs will be reported to the DSMB in summary format by blinded treatment arm (A and B).

The data and safety monitoring reports will be prepared by an independent statistician.

## 13.4 Adverse Events of Interest

### 13.4.1 New diagnosis of diabetes

Diabetes as diagnosed on the basis of:

1) an annual HbA1c or fasting plasma glucose test using the WHO criteria (a HbA1c value of 6.5% or higher or a fasting plasma glucose of 7.0 mmol/L or higher, with confirmation by a second test within 4 weeks; or

2) community-based testing during usual clinical care (GP or other clinicians e.g. hospitalisation with a hyperglycaemic crisis or classic symptoms of hyperglycaemia and a random plasma glucose test of 11.1 mmol/L and/or commencement of glucose lowering medication for glucose control).

### 13.4.2 New diagnosis of myopathy

All participants will be advised to discuss a new diagnosis of myopathy with their usual treating GP. The decision to cease or reduce the study medication will be made by the GP in consultation with the participant. The participant will continue in the study on a reduced dose or remain off study medication and have routine follow up (study visits and phone calls) for observational purposes (see section 7.1).


For peer review only - http://bmjopen.bmj.com/site/about/guidelines.xhtml

### 13.4.3 Liver impairment

Liver impairment defined as:

1) an elevated ALT and/or AST across two or more tests (elevations greater than 3x ULN and elevated bilirubin (2x ULN)) and

2) no other demonstrable cause.

For peer review only


For peer review only - http://bmjopen.bmj.com/site/about/guidelines.xhtml

# 14. Adherence to Ethical, Regulatory and Administrative Considerations

## 14.1 Ethical considerations

### 14.1.1 General

This study will be conducted in accordance with the Declaration of Helsinki 1964 as revised in Edinburgh in 2000 and with the National Health & Medical Research Council (NHMRC) Guidelines on Human Experimentation.

### 14.1.2 Ethics Committee Approval

The primary ethics committee for the study is the Monash University Human Research Ethics Committee (Project ID 2787 and 21528).

The study has also been reviewed and approved by the following ethics committees:
Curtin University Human Research Ethics Committee (HR113/2015)
RACGP National Research and Evaluation Ethics Committee (14 - 017)
Tasmanian Health and Medical Research Ethics Committee (H0014918)
University of Newcastle Human Research Ethics Committee (H-2016-0266)

### 14.1.3 Information for participants

Before obtaining consent from the participant they must be informed of the objectives, benefits, risks and requirements of the study, as well as the nature of the study medication. A participant consent form will be provided to every participant prior to baseline visit 1 (posted if time permits) or at baseline visit 1.

### 14.1.4 Informed consent

a) All participants must give their written informed consent at baseline visit 1 to continue in the study.
b) Informed consent is obtained from the participant by the STAREE Field Officers. The study staff should fully inform the participant of all pertinent aspects of the STAREE study by reviewing the study information and consent form. All participants should be informed to the fullest extent possible about the study, in language and terms they are able to understand.
c) Prior to a participant’s involvement in the study, the written Informed Consent Form should be signed, name filled in and personally dated by the participant and by the staff member who conducted the informed consent discussion. A copy of the signed and dated written Informed Consent Form will be provided to the participant. The original consent form is to be stored in the participant’s individual study file, held by the investigator.
d) The form used for obtaining the participant's informed consent must be the current version that has been reviewed and approved by the appropriate Ethics Committee.

### 14.1.5 Protocol Amendments

All amendments and revisions to the trial protocol will be submitted to the Monash University Human Research Ethics Committee and on approval will be communicated to STAREE investigators and relevant staff through internal processes such as email and group meetings. All updates will be chronologically documented in the current version of the protocol.


For peer review only - http://bmjopen.bmj.com/site/about/guidelines.xhtml

### 14.1.6 Confidentiality

All study-related information will be stored securely at the study sites. All participant information will be stored in locked filing cabinets in areas accessible only by STAREE personnel. All participant CRFs will contain a coded identification number only to maintain participant confidentiality. All records that contain names or other personal identifiers, such as informed consent forms, will be stored separately from study records identified by code number. All local databases will be secured with password-protected access systems. Other forms and lists, that link participant ID numbers to other identifying information will be stored in a separate, locked file in an area with limited access. Hardcopy information will be uploaded into a secure study database housed in the high security Monash University Data Centre at Clayton, Melbourne. All information collected for this project will be retained for a period of no less than 15 years following the completion of the project. Hardcopy information will be shredded and destroyed, and electronic data will be deleted from the secure STAREE database after a period of no less than 15 years after the completion of the project, in accordance with the Monash University Human Research Ethics Committee recommendations for drug trials and Good Clinical Practice Guidelines.

## 14.2 Regulatory considerations

### 14.2.1 Financing

Monash University is the sponsor of the STAREE trial.

STAREE has been awarded project grants from the NHMRC (Australia) (APP1068146 and APP1161503) and from the Heart Foundation (#101663). STAREE MIND and STAREE HEART have been awarded NHMRC grant funding (#2006611 and #1165440).

### 14.2.2 Trial registration

STAREE is registered with clinicaltrials.gov (Identifier NCT02099123). First posted March 28, 2014.

### 14.2.3 Declaration of interest

The STAREE investigators have established a policy regarding Conflict of Interest. The policy aims to protect the integrity of the project's decision-making process and to manage risk. The policy requires full disclosure by all of the investigators of their, and their immediate families, financial relationships with all pharmaceutical and biomedical companies judged to have an active or potential interest in the conduct and outcome of the study. The disclosures of each project investigator will be documented in all publications.

## 14.3 Indemnity

Monash University shall at all times indemnify the study investigators and their staff from claims that may be made against them for any injury sustained by a study participant as a consequence of the follow up for this study as outlined in this protocol. GPs insurers will be asked to indemnify the GP co-investigators who are participating in the study. Ramsay Healthcare will indemnify the study medication.


For peer review only - http://bmjopen.bmj.com/site/about/guidelines.xhtml

## 14.4 Data Access

The Data Access and Publications Committee will oversee the intra-study data sharing process, with input from the STAREE Executive Committee.

Access to STAREE data for analysis, preparation and submission of publications and presentations will be decided according to the STAREE Data Access Policy and the STAREE Publications Policy.

The policies are based on the following principles:

- STAREE data is to be used for the purposes of furthering the public good in relation to health.
- STAREE data is a collaborative resource. Data elements cannot be reserved by researchers for exclusive use.
- STAREE data is to be used for projects that are scientifically, methodologically and ethically sound.
- The privacy of STAREE participants is to be protected.

## 14.5 Governance

*Central Coordinating Centre*

The STAREE Central Coordinating Centre is located at the School of Public Health and Preventive Medicine, Monash University, Melbourne.

*Steering Committee (SC)*

STAREE is overseen and coordinated by a steering committee comprised of Prof Sophia Zoungas (Chair), Monash University; Prof John McNeil, Monash University; Prof Andrew Tonkin, Monash University; Prof Rory Wolfe, Monash University; Prof Christopher Reid, Monash University; Prof Mark Nelson, University of Tasmania; Prof Lawrie Beilin, University of Western Australia; Prof Stephen Nicholls, Monash University; Associate Professor Trevor Chong, Monash University, Prof Geoffrey Cloud, Monash University, Dr Stephanie Ward, Monash University and Prof Anthony Wierzbicki (King's College, UK).

The Steering Committee will provide oversight to the overall policy and direction of the project. It will be responsible for protocol generation and, where appropriate, modification, budgeting and all funding applications. The Steering Committee will meet regularly throughout the planning, execution and data analysis phases of the project, when it will consider reports from the Study Director and the EAC. The Steering Committee will be responsible for all the publications and communications relating to the study.

For peer review only


For peer review only - http://bmjopen.bmj.com/site/about/guidelines.xhtml

STAREE Governance Structure

## 14.6 Administrative Organisation

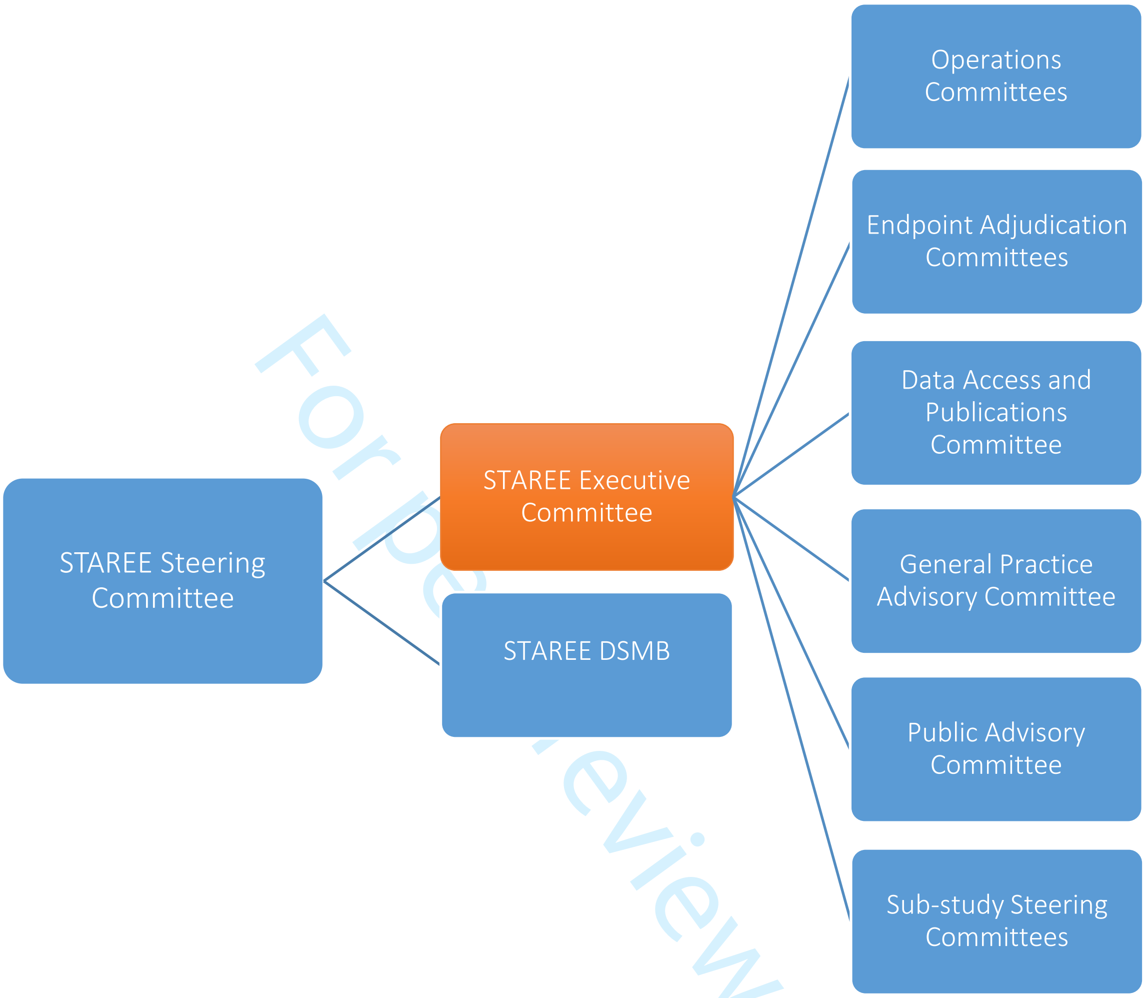


### 14.6.1 Individual and committee responsibilities

*Principal Investigators:* Prof Sophia Zoungas and Prof John McNeil

***STAREE Executive Committee***

The STAREE Executive Committee will be responsible for the overall design, management and conduct of STAREE including preparation of the protocol and revisions, preparation of investigators brochure and case report forms (CRFs), publication of study reports, approving the operational plan for the study, and financial management of the trial. The committee will monitor the progress of recruitment against targets, and if necessary, institute remedial actions. It will also review the rates of adherence to randomised treatments and the data from the quality assurance program.

***National Operations Committee***

The National Operations Committee will be responsible for implementing the study, monitoring the progress of recruitment and ensuring adherence and standardisation through the study. It will also need to ensure the coordination, consistency and transfer of data to the Data Management Centre. The committee will ensure quality control review of laboratory



For peer review only - http://bmjopen.bmj.com/site/about/guidelines.xhtml

data, clinical measurements and data collection, completeness and entry times of data, site monitoring and source documentation. These committees may consist of the Principal Investigator or Project Managers and other key research personnel who will report directly to the Steering Committee.

### *Internal Data Monitoring Committee (DMC)*

The STAREE Internal Data Monitoring Committee (DMC) is responsible for reviewing information related to the conduct of the trial and monitoring activity as per STAREE's Monitoring Plan, Monash University's Research Data Management Guidelines and the NHMRC's Australian Code for the Responsible Conduct of Research. The STAREE Internal DMC shall make recommendations to the Monash University STAREE Executive Committee. Recommendations shall include all aspects of Data Monitoring as per the STAREE Monitoring Plan.

DMC members will be appointed by the Executive and Management of the STAREE Clinical Trial and may be involved in the study's collection and management of data, including data monitoring. Appointed committee members will include a Committee Chair, Data Statistician and an individual who has had previous experience with a DMC or experience with trials of medicinal investigational products.

### *Data Safety and Monitoring Board*

The Data Safety and Monitoring Board (DSMB) will monitor the establishment and implementation of the STAREE Data Management System and review all safety, data and quality assurance reports for the study. The DSMB chair will report to the Steering Committee of STAREE on data management issues associated with the study and will provide recommendations to the Steering Committee in relation to subject recruitment, event rate tracking, monitoring adverse events and conducting interim analyses. The committee will operate in accordance with the DSMB Charter and Plan.

The DSMB will have responsibility for monitoring quality control of the data, progress of recruitment and safety aspects of the trial. The DMC will present data for review by the DSMB by a blinded treatment arm (e.g., 'A' and 'B'). The DSMB will monitor all-cause mortality rates in the two randomised groups. Additionally one pre-specified interim analysis may occur when an average of 3.25 years of follow up per participant has accrued and >50% of the primary endpoints have occurred. The DSMB will be responsible for reviewing any interim analysis on unblinded data and providing recommendations to the trial executive committee. The DSMB will review deaths, serious adverse events and other endpoint data on a periodic basis. Administrative support will be provided by an independent biostatistician and research assistant.

The DSMB will be provided with data every 6 months or as requested. The DSMB will meet annually for the first 2 years and thereafter will meet twice annually, by teleconference or in-person where possible to review study progress, data quality control, address policy issues and review total mortality data, adverse events and all safety data and monitor the study progress and data quality. The DSMB has expertise related to the conduct of clinical trials *per se* and in the primary care sector, the elderly, epidemiology, biostatistics, clinical pharmacology, clinicians, and cardiovascular disease. Confidentiality will be maintained


For peer review only - http://bmjopen.bmj.com/site/about/guidelines.xhtml

during all phases of the trial including monitoring, preparation of interim results, review, and response to monitoring recommendations.

*Endpoint Adjudication Committees (EACs)*
The Endpoint Adjudication Committees (EACs) will be responsible for developing adjudication criteria and processes to capture key data required for the identification and adjudication of the following study endpoints: All-cause mortality, myocardial infarction, heart failure, stroke, and dementia. The EACs will operated in accordance with the Endpoint Adjudication Committee Charter.

The following EACs will be responsible for the assessment of suspected endpoints through examination of relevant clinical information and for their adjudication according to the pre-specified endpoint definitions.

Death Subcommittee
Dementia Subcommittee
Heart Disease (Myocardial Infarction) Subcommittee
Heart Disease (Heart Failure) Subcommittee
Stroke Subcommittee

The EAC subcommittees will be chaired by specialist clinicians with expertise to oversee adjudication of the relevant endpoints. All EAC members will be selected by the STAREE Executive Committee and will be appointed for the duration of the study. Administrative support, training and source documentation required for EAC members to carry out their roles will be provided by the EAC Coordinator and STAREE study staff.

*Data Access and Publications Committee*
The Data Access and Publications Committee will be responsible for developing and implementing the data access, sub-studies and ancillary studies, and publication policies. It will oversee the processes of receiving, reviewing and approving applications from researchers for STAREE baseline data, for conduct of sub-studies and ancillary studies, and requests for publication, approval for abstracts and submission to conferences. Authorship of publications will be guided by the STAREE data access and publications policies.

*General Practice Advisory Committee*
The General Practice Advisory Committee will advise the National Operations Committee and other committees on aspects of the trial related to family practice.

*Public Advisory Group*
The STAREE Public Advisory Group will be a forum to facilitate the flow of information from the STAREE clinical trial to the public. It will also provide opportunities for members of the group to provide feedback and input from the community perspective on how information is disseminated to the public about the STAREE trial.

*STAREE Healthy Ageing Biobank Steering committee*
Access and utilisation of the biospecimens stored in the STAREE Healthy Ageing Biobank will be governed by the STAREE Healthy Ageing Biobank steering committee. The STAREE

For peer review only


For peer review only - http://bmjopen.bmj.com/site/about/guidelines.xhtml

Healthy Ageing Biobank steering committee will review detailed proposals outlining the research to be undertaken, and access to biospecimens will only be granted if the proposals are assessed to have scientific merit and are adequately funded. Accurate records of the aliquots of each stored biospecimen will be achieved through the use of a Nunc Next Generation CryoTube Scanner and software. The software will be utilised to track each sample which will include storage or retrieval of a specimen by an approved researcher. Any secondary research which uncovers significant genetic information will be notified to the Biobank Steering committee, which will determine who will be notified about these results.

*STAREE MIND Steering committee*

The STAREE MIND Steering Committee will oversee integration of STAREE MIND with the parent trial. It will be responsible for design, management and conduct of STAREE MIND including preparation of the protocol, investigators brochures and case report forms (CRFs), publication of study reports, approval of the operational plan, financial management and will monitor the progress of recruitment. The STAREE MIND committee will report on progress to the STAREE Steering and Executive committees.

*STAREE HEART Steering committee*

The STAREE HEART Steering Committee will oversee integration of STAREE HEART with the parent trial. It will be responsible for design, management and conduct of STAREE HEART including preparation of the protocol, investigators brochures and case report forms (CRFs), publication of study reports, approval of the operational plan, financial management and will monitor the progress of recruitment. The STAREE HEART committee will report on progress to the STAREE Steering and Executive committees.

For peer review only


For peer review only - http://bmjopen.bmj.com/site/about/guidelines.xhtml

## 14. Data Management and Training

### 14.1 Case report forms

All data from assessments conducted during the study will be recorded on an appropriate case report form (CRF) for each participant. Electronic CRFs will be accessible from the STAREE website. The CRFs must be completed during or after each participant visit (or as soon as all data is available, e.g. once pathology results are obtained) and uploaded onto the STAREE web-based portal. Original hard-copy CRFs and other source documents will be stored in the participant's file at the local study site.

CRFs will be kept current to reflect the participant's course throughout the study. Participants will not be identified on the CRF by name. Appropriately coded identification (GP, Practice and Subject Identification Numbers) and an acrostic of the participant's initials will be used.

The data from the CRFs will be uploaded onto the STAREE web-based data management portal. All information collected will be treated in accordance with professional and ethical guidelines. All corrections and alterations of data on the CRFs must be made according to the instructions provided and must be dated and initialled.

### 14.2 Data Management

Data collection and management will be centralised at the STAREE data management centre, Department of Epidemiology and Preventive Medicine, Monash University, Melbourne. Study data collected at local study sites will be entered on electronic CRFs with source documentation retained in the participant file. All information related to potential endpoints will be copied from the original documents sourced through clinical notes and hospital medical records and sent de-identified to the STAREE data management centre. Serious adverse event and endpoint data will be accepted as either a) photocopied clinical records or b) scanned or photographed image files of clinical records. The clinic will retain all original information related to the clinical event or endpoint in the subject files.

Electronic study records will be accessible through the internet at the STAREE web-portal. Access to this website will be made available to registered study staff via password protected log-in procedures.

Monash University implements a defence in depth approach to information security and employs a multitude of controls to protect the study's infrastructure and data. These controls are regularly audited to ensure they meet global best practices and are aligned with ISO 27001 security practices. Data collected as part of the STAREE clinical trial will be stored on University managed secure and resilient infrastructure located in Australia that complies with all applicable data protection and privacy obligations. Some specialist data collected as part of the STAREE clinical trial may be entered into applications that are operated by external third parties. This will only be permitted where all applicable data protection and privacy obligations are in place with those external third parties.

Monash University values the privacy of every individual's personal information and is committed to the protection of that information from unauthorised use and disclosure, except where permitted by law. Monash University is subject to and ensures that personal

For peer review only


For peer review only - http://bmjopen.bmj.com/site/about/guidelines.xhtml

and health information it holds or has access to, is handled in accordance with data protection and privacy legislation, including the Privacy and Data Protection Act 2014 (Vic) and the Health Records Act 2001 (Vic) and where required, the Privacy Act 1988 (Cth) and applicable international regulations.

## 14.3 Data analysis

The data will be analysed by statisticians from the Biostatistics Unit, Department of Epidemiology and Preventive Medicine, Monash University.

Baseline characteristics of the two treatment groups will be tabulated and imbalance defined as a 0.25 standard deviation difference in means (quantitative characteristics) or an odds ratio of 1.5 (binary characteristics).

All comparisons between treatment arms will be on an intention-to-treat basis i.e. according to the group to which participants were randomised and without reference to their actual compliance with assigned treatment.

Each of the co-primary endpoints will be analysed separately in time-to-event analyses. Event rates (time to first event within each endpoint definition) will be compared between groups using a hazard ratio, 95% confidence interval and two-sided p-value from a Cox proportional hazards regression model fitted to the endpoint with censoring for non-events at their most recent study visit and a single covariate being an indicator of the group to which the individual was randomised, statin or placebo. The proportional hazards assumption will be tested for each model.

A gate-keeping procedure will be used to adjust for the multiple testing created by the presence of two co-primary endpoints. First, MACE will be tested at alpha=0.05 and, if the MACE p-value is <0.05 then second, disability-free survival will be tested at alpha=0.05.

Secondary analyses of the co-primary endpoints will be performed using extended Cox proportional hazards regression models based on time(s) to any event within the endpoint definition (for relevant endpoints that can occur more than once).

No statistical adjustment will be made for the multiple secondary endpoints but the reporting of all secondary endpoint analyses will make clear whether any of the co-primary endpoints were statistically significant and will state the number of secondary endpoints proposed a priori in the study protocol.

In the survival analyses, loss to follow up will be considered a censoring event. This equates to an assumption that data is missing at random given the participant's treatment group and the timing of their loss to follow up. The adequacy of this assumption will be checked in sensitivity analyses that will include both imputation approaches and adjustment for baseline covariates predictive of propensity for dropout. The analyses will be performed using commercial statistical software packages (Stata or SAS).


For peer review only - http://bmjopen.bmj.com/site/about/guidelines.xhtml

### 14.4 Training

All STAREE staff will be trained by senior study staff and study investigators and undertake a competency assessment prior to commencing their specific role.

All Staff will be provided with background information on the rationale of the STAREE trial and the study protocol. They will also be required to familiarise themselves with the key principles of Good Clinical Practice (GCP) and will attend an accredited GCP training course.

All study activities are described in standard operating procedures (SOPs), and staff will be regularly encouraged to refer to SOPs and SOP updates for guidance.

#### 14.4.1 Research Officers

STAREE Research Officers are responsible for and will be trained in the conduct of phone screening for potential study participants, in use of the STAREE database, booking and confirming participant visits, conducting 6 month participant phone calls and labelling and dispatch of medication, receipt of study medication and follow up missing documents for randomisation and endpoint ascertainment.

#### 14.4.2 GP Liaison Officers

STAREE GP Liaison Officers are responsible for engaging with general practices and registering GPs as STAREE co-investigators. They will be provided with practical guidance in conducting GP practice visits to recruit GPs, in data entry, and in searching GP practice databases for potential participants.

#### 14.4.3 Research Field Officers

STAREE Field Officers are responsible for conducting baseline screening visits and annual follow up visits with study participants. They will receive training in database entry, conducting informed consent discussions with potential participants, visit conduct and performing specific study assessments (e.g. weight, height and blood pressure measurement and taking personal and medical histories) and assessing the suitability of a participant to participate in the trial.

Field Officers will observe baseline 1 and 2 and annual visits that are being conducted by a trained Field Officer to become familiar with the visit procedures. Training in the conduct of cognitive assessments will be provided in the form of training videos, SOPs and role plays. Before Field Officers can conduct independent study visits, they will undertake specific training in cognitive assessments with a Senior Field Officer and specialist STAREE Investigator and conduct 3-6 study visits under the supervision from a Senior Field Officer.

Accreditation to perform study visits will be provided subject to satisfactory performance in both the cognitive assessment training and supervised visit conduct. To maintain accreditation, Field Officers will be required to conduct a minimum of 4 visits per month (at least 2 with cognitive assessments) and performance will be reviewed annually.


For peer review only - http://bmjopen.bmj.com/site/about/guidelines.xhtml

## 15. Study Monitoring and Auditing

Monitoring and auditing in STAREE will include monitoring of investigative sites and visit conduct by study staff. This will involve monitoring of all study documentation and activities including participant files, data quality, administrative activities and endpoint collection as detailed further in the STAREE monitoring plan.

All STAREE study sites will be monitored annually by trained STAREE clinical research Site Monitors, following GCP guidelines. Site monitoring will be undertaken at all STAREE sites to ensure that the study is being conducted according to the protocol and appropriate regulatory requirements, that protocol deviations are appropriately documented and reported, the site complies with safety regulations and that personnel at each site meet GCP obligations.

All field staff involved in the STAREE study will have their activity reviewed by the Visit Conduct Monitor. Monitoring will ensure that all visit activity is conducted in accordance with the study protocol, relevant SOPs and test administration SOPs.

Study monitors will submit written reports summarising their findings to the internal Data Monitoring Committee (DMC). Where monitoring identifies risks or issues for participant safety or trial processes, appropriate steps will be taken to remedy the issue including re-training of staff and repeat monitoring.

For peer review only


For peer review only - http://bmjopen.bmj.com/site/about/guidelines.xhtml

## 16. Dissemination Plan

The STAREE dissemination plan will ensure that the outputs from the research inform general practice and maximise the benefit to older people.

The key audiences for the STAREE results are:

- General practitioners
- Physicians and other health professionals/clinicians
- STAREE participants
- The general public (including community groups such as Council of the Older Australian, Probus groups etc.)
- Professional organisations (e.g. RACGP)
- Funding organisations (e.g. NHMRC, Monash University)
- Scientific and medical research communities

Representatives of these key stakeholder groups will be invited to take part in discussion of the STAREE findings in relation to current clinical practice and policy and how to best translate the research findings into recommendations for wider audiences.

Multiple channels of communication will be used to disseminate STAREE findings to the wider community. These will include the following:

- Written feedback to STAREE participants and GP co-investigators on the study results and recommendations arising from the study.
- Published academic journal articles, plain English summaries, research summaries for professional journals, newsletters, presentations, and frequently asked questions section will be made accessible on the STAREE website.
- Workshops and presentations at scientific meetings, GP conferences and community and public venues.


For peer review only - http://bmjopen.bmj.com/site/about/guidelines.xhtml

## 17. References


1. Bongaarts J. Human population growth and the demographic transition. Philos Trans R Soc Lond B Biol Sci. 2009;364(1532):2985-90.
2. Christensen K, Doblhammer G, Rau R, Vaupel JW. Ageing populations: the challenges ahead. Lancet. 2009;374(9696):1196-208.
3. Jacobsen L, Mather M, Lee M, Kent M. America's Ageing Population. Population Bulletin. 2011;66:2-3.
4. Productivity Commission 2011, Caring for Older Australians: Overview, Report No. 53, Final Inquiry Report, Canberra.
5. Zoungas S, Curtis A, Tonkin A, McNeil J. Statins in the elderly: an answered question? Curr Opin Cardiol. 2014;29(4):372-80.
6. Jonsson A, Sigvaldason H, Sigfusson N. Total cholesterol and mortality after age 80 years. Lancet. 1997;350(9093):1778-9.
7. Schatz IJ, Masaki K, Yano K, Chen R, Rodriguez BL, Curb JD. Cholesterol and all-cause mortality in elderly people from the Honolulu Heart Program: a cohort study. Lancet. 2001;358(9279):351-5.
8. Volpato S, Leveille SG, Corti MC, Harris TB, Guralnik JM. The value of serum albumin and high-density lipoprotein cholesterol in defining mortality risk in older persons with low serum cholesterol. J Am Geriatr Soc. 2001;49(9):1142-7.
9. Weverling-Rijnsburger AW, Jonkers IJ, van Exel E, Gussekloo J, Westendorp RG. High-density vs low-density lipoprotein cholesterol as the risk factor for coronary artery disease and stroke in old age. Arch Intern Med. 2003;163(13):1549-54.
10. Foody JM, Wang Y, Kiefe CI, Ellerbeck EF, Gold J, Radford MJ, et al. Long-term prognostic importance of total cholesterol in elderly survivors of an acute myocardial infarction: the Cooperative Cardiovascular Pilot Project. J Am Geriatr Soc. 2003;51(7):930-6.
11. Schupf N, Costa R, Luchsinger J, Tang MX, Lee JH, Mayeux R. Relationship between plasma lipids and all-cause mortality in nondemented elderly. J Am Geriatr Soc. 2005;53(2):219-26.
12. Prospective Studies C, Lewington S, Whitlock G, Clarke R, Sherliker P, Emberson J, et al. Blood cholesterol and vascular mortality by age, sex, and blood pressure: a meta-analysis of individual data from 61 prospective studies with 55,000 vascular deaths. Lancet. 2007;370(9602):1829-39.
13. Stancu C, Sima A. Statins: mechanism of action and effects. J Cell Mol Med. 2001;5(4):378-87.
14. Nicholls SJ, Tuzcu EM, Sipahi I, Grasso AW, Schoenhagen P, Hu T, et al. Statins, high-density lipoprotein cholesterol, and regression of coronary atherosclerosis. JAMA : the journal of the American Medical Association. 2007;297(5):499-508.
15. Maron DJ, Fazio S, Linton MF. Current perspectives on statins. Circulation. 2000;101(2):207-13.
16. Greenwood J, Mason JC. Statins and the vascular endothelial inflammatory response. Trends Immunol. 2007;28(2):88-98.
17. Efficacy and safety of statin therapy in older people: a meta-analysis of individual participant data from 28 randomised controlled trials. Lancet. 2019;393(10170):407-15.
18. Cholesterol Treatment Trialists C, Mihaylova B, Emberson J, Blackwell L, Keech A, Simes J, et al. The effects of lowering LDL cholesterol with statin therapy in people at low risk of vascular disease: meta-analysis of individual data from 27 randomised trials. Lancet. 2012;380(9841):581-90.


For peer review only


For peer review only - http://bmjopen.bmj.com/site/about/guidelines.xhtml

19. Afilalo J, Duque G, Steele R, Jukema JW, de Craen AJ, Eisenberg MJ. Statins for secondary prevention in elderly patients: a hierarchical bayesian meta-analysis. Journal of the American College of Cardiology. 2008;51(1):37-45.
20. Shepherd J, Blauw GJ, Murphy MB, Bollen EL, Buckley BM, Cobbe SM, et al. Pravastatin in elderly individuals at risk of vascular disease (PROSPER): a randomised controlled trial. Lancet. 2002;360(9346):1623-30.
21. Han BH, Sutin D, Williamson JD, Davis BR, Piller LB, Pervin H, et al. Effect of Statin Treatment vs Usual Care on Primary Cardiovascular Prevention Among Older Adults: The ALLHAT-LLT Randomized Clinical Trial. JAMA internal medicine. 2017;177(7):955-65.
22. Glynn RJ, Koenig W, Nordestgaard BG, Shepherd J, Ridker PM. Rosuvastatin for primary prevention in older persons with elevated C-reactive protein and low to average low-density lipoprotein cholesterol levels: exploratory analysis of a randomized trial. Annals of internal medicine. 2010;152(8):488-96, w174.
23. Ridker PM, Lonn E, Paynter NP, Glynn R, Yusuf S. Primary Prevention With Statin Therapy in the Elderly: New Meta-Analyses From the Contemporary JUPITER and HOPE-3 Randomized Trials. Circulation. 2017;135(20):1979-81.
24. Routledge PA, O'Mahony MS, Woodhouse KW. Adverse drug reactions in elderly patients. Br J Clin Pharmacol. 2004;57(2):121-6.
25. Brugts JJ, Yetgin T, Hoeks SE, Gotto AM, Shepherd J, Westendorp RG, et al. The benefits of statins in people without established cardiovascular disease but with cardiovascular risk factors: meta-analysis of randomised controlled trials. BMJ. 2009;338:b2376.
26. Bellosta S, Paoletti R, Corsini A. Safety of statins: focus on clinical pharmacokinetics and drug interactions. Circulation. 2004;109(23 Suppl 1):III50-7.
27. Graham DJ, Staffa JA, Shatin D, Andrade SE, Schech SD, La Grenade L, et al. Incidence of hospitalized rhabdomyolysis in patients treated with lipid-lowering drugs. JAMA : the journal of the American Medical Association. 2004;292(21):2585-90.
28. Davidson MH, Robinson JG. Safety of aggressive lipid management. Journal of the American College of Cardiology. 2007;49(17):1753-62.
29. Sathasivam S. Statin induced myotoxicity. European journal of internal medicine. 2012;23(4):317-24.
30. Hilmer S, Gnjidic D. Statins in older adults. Australian Prescriber. 2013;36(3):79-82.
31. Sattar N, Preiss D, Murray HM, Welsh P, Buckley BM, de Craen AJ, et al. Statins and risk of incident diabetes: a collaborative meta-analysis of randomised statin trials. Lancet. 2010;375(9716):735-42.
32. FDA drug safety communication: Important safety label changes to cholesterol-lowering statin drugs 2012.
33. Cholesterol Treatment Trialists C, Emberson JR, Kearney PM, Blackwell L, Newman C, Reith C, et al. Lack of effect of lowering LDL cholesterol on cancer: meta-analysis of individual data from 175,000 people in 27 randomised trials of statin therapy. PloS one. 2012;7(1):e29849.
34. Ott BR, Daiello LA, Dahabreh IJ, Springate BA, Bixby K, Murali M, et al. Do Statins Impair Cognition? A Systematic Review and Meta-Analysis of Randomized Controlled Trials. Journal of general internal medicine. 2015.
35. Catapano AL, Graham I, De Backer G, Wiklund O, Chapman MJ, Drexel H, et al. 2016 ESC/EAS Guidelines for the Management of Dyslipidaemias. European heart journal. 2016;37(39):2999-3058.

For peer review only


For peer review only - http://bmjopen.bmj.com/site/about/guidelines.xhtml

36. Naci H, Brugts J, Ades T. Comparative tolerability and harms of individual statins: a study-level network meta-analysis of 246 955 participants from 135 randomized, controlled trials. Circ Cardiovasc Qual Outcomes. 2013;6(4):390-9.
37. Bays H, Cohen DE, Chalasani N, Harrison SA. An assessment by the Statin Liver Safety Task Force: 2014 update. Journal of clinical lipidology. 2014;8(3):S47-S57.
38. Lindsley CW. 2012 trends and statistics for prescription medications in the United States: CNS therapeutics continue to hold leading positions. ACS chemical neuroscience. 2013;4(8):1133-5.
39. PBS Information Management Section. Pharmaceutical Policy Branch. Expenditure and prescriptions twelve months to 30 June 2014.
40. Simons LA, Ortiz M, Calcino G. Long term persistence with statin therapy -- experience in Australia 2006-2010. Australian family physician. 2011;40(5):319-22.
41. Trusler D. Statin prescriptions in UK now total a million each week. Bmj. 2011;343:d4350.
42. Sheppard JP, Singh S, Fletcher K, McManus RJ, Mant J. Impact of age and sex on primary preventive treatment for cardiovascular disease in the West Midlands, UK: cross sectional study. Bmj. 2012;345:e4535.
43. Chokshi NP, Messerli FH, Sutin D, Supariwala AA, Shah NR. Appropriateness of statins in patients aged >/=80 years and comparison to other age groups. The American journal of cardiology. 2012;110(10):1477-81.
44. Robinson JG, Booth B. Statin use and lipid levels in older adults: National Health and Nutrition Examination Survey, 2001 to 2006. Journal of clinical lipidology. 2010;4(6):483-90.
45. Morgan TK, Williamson M, Pirotta M, Stewart K, Myers SP, Barnes J. A national census of medicines use: a 24-hour snapshot of Australians aged 50 years and older. The Medical journal of Australia. 2012;196(1):50-3.
46. National Vascular Disease Prevention Alliance. Guidelines for the management of absolute cardiovascular disease risk. 2012.
47. Shepherd J, Cobbe SM, Ford I, Isles CG, Lorimer AR, MacFarlane PW, et al. Prevention of coronary heart disease with pravastatin in men with hypercholesterolemia. West of Scotland Coronary Prevention Study Group. The New England journal of medicine. 1995;333(20):1301-7.
48. Stone NJ, Robinson JG, Lichtenstein AH, Bairey Merz CN, Blum CB, Eckel RH, et al. 2013 ACC/AHA guideline on the treatment of blood cholesterol to reduce atherosclerotic cardiovascular risk in adults: a report of the American College of Cardiology/American Heart Association Task Force on Practice Guidelines. Journal of the American College of Cardiology. 2014;63(25 Pt B):2889-934.
49. East Melbourne: Royal Australian College of General Practitioners. Guidelines for preventative activities in general practice. (2012).
50. NICE. Chronic Kidney Disease - Early identification and management of chronic kidney disease in adults in primary and secondary care. (2014).
51. Collins R, Reith C, Emberson J, Armitage J, Baigent C, Blackwell L, et al. Interpretation of the evidence for the efficacy and safety of statin therapy. The Lancet. 2016;388(10059):2532-61.
52. Ridker PM, Pradhan A, MacFadyen JG, Libby P, Glynn RJ. Cardiovascular benefits and diabetes risks of statin therapy in primary prevention: an analysis from the JUPITER trial. Lancet. 2012;380(9841):565-71.


For peer review only - http://bmjopen.bmj.com/site/about/guidelines.xhtml

53. Ganga HV, Slim HB, Thompson PD. A systematic review of statin-induced muscle problems in clinical trials. American heart journal. 2014;168(1):6-15.
54. Bland RC, Newman SC. Mild dementia or cognitive impairment: the Modified Mini-Mental State examination (3MS) as a screen for dementia. Canadian journal of psychiatry Revue canadienne de psychiatrie. 2001;46(6):506-10.
55. Radloff LS. The CES-D Scale: A Self-Report Depresion Scale for Research in the General Population. Applied Psychological Measurement. 1977;1(3):385-401.
56. Manini TM, Beavers DP, Pahor M, Guralnik JM, Spring B, Church TS, et al. Effect of Physical Activity on Self-Reported Disability in Older Adults: Results from the LIFE Study. J Am Geriatr Soc. 2017;65(5):980-8.
57. Ware JE, Jr., Sherbourne CD. The MOS 36-item short-form health survey (SF-36). I. Conceptual framework and item selection. Medical care. 1992;30(6):473-83.
58. Ross TP. The reliability of cluster and switch scores for the Controlled Oral Word Association Test. Archives of clinical neuropsychology : the official journal of the National Academy of Neuropsychologists. 2003;18(2):153-64.
59. Koss E, Ober BA, Delis DC, Friedland RP. The Stroop color-word test: indicator of dementia severity. The International journal of neuroscience. 1984;24(1):53-61.
60. Shapirol AM, Benedict RHB, Schretlen D, Brandt J. Construct and Concurrent Validity of the Hopkins Verbal Learning Test-Revised. The Clinical Neuropsychologist. 1999;13(3):348-58.
61. Smith A. Symbol Digit Modalities Test (SDMT). Manual (Revised). Western Psychological Services, Los Angeles, 1982.
62. Morris JC, Heyman A, Mohs RC, Hughes JP, van Belle G, Fillenbaum G, et al. The Consortium to Establish a Registry for Alzheimer's Disease (CERAD). Part I. Clinical and neuropsychological assessment of Alzheimer's disease. Neurology. 1989;39(9):1159-65.
63. Lezak MD. Neuropsychological assessment, 3rd ed. New York: Oxford University Press. 1995.
64. Mitrushina M, Boone KB, Razani J, D'Elia LF. Handbook of Normative Data for Neuropsychological Assessment. 2 edn. ed: Oxford University Press Inc.; 2005.
65. Svarstad BL, Chewning BA, Sleath BL, Claesson C. The Brief Medication Questionnaire: a tool for screening patient adherence and barriers to adherence. Patient education and counseling. 1999;37(2):113-24.
66. Morisky DE, Green LW, Levine DM. Concurrent and predictive validity of a self-reported measure of medication adherence. Medical care. 1986;24(1):67-74.
67. Langa KM, Larson EB, Crimmins EM, Faul JD, Levine DA, Kabeto MU, et al. A Comparison of the Prevalence of Dementia in the United States in 2000 and 2012. JAMA internal medicine. 2017;177(1):51-8.
68. Ahmadi-Abhari S, Guzman-Castillo M, Bandosz P, Shipley MJ, Muniz-Terrera G, Singh-Manoux A, et al. Temporal trend in dementia incidence since 2002 and projections for prevalence in England and Wales to 2040: modelling study. Bmj. 2017;358:j2856.
69. Kawas CH, Katzman R. Epidemiology of Dementia and Alzheimer Disease. . Alzheimer Disease. 2nd Ed ed. Philadelphia:: Lippincott Williams & Wilkins; 1999. p. 95-116.

For peer review only


For peer review only - http://bmjopen.bmj.com/site/about/guidelines.xhtml

# A randomised clinical trial of STAtin therapy for Reducing Events in the Elderly (STAREE)

**PROTOCOL**

**Version 3.1**

**APPENDICES 1-11**

For peer review only

## Appendix 1: STAREE hub locations (update July 2019)

| Site location | STATE | HREC | Start Date | Office location |
|---|---|---|---|---|
| Melbourne and surrounds | VIC | MUHREC | 26/08/2014 | Monash University, 553 St Kilda Rd, Melbourne |
| Geelong | VIC | MUHREC | 05/09/2016 | Monash University, 553 St Kilda Rd, Melbourne |
| Mornington | VIC | MUHREC | 14/09/2016 | Monash University, 553 St Kilda Rd, Melbourne |
| Mildura | VIC | MUHREC | 16/01/2017 | School of Rural Health, Monash, Mildura (closed 2018) |
| Bendigo | VIC | MUHREC | 20/03/2017 | School of Rural Health, Monash, Bendigo |
| Gippsland | VIC | MUHREC | 20/03/2017 | Monash University, 553 St Kilda Rd, Melbourne |
| East Gippsland | VIC | MUHREC | 1/4/2019 | Monash University, 553 St Kilda Rd, Melbourne |
| Warrnambool | VIC | MUHREC | 26/04/2018 | Monash University, 553 St Kilda Rd, Melbourne |
| Albury/Wodonga | VIC | MUHREC | 19/04/2018 | Monash University, 553 St Kilda Rd, Melbourne |
| Hobart | TAS | HREC (Tasmania) network | 01/06/2015 | Menzies Institute for Medical research, University of Tasmania, Hobart |
| Burnie | TAS | HREC (Tasmania) network, | 01/01/2017 | Menzies Institute for Medical research, University of Tasmania, Launceston |
| Launceston | TAS | HREC (Tasmania) network | 01/01/2017 | Menzies Institute for Medical research, University of Tasmania, Launceston |
| Perth and regional WA | WA | HREC (Curtin), | 16/06/2015 | Curtin University, Perth |
| Newcastle | NSW | HREC (Uni Newcastle) | 11/09/2017 | University of Newcastle, Newcastle |
| Tamworth | NSW | HREC (Uni Newcastle) | 28/05/2018 | University of Newcastle, Newcastle |
| Port Macquarie | NSW | HREC (Uni Newcastle) | 22/07/2019 | University of Newcastle, Newcastle |
| Sydney | NSW | HREC (Uni Newcastle) | 21/05/2018 | University of Newcastle, Newcastle |
| Brisbane | Qld | MUHREC | 17/07/2017 | Corporate House, Greenslopes, Brisbane |
| Sunshine coast/Hervey Bay | Qld | MUHREC | 17/07/2017 | Corporate House, Greenslopes, Brisbane |
| Gold Coast | Qld | MUHREC | 17/07/2017 | Corporate House, Greenslopes, Brisbane |
| Toowoomba | Qld | MUHREC | 17/07/2017 | Corporate House, Greenslopes, Brisbane |
| Adelaide | SA | MUHREC | 24/04/2018 | Victoria Park, Dulwich, Adelaide |

For peer review only

## Appendix 2: Brief definitions of STAREE endpoints

| **PRIMARY ENDPOINTS** | |
|---|---|
| Death, Dementia and Disability | Survival free of dementia or persistent physical disability (as derived from the endpoints of all-cause mortality, dementia and persistent physical disability) |
| Major Adverse Cardiovascular Event (MACE) | Major Adverse Cardiac Event (MACE); cardiovascular death or acute and non-fatal myocardial infarction (MI) or stroke. |
| **SECONDARY ENDPOINTS** | |
| All-cause mortality | Any cause for death; will be classified based on primary cause such as stroke, MI, heart failure, cancer or other.<br><br>(All sudden deaths are presumed to be caused by a major cardiovascular event unless confirmed otherwise). |
| Cardiovascular death | Death resulting from a cardiovascular event (i.e. MI, stroke, heart failure). |
| Myocardial Infarction | Non-fatal: Evidence of myocardial necrosis in a clinical setting consistent with myocardial ischemia<br>Fatal: Death resulting from MI |
| Stroke | Fatal: Death resulting from stroke<br><br>Non-fatal: Acute episode of focal or global neurological dysfunction lasting more than 24 hours (unless interrupted by surgery or death) with no apparent cause other than of vascular origin. Imaging is required for classification.<br><br>OR |

For peer review only

| | |
|---|---|
| | Acute episode of focal or global neurological dysfunction lasting less than 24 hours and with no apparent cause other than of vascular origin AND imaging supporting stroke diagnosis. |
| Persistent Physical Disability | Persistent disability or loss of physical function as reflected on any one of the Life Ability ADL items (Q9-14) as either "a lot of difficulty" or "unable to do this activity" and/or "Yes" to receiving help, across 2 or more records (> 6 months duration).<br><br>OR<br><br>If it is not possible to obtain a Life Ability ADL assessment, relevant clinical records such as notification of admission to aged care or community assessment. |
| Dementia | Criteria-based: Acquired impairment in cognitive function that is significant (≥2 SD decline below population norms) and interferes with independence in everyday activities. [1]<br><br>Record-based: Credible diagnosis of dementia on medical record, without any supporting cognitive data available. |
| Other Cognitive Impairment | All other instances of cognitive impairment (not meeting criteria for "Dementia" above). |
| Approval for permanent residential care | Approval for permanent residential care (based on reporting by an Aged Care Assessment Team). |
| Diagnosis requiring hospitalisation | Hospitalisation extending more than 24 hours or across a minimum of two calendar days. Excludes elective procedures. |
| Heart Failure | A heart failure event, treated in the emergency room or being admitted to hospital and defined as including at least one criterion for heart failure symptoms, at least two for heart failure signs, and imaging or biomarker evidence. |
| Atrial Fibrillation | Verified: Any ECG rhythm strip showing AFib verified by a medical professional |

For peer review only

| | |
|---|---|
| | OR<br><br>Medical professional diagnosed AFib<br><br>OR<br><br>Procedures undertaken for AFib (e.g. cardio version, ablation)<br><br>OR<br><br>Possible: Irregular heart rhythm during heart rate measurement (e.g. irregular pulse on BP measure )<br><br>AND<br><br>Taking medication used to manage AF (oral anticoagulant agents without other indication (ADD ATC CODE), cardiac glycosides (ATC-Code C01A), antiarrhythmics, Class I and III (ATC-Code C01B)) |
| Hospitalisation for Arterial Revascularisation Procedures | Treatment or surgical intervention to relieve symptoms associated with coronary, arterial disease. Coronary, cerebral or peripheral revascularisation procedures, including percutaneous coronary intervention (PCI), coronary artery bypass graft surgery(CABG), carotid endarterectomy or carotid bypass, other arterial surgery (with or without stenting), other arterial surgery (aortic aneurysm repair, aortobifemoral bypass, femoropopliteal bypass, femoral-tibial bypass and cerebral aneurysm repair) |
| Fatal and non-fatal cancer | Malignant neoplasm confirmed by histopathology or imaging (non-melanoma skin cancers excluded). |
| Quality of life | Determined by the score (0-100) in each of the 8 domains of the SF-36; physical functioning, role limitations due to physical problems, bodily pain, general health perceptions, vitality, social functioning, role-limitations due to emotional problems, and mental health [56]. Based on population norms, a score of 50 is average, 10 point variation is one standard deviation (15 points = 1.5SD). Summary score collapses domains into two scores – age bracket specific |
| Cost-effectiveness of statin | To be derived from health economic analysis |

For peer review only

| TERTIARY ENDPOINTS | |
|---|---|
| Frailty Phenotype | Frailty phenotype will be measured using an approach based on the simplified Women's Health Initiative.[2] |
| **ADVERSE EVENTS OF INTEREST** | **BRIEF DEFINITION** |
| New diabetes | Diabetes as diagnosed on the basis of:<br>1) an annual HbA1c or fasting plasma glucose test using the WHO criteria (a HbA1c value of 6.5% or higher or a fasting plasma glucose of 7.0 mmol/L or higher, with confirmation by a second test within 4 weeks; or<br>2) community-based testing during usual clinical care (GP or other clinicians e.g. hospitalisation with a hyperglycaemic crisis or classic symptoms of hyperglycaemia and a random plasma glucose test of 11.1 mmol/L and/or commencement of glucose lowering medication for glucose control). |
| Myopathy | Participant description of any muscle symptoms including type, duration and effect of exercise. |
| Liver impairment | Elevated ALT and/or AST across two or more tests (elevations greater than 3x ULN and elevated bilirubin (2x ULN)) and no other demonstrable cause |

1. American Psychiatric Association. (2022). Neurocognitive Disorders. In *Diagnostic and statistical manual of mental disorders* (5th ed., text rev.).

2. Zaslavsky, Oleg, et al. "Comparison of the simplified sWHI and the standard CHS frailty phenotypes for prediction of mortality, incident falls, and hip fractures in older women." *Journals of Gerontology Series A: Biomedical Sciences and Medical Sciences* 72.10 (2017): 1394-1400.

For further information see also supplement document *Endpoint Definitions*



For peer review only

## Appendix 3 : Cytochrome P4503 A4 inhibitors clinical recommendations

(updated November 2020)

| CYP450 3A4 Inhibitors | | | | |
|---|---|---|---|---|
| **Brand Name** | **Generic Name** | **Indication** | **Drug Class** | **Study Recommendations** |
| Amiodarone<br>Aratac<br>Cardinorm<br>Cordarone<br>Rithmik | Amiodarone | Angina/Arrhythmia | Antiarrhythmic agent | **Limit study medication to one tablet (20 mg) for the duration of amiodarone therapy.** |
| Magicul<br>Tagamet | Cimetidine | Gastroesophageal reflux disease | Histamine receptor antagonist | **EXCLUDE/ withhold study medication for the duration of cimetidine therapy** |
| Cyclosporin<br>Neoral | Cyclosporin | Transplant rejection, rheumatoid arthritis | Immunosuppresant | **EXCLUDE/ withhold study medication for the duration of cyclosporin therapy** |
| Azol | Danazol | Endometriosis | Gonadotropin Inhibitor | **EXCLUDE/ withhold study medication for the duration of danazol therapy** |
| Cardizem<br>Coras<br>Diltahexal<br>Diltiazem<br>Dilzem<br>Vasocardol | Diltiazem | Angina/Hypertension | $Ca^{+}$ Channel Blocker | **Limit study medication to one tablet (20 mg) for the duration of diltiazem therapy.** |
| Telzir | Fosamprenavir | HIV | Protease Inhibitor | **EXCLUDE** |
| Kaletra | Lopinavir/ Ritonavir | HIV/AIDS | Protease Inhibitor | **EXCLUDE** |
| Anpec<br>Cordilox<br>Isoptin<br>Tarka<br>Veracaps | Verapamil | Angina/Hypertension/Arrhythmia | Ca+ Channel Blocker | **Limit study medication to one tablet (20 mg) for the duration of verapamil therapy.** |
| **CYP450 3A4 Inhibitors** | | | | |

For peer review only

| Brand Name | Generic Name | Indication | Drug Class | Study Recommendations |
|---|---|---|---|---|
| Norvir | Ritonavir | HIV/AIDS | Protease inhibitor and potent CYP3A4 inhibitor | **EXCLUDE** |
| Included in: Prezcobix Evotaz Stribild Genvoya Tybost | Cobicistat | HIV/AIDS | Potent CYP3A4 inhibitor | **EXCLUDE** |
| Maviret | Glecaprevir/ Pibrentasvir | Hepatitis C | Protease inhibitor | **EXCLUDE/ withhold study drug for the duration of protease inhibitor therapy (usually 12 weeks)** |
| Vosevi | Sofosbuvir/ Velpatasvir/ Voxilaprevir | Hepatitis C | Protease inhibitor | **EXCLUDE/ withhold study drug for the duration of protease inhibitor therapy (usually 12 weeks)** |
| Prevymis | Letermovir | Cytomegalovirus (CMV) prevention | CMV DNA terminase complex inhibitor | **EXCLUDE/ withhold study drug for the duration of letermovir therapy** |

| **Antibiotics and Antifungals for Long Term Oral Use Only** | | | | |
|---|---|---|---|---|
| **Brand Name** | **Generic Name** | **Indication** | **Drug Class** | **Study Recommendations** |
| Clarac Clarihexal Clarithro Clarithromycin Kalixocin Klacid Nexium Hp7 Combination pack | Clarithromycin | Bacterial infection | Macrolide antibiotic | **Limit study medication to one tablet (20 mg) for the duration of clarithromycin therapy.** |
| EES E-mycin Eryc Erythromycin | Erythromycin | Bacterial infection | Macrolide antibiotic | **Limit study medication to one tablet (20 mg) for the duration of erythromycin therapy.** |

For peer review only

| | | | | |
|---|---|---|---|---|
| Diflucan<br>Fluconazole | Fluconazole | Fungal infection | Antifungal | **Limit study medication to one tablet (20 mg) for the duration of fluconazole therapy.** |
| Lozanoc<br>Sporanox | Itraconazole | Fungal infection | Antifungal | **EXCLUDE/ withhold study medication for the duration of itraconazole therapy** |
| Nizora | Ketoconazole | Fungal infection | Antifungal | **EXCLUDE/ withhold study medication for the duration of ketoconazole therapy** |
| Fucidin | Fusidic acid or sodium fusidate | Bacterial infection | Antibiotic | **EXCLUDE/ withhold study medication for the duration of and one week after completion of fusidic acid therapy** |

1. www.drugbank.ca
2. www.medicines.org.uk
3. www.drugs.com/pro/atorvastatin.html
4. www.medsafe.govt.nz
5. Stockley's Drug Interactions

For peer review only

## Appendix 4: Medication compliance questions

1. Have you been taking study medication? Yes / No
2. If Yes, how may study tablets do you take each day? 1 tablet / 2 tablets / other___
3. In an average week, how many days do you take study medication? _____ days
4. Do you ever miss taking your study medications? Yes / No
5. Comments (i.e. reasons for occasionally missing or altering dose) ________

OR, if they answer NO to question 1, then:

2. What reason(s) did you stop taking study medication? ______________
3. What date did you stop taking study medication?
4. Who made this decision? GP or PCP / Self / Other

For peer review only

## Appendix 5: Reporting of AEs and SAEs

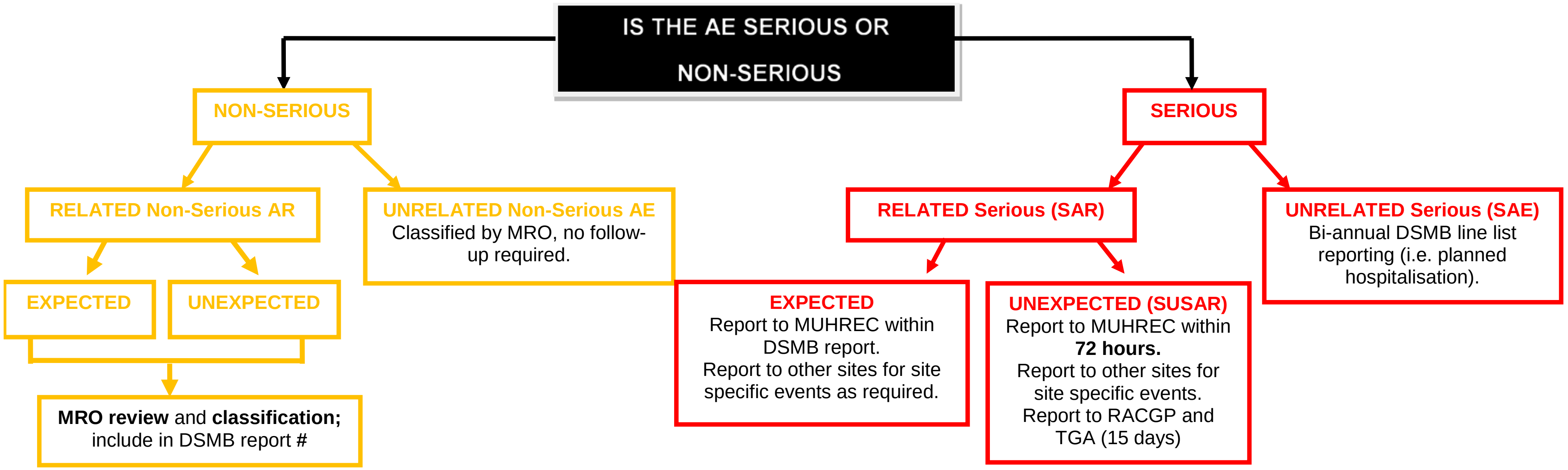


**Whether an AE is related/unrelated and expected/unexpected** is based on the current TGA Product Information sheet (dated 19/6/13) reference to the list of potential ADRs will be conducted by the study medical officer and documented under the categories above.

If SAR deemed related to study medication, classification of likelihood is made (**POSSIBLE / PROBABLE / DEFINITE)** based on Naranjo ADR probability scale

| | |
|---|---|
| **AE** Adverse Event | Any unfavourable medical occurrence |
| **SAE** Serious Adverse Event | An event that:<br>❖ requires hospitalisation/prolongation of existing hospitalisation<br>❖ results in death<br>❖ is life threatening<br>❖ results in persistent or significant disability/incapacity<br>❖ is a congenital anomaly/birth defect; or<br>❖ is a medically important event or reaction |
| **SAR** Serious Adverse Drug Reaction | A SAE that is deemed related to study medication (note serious ADR also used; also non-serious ADR) |
| **SUSAR** Serious, Unexpected, Suspected Adverse Reaction | An **unexpected** SAE where there is **some degree of probability** that the event is related to the study medication. |
| **PI** Principal Investigator | Principal Investigator or a co-investigator delegated this responsibility |
| **Comment from the PI** | A statement about the significance of the information, the possible impact on study participants, and action taken or recommended |

***DSMB report: submitted six-monthly: includes line list of non-serious AEs of interest # (muscle symptoms, changes to memory) and summary of related SAEs, both expected and unexpected; the former based on the current Product Information sheet; Reports from DSMB to be forwarded to all committees & ADR select section to TGA***

For peer review only

## Appendix 6: Assessment tools

| Outcome Measure | Tool (abbreviation) | References |
|---|---|---|
| Global cognitive function | Modified Mini-Mental State Examination (3MS) | Tombaugh, T.N. et al. (2005). Test-retest reliable coefficients and 5-year change scores for the MMSE and 3MS. *Archives of Clinical Neuropsychology*, 20, 485-503<br><br>Ryan, J., et al. (2019). Normative performance of healthy older individuals on the Modified Mini-Mental State (3MS) examination according to ethno-racial group, gender, age, and education level. *The Clinical Neuropsychologist*, 33(4), 779-797. |
| Verbal episodic memory | Hopkins Verbal Learning Test – Revised (HVLT-R) | Shapirol, A.M., et al. (1999). Construct and Concurrent Validity of the Hopkins Verbal Learning Test-Revised. *The Clinical Neuropsychologist,* 13(3), 348-358<br><br>Ryan, J., et al. (2021). Normative performance of older individuals on the Hopkins Verbal Learning Test-Revised (HVLT-R) according to ethno-racial group, gender, age and education level. *The Clinical Neuropsychologist*, 35(6), 1174-1190 |
| Verbal fluency, language | Controlled Oral Word Association Test (COWAT) | Ross, T.P., 2003. The reliability of cluster and switch scores for the Controlled Oral Word Association Test. *Archives of Clinical Neuropsychology*, 18(2), 153-164<br><br>Ravdin, L.D., et al. (2003). Letter and semantic fluency in older adults: effects of mild depressive symptoms and age-stratified normative data. *The Clinical Neuropsychologist*, 17(2), 195-202 |
| Visual agnosia | Lurian Overlapping Figures (Lurian) | Luria, A.R. (1966). Higher Cortical Functions in Man. New York, NY: Basic Books.<br><br>Reid, W., et al. (1996). Age at onset and pattern of neuropsychological impairment in mild early-stage Alzheimer disease: a study of a community-based population. *Archives of Neurology*, 53(10), 1056-1061 |
| Executive function | Victoria Stroop Colour-Word Test (Stroop) | Koss, E., et al. (1984). The Stroop color-word: Indicator of dementia severity. *International Journal of* Neuroscience, 24(1), 53-61<br><br>Troyer, A.K., et al. (2006). Aging and response inhibition: Normative data for the Victoria Stroop Test. *Aging, Neuropsychology, and Cognition*, 13(1), 20-25 |

For peer review only

| | | |
|---|---|---|
| Executive function | Trail Making Test (TMT) | Corrigan, J.D., & Hinkeldey, M.S. (1987). Relationships between Parts A and B of the Trail Making Test. *Journal of Clinical Psychology,* 43, 402-409<br><br>Mitrushina, M., et al. (2005). Handbook of Normative Data for Neuropsychological Assessment, Appendix 4M, Oxford, UK: Oxford University Press |
| Processing speed | Symbol Digit Modalities Test (SDMT) | Smith, A. Symbol Digit Modalities Test (SDMT). Manual (Revised) (1982). Los Angeles, CA: Western Psychological Services.<br><br>Kiely, K.M., et al. (2014). Butterworth, P., Watson, N. & Wooden, M. The Symbol Digit Modalities Test: Normative data from a large nationally representative sample of Australians. *Archives of Clinical Neuropsychology,* 29, 767-775 |
| Constructional praxis | Consortium to Establish a Registry for Alzheimer's Disease (CERAD) Neuropsychological Battery; Constructional Praxis Subscale (constructional praxis) | Morris, J.C. et al. (1989). The Consortium to Establish a Registry for Alzheimer's Disease (CERAD). Part I. Clinical and neuropsychological assessment of Alzheimer's disease. *Neurology,* 39, 1159-1165.<br><br>Welsh, K.A., et al. (1994). The Consortium to Establish a Registry for Alzheimer's Disease (CERAD). Part V. A normative study of the neuropsychological battery. *Neurology*, 44, 609-614. |

For peer review only

# PARTICIPANT CONSENT FORM

*MONASH UNIVERSITY*

## Appendix 7: STAREE participant information sheet/consent form

For peer review only


For peer review only - http://bmjopen.bmj.com/site/about/guidelines.xhtml

# PARTICIPANT CONSENT FORM

*MONASH UNIVERSITY*

For peer review only


For peer review only - http://bmjopen.bmj.com/site/about/guidelines.xhtml

# Participant Consent Form

*Monash University*

# Participant Information Sheet/Consent Form

*Monash University*

**Full Project Title:** A randomised clinical trial of STAtin therapy for Reducing Events in the Elderly (STAREE)

This participant information and consent form is 8 pages long. Please make sure you have all the pages.

This document tells you about the research project. It explains the tests and treatments involved. Knowing what is involved will help you decide if you want to take part.

Please read this information carefully. Ask questions about anything that you do not understand or want to know more about. Before deciding whether or not to take part, you may want to talk about it with a relative, friend or your local doctor.

If you decide to take part in the research project, you will be asked to sign the consent section. By signing it you are telling us that you:

- Have read the participant information sheet and consent form;
- Understand what you have read;
- Agree to take part in the research project;
- Agree to take the study medication as instructed for an average of 5 years;
- Agree to undergo the study assessments as described in this document;
- Agree to attend clinic visits annually;
- Agree to your medical records being accessed; and
- Agree to having your data accessed via data linkage (bringing together information from different sources such as registries, hospitals etc.).

You will be given a copy of this Participation Information and Consent Form to keep.

For any queries, information on the project or to speak to the research team please call **1800 770 664**.

## Why are you being given this Participant Information and Consent Form?

We would like to invite you to participate in this research project as you are aged 70 or older and do not have current serious disease or disability.

## Who is running this study?

The study is run by the School of Public Health and Preventive Medicine, Monash University. Your general practitioner (GP) may be a co-investigator and may receive up to $100 per patient for the use of their practice facilities.

## What is the study about?

Statins (HMG-CoA reductase inhibitors) are a class of drug that block the action of a chemical (HMG-CoA reductase) in the liver that is needed to make cholesterol. This


For peer review only - http://bmjopen.bmj.com/site/about/guidelines.xhtml

# PARTICIPANT CONSENT FORM

*MONASH UNIVERSITY*

action lowers cholesterol and lipid levels in the blood. The health benefits of statins may include reducing the number of new cases of heart disease, stroke and dementia. However, there are side-effects from statins that are still being debated. We are investigating if the benefits of statins outweigh the risks in healthy people aged 70 and over. We aim to determine if taking statins prevents a decline in the physical and mental abilities associated with ageing which may lead to long term residential care and a loss of independence.

STAREE will be conducted in general practices across Australia and aims to recruit at least 10,000 participants nationally. Half of the participants will receive statin therapy and half will receive a placebo tablet (tablet with inactive ingredients). We will then observe the 2 groups over an average 5 year period to see what differences there are for levels of physical disability, death, heart attacks, diabetes, dementia and general quality of life. We will collect this data from you, your GP's records, and any hospital or specialist you visit.

You will also be asked to fill out a separate consent form authorising access to your complete Medicare, Pharmaceutical Benefits Scheme (PBS), Repatriation Pharmaceutical Benefits Scheme (RPBS) data and various health data sets. Medicare collects information on your doctor visits and the associated costs, while the PBS/RPBS collects information on the prescription medications you have filled at pharmacies. This consent form is sent securely to the Department of Human Services which holds this information confidentially. We also ask for your consent to access other health data information such as hospital discharge summaries and national registry health information (i.e. National Death Index).

Providing us with this information helps us to assess your health status throughout the study.

#### WHAT ARE WE ASKING YOU TO DO?

**Baseline Visit 1**: If you agree to participate, you will be asked to give your consent by signing this form. After you give your consent to enter this study, research staff will briefly look at your medical records to ensure there are no underlying medical conditions that would make you ineligible for the study, and to check any pathology results from the previous 6 months to ensure no tests are repeated unnecessarily. We will then measure your blood pressure, heart rate and ask you some questions about your general health and medical history, your family medical history, current medications, and your daily activities. We will ask you to complete other questionnaires that will assess your mood and involve some thinking tasks. You will have a brief physical examination including measuring your weight, height, waist and hip circumference.

We will then ask you to take a placebo tablet of study medication once daily for four weeks. This will help get you in the routine of taking a tablet every day.

The research staff will also ask you to attend a local pathology service for a fasting blood test and a urine test. Approximately 12 ml (about 2 teaspoons) of blood will be taken and this sample will be used to check your cholesterol, liver and kidney function and sugar levels. Approximately 20 ml of urine will also be collected. This will provide information


For peer review only - http://bmjopen.bmj.com/site/about/guidelines.xhtml

# PARTICIPANT CONSENT FORM

*MONASH UNIVERSITY*

about your kidney function and cardiovascular health.

Based on these test results, the physical examination and your medical and medication history, we will ask you to see your GP to discuss if it is appropriate for you to continue in the study and attend baseline visit 2.

**Baseline Visit 2**: At the second visit, if you have taken your study medication, and you satisfy all other eligibility criteria, you will be randomly assigned to take two tablets of either statin (atorvastatin 40 mg) or placebo (a dummy tablet), at the same time every day with a drink of water for an average of 5 years. You will need to commit to taking the study medication regularly as well as attend all the study visits.

The medication you receive will be determined by a computer program. Neither you, research staff, nor your GP will know if you are taking the active (statin) or placebo medication. You will have equal chance of receiving either statin or placebo.

In the event of an emergency, any doctor can find out what treatment you are taking if they need to know.

During this visit, you will be asked to complete tasks that measure thinking and memory function and a questionnaire on your health and physical ability.

**Annual clinic visit:** Each year, you will be asked to attend a study visit or a phone call where staff will conduct similar activities as in your baseline visits. You will be asked to attend this visit, even if you stop taking the study medication.

**6 monthly phone call**: Every six months the research staff will phone you and ask you about your recent medical history, medications and how you are going on the trial.

### CAN I LEAVE THE STUDY?

Participation in any research project is voluntary. If you do not wish to take part, you do not have to. If you decide to take part and later change your mind, you can change your level of involvement with the project at any stage.

While full participation in the study means that you are taking the study medication and having study visits and phone calls, there are other levels of participation that you may consider. Stopping or discontinuing study medication (even for a short time period) does not mean you have to stop your involvement in the trial.

The others levels of involvement are:

- Stopping your study medication but still attending study visits and receiving phone calls
- Stopping your study medication and also stopping study visits and phone calls
- Stopping your study medication, stopping study visits and phone calls and stopping collection of data from your medical records

Please note, if you decide to not continue your participation in the study, the data that has been collected up to this point will remain part of the study data. It is important that this data remains in the study because if it is excluded the safety of the research project may be compromised. Retention of this data is also important to ensure appropriate reporting to the study Data and Safety Monitoring Board.



For peer review only - http://bmjopen.bmj.com/site/about/guidelines.xhtml

# PARTICIPANT CONSENT FORM

*MONASH UNIVERSITY*

StaREE
STAtins in Reducing Events in the Elderly

If you do decide to stop your involvement from the study, we may ask you about your reasons. This information is important for the conduct of future trials.

Regardless of your level of participation or if you stop your involvement completely from the study, it will not affect your routine treatment, your relationship with your GP or your relationship with Monash University.

**WHAT ARE THE POSSIBLE BENEFITS OF TAKING PART?**

We cannot guarantee that you will receive any health benefits from this research. However, some participants may experience health benefits from being placed on atorvastatin. These benefits may include the following:

- Reductions in the levels of cholesterol in the blood,
- Improved health of blood vessels throughout the body,
- A reduced risk of cardiovascular disease (e.g.) heart attack or stroke).

In addition, some scientific studies have suggested that statins may be associated with reduced risk of dementia, depression, and cancer. However, these effects are less certain.

If the study shows that there are net health benefits from statin therapy, it may enable this treatment to be available to more people in the future. We will also inform your doctor of any abnormal findings from your measurements, and memory and thinking tasks, so that they can discuss this with you.

**WHAT ARE THE POSSIBLE RISKS AND DISADVANTAGES OF TAKING PART?**

Atorvastatin, the statin used in this study, is an established safe and well-tolerated medication to lower cholesterol. However, like all medications, atorvastatin can cause side-effects. These side-effects do not affect everyone. You may experience no side effects. If you do experience side effects from the study medication they may be mild, moderate or severe. Many side effects are temporary or reversible, however, some side effects can be serious, long lasting or permanent. If this occurs, your doctor may ask you to stop taking the medication.

You could experience side effects that researchers do not expect or do not know about and these side effects could be serious.

Tell your doctor immediately about any new or unusual symptoms that you experience. Your doctor will monitor any side effects that do occur and will discuss the best way of managing them with you.

The reported side effects of atorvastatin:

- Common side effects (occur in 1-10% of patients): headache, muscle pain, constipation, feeling sick and increase in blood sugar levels.
- Uncommon side effects (0.1-1%): skin rash, itching and hives.
- Rare side effects (0.01-0.1%): hypersensitivity (allergic) reactions and severe muscle disorders (myopathy).

You may also refer to the Product Information Sheet provided to you at your first study visit.


For peer review only - http://bmjopen.bmj.com/site/about/guidelines.xhtml

# PARTICIPANT CONSENT FORM

*MONASH UNIVERSITY*

You should not drink more than one or two small glasses of grapefruit juice each day as large quantities of grapefruit juice can change the effects of atorvastatin. You should also avoid consuming excessive amounts of alcohol on a regular basis while taking the study medication.

If you suffer any injuries or complications as a result of this research project, you should contact the study team as soon as possible so they can assist with arranging appropriate medical treatment. If you are eligible for Medicare, you can receive any medical treatment required, free of charge, as a public patient in any Australian public hospital.

### WHAT IF NEW INFORMATION ARISES DURING THE RESEARCH PROJECT?

Sometimes during the course of a research project, new information becomes available about the treatment being studied. If this happens, your doctor will tell you about it and discuss with you whether you want to continue in the research project. If you decide to withdraw, your doctor will continue with your regular health care. If you decide to continue in the research project you will be asked to sign an updated consent form.

Also, on receiving new information, your doctor may consider it to be in your best interest to withdraw you from the research project. If this happens they will explain the reasons and arrange for your regular health care to continue.

### WHAT WILL HAPPEN TO INFORMATION ABOUT ME?

Any information collected in this study will not be published in any manner that could identify you as an individual, during or after the conclusion of this project. We will only publish group data. Your personal and health information which we will collect as part of this study is reported on special forms by the research staff. Your name is not recorded on these forms. You are only identified by your initials and a study-specific number assigned by research staff.

By signing the attached consent form, you are agreeing to the release of your medical records held by your doctor or hospital. This information will be collected, stored and analysed only for the purposes of this study, and will be limited to the medical details related to this study. Any information collected from your clinic about you will be overseen by the clinic GPs.

Medical tests and results collected as part of the study may reveal an underlying condition. Any results outside the normal range (such as elevated blood pressure or abnormal pathology) will be communicated to your GP.

Final results of the project and the arm of the trial you were part of, will be communicated to you at the conclusion of the trial.

Monash University implements a defence in-depth approach (multiple layers of security) to information security and employs a multitude of controls to protect our infrastructure and data. These controls are regularly audited to ensure they meet global best practices and are aligned with ISO 27001 security practices. Data collected as part of the STAREE clinical trial will be stored on University-managed, secure and resilient infrastructure located in Australia that complies with all applicable data protection and privacy obligations. Some specialist data collected


For peer review only - http://bmjopen.bmj.com/site/about/guidelines.xhtml

# Participant Consent Form

*Monash University*

as part of the STAREE clinical trial may be entered into applications that are operated by external third parties. This will only be permitted where all applicable data protection and privacy obligations are in place with those external third parties.

All information collected for this project will be retained for a period of no less than 15 years following the completion of the project. Hardcopy information will be shredded and destroyed and electronic data will be deleted from the secure STAREE database after a period of no less than 15 years after the completion of the project, in accordance with the Monash University Human Research Ethics Committee recommendations for drug trials and Good Clinical Practice Guidelines.

### Who is funding the research?

The main funding body of STAREE is the National Health and Medical Research Council of Australia.

### Who has reviewed the research project?

All research in Australia involving humans is reviewed by an independent group of people called a Human Research Ethics Committee (HREC). The ethical aspects of this project have been approved by the Monash University Human Research Ethics Committee (MUHREC) and The Royal Australian College of General Practitioners National Research and Evaluation Ethics Committee (NREEC).

This project will be carried out according to the *National Statement on Ethical Conduct in Human Research (2007)*. This statement has been developed to protect the interests of people who agree to participate in human research studies.

### Further information and who to contact

Please ask the research staff member if you have any further questions. You can also consult your own doctor if you have any concerns during the course of the research.

Specific enquiries related to this study can also be made to the following person at STAREE:

Name: Professor Sophia Zoungas
(Monash University):
Phone: +61 3 9903 0711
E-mail: sophia.zoungas@monash.edu

For matters relating to research at the site at which you are participating, or if you have any complaints about any aspect of the project, the way it is being conducted or any questions about being a research participant in general, you may contact the Executive Officer, Monash University Human Research Ethics (MUHREC) quoting project 21528:

Executive Officer
Monash University Human Research
Ethics Committee (MUHREC)
Room 111, Building 3e

Research Office
Monash University VIC 3800
Tel: +61 3 9905 2052
Email: muhrec@monash.edu
Fax: +61 3 9905 3831


For peer review only - http://bmjopen.bmj.com/site/about/guidelines.xhtml

# PARTICIPANT CONSENT FORM

*MONASH UNIVERSITY*

For peer review only


For peer review only - http://bmjopen.bmj.com/site/about/guidelines.xhtml

# PARTICIPANT CONSENT FORM

*MONASH UNIVERSITY*

For peer review only



For peer review only - http://bmjopen.bmj.com/site/about/guidelines.xhtml

# Participant Consent Form

*Monash University*

**Title** A randomised clinical trial of STAtin therapy for Reducing Events in the Elderly (STAREE)

## Declaration by participant

I have read the Participation Information Sheet or someone has read it to me in a language that I understand.

I understand the purposes, procedures and risks of the research described in the project.

I understand I will be asked to take a study medication for the duration of the project, and be contacted on a regular basis by phone or in person.

I give permission for my doctors, other health professionals, hospitals or laboratories to release information to Monash University concerning my health and treatment for the purposes of this project. I understand that such information will remain confidential.

I give permission for information that I provide for this research project to be linked to various health datasets for the purposes of the project's outcomes and future research.

I have had an opportunity to ask questions and I am satisfied with the answer I have received.

I freely agree to participate in this research project as described and understand that I am free to change my level of participation or withdraw at any time during the study without affecting my future health care.

I understand that if I decide to discontinue the study medication, I may be asked to attend follow-up visits to allow collection of information regarding my health status.

I understand that if I choose to withdraw from the project that any data that has been collected prior to my withdrawal will be retained in the study database.

I understand I will be given a signed copy of this document to keep.

Name of participant (please print)________________________________________

Signature____________________________________Date____________________

## Declaration by researcher

I have given a verbal explanation of the research project, its procedures and risks and I believe that the participant has understood that explanation.

Name of researcher (please print)________________________________________

Signature____________________________________Date____________________



For peer review only - http://bmjopen.bmj.com/site/about/guidelines.xhtml

## Appendix 8: List of relevant documents.

- DSMB Charter and Plan
- Endpoint Adjudication Committee Charter
- Monitoring plan
- Register of interests for STAREE Investigators and DSMB members
- STAREE HEART protocol

For peer review only



For peer review only - http://bmjopen.bmj.com/site/about/guidelines.xhtml

# Appendix 9: COVID pandemic response

**As a result of government and workplace restrictions due to the COVID-19 pandemic and to ensure safety of staff and participants, the following changes were made to the STAREE clinical trial following ethics approval:**

13th March 2020: Risk management plan formed and approved by STAREE Executive committee

16th March 2020: Invitation letters to participants suspended

20th March 2020: Face to face visits ceased. GP recruitment suspended.

23rd March 2020: Annual Visit 1 was modified to be conducted as a phone call visit

24th April 2020: Baseline Visit 2 was modified to be conducted as a phone call visit

18th May 2020: Annual Visits 2 and 4 were modified to be conducted as phone call visits

27th May 2020: Resumed sending invitation letters to participants. Baseline Consent visit (BV-C) instituted as a phone/zoom call.

July 2020: Physically distanced face to face BV1 visits (SD-BV1) commenced (phone visits offered as alternative if participant preference).

**Changes to the schedule:**

To provide a physically distanced or phone visit the following modifications were made to the visit schedule:

Physically distanced BV1 (SD-BV1): The consent procedure was moved to a separate phone/zoom visit called Baseline Consent (BV-C). Some sections of the usual Baseline 1 visit were moved to the Baseline 2 visit.

3MS: to be completed in full during in-person SD-BV1 or to a modified version used for phone based BV1s.

SD-BV2: All BV2 visits will be conducted by phone.

Annual Visits: The annual visit schedule has been modified so that all annual visits can be conducted over the phone during the COVID-19 pandemic. Face to face visits will resume in 2023.



For peer review only - http://bmjopen.bmj.com/site/about/guidelines.xhtml

# Appendix 10: STAREE MIND protocol

**STAREE-MIND Imaging Substudy**

PROTOCOL

Version 2.2

For peer review only

Protocol authors: Dr Andrea Curtis, Dr Simone Spark, Dr Ian Harding, Professor Sophia Zoungas

Affiliation: School of Public Health and Preventive Medicine, Monash University

**Contact for public enquiries**

Phone: 1800 770 664

Email: staree@monash.edu

Fax: +61 (3) 9903 0737

**Contact for scientific enquiries**

Professor Sophia Zoungas (Principal Investigator)

Sophia.zoungas@monash.edu

Phone: +61 (3) 9903 0711

Address: Level 5/99 Commercial Rd, Melbourne 3004


For peer review only - http://bmjopen.bmj.com/site/about/guidelines.xhtml

**Recruitment Status:** Recruiting

**Acronyms used in this protocol**:

ABS: Australian Bureau of Statistics

ADR: Adverse Drug Reaction

ADCS-IADL: Alzheimer's Disease Cooperative Study Instrumental Activities of Daily Living

ADAS-Cog: The Alzheimer's Disease Assessment Scale - Cognition

AE: Adverse events

CAM: Confusion Assessment Method

CES-D-10: Centre for Epidemiologic Studies Depression Scale – 10 items

COWAT: Controlled Oral Word Association Test

CRF: Case Report Forms

DMC: Data Management Committee

DOHA: Department of Health and Ageing

DSMB: Data Safety and Monitoring Board

EAC: End-point Adjudication Committee

HVLT-R: Hopkins Verbal Learning Test – Revised

MI: Myocardial Infarction

MRI: Magnetic Resonance Imaging

PET: Positron Emission Tomography

SAE: Serious Adverse Events

SDMT: Symbol Digit Modalities Test

STAREE: STAtin therapy for Reducing Events in the Elderly

3MS: Modified Mini-Mental State test

For peer review only



For peer review only - http://bmjopen.bmj.com/site/about/guidelines.xhtml

For peer review only


For peer review only - http://bmjopen.bmj.com/site/about/guidelines.xhtml

# 1 Introduction

## 1.1 Background

### 6.1.1 *Rationale*

Statins—HMG-CoA reductase inhibitors—are a cornerstone treatment of cardiovascular disease. They decrease circulating low density lipoprotein (LDL) cholesterol levels. This effect underpins their ability to reduce the incidence of coronary heart disease and stroke events and possibly their long-term sequelae which include cardiac failure, disability, cognitive decline and dementia.

There are several different types of statins but the most commonly prescribed is atorvastatin.[1] Statins can be sub-grouped according to their hydrophilic or lipophilic nature. Atorvastatin, lovastatin, and simvastatin are lipophilic. Pravastatin, rosuvastatin, and fluvastatin are hydrophilic. Lipophilic statins are able to cross cell membranes including the blood brain barrier.[2] It has been proposed that lipophilic statins may be more effective in the prevention of cognitive decline and dementia.[3]

***Statins, lipids and dementia***

The main lipid in the brain is cholesterol. Scientific evidence suggests that a diet high in fat and cholesterol is associated with an increased risk of AD.[4] High levels of cholesterol inhibit the release of soluble amyloid precursor protein, leading to overproduction of Aβ protein.[5-7] In vivo models of rabbits fed cholesterol show accumulation of excess Aβ protein, which is cleared with return to a normal diet.[8] An increase in intracranial atherosclerosis has also been associated with an increased risk of developing dementia.[9] Statins may reduce risk of dementia through reductions in atheroma burden and in the accumulation of Aβ protein, the major constituent of the senile plaques that are a principal pathological feature of AD.[10]

***Statins, inflammation and dementia***

Substantial evidence implicates inflammatory processes in the development of vascular dementia and AD. Transgenic mouse AD models show increased inflammatory markers with onset of AD pathology.[11] IL-1β is overexpressed sixfold in AD patients' brains compared with controls, especially near amyloid plaques.[12] Increased inflammatory protein levels have been found in the brains and plasma of dementia patients.[13-15] In human studies, serum pro-inflammatory markers may predict the likelihood of dementia and cognitive decline. For example, elevated C-reactive protein (CRP) levels were related to a significantly increased risk for vascular dementia and AD, with or without cardiovascular disease, in the Honolulu Asia Aging Study.[15] The Rotterdam Study found a relationship between elevated levels of α1 antichymotrypsin, CRP, IL-6, soluble intercellular adhesion molecule, and soluble vascular cell adhesion protein 1, and an increased risk for vascular dementia and AD.[13] Meta-analysis showed significantly higher concentrations of the pro-inflammatory cytokines IL-6, TNF-α, IL-1β, IL-12, and IL-18 in the peripheral blood of AD subjects compared with controls.[16] Such data indicate that inflammation may be a key pathogenic accompaniment of dementia. Statins may reduce risk of dementia by inhibiting the pro-inflammatory response that exacerbates and drives the pathological processes leading to neuronal loss.


For peer review only - http://bmjopen.bmj.com/site/about/guidelines.xhtml

***Proposed mechanisms by which statins inhibit pathognomonic Alzheimer's disease***

In AD, Amyloid-β protein (Aβ) is cleaved from trans-membranous amyloid precursor protein (APP) and secreted extracellularly.[17] Aβ may aggregate to oligomers in the synaptic space and alter ion channels by oxidative stress, leading to an influx of excitatory toxic ions. With iron ($Fe^{2+}$) and copper ($Cu^{2+}$) ions, Aβ oligomers lead to the production of hydrogen peroxide, which directly damages lipid-rich cell membranes. Aβ is internalized after attachment to apolipoprotein E4 (ApoE4) lipoprotein receptors. Intracellularly, Aβ may aggregate to form neuritic plaques which may either activate microglia, leading to inflammation, or form highly active free oxygen radicals that facilitate nitric oxide synthase (NOS). Statins directly inhibit this detrimental cascade by blocking Aβ cleavage from APP, by reducing the cellular uptake of Aβ, in part by the suppression of ApoE secretion, and by inhibiting the expression of inducible NOS which, has anti-inflammatory effects and counteracts microglia activation.[17].

***Statins, endothelial nitric oxide synthase (eNOS) and vascular dementia***

Statins are also proposed to reduce the risk of vascular dementia through their regulation of nitric oxide production and eNOS.[18] eNOS activation exerts a protective effect in ischemic stroke patients, which is partly due to its vasodilatory effects resulting in increased cerebral blood flow.[18]

***Effects of statins on cognition and dementia.***

Most of the data on the effect of statins on cognitive function and development of dementia, both AD and vascular dementia, are from population based studies which suggest a reduction in dementia among statin users (Table 1). A new observational study of older US patients reported that AD risk was reduced by 12-15% amongst those with high statin exposure compared to those with low statin exposure.[24] Furthermore, re-analysis of patient level observational data has reported a 20% lower risk of AD in older statin users, and a slower decline in global cognitive scores in those with an ApoE4/ApoE4 genotype.[25] However, these observational studies are subject to numerous biases, foremost among them the inability to establish causal relationships; confounding by indication and unmeasured factors; self-report of statin use; and survival bias. Whilst such biases can be overcome with an appropriately designed RCT, previous RCTs examining the effect of statins on cognitive function and dementia have not reported significant effects. A 2015 meta-analysis investigating randomised evidence on the impact of statins on cognitive outcomes found no statistically significant effect on a crude overall measure of cognition.[26] However, these findings were limited by inclusion of trials that were too small, too short (with less than 1 year of follow up) and too early (<65 years, where the base rate of cognitive decline is very low and subtle effects may not be measureable) or late in the disease course (disease established and irreversible). Indeed, this meta-analysis could not address either long-term efficacy or safety for neurocognitive outcomes, particularly in older populations, because very few such trials exist.

***Adverse effects of statins on cognitive impairment.***

In 2012, the FDA investigated the US spontaneous reporting database (AERS), the published medical literature (case reports and observational studies), and RCTs with respect to the


For peer review only - http://bmjopen.bmj.com/site/about/guidelines.xhtml

effect of statin therapy on cognition.[27] AERS post-marketing reports described ill-defined memory loss with statin treatment, which was reversible on withdrawal. Time to onset of impairment was highly variable, ranging from 1 day to years after first statin exposure. The investigation did not reveal associations between cognitive impairment and specific statins or statin doses, specific age groups or concomitant medication use. Also, cognitive decline was not associated with fixed or progressive impairment. Nonetheless, in 2012 the FDA modified the statin labelling to include a 'black box' warning for a potential risk of adverse cognitive effects such as reversible memory loss and confusion.[27]

**1.2 Summary of rationale**

Atorvastatin and Rosuvastatin are the two drugs with the highest cost to the Australian government and patients, at greater than $1 billion per year.

Based on absolute cardiovascular risk, for which age is the dominant factor, statin use is expected to increase, especially among those >70 years.

Neurological disorders are the outstanding significant cause of non-fatal burden of disability in this country.[28] By 2023, this disease group will contribute almost half of Australia's disability burden.[28] One of the most pervasive and debilitating of these conditions is dementia (including vascular dementia and Alzheimer disease (AD)). By 2050, the number of Australians living with dementia will triple to around 900,000.[29]

Equipoise around the efficacy and safety of statins in the prevention of cognitive decline and dementia exists. Effects in older people are unknown.

If statins produce even a modest reduction or increase in cognitive decline and dementia, the impact across the whole population and on the cost of care for older people, will be considerable. Identifying such effects requires a very large RCT such as STAREE.

STAREE-MIND aims to add an additional suite of neuroimaging assessments in a subset of participants in the parent STAREE trial, making it possible to identify effects with potentially significant impact.


For peer review only - http://bmjopen.bmj.com/site/about/guidelines.xhtml

## 2 Study MIND Design

STAREE MIND is a substudy nested in the STAREE double blind randomised placebo-controlled trial. STAREE is investigating whether statins can prolong good health and maintain independence amongst older people and is enrolling men and women over 70 years of age who are free from cardiovascular disease, diabetes and dementia. The STAREE protocol is Version 2 dated October 2019.

STAREE MIND will recruit a subset of 50 to 500 STAREE participants before they are randomised to STAREE study drug. STAREE MIND will involve an additional suite of brain imaging in these participants. The imaging will take place at study entry and at 4 years follow-up.

## 3 Objectives

The aims are to conduct an imaging ancillary study in STAREE participants that will:

1. Determine the effect of statin treatment over a period of 4 years on longitudinal measures of brain health, including magnetic resonance imaging (MRI) of grey matter volume, white matter integrity and white matter hyperintensities, cerebral perfusion and metabolism, and iron loading.
2. Determine the relationship between imaging changes in the brain and incident cognitive decline and dementia.

## 4 Study Population

### 4.1 Inclusion criteria

- Men and women aged ≥70 years living independently in the Australian community who are participants in the STAREE RCT.
- Willing and able to provide informed consent and accept the STAREE-MIND study requirements, including attending the Monash Biomedical Imaging Facility (located at Blackburn Rd, Clayton, VIC) or the Herston Imaging Research Facility (located at the Royal Brisbane & Women's Hospital campus, Herston, QLD).

### 4.2 Participant recruitment

STAREE-MIND will recruit participants from among those enrolled in the STAREE RCT. Participants will be provided with the STAREE-MIND PICF and invited to participate in the substudy. Participants will be invited to provide informed consent by signing the PICF prior to any assessments being conducted. For participants who consent to STAREE-MIND, a booking for neuroimaging will be made by STAREE administration staff.

The initial neuroimaging assessment will occur prior to commencing STAREE study drug.

STAREE-MIND imaging procedures will be conducted at the Monash Biomedical Imaging Facility (located at 770 Blackburn Rd, Clayton) and the Herston Imaging Research Facility (located at the Royal Brisbane & Women's Hospital campus, Herston, QLD). Participation in STAREE-MIND will therefore be limited to STAREE participants from Greater Melbourne


For peer review only - http://bmjopen.bmj.com/site/about/guidelines.xhtml

and regional Victoria, or Greater Brisbane and regional Queensland, who are able to travel to one of these locations. Participants will be offered $20 to cover their time and costs incurred for transport to the respective facility.

### 4.3 Contingency plans for participant well-being

The procedures used in STAREE-MIND are not diagnostic. However, MRI scans may incidentally reveal an unexpected brain abnormality. All MRI scans are reviewed by a clinical radiologist, and any abnormality is reported. A clinical radiologist will provide a report on the abnormality, including a recommendation as to whether clinical follow-up is necessary.

Participants will be informed on the PICF that if an abnormality is identified, and determined to require clinical follow-up by the consulting radiologist, a report will be forwarded to the participant's doctor and the participant will be contacted by the research team to indicate that follow-up with their doctor is necessary. In line with duty of care requirements, participants who do not wish to be informed of an abnormality under any circumstances, regardless of severity, will not be enrolled in the study.

### 4.4 Withdrawal of participants from the STAREE-MIND study

Participants will be free to withdraw from the STAREE-MIND study at any time by notifying the STAREE research team. If participants withdraw from STAREE-MIND they will be requested to allow the retention of the personal and health information that has been collected. Withdrawal from STAREE-MIND will not affect participants' continued participation in the STAREE RCT.

## 5 Measurements and Analytical Methods

### 5.1 Schedule of study visits

The STAREE participant timeline is shown in Figure 1. STAREE MIND is a substudy of the STAREE RCT and the neuroimaging assessments will align with the STAREE Measurement and Activity schedule as shown in Table 1.

Each neuroimaging visit will take part during normal office hours, and will require 1 hour of the participant's time on a date convenient to them. The neuroimaging sessions will be undertaken at Monash Biomedical Imaging (770 Blackburn Road, Clayton) or the Herston Imaging Research Facility (located at the Royal Brisbane & Women's Hospital campus, Herston, QLD). Study staff will acquire informed consent. MRI scanning will be performed by a MRI clinical radiographer from Monash Health or Metro North Hospital and Health Service.

### 5.2 Procedures at each Neuroimaging visit

At each STAREE-MIND neuroimaging visit the following steps will be undertaken:

1. The explanatory statement will be reviewed with the participant and written informed consent will be ascertained.

2. Participants will be screened for MRI contra-indications (e.g., pacemaker, implants, etc) by the MRI radiographer.


For peer review only - http://bmjopen.bmj.com/site/about/guidelines.xhtml

3. The participant will be prepared for scanning. This involves lying down on the scanner bed, inserting ear-plugs, and being provided with the 'emergency' buzzer (that can be used to terminate the scan), and being checked for claustrophobia.

4. The participant will be required to remain still for the 45-min scan period while MR measures are acquired.

5. The participant will be offered $20 re-imbursement for travel expenses and time.

**5.3 MRI procedure**

Standard MRI acquisitions will be obtained over no more than 45mins, and will include:

- Structural T1- and T2-weighted images of brain anatomy
- Susceptibility weighted imaging (SWI) for quantitative susceptibility mapping
- Diffusion-weighted imaging (DWI) of brain white matter microstructure
- Arterial Spin Labelling (ASL) of brain perfusion
- Phase-contrast angiography of carotid blood flow

All acquisitions are non-invasive and participants remain passive throughout, with no required responses or inputs.

For peer review only



For peer review only - http://bmjopen.bmj.com/site/about/guidelines.xhtml

Figure 1: STAREE and STAREE-MIND (in red) Participant Timeline (UPDATED)

**Call**

- Particpant calls study centre
- Participant screened

**Baseline Visits - STAREE**

- STAREE tria informed consent obtained
- Questionnaires and baseline assessments
- Review inclusion/excusion criteria

**Randomisation**

- Participants randomised to placebo or active statin
- Participants asked if they wish to participate in the STAREE-MIND substudy
- Participants consented to STAREE-MIND substudy
- Participants to attend STAREE-MIND neuroimaging assessment

**Post randomisation and STAREE-MIND visit**

- Participants posted STAREE study drug (continued annually)
- Participants to take 1 tablet of study drug

**Call 4 weeks after randomisation**

- Participants to increase dosage to 2 tablets of study drug

**Annually**

- Participants will be phoned annually at 6, 18, 30, 42 and 54 months

**Annually**

- Participants will attend an annual visit at 12, 24, 36, 48 and 60 months in person or by phone


For peer review only - http://bmjopen.bmj.com/site/about/guidelines.xhtml

**Table 1: STAREE measurement and study activity schedule**. X indicates that all measures carried out, except if a superscript is present, indicating only those tests are performed at that time point. (UPDATED)

| | Screening | Baseline visits | | | | Follow-up visits |
|---|---|---|---|---|---|---|
| Measurement/Activity | Screening call | Baseline Visit 1 | GP visit | Baseline Visit 2 | Post-randomisation | Follow-up (year 4) |
| **Type of visit** | phone | clinic | clinic | clinic | phone | Phone or clinic |
| **Month in trial (week)** | 0 | 0 | 0 | 0 | (0-4) | 48 |
| **Review inclusion/Exclusion criteria** | X | X | X | X | | |
| **Obtain informed consent** | | X | | | | |
| **Dispense study medication** | | X (run-in med only) | | Post (post randomisation) | | X |
| **Medication titration** | | | | | X | |
| **Assess medication compliance** | | | | X | | X |
| **Concomitant medications** | | X | | | | X |
| **Blood pressure & heart rate/rhythm[a], height[c], weight[b], waist:hip circumference[b]** | | X[a,b,c] | | | | As per STAREE protocol |
| **Demographics, family[1] & personal history & lifestyle factors, physical function** | | X[1] | | | | X |
| **Routine Laboratory testing, GP referral to local pathology**<br><br>Fasting bloods: total cholesterol[1], HDL[1], LDL[1], triglyceride[1], CK[2], glucose[2], Hb[2] creatinine (eGFR)[3], ALT[3], AST[3], HbA1c[3],<br><br>Urine: albumin:creatinine ratio[3] | | X[1,2,3] | | | | X[3] |
| **Quality of life**<br><br>-SF-36 | | | | X | | X |
| **Assess cognitive function** | | X | | | | As per STAREE protocol |



For peer review only - http://bmjopen.bmj.com/site/about/guidelines.xhtml

For peer review only

| | | | | | | |
|---|---|---|---|---|---|---|
| - 3MS (and CES-D 10 to exclude depression); CAM and ADCS-IADL as required | | | | | | |
| **Detailed cognitive tests**<br><br>- COWAT, Stroop, HVLT-R, SDMT, Constructional praxis, Lurian overlapping figures, Trail Making Test | | As per STAREE protocol | | As per STAREE protocol | | As per STAREE protocol |
| **Assess physical disability**<br><br>- Life Ability ADLs | | X | | | | X |
| **Biobank samples collected** | | | | X | | |
| **Clinical event reporting**<br><br>- Questionnaire & medical report review | | | | | | X |
| **STAREE-MIND substudy neuroimaging (MRI)*** | | | | | X | X |

*STAREE-MIND neuroimaging will be conducted at entry to the STAREE RCT (between Baseline visit 2 and Randomisation). All neuroimaging assessments will be conducted at the Monash Biomedical Imaging Facility (located at Blackburn Rd, Clayton) or the Herston Imaging Research Facility (located at the Royal Brisbane & Women's Hospital campus, Herston, QLD). Follow-up visits will take place in year 4.

For peer review only



For peer review only - http://bmjopen.bmj.com/site/about/guidelines.xhtml

# 6 Adherence to Ethical, Regulatory and Administrative Considerations

## 6.1 Ethical considerations

### 6.1.1 *General*

This study will be conducted in accordance with the Declaration of Helsinki 1964 as revised in Edinburgh in 2000 and with the National Health & Medical Research Council Guidelines on Human Experimentation.

### 6.1.2 *Ethics Committee Approval*

The primary ethics committee for the parent STAREE study is the Monash University Human Research Ethics Committee (CF14/1927 – 2014000975).
The study is also approved by the Governance Office (SSA) in Royal Brisbane and Women's Hospital (TBC).

### 6.1.3 *Information for participants*

Before obtaining consent from the participant they must be informed of the objectives, benefits, risks and requirements of the STAREE-MIND substudy, as well as the nature of the neuroimaging assessments. A participant consent form should be given to every participant at STAREE Baseline visit 1.

### 6.1.4 *Informed consent*

a) All STAREE participants must give their informed consent **before** they can be enrolled in the STAREE-MIND substudy.
b) Two copies of the consent form are to be provided, one for the participant and one for the investigator.
c) Informed consent is obtained from the participant by the site investigator and/or by the research staff. The research staff should fully inform the participant of all pertinent aspects of the STAREE-MIND substudy by reviewing the study information and consent form. All participants should be informed to the fullest extent possible about the study, in language and terms they are able to understand.
d) Prior to a participant's involvement in the study, the written Informed Consent Form should be signed, name filled in and personally dated by the participant and by the person who conducted the informed consent discussion. A copy of the signed and dated written Informed Consent Form will be provided to the participant. The original consent form is to be stored in the participant's individual study file, held by the investigator. A copy of signed, informed consent must be cited by study staff at the relevant Imaging Facility prior to commencement of the data collection session.
e) The form used for obtaining the participant's informed consent must be the current version that has been reviewed and approved by the appropriate Ethics Committee.


For peer review only - http://bmjopen.bmj.com/site/about/guidelines.xhtml

## 6.2 Regulatory considerations

### 6.1.5 *Financing*

STAREE MIND neuroimaging substudy has been awarded a project grant from the National Heart Foundation (Australia).

### 6.1.6 *Trial registration*

STAREE is registered with clinicaltrials.gov (Identifier NCT02099123). First posted March 28, 2014.

### 6.1.7 *Disclosure of conflict of interest*

Full disclosure by all of the investigators of their, and their immediate families, financial relationships with all pharmaceutical and biomedical companies judged to have an active or potential interest in the conduct and outcome of the study will be made.

## 6.3 Indemnity

Monash University shall at all times indemnify the study investigators and their staff from claims that may be made against them for any injury sustained by a study participant as a consequence of the follow-up for this study as outlined in this protocol. GPs insurers will be asked to indemnify the GP co-investigators who are participating in the study. Ramsay Healthcare will indemnify the study medication.

## 6.4 Governance

STAREE-MIND is overseen and co-ordinated by a steering committee comprised of Prof Sophia Zoungas (chair), Monash University; Prof John McNeil, Monash University; Dr Trevor Chong, Monash University; Dr Joanne Ryan, Monash University; Dr Ian Harding, Monash University.

The Steering Committee will provide oversight to the overall policy and direction of the project. It will be responsible for protocol generation and, where appropriate, modification, budgeting and all funding applications. The Steering Committee will meet regularly throughout the planning, execution and data analysis phases of the project, when it will consider reports from the Study Director and the EAC. The Steering Committee will be responsible for all the publications and communications relating to the study.

# 7 Data Management

## 7.1 Data handling and record keeping

All the data from assessments conducted during STAREE-MIND will be recorded as scanned images for each participant. All information will be treated in accordance with professional conduct. Scanned images will be stored on secure data servers at Monash University, and curated by staff at Monash Biomedical Imaging and Monash University IT (eSolutions). Scans from HIRF will be exported to Monash data servers, and stored locally temporarily for clinical reporting and short-term data redundancy.


For peer review only - http://bmjopen.bmj.com/site/about/guidelines.xhtml

## 8 References


1. Australian Government Department of Health. Australian Statistics on Medicines 2015.
2. Wood, W.G., Eckert, G.P., Igbavboa, U. & Muller, W.E. Statins and neuroprotection: a prescription to move the field forward. *Annals of the New York Academy of Sciences* **1199**, 69-76 (2010).
3. Bettermann, K.*, et al.* Statins, risk of dementia, and cognitive function: secondary analysis of the ginkgo evaluation of memory study. *Journal of stroke and cerebrovascular diseases : the official journal of National Stroke Association* **21**, 436-444 (2012).
4. Mattson, M.P. Pathways towards and away from Alzheimer's disease. *Nature* **430**, 631-639 (2004).
5. Bodovitz, S. & Klein, W.L. Cholesterol modulates alpha-secretase cleavage of amyloid precursor protein. *The Journal of biological chemistry* **271**, 4436-4440 (1996).
6. Refolo, L.M.*, et al.* Hypercholesterolemia accelerates the Alzheimer's amyloid pathology in a transgenic mouse model. *Neurobiology of disease* **7**, 321-331 (2000).
7. Urmoneit, B., Turner, J. & Dyrks, T. Cationic lipids and disturbance of cellular cholesterol and sphingomyelin distribution modulates gamma-secretase activity within amyloid precursor protein in vitro. *Prostaglandins & other lipid mediators* **55**, 331-343 (1998).
8. Sparks, D.L., Liu, H., Gross, D.R. & Scheff, S.W. Increased density of cortical apolipoprotein E immunoreactive neurons in rabbit brain after dietary administration of cholesterol. *Neuroscience letters* **187**, 142-144 (1995).
9. Dolan, H.*, et al.* Atherosclerosis, dementia, and Alzheimer disease in the Baltimore Longitudinal Study of Aging cohort. *Annals of neurology* **68**, 231-240 (2010).
10. Barone, E., Di Domenico, F. & Butterfield, D.A. Statins more than cholesterol lowering agents in Alzheimer disease: their pleiotropic functions as potential therapeutic targets. *Biochemical pharmacology* **88**, 605-616 (2014).
11. Wyss-Coray, T. Inflammation in Alzheimer disease: driving force, bystander or beneficial response? *Nature medicine* **12**, 1005-1015 (2006).
12. Griffin, W.S.*, et al.* Brain interleukin 1 and S-100 immunoreactivity are elevated in Down syndrome and Alzheimer disease. *Proceedings of the National Academy of Sciences of the United States of America* **86**, 7611-7615 (1989).
13. Engelhart, M.J.*, et al.* Inflammatory proteins in plasma and the risk of dementia: the rotterdam study. *Archives of neurology* **61**, 668-672 (2004).
14. Peila, R. & Launer, L.J. Inflammation and dementia: epidemiologic evidence. *Acta neurologica Scandinavica. Supplementum* **185**, 102-106 (2006).
15. Schmidt, R.*, et al.* Early inflammation and dementia: a 25-year follow-up of the Honolulu-Asia Aging Study. *Annals of neurology* **52**, 168-174 (2002).
16. Swardfager, W.*, et al.* A meta-analysis of cytokines in Alzheimer's disease. *Biological psychiatry* **68**, 930-941 (2010).
17. Menge, T., Hartung, H.P. & Stuve, O. Statins--a cure-all for the brain? *Nature reviews. Neuroscience* **6**, 325-331 (2005).
18. Wang, Q.*, et al.* Statins: multiple neuroprotective mechanisms in neurodegenerative diseases. *Experimental neurology* **230**, 27-34 (2011).


For peer review only


For peer review only - http://bmjopen.bmj.com/site/about/guidelines.xhtml

19. PBS Information Management Section. Pharmaceutical Policy Branch. Expenditure and prescriptions twelve months to 30 June 2013 PBS.
20. Simons, L.A., Ortiz, M. & Calcino, G. Long term persistence with statin therapy -- experience in Australia 2006-2010. *Australian family physician* **40**, 319-322 (2011).
21. Morgan, T.K.*, et al.* A national census of medicines use: a 24-hour snapshot of Australians aged 50 years and older. *The Medical journal of Australia* **196**, 50-53 (2012).
22. Ko, D.T., Mamdani, M. & Alter, D.A. Lipid-lowering therapy with statins in high-risk elderly patients: the treatment-risk paradox. *JAMA : the journal of the American Medical Association* **291**, 1864-1870 (2004).
23. Pencina, M.J.*, et al.* Application of new cholesterol guidelines to a population-based sample. *The New England journal of medicine* **370**, 1422-1431 (2014).
24. Zissimopoulos, J.M., Barthold, D., Brinton, R.D. & Joyce, G. Sex and Race Differences in the Association Between Statin Use and the Incidence of Alzheimer Disease. *JAMA Neurol* **74**, 225-232 (2017).
25. Geifman, N., Brinton, R.D., Kennedy, R.E., Schneider, L.S. & Butte, A.J. Evidence for benefit of statins to modify cognitive decline and risk in Alzheimer's disease. *Alzheimer's research & therapy* **9**, 10 (2017).
26. Ott, B.R.*, et al.* Do Statins Impair Cognition? A Systematic Review and Meta-Analysis of Randomized Controlled Trials. *Journal of general internal medicine* (2015).
27. FDA drug safety communication: Important safety label changes to cholesterol-lowering statin drugs 2012.
28. Begg, S.*, et al. The burden of disease and injury in Australia, 2003*, (Australian Institute of Health and Welfare (AIHW), Canberra, 2007).
29. Australian institute of Health and Welfare. Canberra. Dementia in Australia. Cat. no. AGE 70. Canberra: AIHW. (2012).


For peer review only


For peer review only - http://bmjopen.bmj.com/site/about/guidelines.xhtml

## Appendix 11: Detailed protocol revisions

| No. | Section | Section | Initials/Date |
|---|---|---|---|
| Version 1.2.2 June 2014 | Original approved version (drafts leading up to next version) | | |
| Version 1.3 March 2015 | Cognitive tests added | 5.2<br>7.1.5<br>Study Activity Schedule | SS 27/4/2015 |
| | CES-D-10 added | 5.2<br>7.1.2<br>Study Activity Schedule | |
| | Change from 2 yearly annual visits to yearly annual visits | 7.1.8<br>Study Activity Schedule | |
| | Biobank sub-study – sample collection | 7.1.5<br>Study Activity Schedule | |
| | One month follow up visit added | 7.1.7 | |
| Version 1.3.1 July 2015 | SF-12 changed to SF-36 | 5.2 | SS 17/8/2015 |
| | Biobank Steering Committee added to Governance structure | 12.5.1 | |
| Version 1.3.2 January 2016 | Endpoint definitions expanded | 5.2, 5.3, 11.1, 11.2<br>Appendix 2 | FOH 27/1/2016 |
| | Study medication post out change | | |
| | Study medication compliance questionnaire added | Appendix 4 | |
| | Table of contents added | | |
| | Expansion of SAE and SUSAR reporting | 11.2 | |
| Version 1.3.3 July 2016 | Updated exclusion criteria regarding cytochrome P450 3A4 inhibitors | 6.2<br>8.2.1 (added)<br>Appendix 3 | SS 13/7/2016 |
| | Community participant follow up added | 6.3.1 | |
| | International Steering Committee added | 12.5.1 | FO 14/7/2016 |



For peer review only - http://bmjopen.bmj.com/site/about/guidelines.xhtml

For peer review only

| | | | |
|---|---|---|---|
| | Change in biobank study samples collected | 7.1.5 | SS<br>13/9/2016 |
| | Addition of reporting requirements for AEs and SAEs | 11.1.0<br>Appendix 5 | SS<br>22/3/2017 |
| Version 2.0<br>July 2018 | Updates to align protocol with SPIRIT 2013 statement recommendations | Across protocol | AC/SZ/SS<br>18/7/2018 |
| | Addition of STAREE hubs | Appendix 1 | SS<br>18/7/2018 |
| | Addition of Data access policy | Section 12.4 | AC<br>12/3/2019 |
| | Addition of secondary endpoints and clarification of both primary and secondary endpoints | Section 5 | AC<br>12/3/2019 |
| | Addition of new Forest plot | Section 1.2 | AC<br>12/3/2019 |
| | Addition of single-lead ECG, IPAQ-E and TICS-M to measurement and study schedule | Table 2 | AC/SS<br>13/9/2019 |
| | Updated exclusion criteria regarding cytochrome P450 3A4 inhibitors | Appendix 3 | AC/IH/SS<br>9/9/2019 |
| | Updated dementia definition from DSM IV to DSM V | Appendix 2 | ES/SZ/SS<br>13/9/2019 |
| Version 3.0 | Removal of Bictegravir from table | Appendix 3 | AK<br>10/2/20 |
| | Addition of information on data security | Section 12.4 | AC/SS<br>10/2/20 |
| | Clarification of definition of disability free survival | Section 5.1 p 27 | AC<br>10/2/20 |
| | Modification of dementia and cognitive decline endpoint brief definitions | Appendix 2 | AC<br>13/2/20 &<br>July 2022 |
| | COVID response – schedule update | Appendix 9/ Table 2 | SS<br>27/05/2020 |
| | Modification of heart failure endpoint brief definition | Appendix 2 | AC<br>1/07/2020 |


For peer review only - http://bmjopen.bmj.com/site/about/guidelines.xhtml

| | | | |
|---|---|---|---|
| | Changes to power calculations, sample size and follow-up period | Section 10 | |
| | Addition of Executive Summary | Section 1 | AC 1/06/2021 |
| | Addition of STAREE MIND protocol | Appendix 10 | AC 1/06/2021 |
| | Extended Revisions table moved to appendices and replaced with summary revision table | Appendix 11 | AC 1/06/2021 |
| *Version 3.2* | Addition of Frailty phenotype as a pre-specified tertiary endpoint | Section 3.3, Section 6, Appendix 2 | AC 13/02/2023 |
| | Corrected references in Appendix 6 | Appendix 6 | AC 13/02/2023 |

For peer review only


For peer review only - http://bmjopen.bmj.com/site/about/guidelines.xhtml

# PARTICIPANT CONSENT FORM

*MONASH UNIVERSITY*

# PARTICIPANT INFORMATION SHEET/CONSENT FORM

*MONASH UNIVERSITY*

**Full Project Title:** The STAREE Healthy Ageing Biobank

This participant information and consent form is 9 pages long. Please make sure you have all the pages. For any queries, information on the project or to speak to the research team please call **1800 770 664**.

## INTRODUCTION

You are being invited to provide a blood and urine sample for the STAREE Healthy Ageing Biobank because you are a participant in the STAREE Clinical Trial. The sample you provide for the STAREE Healthy Ageing Biobank is in addition to any blood and urine that you have taken for the STAREE clinical trial. This is because research using blood and urine samples may help us to find out more about what causes disease, how to prevent it, and how to treat it. The research project aims to create a collection of blood and urine samples which scientists can then use in the future to identify biomarkers or genes associated with conditions such as heart attacks, strokes, cancer and Alzheimer's disease.

This Participant Information and Consent Form tells you about the research project. It explains what is involved to help you decide if you want to take part.

Please read this information carefully. Ask questions about anything that you don't understand or want to know more about. Before deciding whether or not to take part, you might want to talk about it with a relative, friend or your local health worker.

Participation in this research is voluntary. If you don't wish to take part, you don't have to. Note that consent for this additional research is separate to the informed consent you have already provided for participation in the STAREE Clinical Trial. If you do not wish to take part in this additional project you may still participate in the STAREE Clinical Trial. Additionally, consent can be given for different parts of the study, as indicated by the checked boxes at the end of this consent form.

If you decide you want to take part in the research project, you will be asked to sign the consent section. By signing it you are telling us that you:

- understand what you have read;
- consent to take part in the research project;


For peer review only - http://bmjopen.bmj.com/site/about/guidelines.xhtml

# PARTICIPANT CONSENT FORM

MONASH UNIVERSITY

- consent to be involved in the procedures described;
- consent to the use of your personal and health information as described.

You will be given a copy of this Participant Information and Consent Form to keep.

**WHAT IS THE PURPOSE OF THIS RESEARCH PROJECT?**

- *What are biomarkers and genes, and why are they important?*

Biomarkers, including proteins, which are found in the blood and urine, are molecules that are needed for the structure, function and regulation of cells that make up the body's tissues and organs. We are still learning about the proteins that are present in healthy people such as yourself.

In addition to proteins, genetic material (or DNA) can also be collected from blood and urine. The unique way in which our many genes are arranged, is what makes us all different. However there are some sections of DNA which will almost always cause some diseases, or at least increase the risk of someone suffering from a condition or disease. We still have much to learn about these critical sections of DNA. For example, it is estimated that the genetic predisposition for heart disease may involve more than 200 genes. However the exact role is known for only a small number of these genes.

Previous studies have shown that changes in certain proteins and genetic material can be detected in people with cardiovascular disease, forms of cancer, and in Alzheimer's disease. However much more work needs to be carried out before we know which particular proteins and genes play the biggest role in actually causing these diseases.

- What is this study about?

We would like to see whether we can identify known or new proteins and/or genes in the blood and urine that may be linked with the incidence of strokes, heart attacks, bleeding episodes, changes in mental function and development of cancer.

We are seeking to collect blood and urine samples from participants who decide to take part in the STAREE Clinical Trial.

This research has been funded by Monash University.


For peer review only - http://bmjopen.bmj.com/site/about/guidelines.xhtml

# PARTICIPANT CONSENT FORM

*MONASH UNIVERSITY*

**WHAT DOES PARTICIPATION IN THIS RESEARCH PROJECT INVOLVE?**

**Procedures**: We are asking you to provide a blood and urine sample for the STAREE Biobank.

At a STAREE Biobank visit, either a research nurse, or other trained professional, will collect the blood sample for the STAREE Biobank. You will be provided with a collection cup for the urine sample.

The sample collection for the Biobank involves taking 30–40 ml (just over 2 tablespoons) of your blood and collecting up to 70mls of your urine, and will add about 15 minutes to the length of your second visit or take about 15 minutes at a convenient location near you.

You are **not** required to fast before providing the additional blood and urine sample for the STAREE Biobank. After you have provided the blood and urine sample, it will have your name and any identifying features removed, and replaced with a code number. That code number will be the only information attached to your sample.

Your blood and urine samples will be processed and transported (if necessary) to the STAREE Healthy Ageing Biobank laboratory at the Clinical Trials Centre, Monash University, for storage (and other locations, such as the Alfred hospital, may be used as long-term storage sites in future). Research staff at this laboratory will not be able to link the code number to your identity and therefore will not know to whom the sample belongs.

**Follow-up:** If you agree to provide a blood and urine sample for the Biobank, but do not ultimately continue on in the STAREE Clinical Trial for whatever reason, you can still choose to remain a participant in the Biobank. This will mean that your stored blood and urine samples are kept in the STAREE Biobank, and we will contact you on an annual basis for up to 5 years to ask you a few questions about your health. We will also continue to have access to your medical records (your doctor's records, and those of any hospital or specialist whom you may visit).

We also ask you for permission to obtain information about you from a government database known as the National Death Index. Providing us with this information greatly facilitates us knowing about your health status.

(If you remain in the STAREE Clinical Trial, follow-up will consist of the above, but annual contact will occur via face-to-face visits with the research staff).

You may be asked to provide another 30–40 ml of your blood and up to 70mls of urine at another time during the study, such as at the 3 year time point and/or at the end of the study (in about 5 years).


For peer review only - http://bmjopen.bmj.com/site/about/guidelines.xhtml

# Participant Consent Form

*Monash University*

**Reimbursement:** You will not be paid for your participation in this research.

Although knowledge acquired through medical research may lead to discoveries that are of commercial value, there will be no financial benefit to yourself, your family or the STAREE Biobank.

### What are the possible benefits?

You will not benefit directly from the results of this research, but it is hoped that the results of research on your blood and urine, and those from other participants, may help doctors gain further understanding of how to reduce the risk of death, heart attacks, strokes, heart failure, cancer or dementia in people like yourself. Your blood and urine will be helpful whether you develop a major disease or not.

### What are the possible risks?

**Blood collection:** When blood samples are taken from a vein in your arm you may experience some discomfort. A small number of participants may faint, have some bruising, or a minor infection or blood clot form under the skin. These are generally minor discomforts which should resolve themselves in a few days.

If you should feel light-headed or dizzy during or after the blood sample collection procedure you will be encouraged to remain at the Clinical Trials Centre (or alternative blood collection site) until you feel comfortable leaving.

**Confidentiality:** The code number assigned to your blood and urine sample will be linked to your personal information that is stored on a separate database held at Monash University, Department of Epidemiology & Preventive Medicine. Therefore, the possibility exists that your blood and urine sample could be re-identified, however this is most unlikely to occur – see the section **What will happen to information about me?**

**Genetic testing:** Genetic testing of samples may occur in the future if additional funding is available. Any future projects must be approved by a Human Research Ethics Committee. If any future research uncovers any significant information specific to your health, our STAREE Steering Committee may decide to authorise someone to contact you and offer you access to this information. You may decline the information. "Significant" information is any findings that are deemed very serious or highly significant, that have been validated and to which there is known medical support available. If you wish to be given this information a qualified person will explain it to you. You should also consider whether this information should be made known to your family members. Sharing these findings could help avoid similar medical problems in your family. Before deciding whether or not to receive these results, you may want to discuss this with a relative or a significant other first. By signing this consent, you will


For peer review only - http://bmjopen.bmj.com/site/about/guidelines.xhtml

# Participant Consent Form

*Monash University*

be asked to select if you wish to be informed or not informed of any serious or highly significant genetic results in the future that has any specific health implications for you or your genetic relatives – see page 9.

### Do I have to take part in this research project?

Participation in any research project is voluntary. If you do not wish to take part, you do not have to. If you decide to take part and later change your mind, you are free to withdraw from the project at a later stage.

If you decide to withdraw, please notify a member of the STAREE research team in writing. This will allow that person or the research supervisor to inform you if there are any special requirements linked to withdrawing.

If you decide to leave the project, the researchers would like to keep the blood and urine samples and the personal and health information about you that has already been collected. This is to help them make sure that the results of the research can be measured properly. However, you may elect to have samples destroyed and health and personal information removed from the study. If you choose to have your samples and information removed you must tell the researchers before you withdraw from the research project.

Your decision whether to take part or not, or to take part and then withdraw, will not affect your relationship with the researchers or your doctor.

### How will I be informed of the final results of this research project?

The establishment of the STAREE Healthy Ageing Biobank is purely for research purposes only. At this formative stage, the Biobank cannot be used for the diagnosis of diseases because we do not yet know which particular biomarkers or genes will emerge, in the years to come, as being the most significant in terms of predicting disease onset. The ongoing nature of the development of the Biobank means that the final results of this project will not be available for some considerable time. If however in the future, genetic testing is undertaken and you consent to take part in this study, you will be asked at the end of this information sheet to consider being notified or not notified of any significant result (see also page 9).

Eventually, results of group data from the research that is conducted will be published in medical journals, and presented at national and international conferences.

### What will happen to information about me?

The STAREE Biobank laboratory is equipped to store and handle blood and urine samples appropriately for future tests. Your potentially re-identifiable blood and urine samples will be



For peer review only - http://bmjopen.bmj.com/site/about/guidelines.xhtml

# PARTICIPANT CONSENT FORM

*MONASH UNIVERSITY*

stored for a long time, possibly for many years after the completion of the study. When all the samples are no longer required they will be destroyed by the STAREE Biobank laboratory, or the laboratory in which the research has been conducted, in a safe manner according to local laboratory procedures.

**Who will make use of my blood and urine samples?**

The STAREE Biobank is a non-profit service dedicated to providing a resource to scientists involved in medical research. Researchers from universities, hospitals, and other health organizations conduct research using blood and urine. They may contact the STAREE Biobank and request samples for their studies. The researcher must have funding to carry out the study, and must have the study reviewed scientifically, before they are permitted to access the blood and urine samples from the STAREE Biobank. All studies must have approval by a Human Research Ethics Committee. Your samples will NOT be sold. The STAREE Biobank may charge researchers a fee to recover some of the costs of storing and administering its collection of samples.

Your blood and urine sample will not be linked to your name, initials, date of birth or any other information that may identify you.

**What types of tests will be performed?**

The tests performed on your blood and urine may include tests for inflammation, nutrition, insulin, hormones and genes that are thought to be related to heart disease, diabetes, dementia, cancer or other major diseases. Some researchers may develop new tests to identify diseases. Others may develop new ways to treat or even cure diseases. In the future, some of the research may help develop new products, such as tests and drugs.

Some research looks at diseases that are passed on in families (called familial or hereditary genetic research). Research done with your blood and urine may look for genetic causes and signs of disease.

Rapid advances in technology make it impossible to predict what new tests or studies may be possible in the future. The selection of tests will be guided by current and future scientific information about heart disease, diabetes, dementia, cancer or other major diseases.

**You may consent to having your blood and urine sample tested for biomarkers, genetic markers, or both; see Consent Form, Page 9.**

Any information obtained in connection with this research project that can identify you will remain confidential and will only be used for the purpose of this research project. It will only be disclosed with your permission, except as required by law.


For peer review only - http://bmjopen.bmj.com/site/about/guidelines.xhtml

# PARTICIPANT CONSENT FORM

*MONASH UNIVERSITY*

In any publication and/or presentation, information will be provided in such a way that you cannot be identified. The STAREE Biobank and the STAREE Co-ordinating Centre (Monash University) are responsible for ensuring the information about you is kept private. They will take every precaution to safeguard your identity, and prevent misuse of records. Only authorised people who have agreed in writing to protect your identity will have access to your linked information. Therefore, all other researchers working with your samples and clinical information will not know your identity. This will make it very difficult for any research results (and importantly, those relating to genetic information) to be linked to you or your family. Also, people outside the research process will not have access to results about any one person, which will help to protect your privacy.

### CAN I ACCESS RESEARCH INFORMATION KEPT ABOUT ME?

A report of the individual results of any tests done on your blood and urine samples will not routinely be made available to you, your family members or any other person. This is because research can take a long time and must use blood and/or urine samples from many people before results are known. Results from research using your samples may not be ready for many years and will not affect your care right now, but they may be helpful to people like you in the future.

In accordance with relevant Australian and/or Victorian privacy and other relevant laws, you have the right to access the information collected and stored by the researchers about you. Please contact one of the researchers named at the end of this document if you would like to access your information.

Further, in accordance with regulatory guidelines, the information collected in this research project will be kept indefinitely. You must be aware that the information may become de-identified at some point and access to information about you after this point will not be possible.

### IS THIS RESEARCH APPROVED?

The ethical aspects of this research project have been approved by the Alfred Hospital Ethics Committee.

This project will be carried out according to the *National Statement on Ethical Conduct in Human Research (2007)* produced by the National Health and Medical Research Council of Australia. This statement has been developed to protect the interests of people who agree to participate in human research.


For peer review only - http://bmjopen.bmj.com/site/about/guidelines.xhtml

# Participant Consent Form

*Monash University*

## Further information and who to contact

Please ask the research staff member if you have any further questions.

For specific enquiries related to this study, or matters relating to research at the site at which you are participating, or any questions about being a research participant in general, you may contact:

Name: Associate Professor Sophia Zoungas (Monash University):
Phone: +61 3 9903 0711
E-mail: sophia.zoungas@monash.edu

If you have any complaints about any aspect of the project or the way it is being conducted, you may contact:
Complaints Officer
Office of Ethics & Research Governance
Alfred Health

Tel: (03) 9076 3619

You will need to quote the following Alfred Health project number: 266/15.


For peer review only - http://bmjopen.bmj.com/site/about/guidelines.xhtml

# Participant Consent Form

*Monash University*

**Title** The STAREE Healthy Ageing Biobank

## Declaration by Participant

I have read, or have had read to me, the *"The STAREE Healthy Ageing Biobank" Patient Information, Version 1.6.1, Dated 17 April 2018* in a language that I understand, and I understand the purposes, procedures and risks of this research project as described within it.

I have had an opportunity to ask questions and I am satisfied with the answers I have received.

I freely agree to participate in this research project, as described.

I understand that I will be given a signed copy of this document to keep.

The researcher has agreed not to reveal my identity and personal details if information about this project is published or presented in any public form.

I agree to my blood and urine samples being stored and tested for **biomarkers** in the future

☐ YES ☐ NO

I agree to my blood and urine samples being stored and tested for **genetic markers** in the future

☐ YES ☐ NO

I agree to be **informed** of any significant genetic results that have specific health implications for me and/or my genetic relatives

☐ YES ☐ NO

Name of participant (please print)________________________________________

Signature____________________________________Date__________________

## Declaration by Researcher

I have given a verbal explanation of the research project, its procedures and risks and I believe that the participant has understood that explanation.

Name of researcher (please print)________________________________________

Signature____________________________________Date__________________



For peer review only - http://bmjopen.bmj.com/site/about/guidelines.xhtml

**Appendix 4 Assumptions for STAREE disability free survival (DFS) event rates**

DFS event rates for STAREE were calculated based on the event rates for Australian participants in the ASPREE study.

ASPREE Australian participants – rates averaged over 4.7years of follow up (Ryan J, Storey E, Murray AM et al. Randomized placebo-controlled trial of the effects of aspirin on dementia and cognitive decline. Neurology. 2020 Jul 21;95(3):e320-e331)

| | |
|---|---|
| 70-74y: | 3.42 per 1000 person-years |
| 75-79y: | 7.31 per 1000 person-years |
| 80-84y: | 16.00 per 1000 person-years |
| 85+y: | 21.37 per 1000 person-years |

ASPREE Australian participants – rates in years 2-7 if zero events in first year of 4.7y of follow-up

| | |
|---|---|
| 70-74y: | 4.39 per 1000 person-years |
| 75-79y: | 9.30 per 1000 person-years |
| 80-84y: | 20.24 per 1000 person-years |
| 85+y: | 27.52 per 1000 person-years |

Assuming STAREE dementia process produces rates 33% higher

| | |
|---|---|
| 70-74y: | 5.84 per 1000 person-years |
| 75-79y: | 12.37 per 1000 person-years |
| 80-84y: | 26.92 per 1000 person-years |
| 85+y: | 36.60 per 1000 person-years |

DFS rates based on assumed dementia rates above and combining with assumed death and persistent physical disability rates as follows: (death+dementia+disability) less 10% to account for individuals experiencing more than one of the three types of event

| | | | |
|---|---|---|---|
| 70-74y: | (10.0 | + 5.84 + 4.5)*0.9 | = 18.3 per 1000 person-years |
| 75-79y: | (17.7 | + 12.37 + 7.6)*0.9 | = 33.9 per 1000 person-years |
| 80-84y: | (33.6 | + 26.92 + 12.5)*0.9 | = 65.7 per 1000 person-years |
| 85+y: | (66.6 | + 36.60 + 25)*0.9 | = 115.4 per 1000 person-years |

For peer review only

**Appendix 5**

**Data and Safety Monitoring Board Charter for A randomised clinical trial of STAtin therapy for Reducing Events in the Elderly (STAREE)**

**(Short title: STAREE DSMB Charter)**

**Charter approved by Monash University**

Signature: ________________________________ Date:____________

**Sponsor Signatory**: Professor Sophia Zoungas

**DSMB Approved:**

Signature: ________________________________ Date:____________

**DSMB Chair:**

For peer review only

## 1. Introduction

**Trial name:** Statins in Reducing Events in the Elderly (STAREE)

**Trial objectives:**

**Scope of Charter:** This charter outlines the composition and responsibilities of the STAREE DSMB.

## 2. DSMB responsibilities

The Data and Safety Monitoring Board (DSMB) will monitor participant safety, data quality and evaluate the progress of the study.

- Review the research protocol, informed consent documents and plans for data safety and monitoring;
- Review any proposed modifications to the trial prior to their implementation;
- Evaluate the progress of the trial, including periodic assessments of data quality and timeliness, recruitment, accrual and retention, participant risk versus benefit, performance of the trial sites, and other factors that can affect study outcomes;
- Consider factors external to the study when relevant information becomes available, such as scientific or therapeutic developments that may have an impact on the safety of the participants or the ethics of the trial;
- Review study performance, make recommendations and assist in the resolution of problems reported by the Principal Investigators;
- Make recommendations to the Principal Investigators concerning continuation, termination or other modifications of the trial based on the observed beneficial or adverse effects of the treatment under study;
- If appropriate, review interim analyses in accordance with stopping rules, which are clearly defined in advance of data analysis and have the approval of the DSMB; and
- Ensure the confidentiality of the study data and the results of monitoring.

## 3. Membership

The DSMB will consist of 4 members and 3 will constitute a quorum.

Membership consists of persons completely independent of the investigators who have no financial, scientific, or other conflict of interest with the trial. Written documentation attesting to absence of conflict of interest is required from each member.

Professor John Simes, University of Sydney, has been selected by the Principal Investigators to serve as the Chairperson and is responsible for overseeing the meetings and developing the agenda in consultation with the Principal Investigators. The Chair is the contact person for the DSMB. Monash University will provide the logistical management and support for the DSMB.

## 4. Organisation of meetings

At the first meeting the DSMB will discuss the protocol, suggested modifications and establish guidelines to study monitoring by the Board. In consultation with the Principal Investigator, the DSMB chairperson will prepare the agenda to address the review of study materials, modifications to the study protocol and informed consent document, initiation of the trial, appointment of a safety

For peer review only

officer, reporting of adverse events, statistical analysis plan including interim analysis and stopping rules and/or other matters as appropriate.

Meetings of the DSMB will be held annually for the first two years and 6 monthly thereafter. An emergency meeting of the DSMB may be called at any time by the Chairperson if participant safety questions or other unanticipated problems arise.

Meetings will be closed to the public because discussions may address confidential participant data. Meetings may be convened as conference calls as well as in person.

Meetings will consist of open and closed sessions. Discussion held in all sessions will be confidential.

At least one of the Principle Investigators and key members of the study team will attend open sessions. Open session discussion will focus on the conduct and progress of the study, including participant accrual, protocol compliance and problems encountered. Unblinded data will not be presented in the open sessions.

Closed sessions will be attended by the DSMB members. The study statistician may be present at the request of the DSMB. Any data by blinded study group and, as necessary, unblinded data, will be presented during the closed session. The DSMB can request review of unblinded data during the closed session. Investigators will still remain blinded.

Each meeting must include a recommendation to continue or terminate the study made by a formal DSMB majority or unanimous vote. Should the DSMB decide to issue a termination recommendation, the full vote of the DSMB is required. A recommendation to terminate the study may be made by the DSMB at any time by majority vote.

Following each DSMB meeting, the Chair will formally write to the STAREE Chief Investigators to advise them that the monitoring of the study's progress is up to date, and that the DSMB recommends study continuation or discontinuation (whichever applies).

**5. Documentation**

DSMB interim report templates will be prepared by the study staff, typically the statistician, to be reviewed by the DSMB members at the first meeting. Interim data reports generally consist of two parts;

- Part 1 – Open session report, and
- Part 2 – Closed session report

The format and content of the reports for both the open and closed sessions and plans for interim analyses will be finalised and approved by the DSMB, although changes throughout the trial may be requested.

The reports will list and summarise safety data and describe the status of the study. All meeting materials will be sent to the DSMB at least 10 to 14 days prior to the meeting by secure email.

5.1 Open Session reports

Open session reports will include:

- Site administrative reports describing –
    - Numbers of participants screened, enrolled, completed and withdrawn
    - Baseline characteristics of the study population

For peer review only

- o General information on study status
- o Blinded listings of adverse events and serious adverse events as requested by the DSMB
- o Other information as requested by the DSMB on a continuing or one off basis

5.2 Closed Session reports

Closed session reports will include:

- Information described in Part 1 but by blinded treatment group (e.g. group A/ group B).
- Information on:
  - o Study outcomes
  - o Safety data
  - o Efficacy data
  - o Interim analyses

Closed session reports will be destroyed at the conclusion of the meeting. If meetings are held by telephone printed copies of the closed reports should be destroyed immediately following the meeting.

5.3 Additional reports

Mailings to the DSMB: As per the schedule (agreed by the DSMB) blinded safety data will be communicated to all or selected DSMB members

Access to Interim data: Access to the accumulating endpoint data will be limited to as small a group as possible, such as the independent statistician. Limiting the access of interim data to the DSMB members relieves the investigator of the burden of deciding whether it is ethical to continue to randomise individuals and helps protect the study from bias in the recruitment and/or evaluation.

5.4 Interim analyses

The DSMB will determine when interim analyses will take place. Interim analyses will be reviewed in closed sessions and may be presented in blinded group format (e.g. group A/ group B). The DSMB may also request review of the unblinded data.

Interim analyses of efficacy data will be performed only if they are planned and approved in advance and if stopping criteria are clearly defined.

**6. DSMB reports**

It is the responsibility of the Principal Investigators to distribute the DSMB recommendations to all co-investigators and to ensure that copies are submitted to all ethics committees associated with the study.

**7. Confidentiality**

All materials, discussions and proceedings of the DSMB are completely confidential. Members and other participants in DSMB meetings are expected to maintain confidentiality.

For peer review only

**Appendix 6 – STAREE investigators, sites and acknowledgements**

**The STAREE investigator group includes the following:**

**Principal Investigator:**

Prof. Sophia Zoungas MBBS (Hons) FRACP PhD

**Steering Committee:**

Prof. Sophia Zoungas (Chair), Prof. Lawrence Beilin, Ass. Prof. Trevor T-J Chong, Prof. Geoffrey C Cloud, Prof. John J McNeil, Prof. Mark R Nelson, Prof. Stephen J Nicholls, Prof. Christopher M Reid, Prof. Andrew Tonkin, Dr Stephanie A Ward, Prof. Anthony S Wierzbicki, Prof. Rory Wolfe

**Executive Committee:**

Prof. Sophia Zoungas (Chair), Dr Andrea J Curtis, Ass. Prof. Ingrid Hopper, Dr Alissia Kost, Prof. John J McNeil, Prof. Mark R Nelson, Prof. Christopher M Reid, Ass. Prof. Joanne Ryan, Dr Simone Spark, Prof. Rory Wolfe.

**Data Safety Monitoring Committee:**

Prof. John Simes (Chair), Prof. Graeme Hankey, Prof. A Mark Richards and Prof. Mark Woodward. Dr Alan Herschtal and Dr Thao Le (independent statisticians).

**Endpoint Adjudication Committees:**

**Death:**

Prof. Mark R Nelson (Chair), Prof. Danny Liew, Dr Vazhma Qaderi, Prof. Anthony S Wierzbicki.

**Dementia/Disability:**

Ass. Prof. Trevor T-J Chong (Chair), Dr Chris Moran, Dr Stephanie A Ward.

**Myocardial Infarction:**

Prof. Stephen J Nicholls (Chair), Ass. Prof. Dion Stub, Prof. Andrew Tonkin.

**Stroke:**

Prof. Geoffrey C Cloud (Chair), Prof.Craig Anderson, Dr Barry Moynihan, Prof. Michael O'Sullivan.

**Heart Failure:**

Ass. Prof. Ingrid Hopper (Chair), Prof. John Amerena, Prof. David Kaye.

**General Practice Advisory Committee:**

Prof. Mark R Nelson (Chair), Dr Gary Deed, Dr Alissia Kost.

**Operations Committee:**

Dr Simone Spark (Chair), Ms Lucy Boorn, Ms Sue Critchley, Dr Andrea J Curtis, Mr Zachary Flanagan, Mr Stephen Glanville, Dr Alissia Kost, Ms Kate Vaughan.

**Data Access and Publications Policy Committee:**

Prof. Sophia Zoungas, Dr Andrea J Curtis, Mr Zachary Flanagan, Prof. Christopher M Reid, Dr Simone Spark, Prof. Rory Wolfe.

**STAREE Regional Leaders:**

**Victoria and South Australia:**

For peer review only

Prof. Sophia Zoungas (Melbourne, Central Victoria, Northern Victoria, South West Victoria, Geelong, Gippsland, East Gippsland, Mildura, Adelaide, Mount Gambier)

**Tasmania:**

Prof. Mark R Nelson (Hobart, Launceston, Burnie)

**Western Australia:**

Prof. Christopher M Reid (Perth, Bunbury)

**Queensland:**

Dr Gary Deed (Brisbane, Gold Coast, Toowoomba, Sunshine Coast, Hervey Bay)

**New South Wales:**

Prof. Andrew Boyle (Sydney, Newcastle, Port Macquarie, Wollongong, North Coast, Tamworth)

**Sub studies**

STAREE-Heart Principal Investigator: Ass. Prof, Ingrid Hopper

STAREE-MIND Principal Investigator: Ass. Prof. Joanne Ryan

**Acknowledgements**

We thank all past and present STAREE staff.

We thank Professors Elsdon Storey and Robyn Langham for their past contributions to the STAREE trial Steering Committee.

A list of all current STAREE staff can be found at: https://www.monash.edu/medicine/staree/about-staree/our-team

For peer review only

# Reporting checklist for protocol of a clinical trial.

Based on the SPIRIT guidelines.

## Instructions to authors

Complete this checklist by entering the page numbers from your manuscript where readers will find each of the items listed below.

Your article may not currently address all the items on the checklist. Please modify your text to include the missing information. If you are certain that an item does not apply, please write "n/a" and provide a short explanation.

Upload your completed checklist as an extra file when you submit to a journal.

In your methods section, say that you used the SPIRITreporting guidelines, and cite them as:

Chan A-W, Tetzlaff JM, Gøtzsche PC, Altman DG, Mann H, Berlin J, Dickersin K, Hróbjartsson A, Schulz KF, Parulekar WR, Krleža-Jerić K, Laupacis A, Moher D. SPIRIT 2013 Explanation and Elaboration: Guidance for protocols of clinical trials. BMJ. 2013;346:e7586

| | | Reporting Item | Page Number |
|---|---|---|---|
| **Administrative information** | | | |
| Title | #1 | Descriptive title identifying the study design, population, interventions, and, if applicable, trial acronym | 1 |
| Trial registration | #2a | Trial identifier and registry name. If not yet registered, name of intended registry | 4 |
| Trial registration: data set | #2b | All items from the World Health Organization Trial Registration Data Set | Appendix 2 |
| Protocol version | #3 | Date and version identifier | Appendix 3: STAREE protocol V3.0 |
| Funding | #4 | Sources and types of financial, material, and other support | 25 |
| Roles and | #5a | Names, affiliations, and roles of protocol | 1 & 25 |

For peer review only

| | | | |
|---|---|---|---|
| responsibilities: contributorship | | contributors | |
| Roles and responsibilities: sponsor contact information | #5b | Name and contact information for the trial sponsor | 25 |
| Roles and responsibilities: sponsor and funder | #5c | Role of study sponsor and funders, if any, in study design; collection, management, analysis, and interpretation of data; writing of the report; and the decision to submit the report for publication, including whether they will have ultimate authority over any of these activities | 25 |
| Roles and responsibilities: committees | #5d | Composition, roles, and responsibilities of the coordinating centre, steering committee, endpoint adjudication committee, data management team, and other individuals or groups overseeing the trial, if applicable (see Item 21a for data monitoring committee) | 15, 20, 25, Appendix 1 |
| **Introduction** | | | |
| Background and rationale | #6a | Description of research question and justification for undertaking the trial, including summary of relevant studies (published and unpublished) examining benefits and harms for each intervention | 5 to 9 |
| Background and rationale: choice of comparators | #6b | Explanation for choice of comparators | 5 to 9 |
| Objectives | #7 | Specific objectives or hypotheses | 9 |
| Trial design | #8 | Description of trial design including type of trial (eg, parallel group, crossover, factorial, single group), allocation ratio, and framework (eg, superiority, equivalence, non-inferiority, exploratory) | 10 |
| **Methods: Participants, interventions, and** | | | |

For peer review only

| | | | |
|---|---|---|---|
| outcomes | | | |
| Study setting | #9 | Description of study settings (eg, community clinic, academic hospital) and list of countries where data will be collected. Reference to where list of study sites can be obtained | 10 |
| Eligibility criteria | #10 | Inclusion and exclusion criteria for participants. If applicable, eligibility criteria for study centres and individuals who will perform the interventions (eg, surgeons, psychotherapists) | 11 |
| Interventions: description | #11a | Interventions for each group with sufficient detail to allow replication, including how and when they will be administered | 12 |
| Interventions: modifications | #11b | Criteria for discontinuing or modifying allocated interventions for a given trial participant (eg, drug dose change in response to harms, participant request, or improving / worsening disease) | 12, 13 |
| Interventions: adherance | #11c | Strategies to improve adherence to intervention protocols, and any procedures for monitoring adherence (eg, drug tablet return; laboratory tests) | 12 & 13 |
| Interventions: concomitant care | #11d | Relevant concomitant care and interventions that are permitted or prohibited during the trial | 13 |
| Outcomes | #12 | Primary, secondary, and other outcomes, including the specific measurement variable (eg, systolic blood pressure), analysis metric (eg, change from baseline, final value, time to event), method of aggregation (eg, median, proportion), and time point for each outcome. Explanation of the clinical relevance of chosen efficacy and harm outcomes is strongly recommended | 9, 14-16 |
| Participant timeline | #13 | Time schedule of enrolment, interventions (including any run-ins and washouts), assessments, and visits for participants. A schematic diagram is highly recommended (see Figure) | Appendix 3 |
| Sample size | #14 | Estimated number of participants needed to | 16-18, Appendix |

For peer review only

| | | | |
|---|---|---|---|
| | | achieve study objectives and how it was determined, including clinical and statistical assumptions supporting any sample size calculations | 4 |
| Recruitment | #15 | Strategies for achieving adequate participant enrolment to reach target sample size | 9-10 |
| **Methods: Assignment of interventions (for controlled trials)** | | | |
| Allocation: sequence generation | #16a | Method of generating the allocation sequence (eg, computer-generated random numbers), and list of any factors for stratification. To reduce predictability of a random sequence, details of any planned restriction (eg, blocking) should be provided in a separate document that is unavailable to those who enrol participants or assign interventions | 11 |
| Allocation concealment mechanism | #16b | Mechanism of implementing the allocation sequence (eg, central telephone; sequentially numbered, opaque, sealed envelopes), describing any steps to conceal the sequence until interventions are assigned | 11 |
| Allocation: implementation | #16c | Who will generate the allocation sequence, who will enrol participants, and who will assign participants to interventions | 11 |
| Blinding (masking) | #17a | Who will be blinded after assignment to interventions (eg, trial participants, care providers, outcome assessors, data analysts), and how | 14 |
| Blinding (masking): emergency unblinding | #17b | If blinded, circumstances under which unblinding is permissible, and procedure for revealing a participant's allocated intervention during the trial | 14 |
| **Methods: Data collection, management, and** | | | |

For peer review only

| | | | |
|---|---|---|---|
| **analysis** | | | |
| Data collection plan | #18a | Plans for assessment and collection of outcome, baseline, and other trial data, including any related processes to promote data quality (eg, duplicate measurements, training of assessors) and a description of study instruments (eg, questionnaires, laboratory tests) along with their reliability and validity, if known. Reference to where data collection forms can be found, if not in the protocol | 12, Appendix 3 STAREE protocol V 3.0 |
| Data collection plan: retention | #18b | Plans to promote participant retention and complete follow-up, including list of any outcome data to be collected for participants who discontinue or deviate from intervention protocols | Appendix 3: STAREE protocol V 3.0 |
| Data management | #19 | Plans for data entry, coding, security, and storage, including any related processes to promote data quality (eg, double data entry; range checks for data values). Reference to where details of data management procedures can be found, if not in the protocol | Appendix 3: STAREE Protocol V3.0 |
| Statistics: outcomes | #20a | Statistical methods for analysing primary and secondary outcomes. Reference to where other details of the statistical analysis plan can be found, if not in the protocol | 18-20 |
| Statistics: additional analyses | #20b | Methods for any additional analyses (eg, subgroup and adjusted analyses) | 20 |
| Statistics: analysis population and missing data | #20c | Definition of analysis population relating to protocol non-adherence (eg, as randomised analysis), and any statistical methods to handle missing data (eg, multiple imputation) | 19 |
| **Methods: Monitoring** | | | |
| Data monitoring: formal committee | #21a | Composition of data monitoring committee (DMC); summary of its role and reporting structure; statement of whether it is independent from the | 20-21 |

For peer review only

| | | | |
|---|---|---|---|
| | | sponsor and competing interests; and reference to where further details about its charter can be found, if not in the protocol. Alternatively, an explanation of why a DMC is not needed | |
| Data monitoring: interim analysis | #21b | Description of any interim analyses and stopping guidelines, including who will have access to these interim results and make the final decision to terminate the trial | 21 |
| Harms | #22 | Plans for collecting, assessing, reporting, and managing solicited and spontaneously reported adverse events and other unintended effects of trial interventions or trial conduct | 13 |
| Auditing | #23 | Frequency and procedures for auditing trial conduct, if any, and whether the process will be independent from investigators and the sponsor | Appendix 3: Study Protocol V3.0 |
| **Ethics and dissemination** | | | |
| Research ethics approval | #24 | Plans for seeking research ethics committee / institutional review board (REC / IRB) approval | 21 |
| Protocol amendments | #25 | Plans for communicating important protocol modifications (eg, changes to eligibility criteria, outcomes, analyses) to relevant parties (eg, investigators, REC / IRBs, trial participants, trial registries, journals, regulators) | Appendix 3 STAREE protocol v3.0 |
| Consent or assent | #26a | Who will obtain informed consent or assent from potential trial participants or authorised surrogates, and how (see Item 32) | 10 |
| Consent or assent: ancillary studies | #26b | Additional consent provisions for collection and use of participant data and biological specimens in ancillary studies, if applicable | Appendix 4 |
| Confidentiality | #27 | How personal information about potential and enrolled participants will be collected, shared, and maintained in order to protect confidentiality before, during, and after the trial | Appendix 3 STAREE Protocol |

For peer review only

| | | | |
|---|---|---|---|
| Declaration of interests | #28 | Financial and other competing interests for principal investigators for the overall trial and each study site | 26 |
| Data access | #29 | Statement of who will have access to the final trial dataset, and disclosure of contractual agreements that limit such access for investigators | 22 |
| Ancillary and post trial care | #30 | Provisions, if any, for ancillary and post-trial care, and for compensation to those who suffer harm from trial participation | 13 |
| Dissemination policy: trial results | #31a | Plans for investigators and sponsor to communicate trial results to participants, healthcare professionals, the public, and other relevant groups (eg, via publication, reporting in results databases, or other data sharing arrangements), including any publication restrictions | 21 |
| Dissemination policy: authorship | #31b | Authorship eligibility guidelines and any intended use of professional writers | 22 |
| Dissemination policy: reproducible research | #31c | Plans, if any, for granting public access to the full protocol, participant-level dataset, and statistical code | 22 |
| **Appendices** | | | |
| Informed consent materials | #32 | Model consent form and other related documentation given to participants and authorised surrogates | Appendix 3 |
| Biological specimens | #33 | Plans for collection, laboratory evaluation, and storage of biological specimens for genetic or molecular analysis in the current trial and for future use in ancillary studies, if applicable | 16 (manuscript); Appendix 3: STAREE protocol V3.0 |

None The SPIRIT Explanation and Elaboration paper is distributed under the terms of the Creative Commons Attribution License CC-BY-NC. This checklist can be completed online using https://www.goodreports.org/, a tool made by the EQUATOR Network in collaboration with Penelope.ai

For peer review only
